# The genome of oil-Camellia and population genomics analysis provide insights into seed oil domestication

**DOI:** 10.1186/s13059-021-02599-2

**Published:** 2022-01-10

**Authors:** Ping Lin, Kailiang Wang, Yupeng Wang, Zhikang Hu, Chao Yan, Hu Huang, Xianjin Ma, Yongqing Cao, Wei Long, Weixin Liu, Xinlei Li, Zhengqi Fan, Jiyuan Li, Ning Ye, Huadong Ren, Xiaohua Yao, Hengfu Yin

**Affiliations:** 1 State Key Laboratory of Tree Genetics and Breeding, Research Institute of Subtropical Forestry, Chinese Academy of Forestry, Zhejiang, 311400 Hangzhou China; 2 Key Laboratory of Forest Genetics and Breeding, Research Institute of Subtropical Forestry, Chinese Academy of Forestry, Zhejiang, 311400 Hangzhou China; 3 College of Information Science and Technology, Nanjing Forestry University, Nanjing, 210037 China

**Keywords:** Oil-Camellia, Genome, Population genomics, Genome-wide association analysis, Oil biosynthesis, Domestication

## Abstract

**Background:**

As a perennial crop, oil-Camellia possesses a long domestication history and produces high-quality seed oil that is beneficial to human health. *Camellia oleifera* Abel. is a sister species to the tea plant, which is extensively cultivated for edible oil production. However, the molecular mechanism of the domestication of oil-Camellia is still limited due to the lack of sufficient genomic information.

**Results:**

To elucidate the genetic and genomic basis of evolution and domestication, here we report a chromosome-scale reference genome of wild oil-Camellia (2.95 Gb), together with transcriptome sequencing data of 221 cultivars. The oil-Camellia genome, assembled by an integrative approach of multiple sequencing technologies, consists of a large proportion of repetitive elements (76.1%) and high heterozygosity (2.52%). We construct a genetic map of high-density corrected markers by sequencing the controlled-pollination hybrids. Genome-wide association studies reveal a subset of artificially selected genes that are involved in the oil biosynthesis and phytohormone pathways. Particularly, we identify the elite alleles of genes encoding *sugar-dependent triacylglycerol lipase 1*, *β-ketoacyl-acyl carrier protein synthase III*, and *stearoyl-acyl carrier protein desaturases*; these alleles play important roles in enhancing the yield and quality of seed oil during oil-Camellia domestication.

**Conclusions:**

We generate a chromosome-scale reference genome for oil-Camellia plants and demonstrate that the artificial selection of elite alleles of genes involved in oil biosynthesis contributes to oil-Camellia domestication.

**Supplementary Information:**

The online version contains supplementary material available at 10.1186/s13059-021-02599-2.

## Background

Genomic information plays a fundamental role in crop improvement programs, and large-scale population genomics analyses based on a range of genetic resources provide accurate information for identifying genomic variations underlying the selection of desirable traits [1–3]. Oil-Camellia, in a broad sense, refers to more than 60 shrubs of the genus *Camellia* (Theaceae) whose seed kernels produce high-quality edible oils [4]. Currently, *Camellia oleifera* is the dominant species that is cultivated for Camellia oil production in China [5, 6].

Clearly, the human selection process results in substantial morphological and metabolic alterations that distinguish common cultivars from their wild ancestors [1]. The usage of oil-Camellia for edible oil has a long history (over 2300 years) in China [7], and the content and quality of seed oil have been continuously selected as the primary targets of breeding programs [7]. Compared to their wild progenitors, cultivated oil-Camellia plants often have larger fruit and thinner pericarp, allowing for a boost of seed oil yield. To date, oil-Camellia is cultivated extensively as an oil crop in many countries, including China, The Philippines, India, Japan, Brazil, Thailand, and South Korea [8, 9].

Camellia oil is unique in its chemical composition and medical and healthcare functions. It is rich in unsaturated fatty acids (which account for more than 90% of the total oil), of which the monounsaturated fatty acid, oleic acid, contributes to about 80% of the total oil content [10]. Because the fatty acid composition is very similar to that of olive oil, Camellia oil is also known as the “oriental olive oil” [7]. It also contains tea polyphenol, squalene, and other bioactive substances [11]. Long-term consumption of Camellia oil is beneficial for treating cardiovascular and cerebrovascular diseases and reducing the level of blood cholesterol [11, 12].

To meet the growing demand for high-quality edible oil, the significant problem of oil crop development is to accelerate the breeding process with a high accuracy and efficiency. Although the biochemical pathways of oil biosynthesis have been extensively characterized in many plants [13–16], the molecular basis of the domestication of oil biosynthesis in trees is limited. Advances in olive genomics have revealed novel findings on the oil biosynthesis pathway and provided valuable genomic resources for the genetic improvements in oil crops [17]. In particular, comparative analyses of olive and sesame have uncovered the functional divergence of key oil biosynthesis genes, such as *fatty acid desaturase 2* (*FAD2*), *stearoyl-acyl carrier protein desaturases* (*SADs*), and *enoyl- acyl carrier protein (ACP) reductase* [17].

As a perennial tree crop, oil-Camellia tends to be outcrossed with a long juvenile phase, which makes the breeding process more complex than annual crops [7]. Accelerating the breeding of varieties with increased yield and quality remains challenging due to the lack of genomic information and complex genetic background. Here we present a chromosome-level reference genome of wild *C. oleifera* through an integrative assembly approach of combining Pacific Biosciences (PacBio) sequencing, 10X Genomics sequencing, BioNano DLS optical mapping, and high-resolution chromosome conformation capture (Hi-C) mapping technologies. We also constructed a high-density genetic linkage map using an F1 population consisted of 180 progenies, which provides high-density molecular markers for genetic breeding. Furthermore, we performed transcriptome sequencing on 221 *C. oleifera* accessions and uncovered novel genetic variations associated with seed oil traits. We have shown that a subset of key genes involved in oil biosynthesis are under the selection process. By combining gene expression analysis and genetic variation association analysis, we revealed elite combinations of allelic variations that contribute to the domestication of seed oil. This work reveals pivotal genetic variations underlying the domestication of seed oil and provides insights into genetic improvements in tree breeding.

## Results

### Genome sequencing and assembly of the diploid oil-Camellia genome

To obtain a high-quality reference genome of oil-Camellia, we performed flow cytometry analysis to investigate the ploidy of wild species and major cultivars of oil-Camellia. We found that all cultivars assessed were hexaploid or tetraploid (Additional File 1: Table S1); and the *C. oleifera* var. “Nanyongensis” (CON)—previously identified as a wild progenitor of cultivated oil-Camellia—was revealed as diploid and selected for genome sequencing analysis (Additional File 2: Fig. S1A and Additional File 1: Table S1). The karyotype analysis supported that the CON plant was diploid with 30 chromosomes (2*n* = 2*x* = 30; Additional File 2: Fig. S1B). To further evaluate the genome complexity, we examined the genome based on short sequencing reads. The CON genome was estimated to be 2.95 Gb in size and was highly heterozygous (estimated heterozygosity (Het) = 2.52%], with 76.1% repetitive sequences (Additional File 2: Fig. S2).

**Fig. 1:**
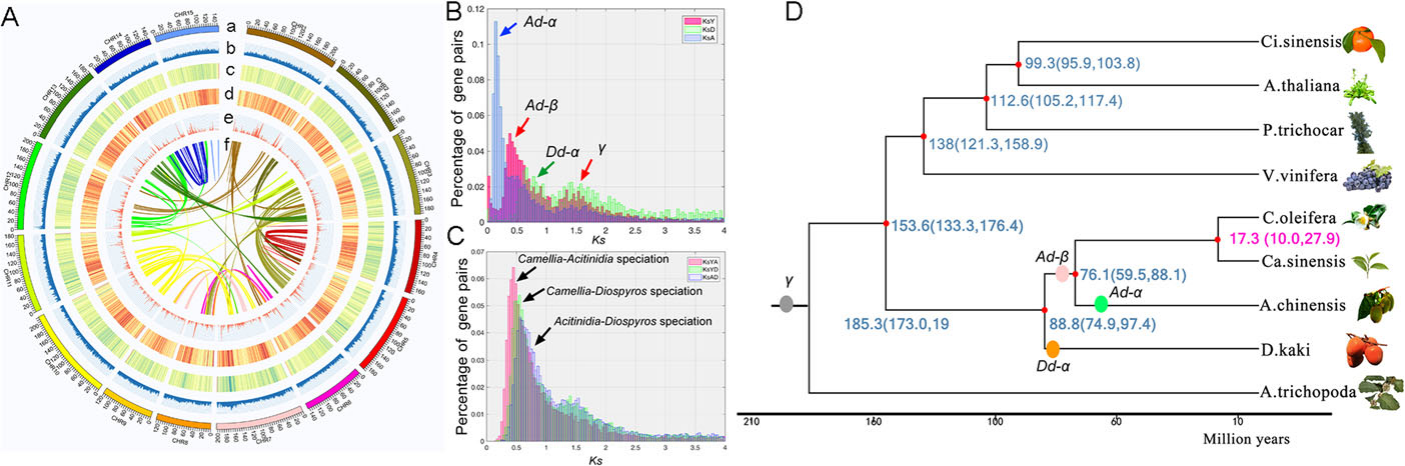
The landscape of genome structure and evolutionary analyses of the CON genome. **A** A circular representation of the characteristics of the assembled CON genome. The different layers of circles are listed: (a) pseudo-molecules of oil-Camellia chromosomes; (b–e) the distribution of GC density, repetitive elements density, gene models, and non-coding RNA genes. (f) the syntenic blocks of the CON genome. **B** Distribution of silent divergence rates (Ks) between gene pairs within the *Diospyros* (KsD), *Actinidia* (KsA), and CON genomes (*Camellia*, KsY). Camellia shows two peaks, indicated by red arrows, which are corresponding to the Ad-β and γ duplication. The Ad-α and Dd-α duplication that are found in *Actinidia* and *Diospyros* are revealed by blue and green arrows. **C** The Ks distribution of orthologous genes between *Camellia-Actinidia* (KsYA), *Camellia-Diospyros* (KsYD), and *Acitinidia-Diospyros* (KsAD); the peaks (indicated by the black arrows) indicate the speciation events. **D** A time-calibrated phylogenetic tree of related plant species. The whole-genome duplication events are indicated by the colored circles. The divergent time of branches is revealed by the numbers on each branch. Ci.sinensis, *Citrus sinensis*; A.thaliana, *Arabidopsis thaliana*; P.trichocarpa, *Populus trichocarpa*; V.vinifera, *Vitis vinifera*; C.oleifera, *Camellia oleifera*; Ca.sinensis, *Camellia sinensis*; A.chinensis, *Acitinidia chinensis*; D.kaki, *Diospyros kaki*; A.trichopoda, *Amborella trichopoda*. Grey circle, the γ triplication event; Orange circle, the Dd-α duplication; Green circle, the Ad-α duplication; Magenta circle, the Ad-β duplication.

To construct a high-quality reference genome, we combined various sequencing techniques, including PacBio sequencing, BioNano sequencing, 10X Genomics sequencing and Hi-C technologies (detailed pipeline of assembly was described in Additional File 2: Fig. S3). Based on this hybrid assembly approach, our de novo genome assembly yielded 4,075 contigs with an N50 length of 1.002 Mb; the Hi-C analysis obtained a genome size of 2.89 Gb with 2,143 scaffolds and an N50 length of 185.36 Mb, consisting of 91.33% of the entire assembled genome into 15 pseudo-chromosomes (Fig. 1A, Additional File 1: Table S2 and Additional File 2: Fig. S4).

### Annotation and analysis of the CON genome

**Fig. 2:**
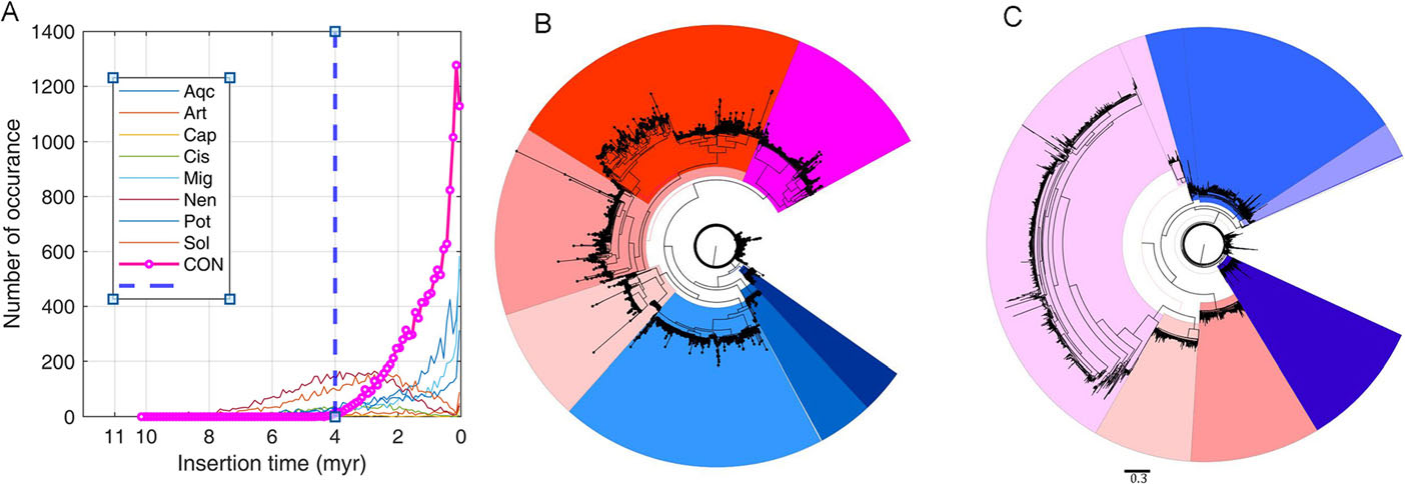
Comparative analyses of LTR elements. **A** The age distribution (LTR insertion time) of CON genome and other higher plants. The dashed line indicates the timepoint of 4 million years. Aqc, *Aquilegia coerulea*; Art, *Arabidopsis thaliana*; Cap*, Carica papaya;* Cis, *Citrus sinensis*; Mig, *Mimulus guttatus*; Nen, *Nelumbo nucifera*; Pot, *Populus trichocarpa*; Sol, *Solanum lycopersicum*; CON, *Camellia oleifera*. The genome sequences of other plant species are derived from Phytozome v12.1.6. **B**, **C** The phylogenic analysis of the Copia and Gypsy LTR elements in the CON genome. Different color shades indicate the expansion of the LTR subfamilies.

The assembled genome contained approximately 69% repetitive DNA, of which 43.64% was long terminal repeat retrotransposon elements (LTR-TE; Additional File 1: Table S3). Copia and Gipsy were the major LTR-TE accounting for 30.12% of the repetitive elements, which was in good agreement with the tea genome [18]. Comparative analysis of TE families showed that the CON genome had predominantly young LTRs (Fig. 2), suggesting a prominent role of genome evolution in oil-Camellia.

We annotated the assembled genome through combining three different approaches: ab initio prediction, homology-based prediction, and transcriptome alignment. In total, we identified 42,426 gene models with a mean gene length of 3,955 bp (Additional File 1: Table S4 and Additional File 1: Table S5). The coding sequences were further annotated through alignments with five different databases; 37,565 protein-coding sequences were revealed, of which 7938 were identified in all databases (Additional File 1: Table S6). Benchmarking Universal Single-Copy Orthologs (BUSCO) analysis revealed that the complete BUSCOs are 90.1% which included 81.3% complete single-copy BUSCOs (Additional File 1: Table S7).

### Genome duplication and evolution

To investigate the evolution of the CON genome, we performed gene family analysis using various plant species. Our analysis of syntenic ortholog pairs revealed two whole-genome duplication (WGD) events: the old gamma, and the *Ad-β* duplication before the diversion of *Actinidia* and *Camellia* (Fig. 1A, B). The comparisons of synonymous mutation rate (*Ks*) between *Camellia*-*Actinidia*, *Camellia*-*Diospyros,* and *Actinidia*-*Diospyros* supported the evolutionary history of Ericales (Fig. 1C). The results indicated that genus *Camellia* lacked a lineage-specific WGD event, which was consistent with previous studies [19]. Furthermore, phylogenetic analysis using single-copy orthologs supported the taxonomic placement of the genus *Camellia*; and time-calibrated analysis revealed that CON diverted from *Camellia sinensis* 17.3 million years ago (Fig. 1D).

### Construction of a genetic linkage map using the cross-population of oil-Camellia cultivars

Based on the draft CON genome sequence, a cross-population of two oil-Camellia cultivars (*C. oleifera* cv. “ChangLin 53” and “ChangLin 81”) was examined by double digest restriction site-associated sequencing (ddRAD) analysis to construct a genetic linkage map. In total, 182 ddRAD libraries (including 180 F1 individuals and two parents) were sequenced, which generated approximately 657.84 Gb data (Additional File 1: Table S8). This resulted in a total of 250,715 valid single nucleotide polymorphism (SNPs) with predictable segregation patterns that were subjected to genetic linage construction using the double pseudo-testcross strategy (Additional File 1: Table S9 and Data S1 [20, 21]) [22]. We further extracted the SNPs that were in accord with the reference CON genome to construct the linkage map (Additional File 2: Fig. S5). The genetic map consisted of 15 linkage groups (LGs) and covered a total of 1937.22 cM with an average interlocus distance of 6.46 cM. The genetic length of each LG ranged from 56.998 cM (LG14) to 200.088 cM (LG07) with an average interlocus distance of 4.071–10.659 cM (Additional File 2: Fig. S5 and Additional File 1: Table S10). This linkage map is valuable and informative for locating the key genetic loci involved in the domestication of economic traits in oil-Camellia.

### Phenotypic and genetic characterizations of the population for genome-wide association analysis

To evaluate the domestication of seed oil traits, a naturally distributed population, containing 221 accessions collected from different areas of China (Additional File 1: Table S11), was examined. We investigated eight important oil traits of all accessions for three consecutive years (Additional File 1: Table S12). We found that the oil traits varied extensively (Additional File 1: Table S13) and followed an approximately normal distribution (Additional File 2: Fig. S6). Particularly, a significant negative correlation (*R* = − 0.95) was revealed between the oleic acid content and linoleic acid content (Additional File 2: Fig. S6).

**Fig. 3:**
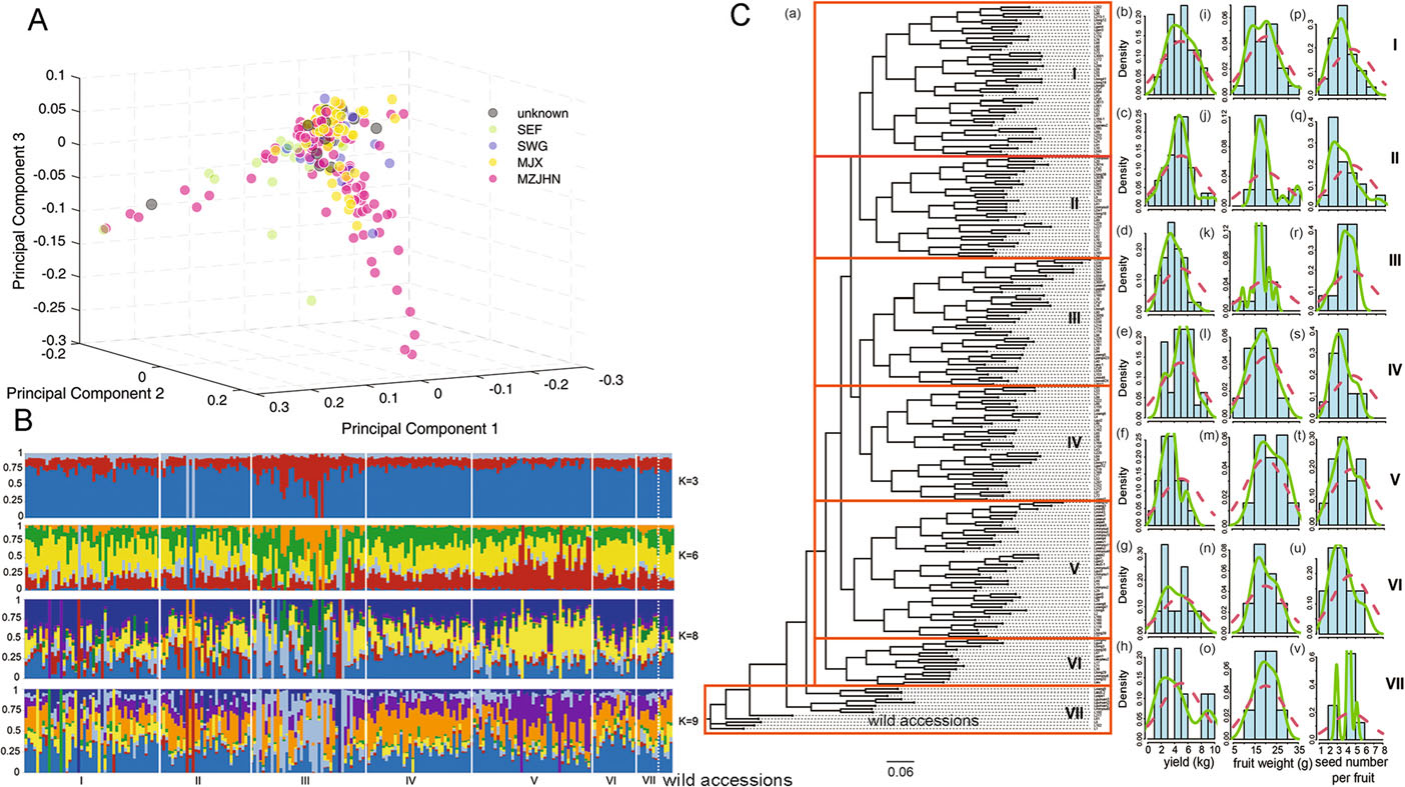
Genetic architecture and analysis of the population of oil-Camellia cultivars. **A** The PCA analysis of *C. oleifera* accessions. Different color of points indicates the origins of the cultivars that are collected from different areas of China. Source data are provided as Table S20. SEF, South East of Fujian; SWG, South West of Guangxi; MJX, Middle of Jiangxi; MZJHN, Middle of Zhejiang and Hunan. **B** The population structures of 221 accessions in association population by admixture analysis. Numbers in the *y*-axis indicate the membership coefficient. Each bar on the *x*-axis represents an accession; colored segments within one bar reflect the proportional contributions of each subpopulation to this individual. The subpopulations are separated by dashed lines. The black bracket indicates the wild accessions. **C** The phylogenetic analysis and phenotypic evaluation of 221 *C. oleifera* accessions. (a) The seven red frames are the seven subpopulations defined based on the phylogenetic tree. (b–h) are the distributions of average fruit yield of per tree (kg) for the seven subpopulations, respectively. (i–o) are the distributions of average weight per fruit (g) for the subpopulations, respectively. (p–v) are the distribution of seed number per fruit for the subpopulations, respectively

We performed transcriptome sequencing of developing kernels of the 221 *C. oleifera* accessions to identify genetic variations associated with oil traits. In total, we obtained 1.84 Tb of clean data containing 12,252 million clean reads (average 8.3 Gb per individual; Additional File 1: Table S11). We aligned the clean reads to the reference CON genome; the average of mapping rate was 82.15%. After the filtering process, the expression levels of transcripts were evaluated (Data S2 [20, 21]). To mitigate the false positives, we used a stringent filtering process for the SNP calling, including different filters regarding read coverage and the SNP call rate (see Materials and Methods for details). In total, we identified 1,849,953 SNPs (Additional File 1: Table S14) and 85,440 short genomic insertions and deletions (InDels; Additional File 1: Table S15), 59.80% of SNPs located in intergenic regions and 19.64% were in the exon regions. To assess the genetic diversity of the population, a core set of 25,581 SNPs [Minor allele frequency (MAF) ≥ 0.05, Het ≤ 0.8, linkage disequilibrium (LD) ≤ 0.03] were obtained for further analyses. We showed there was substantial genetic diversity among the accessions (Fig. 3); only a few accessions collected from the same regions were grouped together, and most of the accessions from different regions were mixed together in the phylogenetic tree (Fig. 3A, C). Maximum likelihood (ML) phylogeny analysis revealed that most *C. oleifera* accessions harboring similar phenotypes were clustered together in the phylogenetic tree, including fruit yield, fruit weight, and number of seeds per fruit (Fig. 3C, Additional File 1: Table S16 and Additional File 2: Fig. S7). Based on the phylogenetic and population structure analysis (Fig. 3B), the 221 accessions were divided into seven subpopulations (Fig. 3C). All seven subpopulations showed rapid LD decay, and the SNPs were in linkage equilibrium when the distances were over 1.0 kb (*r*^2^ < 0.15; Additional File 2: Fig. S8 and Data S3 [20, 21]). These results indicated that the *C. oleifera* cultivars were mainly distinguished by their morphological characteristics rather than their geographic origin.

To identify candidate regions potentially affected by domestication, the nucleotide diversity (*π*), nucleotide diversity ratio (*π* ratio), and population fixation index (*Fst*) were calculated using the wild accessions as the control group (Fig. 3C). In total, we detected 2156 artificially selected windows, corresponding to 522 selection regions (Data S4 [20, 21] and Additional File 2: Fig. S9). The size of the selected genomic regions ranged from 100 kb to 260 kb; and there were 19 to 54 regions distributed among the chromosomes (Data S4 [20, 21] and Additional File 2: Fig. S9B). We obtained 1000 genes within the selected regions (Data S5 [20, 21]) and performed the functional enrichment analyses. We revealed that there were 163 significantly enriched Gene Ontology terms, including the pathways involved in stress response, hormone biosynthesis, response to light, and lipid metabolic process (Additional File 1: Table S17). These results highlight the potential molecular alterations that shape the cultivated oil-Camellia population.

### Integrative association analyses reveal the candidate genes underlying oil domestication

**Fig. 4:**
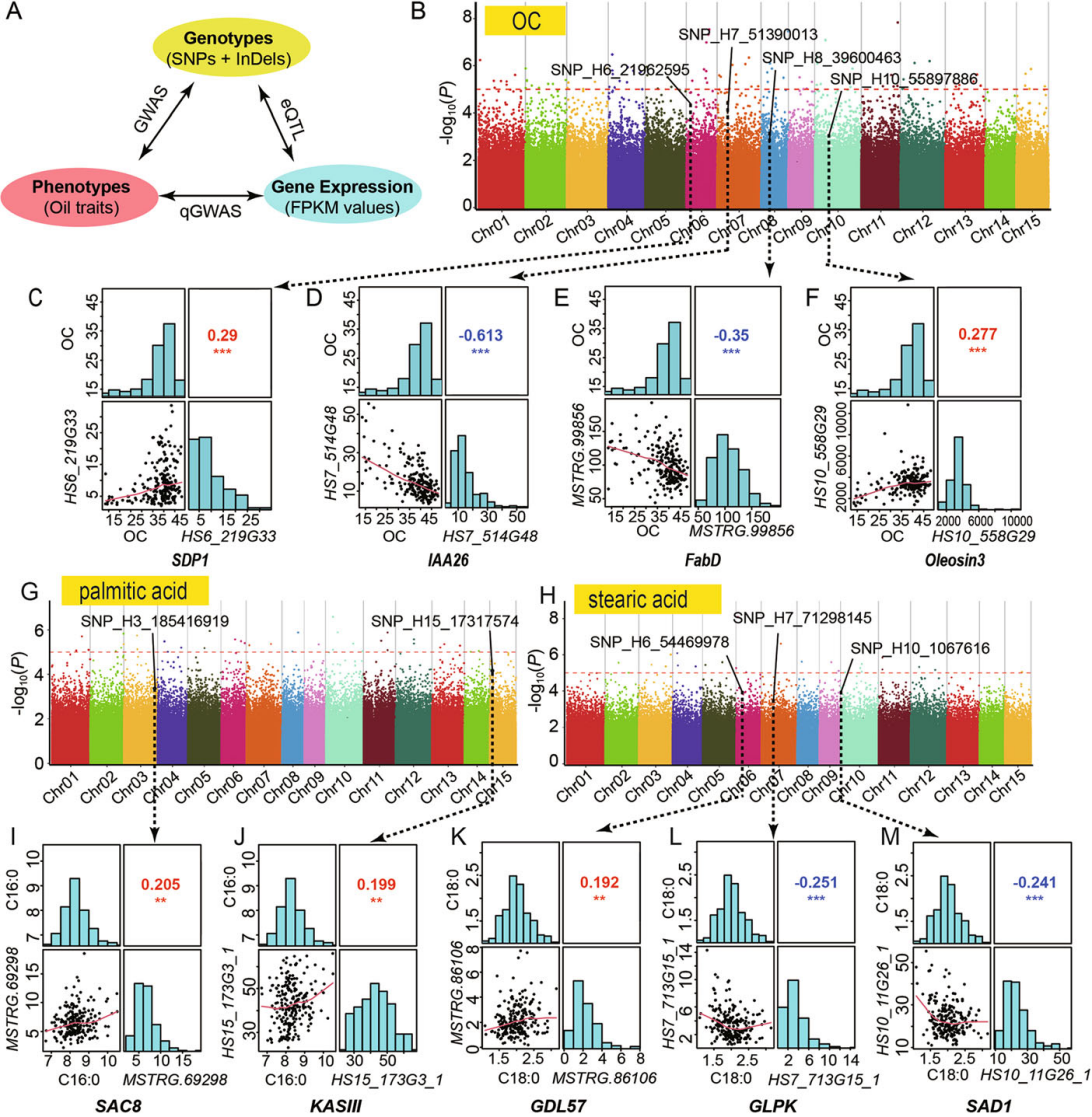
Genome-wide identification and annotations of candidate genes involved in the seed oil domestication. **A** The strategy of association analyses in this study. Three categories of association analyses are performed including GWAS, qGWAS, and eQTL. **B**, **G**, **H** are the Manhattan plots displaying the GWAS results for OC, palmitic acid content and stearic acid content, respectively. The y-axis is the –log_10_(*P* values). Each point represents a molecular marker. Horizontal dashed lines indicate the significance level of *P* value = 10E-05. The labeled SNP loci are the candidate genes involved in the oil biosynthesis genes. **C**, **D**, **E**, **F** are corresponding to the four candidate genes, respectively to *SDP1*, *IAA26*, *FabD*, and *Oleosin3*), that are involved in the OC selection. **I**, **J** are two candidate genes (*SAC8* and *KASIII*) involved in the palmitic acid content selection. **K**, **L**, **M** are three genes (*GDL57*, *GLPK*, and *SAD1*) involved in the stearic acid content selection. In each panel (including **C**-**F** and **I**-**M**), The distribution of corresponding traits and gene expression are shown on the diagonal plots. To the bottom left were the bivariate scatter plots with best fit lines displayed. Correlation coefficients are shown above the diagonal. “***,” “**,” and “*” denote significance with *P* values of 0.001, 0.01, and 0.05, respectively. Red and blue denote the positive and negative correlations, respectively.

To investigate the genetic variations underlying the domestication process, we performed three layers of analyses for mining candidate genes involved in the selection of oil traits: (1) association analyses between oil traits and genetic variations (genome-wide association study, GWAS), (2) correlation analyses between oil traits and transcript expression (quantitative GWAS, qGWAS), and (3) association analyses between transcript expression level and genetic variations (expression quantitative trait loci (eQTL) analysis; Fig. 4A). Using the GWAS analysis, a total of 342 loci significantly associated with one or several oil traits (Fig. 4B, G, H and Additional File 1: Table S18), containing 711 genes located within the 100 kb region, were identified. Based on the qGWAS, 204 genes whose expression levels associated with one or several oil traits (Additional File 1: Table S19) were identified. eQTL analysis uncovered a total of 9001 transcripts as cis-eQTLs and 6,548,567 trans-eQTLs (Data S6 [20, 21]). Kyoto Encyclopedia of Genes and Genomes (KEGG) enrichment analyses of cis- and trans- eQTLs revealed that the lipid metabolism pathway was significantly enriched (Additional File 1: Table S20). Further, gene co-expression network analysis identified gene modules that were correlated with oil trait changes, which was in agreement with the eQTL analysis (Additional File 2: Fig. S10).

**Fig.5:**
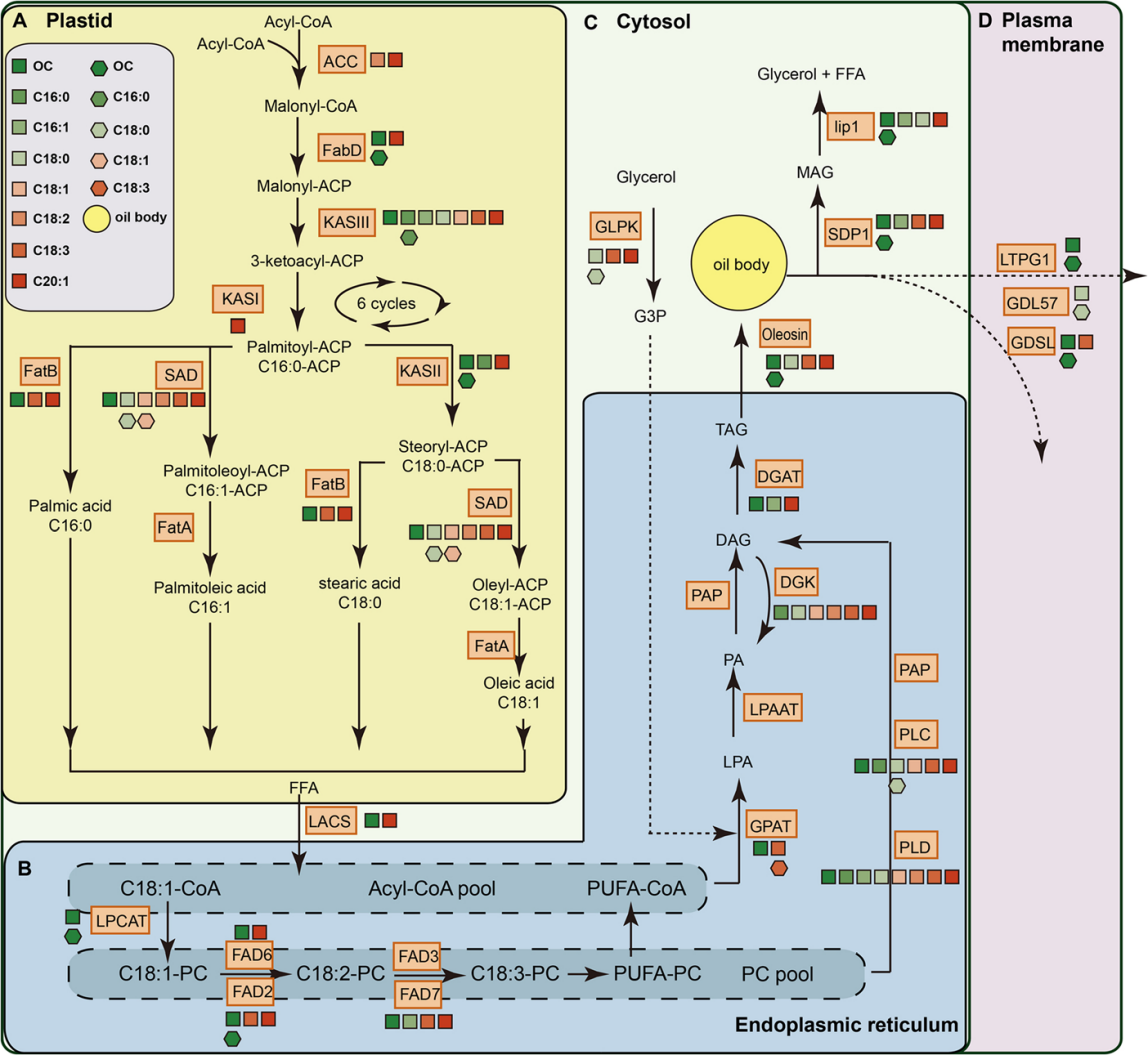
A proposed molecular diagram of the metabolic pathway comprising of the genes associated with ORTs in *C. oelifera.* The biochemical process of enzymes and functions are on the basis of previous reports [23, 24]. **A** The de novo biosynthesis process of free fatty acids in plastid; **B** fatty acids modification, acyl editing and TAG assembly in endoplasmic reticulum; **C** and **D** oil storage, transportation and breakdown in cytosol and plasma membrane. The key enzymes of the oil metabolism pathway are indicated in the light red boxes. The squares and hexagons are indicating the identification of genes in GWAS or qGWAS, respectively. Colored squares and circles are presenting the significant associations of different oil traits. The yellow circle indicates the oil body. C16:0, palmitic acid; C16:1, palmitoleic acid; C18:0, stearic acid; C18:1, oleic acid; C18:2, linoleic acid; C18:3, linolenic acid; C20:1, cis-11-eicosenoic acid; Acyl-CoA, acyl-coenzyme A; ACC, acetyl-CoA carboxylase; FabD, malonyl CoA:ACP transacylase; KAS I, II, III, β-ketoacyl-[acyl carrier protein] synthase I, II, III; SAD, stearoyl ACP desaturase; FatA and FatB, fatty acyl-ACP thioesterase A, B; FFA, free fatty acid; LACS, long-chain acyl-CoA synthase; LPCAT, acyl-CoA: lysophosphatidylcholine acyltransferase; PC, phosphatidylcholine; FAD2 and FAD6, fatty acid Delta-12 desaturase; FAD3 and FAD7, fatty acid Delta-15 desaturase; PUFA, polyunsaturated fatty acid; G3P, glycerol-3 phosphate; GPAT, G3P acyltransferase; LPA, lyso-phosphatidic acid; LPAAT, LPA acyltransferase; PA, phosphatidic acid; PAP, phosphatidic acid phosphatase; DAG, diacylglycerol; DGAT, diacylglycerol acyltransferase; PLC and PLD, phospholipase C and D; DGK, diacylglycerol kinase; SDP1, sugar-dependent triacylglycerol lipase; DGL, diacylglycerol lipase; lip1, monoacylglycerol lipase; GLPK, Glycerol kinase; LTPG1, non-specific lipid transfer protein GPI-anchored 1; GDSL and GDL57, GDSL esterase/lipase

Combined analysis of GWAS and qGWAS discovered 21 high-confidence candidate genes, including 14 genes that were associated with total oil content in the dry kernel (OC; Table 1). We were surprised to find that of these 14 candidate genes, nine were involved in the lipid metabolism pathway (Fig. 5 and Table 1). Five genes revealed to be phytohormone-related transcription factors were also discovered as candidate genes of oil traits domestication (Table 1). To further evaluate the candidate genes, the expression levels of abovementioned candidates were investigated. An integrative analysis of all three layers identified *sugar-dependent triacylglycerol lipase* (*SDP1*), *auxin-responsive protein IAA26* (*IAA26*), *malonyl-CoA:ACP transacylase* (*FabD*), and *Oleosin3* as the artificially selected genes for the OC domestication (Fig. 4B–F), *phosphoinositide phosphatase SAC8* (*SAC8*) and *β-ketoacyl-ACP synthase III* (*KASIII*) for palmitic acid content (Fig. 4G, I, J), and *GDSL esterase/lipase* (*GDL57*), *glycerol kinase* (*GLPK*), and *stearoyl-ACP desaturases* (*SADs*) for stearic acid content (Fig. 4H, K, L, M).

**Table 1:** Candidate genes identified by combined analysis of GWAS and qGWAS

Chr	Gene id	Gene_length (bp)	qGWAS		GWAS		Symbol	Description
			FDR	*R*	SNP localization	*p* value		
Oil content								
6	HS6_219G33	6313	5.25E-06	0.29	21962595	1.35E-05	*SDP1*	Triacylglycerol lipase SDP1
7	HS7_514G48	5054	3.34E-14	− 0.613	51390013	3.14E-05	*IAA26*	Auxin-responsive protein
8	MSTRG.99856	4621	6.38E-05	− 0.3502	39600463	9.43E-04	*FabD*	Malonyl-CoA:ACP transacylase
10	HS10_561G6	914	1.02E-02	− 0.42	29454430	5.47E-04	*ERF5*	Ethylene response factor 11
15	HS15_46G25	935	7.05E-06	− 0.389	4624317	1.92E-05	*GDSL*	GDSL esterase/lipase At4g10955
10	HS10_219G24	4254	2.90E-05	− 0.358	21981975	3.79E-04	*LPCAT*	Acyl-CoA N-acyltransferases superfamily protein isoform 1
13	MSTRG.41142	7418	1.13E-04	− 0.357	179016485	6.73E-04	*FAD2*	Fatty acid desaturase
2	HS2_300G57	3614	1.87E-05	− 0.322	30098439	6.92E-06	*IAA14*	Auxin-responsive protein IAA14
11	HS11_422G6	953	7.68E-03	− 0.236	42215447	3.25E-05	*ERF4*	Ethylene-responsive transcription factor
5	MSTRG.81837	4601	1.19E-02	− 0.209	116227945	7.80E-04	*lip1*	Monoacylglycerol lipase
12	MSTRG.26275	17055	4.28E-02	− 0.136	9644861	8.96E-04	*LTPG1*	Non-specific lipid transfer Protein GPI-anchored 1
3	HS3_761G26	2574	3.89E-02	0.175	76145034	9.81E-06	*ARF17*	Auxin response factor 17-like
8	MSTRG.103039	5768	6.92E-03	0.2272	120840884	3.03E-04	*KASII*	Ketoacyl-ACP synthase II
10	HS10_558G29	422	4.95E-06	0.277	55897886	5.25E-04	*Oleosin3*	OleosinIII
Palmitic acid								
3	MSTRG.69298	11088	1.88E-02	0.205	185416919	7.11E-04	*SAC8*	Phosphoinositide phosphatase SAC8
15	HS15_173G3_1	7778	1.80E-02	0.199	17317574	6.99E-05	*KASIII*	Ketoacyl-ACP synthase III
Stearic acid								
6	MSTRG.86106	4700	3.02E-02	0.192	54469978	1.10E-04	*GDL57*	GDSL esterase/lipase
7	HS7_713G15_1	4379	1.38E-02	− 0.251	71298145	3.36E-04	*GLPK*	Glycerol kinase
10	HS10_11G26_1	3727	4.54E-02	− 0.241	1067616	1.02E-04	*SAD1*	Stearoyl-ACP desaturase
8	HS8_425G5_1	5137	1.49E-02	0.221	42575259	1.86E-04	*PLC1*	Phospholipase C
11	HS11_436G24_1	17347	2.48E-02	− 0.213	43619536	5.37E-04	*PLC6*	Phosphoinositide phospholipase C 6-like

### Discovery of the allelic variations in candidate genes involved in the seed oil domestication

Oil storage is a dynamic process that helps regulate the seed oil yield. We found that strong association signals of OC were mapped to the *Oleosin3* and *SDP1* loci (Fig. 4B). And the expression of both *Oleosin3* and *SDP1* was significantly correlated with OC (Table 1 and Fig. 4C, F). Oleosin plays a structural role in stabilizing lipid bodies during seed desiccation [25, 26], whereas SDP1 is involved in the breakdown of lipids [27]. Therefore, these results together suggest that the selection of oil yield requires a coordinated regulation of lipid biosynthesis enzymes. The Camellia oil is rich of oleic acid (about 80% of total oil), which is distinctive to other seed oils. Our association studies indicated that several selected enzymes were correlated with levels of palmitic acid and stearic acids (Table 1). *KASIII* and *SAD* are two major enzymes upstream of oleic acid biosynthesis (Fig. 5), that were potentially domesticated.

**Fig. 6:**
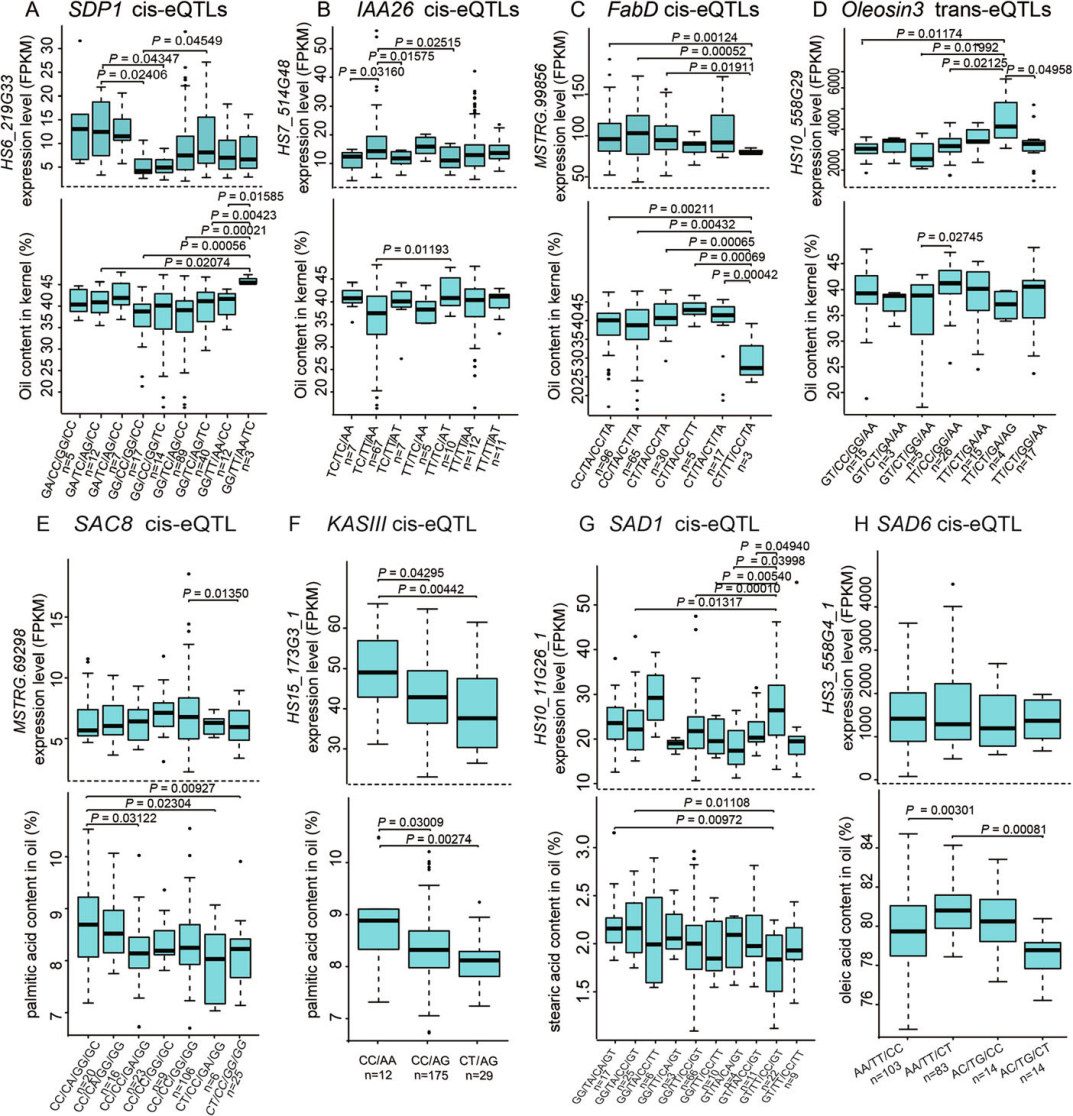
The evaluation of genotype groups of candidate genes involved in the domestication of oil-Camellia. **A** *SDP1*, **B** *IAA26*, **C** *FabD*, **D** *Oleosin3*, **E** *SAC8*, **F***KASIII*, **G** *SAD1*, **H** *SAD6*. The upper panel shows the distribution of expression level of candidate gene; and the lower panel shows the distribution of corresponding traits in different genotypes. In each barplot, the middle line of the box represents the median, the first (25%) and third (75%) quartiles are indicated by the lower and upper boundaries and the whiskers show the minimum and maximum values, excluding outliers. The black points outside the whiskers indicate the outliers. The *x*-axis indicates the genotypes and the number of individuals of each genotype

To further determine the effective allelic variation of candidate genes, we analyzed the individual and conjugated SNPs of candidate genes and their contribution to the variation of oil traits. We found eight candidate genes that possessed SNP combinations significantly associated with variations of oil traits and gene expression (Fig. 6). We showed that, in the cultivated population, some individuals bearing specific alleles of *SDP1*, *IAA26*, *FabD*, and *Oleiosin3* display significantly changes of OC and gene expression (Fig. 6A–D). Meanwhile, the specific alleles of *SAC8*, *KASIII,* and *SAD1/6* were related to the selection of fatty acid contents (Fig. 6E–H). We found that the CC/AA genotype group of *KASIII* contributed to the highest palmitic acid content and the gene expression (Fig. 6F). The AA/TT/CT of *SAD6* was potentially a favorable allele during the selection of high oleic acid content, despite no significant change of gene expression was identified among the different genotype groups (Fig. 6H). These results point to prominent alleles for the molecular breeding toward an improved oil trait of oil-Camellia.

To validate the key SNPs involved in the oil domestication, we have used the Sanger sequencing technology to validate the 23 SNPs located in the eight key candidate genes (Fig. 6) in the oil-Camellia cultivars. In total, we have obtained 3781 Sanger sequences (Data S7 [20, 21]) and detected 4696 successful SNP events (Additional File 1: Table S21). We find that all of the 23 identified SNPs are validated with high accuracies; the confirmation rates of SNPs are between 88.59 and 100% for each SNP with an overall accuracy rate of 97.76% (Additional File 1: Table S21). To evaluate the gene expression profiles, we performed the real-time reverse transcription PCR (qRT-PCR) analysis of the eight key candidate genes (in Fig. 6) to validate the expression levels. We showed that all tested genes displayed significant high correlations between the RNA sequencing (RNA-seq) and qRT-PCR results (Additional File 2: Fig. S11). These results indicate that the identification of SNPs and the gene expression analysis based on the large-scale RNA-seq analysis are highly confident and informative for the molecular breeding of oil-Camellia cultivars.

## Discussion

Genus *Camellia* includes more than 200 species and many of them have been domesticated as economically important crops [28, 29]. *C. sinensis* is the most important economic crop, providing world-popular non-alcoholic beverage from the leaf tissues [30]. The use and selection of oil-Camellia have a long history in China, which mainly focuses on seed oil composition and content. In this study, we reported the high-quality genome sequences of the diploid oil-Camellia (CON) plant and the genome-wide association analysis of identifying key genes involved in the domestication of oil biosynthesis. This presented genomic information of oil-Camellia can enhance understanding of the genetic and genomic characteristics for dissecting trait domestication underlying different selection programs (e.g., leaf metabolism in tea plant and seed oil biosynthesis in oil-Camellia) in closely -related plant species.

Our de novo construction of the oil-Camellia genome provides a high-quality reference genome for comprehensive comparative genomics and population genomics studies. Through an integrative assembly approach using multiple genomic sequences, the current assembly of the CON genome has a contig N50 over 1 Mb (Additional File 1: Table S2). And based on the Hi-C analysis, the genome anchors 15 pseudo-chromosomes with a scaffold N50 of 185.36 Mb, which is consistent with the karyotyping analysis (Additional File 2: Fig. S1B). We also construct a genetic linkage map using an F1 hybrid population of 180 individuals through next-generation sequencing, which gives rise to a whole-genome coverage of valid SNPs for genetic breeding studies (Additional File 2: Fig. S5). The comparative analyses reveal that, like other tea plants, oil-Camellia genome has been through two rounds of WGD events: one was the ancient common paleopolyploid (*γ*) event, and the more recent event was before the divergence of genus *Camellia* and genus *Actinidia* (Ad-*β*) [19]*.* Comparisons of orthologous genes of closely-related species also suggests that the occurrence of *Diospyros*-specific (Dd-*α*) duplication was slightly prior to the Ad-*β* duplication event. These results are consistent with previous analyses [31–33].

The diploid oil-Camellia genome is complex with high heterozygosity and a large proportion of repetitive elements, and these results are in good agreement with previous genomic studies of tea plants [19]. LTR-TE is found to be dominant among the repetitive elements, which potentially plays prominent contributions to the expansion of CON genome [34, 35]. The comparative analysis of insertion time among various plant species, as well as the molecular phylogeny analyses, also indicate that the LTR-TE of CON is undergoing rapid evolution with strong activity. These results together highlight the important role of repetitive elements in the evolution of complex plant genome.

Seed oil content and compositions have been the primary targets of the selection of oil-Camellia. We perform transcriptome sequencing of seed kernels of the GWAS population plants to determine the genetic bases of oil traits domestication. To circumvent the complex genetic backgrounds, we integrate genomic variation, gene expression, and trait variations within the population to mine high-confident candidate genes (Table 1). It is noteworthy that our integrative analysis uncovered that majority of genes (14 out of 21 candidates) are involved in the oil biosynthesis pathway. These results coincide with the oil-orientated domestication program in oil-Camellia. Meanwhile, two genes (*IAA26* and *ethylene response factor*) that are potentially involved in phytohormone signaling pathways were revealed as the regulatory genes (Table 1). It will be informative to further investigate the functions of different alleles in the regulation of seed oil biosynthesis in the future.

To find evidence of selection during the seed oil domestication, we evaluated the candidate genes containing the SNP combinations that are strongly associated with oil traits. We showed that elite alleles are strongly correlated with groups of individuals of different OC, including *SDP1*, which were shown to be selected during seed oil domestication [36]. Furthermore, *KASIII* and *SADs* were found to be under the selection of palmitic acid, stearic acid, and oleic acid content respectively, which were mapped to the plastid pathways of fatty acids biosynthesis (Fig. 5). Therefore, these analyses demonstrate the precision of the integrative approach.

Large-scale genetic analyses in crops have shown that domestication often fixes rare alleles and reduces the genetic diversity in cultivars [2, 37]. The evaluation of specific alleles of candidate genes allowed us to reveal favorable alleles that are under the selection program of oil-Camellia. We showed that the elite alleles, contributing to substantial changes of oil traits (Fig. 6; e.g., in the cases of *SDP1* and *FabD*), are relatively in a small proportion, suggesting an effect of genetic bottlenecks during the oil trait domestication. We also noticed that some genotype groups displayed inconsistent changes of oil traits and gene expression (Fig. 6), which indicated the interactive effects of SNPs within the candidate genes. Further examinations of different populations are needed to reveal the effects of multiple SNPs. This work reported the reference genome and associated resources of genetic variations in oil-Camellia, which will be informative to enhance the genetic improvement programs. The linkage map and the precise variations of candidate genes can contribute to applications of the molecular marker-assisted breeding and genomic selection.

## Conclusions

This study reports a high-quality chromosome-scale genome reference of *Camellia oleifera*, which provides fundamental information for comparative and evolutionary genomics analyses. The genome-wide association analysis of major oil-Camellia cultivars demonstrates that the artificial selection of rare but desirable alleles of genes involved in oil biosynthesis plays an important role in the oil-Camellia domestication process.

## Methods

### Plant materials

A wild progenitor of oil-Camellia was used for the genome sequencing study. The diploid progenitor CON, was obtained from the Guangxi Academy of Forestry (Additional File 2: Fig. S1A; 22°56′ N, 108°21′ E; Nanning, Guangxi Zhuang Autonomous Region, China). For DNA and RNA sample preparation, the plant materials were collected and put into liquid nitrogen immediately; samples were preserved in − 80 °C freezer before use.

To construct the *C. oleifera* linkage map, an F1 population, consisted of 180 progenies, was generated by a cross between cultivar “ChangLin NO.53” and cultivar “Changlin NO.81” in 2010; and “ChangLin NO.53” was employed as the female parent. The cross-population and their parents were preserved in the Dongfanghong Forest Farm (29°01′ N, 119°29′ E; Jinhua, Zhejiang, China).

For the GWAS, a population of 221 *C. oleifera* accessions, covering most of the natural distribution regions of *C. oleifera* in China, was used for sequencing and oil traits analyses. These 221 accessions were selected from a clonal plantation that includes a collection of 494 accessions of *C. oleifera*, which was maintained in Dongfanghong Forest Farm in 2004, using a randomized complete block design with three replications and seven plants per replication [10]. Detailed information of the 221 accessions was listed in Additional File 1: Table S11.

### Genome sequencing, de novo assembly, and annotation

The genomic DNA of CON was prepared by using the young leaves**.** Ten 20 kb de novo SMRTbell libraries were constructed according to the standard manufacturer’s protocol and used for SMRT PacBio genome sequencing. A total of 27,876,348 reads (total size of 320 Gb) were generated and used for initial assembly by the Falcon (v0.3.0) pipeline. The HaploMerger2 (v20180603) program (default parameters) was used to reduce the redundancy, and Arrow program with default parameters was used to correct the sequencing errors. Further, a total of 210 Gb clean data generated by the Illumina NovaSeq6000 platform was used to correct the PacBio reads.

To circumvent the high heterozygosity of the CON genome, a hybrid assembly of strategy was used to construct the high-quality reference genome. The details of the BioNano, 10X Genomics, and Hi-C sequencing procedures were described in the Additional File 3: Method S1; and the hybrid assembly approach was described in Additional File 2: Fig. S3. Finally, to construct a chromosome-scale reference genome, the Hi-C chromosomal interaction was created using HiC-pro [38] software (v2.5.0) (Additional File 2: Fig. S4).

The repetitive elements in the CON genome, including tandem repeats and interspersed repeats, were identified. Tandem repeats were discovered by Tandem Repeats Finder v4.07b. Interspersed repeats in the genome were identified using an integration of independent homology searching and de novo predictions (See details in Additional File 3: Method S2). Non-coding RNA genes (ncRNA) were annotated in this study (see details in Additional File 3: Method S2) and the results were shown in Additional File 1: Table S4.

We annotated the assembled genome through combining three different approaches: ab initio prediction, homology-based prediction, and transcriptome alignment. To obtain the transcriptome data, the total RNA of seven different tissues from CON was sequenced using the Illumina NovaSeq platform. The transcripts homolog prediction was performed initially by MAKER (v2.31.10); Augustus (v3.3.1) and SNAP (v2006-07-28) were used for de novo prediction. Finally, both homolog and de novo prediction results were integrated using MAKER and resulted in the final gene models (Additional File 1: Table S5; see details in Additional File 3: Method S2)

Functional annotation was achieved by comparing predicted proteins against public databases, including NCBI non-redundant protein sequences database (Nr), SwissProt (201709) [39], eggNOG [39], KEGG (v84) [40], Interpro (v5.16-55.0) [41], and GO [42] using Blast (v2.2.3) [43].

### Comparative and evolutionary analyses of the CON genome

A phylogenetic tree was constructed using 308 single-copy orthologous genes from nine different plant genomes. *Ks*-based age distributions of CON were also constructed to unveil WGD events in CON [44]. MUMmer 4.0 [45] was used to identify synteny with other species (i.e., *Amborella trichopoda*, *Arabidopsis thaliana*, *Vitis vinifera*, *Camellia sinensis*, *Citrus. sinensis*, *Populus trichocarpa*, *Actinidia chinensis*, and *Diospyros kaki*). The divergence time among species was inferred using the Bayesian Markov-chain Monte Carlo tree (MCMCTree) package in PAML [46], the expansion and contraction of orthologous gene families were measured using the software CAFÉ 4.2 (https://github. com/hahnlab/CAFE). Circos [47] was used to produce a circular visualization of the CON genome features. Additional information is provided in Additional File 3: Method S2.

### The construction of a linkage map of *C. oleifera*

To construct a high-density linkage map of *C. oleifera*, young leaves were harvested from the F1 individuals and their parents for DNA extraction and ddRADseq. After trimming the low-quality and contaminant sequences using Trimmomatic (v0.32) [48], the clean data was mapped to the reference CON genome using BWA [49]. The calling of SNPs and InDels were performed using GATK4 [50]. The SNP data was further filtered and only the SNP markers with the suitable segregation patterns were used for the genetic map using the double pseudo-testcross strategy (Data S1 [20, 21]) [22]. JoinMap4.1 [51] was used to calculate the marker order and genetic distance. The linkage group (LG) assignments were made according to the alignment results of clean reads (covering the markers) to the reference CON genome. A graphic representation of the map was generated using a custom Perl script (http://github.com/Niuyongchao/Fish_linkage_map). (See details in Additional File 3: Method S3)

### Characterization of oil traits in the *C. oleifera* population for association studies

Key oil traits of *C. oleifera* were measured for all accessions in the association population with at least three ramets per genotype per year for three years. The eight oil traits were OC, palmitic acid content, palmitoleic acid content, stearic acid content, oleic acid content, linoleic acid content, linolenic acid content and cis-11-eicosenoic acid content. The detailed sampling and measurement methods were reported previously [10]. The phenotypic normal fitting, variance, and Pearson’s correlation coefficients (*r*) for the eight quantitative traits were calculated by Data Processing System (DPS v14.50; http://www.chinadps.net/dps_eng/) [52] and the pairs function in R (https://www.r-project.org/).

### RNA sequencing and variant calling of *C. oleifera* association population

In previous study, it has been shown that the seed kernel accumulates lipids during the maturation of seed (between 294 and 324 days after fertilization) [53]. Based on this, we collected the samples around 305 days after fertilization for each accession in the association population; and RNA-seq was performed on Illumina HiSeq 4000 platform. After filtering the low-quality sequences, the high-quality reads were aligned to the reference CON genome using HISAT2 [54]. The new transcripts with sequence length over 200 bp were identified by Cufflinks v2.1.1 (Data S8 [20, 21]) [54]. The SNP calling was performed by GATK4 [50] after removing the reads without match region. The detailed SNP calling and filtering process is as follows: (1) Variant calling was performed for all samples using “UnifiedGenotyper” function of GATK4 with option “-stand_call_conf 10.0,” “-min_base_quality_score 17,” and “-stand_emit_conf 30.0” (which are the defaults values), generating one gVCF file for all sample. (2) To remove false variants, biallelic SNPs were initially extracted using the “SelectVariant” function in GATK4, and the variants were filtered using GATK’s VariantFiltration with option: -Window 4, -filter “QD < 4.0, FS > 60.0, MQ < 40.0”, -G_filter “GQ < 20”. (3) SNPs with minor allele frequency (MAF) < 5% and genotype call-rate < 50% in the population were discarded.

Cufflinks (v2.1.1) was used to calculate the transcripts expression levels of the 221 accessions using the fragments per kilobase of transcript per million fragments (FPKM) method [55]. After removing the transcripts with FPKM < 2 in over 70% accessions, the FPKM values of highly expressed transcripts were used to perform qGWAS and eQTL analysis.

### Population genetic analysis of the association population

The high-quality SNPs were further filtered by PLINK software [56] to mitigate the effect of LD with the parameter: --indep-pairwise 1000 5 0.03. Only SNPs with Het ≤ 0.8 and LD ≤ 0.03 were used to perform principal component analysis (PCA), population structure analysis, and phylogenetic analysis of association population. PCA was conducted using GCTA (v1.25.2) [57], and the first two components were plotted (Additional File 2: Fig. S7). The population structure was analyzed using ADMIXTURE [58]. A MLtree was constructed using SNPhylo [59] to clarify the phylogenetic relationships of the 221 accessions in the population, and the tree was visualized in the Figtree (http://tree.bio.ed.ac.uk/software/figtree/). LD decay was defined as the physical distance between SNP sites with *r*^*2*^ < 0.15 (Additional File 2: Fig. S8). The *r*^*2*^ values were evaluated by nonlinear regression analysis using PopldDecay (v3.40) [60].

### Genome-wide selective sweep analysis

Selective sweep analysis was performed to reveal the genomic signatures of domestication. The *π*, *π* ratio, and *Fst* were calculated for the control group (wild accessions in the Group VII in Fig. 3C) and the cultivated population group using PopGenome [61,62] through a 100-kb sliding window pipeline with a step size of 10 kb. The windows with high values of *π* ratio and *Fst*, representing the top 5% of all windows, were determined as the artificially selected windows. The consecutive selection windows were combined as the selection regions in a chromosome. The candidate genes in the selection regions were retrieved and subjected to the GO enrichment analysis using the hyper-geometric distribution test.

### Transcriptome-wide association analysis

An appropriate statistical model can reduce spurious genotype–phenotype associations and increase statistical power [63]. In this study, four analysis models were used to test the statistical association between genetic variants (SNPs and InDels) and eight oil traits in TASSEL (v5.2.24) [64], and the optimal model mixed linear model (MLM) was confirmed for each trait by comparing the expected and observed *P* values in QQ plot (Additional File 2: Fig. S12). The first ten components in the PCA results were used as the population structure matrix (*Q*; Additional File 1: Table S22), and the pairwise relatedness kinship (*K*) matrix within the association population was assessed by GCTA (v1.25.2) [57]. The *P* value was calculated for each association, and the significant *P* value threshold was set to 1.0E-03 [65–67].

To detect the candidate transcripts for oil traits, T-statistic analysis was implemented between the gene expression (FPKM values) and phenotypes by MatrixEQTL (v2.2; https://cran.r-project.org/web/packages/MatrixEQTL/index.html) [68]. Significance was set at *FDR* ≤ 0.05.

eQTLs were detected by the association analysis of genotypes and gene expression. In this study, eQTL analysis was performed using the same method as the SNP (InDels)-based association analysis for oil traits by MatrixEQTL (v2.2; https://cran.r-project.org/web/packages/MatrixEQTL/index.html) [68]. SNPs were defined as markers, and the expression level of transcripts was considered as phenotypes. The significant *P* value threshold was set to 1.0E-04 [65–67]. The eQTLs detected within the transcripts were regarded as cis-eQTL, and others were treated as trans-eQTLs. The transcripts with cis- and trans-eQTLs were subjected to KEGG enrichment analysis. The KEGG pathway enrichment analysis was performed using KOBAS [69]. Significance was set at *FDR* ≤ 0.05.

### SNP validation through the Sanger sequencing and qRT-PCR of gene expression

The 23 key SNP loci (Fig. 6) were selected for validation through the conventional PCR and Sanger sequencing in 213 *C. oleifera* accessions using cDNA of seed kernels as the templates. The PCR and sequencing primers were shown in Additional File 1: Table S23. The PCR amplicons were purified using the QIAquick PCR purification kit (Cat. 28104, QIAgen) and sequenced on an ABI3730 device.

For the gene expression validation, RNA samples of seed kernels from the oil-Camellia accessions were prepared for the qRT-PCR analysis by a One Step PrimeScript III RT-qPCR Kit (RR600B, Takra, Dalian, China). Actin gene was used as the internal reference and the primer pairs were shown in Additional File 1: Table S24. Initially, quantitation results were evaluated and filtered based on the amplification curve and melting curve for each PCR reaction. To calculate the expression, a standard curve of each candidate was obtained; and we performed the normalization of the relative expression using Actin among samples. Three amplification replicates were obtained and the average values were used for the correlation analysis with the expression profiles based on RNA-seq.

## Supplementary Information

**Additional file 1 **Table S1. The genome ploidy level analysis of cultivated oil-camellia and wild species close to the oil-Camellia. Table S2. The hybrid assembly statistics of the sequenced CON genome. Table S3. Summary of repetitive sequence identification. Table S4. Summary of Non-coding RNA gene annotation. Table S5. The assessment of gene models of the CON genome. Table S6. Summary of gene function annotation using various databases. Table S7. Summary of BUSCOs genome assessment results. Table S8. Statistics of data production by ddRAD sequencing for each individual in F1 population. Table S9. A summary of statistics of all SNP markers types in linkage population. Table S10. Features of the 15 linkage groups (LG) in linkage map of *C. oleifera*. Table S11. Origin of the 221 accessions in the association population and summary of their RNAseq data. Table S12. Details of eight important oil traits in mature kernel of *C. oleifera* all accessions for three consecutive years (2013, 2014 and 2015). Table S13. Analysis of variance for eight oil traits in the association population of *C. oleifera*. Table S14. Statistics of SNPs of *C. oleifera* association population in this study. Table S15. Statistics of InDels of *C. oleifera* association population in this study. Table S16. Mean of fruit traits of *C. oleifera* association population. Table S17. The enriched GO terms based on the genes from selective sweep analysis. Table S18. Loci significantly associated with oil traits in GWAS. Table S19. The key candidate genes mined by qGWAS in *C. oleifera* association population. Table S20. Summary of the significantly enriched KEGG pathways of the genes with cis-eQTLs, trans-eQTLs targeted genes and genes covered the trans-eQTLs. Table S21. The summary of Sanger sequencing validation of SNPs identified by the RNA-seq analysis. Table S22. The first ten components in PCA results of association population. Table S23. Description of the Sanger sequencing primers used in our studies. Table S24. Description of the Real-time quantitative PCR primers used in our studies.

**Additional file 2 **Fig. S1. The diploid progenitor *C. oleifera* “Nanyongensis” (CON) and the karyotyping of the CON plant. Fig. S2. The k-mer distribution of sequencing reads. Fig. S3. The strategy of the genome assembly based on multiple sequencing datasets. Fig. S4. Heatmap of Hi-C chromosomal interaction. Fig. S5. The SNP-based genetic map for *C. oleifera* using the ‘Changlin 53’ × ‘Changlin 81’ population. Fig. S6. Pearson correlation matrix for eight oil traits of *C. oleifera* population. Fig. S7. PCA plots of *C. oleifera* accessions. Fig. S8. LD levels among pairwise SNPs in seven subpopulations. Fig. S9. Genomic signatures of domestication detected by selective sweep analysis. Fig. S10. The relationship between co-expression module and ORTs in the oil-Camellia cultivar population. Fig. S11. The scatter plots for the expression profiles of eight key candidate genes by qRT-PCR analysis and RNA-seq results. Fig. S12. Combine QQ plots for eight oil traits of *C. oleifera* accessions.

**Additional file 3.** Method S1. Genome sequencing and assembly. Method S2. Genome annotation and evaluation. Method S3. Double digest restriction site-associated sequencing (ddRAD) and linkage map construction.

**Additional file 4.** Review history.

## Data Availability

All data needed to evaluate the conclusions in the paper are present in the paper and/or the Supplementary Materials. The original sequencing data are deposited in NCBI Bioproject under accession No. PRJNA732216, including genome sequencing (SRR14710457 to SRR14710508), linkage map construction (SRR14777198 to SRR14777378) and transcriptome sequencing of cultivars (SRR14934120 to SRR14934340) [70]. Associated data of this manuscript, including genome assembly, genome annotation, and transcriptomics analyses, as well as the code scripts for data analysis in this study, are publicly accessible under the GNU General Public License v3.0 from GitHub: https://github.com/Hengfu-Yin/CON_genome_data [20] or Zenodo: https://zenodo.org/record/5768785 [21].
