# Supplementary material for: The genome of oil-Camellia and population genomics analysis provide insights into seed oil domestication
Source: Genome Biol. 2022 Jan 10;23:14. doi: 10.1186/s13059-021-02599-2 (PMC8744323; doi:10.1186/s13059-021-02599-2)
Supplement: Supplementary file 1 — Additional file 1 Table S1. The genome ploidy level analysis of cultivated oil-camellia and wild species close to the oil-Camellia. Table S2. The hybrid assembly statistics of the sequenced CON genome. Table S3. Summary of repetitive sequence identification. Table S4. Summary of Non-coding RNA gene annotation. Table S5. The assessment of gene models of the CON genome. Table S6. Summary of gene function annotation using various databases. Table S7. Summary of BUSCOs genome assessment results. Table S8. Statistics of data production by ddRAD sequencing for each individual in F1 population. Table S9. A summary of statistics of all SNP markers types in linkage population. Table S10. Features of the 15 linkage groups (LG) in linkage map of C. oleifera. Table S11. Origin of the 221 accessions in the association population and summary of their RNAseq data. Table S12. Details of eight important oil traits in mature kernel of C. oleifera all accessions for three consecutive years (2013, 2014 and 2015). Table S13. Analysis of variance for eight oil traits in the association population of C. oleifera. Table S14. Statistics of SNPs of C. oleifera association population in this study. Table S15. Statistics of InDels of C. oleifera association population in this study. Table S16. Mean of fruit traits of C. oleifera association population. Table S17. The enriched GO terms based on the genes from selective sweep analysis. Table S18. Loci significantly associated with oil traits in GWAS. Table S19. The key candidate genes mined by qGWAS in C. oleifera association population. Table S20. Summary of the significantly enriched KEGG pathways of the genes with cis-eQTLs, trans-eQTLs targeted genes and genes covered the trans-eQTLs. Table S21. The summary of Sanger sequencing validation of SNPs identified by the RNA-seq analysis. Table S22. The first ten components in PCA results of association population. Table S23. Description of the Sanger sequencing primers used in our studies. Table S [file 13059_2021_2599_MOESM1_ESM.docx]

**Additional File 1: Supplementary Tables S1-24**

| sample | Mean PI-A | PI-A SD | PI-A %CV | Ploidy level |
| --- | --- | --- | --- | --- |
| *Camellia azalea* | 46932 | 3396 | 7.2 | 2 |
| *Camellia japonica* | 46231 | 3242 | 7.1 | 2 |
| *CON* | 45573 | 2416 | 5.3 | 2 |
| *Camellia oleifera cv. 'XG02'* | 94352 | 4544 | 4.8 | 4 |
| *Camellia oleifera cv. 'XG03'* | 94167 | 3972 | 4.2 | 4 |
| *Camellia oleifera cv. 'XG04'* | 89641 | 4454 | 5 | 4 |
| *Camellia oleifera cv. 'XG06'* | 91015 | 4013 | 4.4 | 4 |
| *Camellia oleifera cv. 'XG07'* | 91573 | 3011 | 3.3 | 4 |
| *Camellia oleifera cv. 'XG08'* | 92287 | 3528 | 3.8 | 4 |
| *Camellia oleifera cv. 'XG09'* | 92758 | 3915 | 4.5 | 4 |
| *Camellia oleifera cv. 'ChangLin4'* | 151732 | 4426 | 2.9 | 6 |
| *Camellia oleifera cv. 'ChangLin18'* | 162596 | 5793 | 4.2 | 6 |
| *Camellia oleifera cv. 'ChangLin53'* | 155022 | 4882 | 3.1 | 6 |
| *Camellia oleifera cv. 'ChangLin166'* | 146719 | 4563 | 3.1 | 6 |
| *Camellia oleifera cv. 'ChangLin81'* | 156012 | 5523 | 3.5 | 6 |
| *Camellia yuhsienensis* | 148259 | 5782 | 3.8 | 6 |

Table S1. The gnome ploidy level analysis of cultivated oil-camellia and wild species close to the oil-Camellia

| Sample Name | Scaffold Number | Scaffold Length (bp) | GC (%) | Contig N50 (bp) | Scaffold N50 (bp) | Chromosome Number | Chromosome Length (%) |
| --- | --- | --- | --- | --- | --- | --- | --- |
| CON | 2,143 | 2,891,061,056 | 37.51 | 1,001,691 | 185,364,083 | 15 | 91.33 |

Table S2. The hybrid assembly statistics of the sequenced CON genome.

| **Type** | **Repeat Number** | **Repeat Size(bp)** | **Percent of genome (%)** |
| --- | --- | --- | --- |
| TR | 1,478,269 | 164,949,106 | 5.710 |
| DNA | 360,066 | 140,483,608 | 4.857 |
| LTR | 843,643 | 870,652,540 | 30.115 |
| LINE | 59,603 | 34,002,958 | 1.175 |
| SINE | 2,977 | 560,222 | 0.019 |
| Other | 104,106 | 45,183,952 | 1.563 |
| Unknown | 1,928,247 | 739,850,088 | 25.591 |
| Total | 4,776,911 | 1,995,682,474 | 69.00 |

Table S3. Summary of repetitive sequence identification

TR, tandem repeat; LTR, long terminal repeat; LINE, long interspersed nuclear elements; SINE, short interspersed nuclear elements.

| Types | | Number | Percent (%) |
| --- | --- | --- | --- |
| tRNA |  | 641 | 27.49 |
| rRNA |  | 970 | 41.60 |
| miRNA |  | 97 | 4.16 |
| snRNA | snRNA | 611 | 26.20 |
|  | CD-box | 326 | 13.98 |
|  | HACA-box | 79 | 3.39 |
|  | splicing | 206 | 8.83 |
| Others |  | 13 | 0.55 |
| Total |  | 2,332 |  |

Table S4. Summary of Non-coding RNA gene annotation

tRNA: transfer RNA; HACA- Box RNAs: H/ACA small nucleolar RNAs; CD-box: Box C/D guide RNAs; miRNA: microRNA; snRNA: small nucleolar RNAs.

| Types |  |
| --- | --- |
| Number of genes | 42462 |
| Total genic length | 167,950,744 |
| Mean gene length | 3955 |
| Number of transcripts | 46,612 |
| Transcripts per gene | 1 |
| Total transcript length | 71,321,209 |
| Mean transcript length | 1,530 |
| Number of exons | 224,607 |
| Exons per transcript | 5 |
| Mean exon length | 317 |
| Number of coding exons | 212,826 |
| Number of introns | 177,995 |
| Mean intron length | 776 |
| Total CDS length | 62,006,458 |
| Mean CDS length | 1330 |

Table S5. The assessment of gene models of the CON genome.

|  | Total | NR | Swissprot | KEGG | TrEMBL | GO |
| --- | --- | --- | --- | --- | --- | --- |
| Number | 37,565 | 37,510 | 26,457 | 9,760 | 32,499 | 19,629 |
| Percentage (%) | 88.47 | 88.34 | 62.31 | 22.99 | 76.54 | 46.23 |

Table S6. Summary of gene function annotation using various databases. NR, NCBI non-redundant protein sequences database; KEGG, Kyoto Encyclopedia of Genes and Genomes; Go, Gene Ontology.

|  | Number | Percent (%) |
| --- | --- | --- |
| Complete and single-copy BUSCOs (S) | 1,171 | 81.3 |
| Complete and duplicated BUSCOs (D) | 127 | 8.8 |
| Fragmented BUSCOs (F) | 36 | 2.5 |
| Missing BUSCOs (M) | 106 | 7.4 |
| Complete BUSCOs (S+D) | 1299 | 90.1 |
| Total BUSCOs | 1,440 |  |

Table S7. Summary of BUSCOs genome assessment results

| ID | ReadSum | BaseSum | GC(%) | Q30(%) | Q20(%) |
| --- | --- | --- | --- | --- | --- |
| Female | 15,102,436 | 4,409,968,184 | 41.51 | 88.52 | 94.84 |
| Male | 18,366,493 | 5,418,115,435 | 42.88 | 89.1 | 95.47 |
| 1 | 6,111,007 | 1,790,594,596 | 39.06 | 91.23 | 95.85 |
| 2 | 7,399,040 | 2,186,644,210 | 41.98 | 88.36 | 94.79 |
| 3 | 10,805,812 | 3,197,759,622 | 47.38 | 82.52 | 91.73 |
| 4 | 13,424,794 | 3,920,039,848 | 41.77 | 89.55 | 93.82 |
| 5 | 27,979,536 | 8,170,024,512 | 42.58 | 89.73 | 93.98 |
| 6 | 47,396,414 | 13,839,752,888 | 45.6 | 83.86 | 91.92 |
| 7 | 6,896,149 | 2,027,467,806 | 42.08 | 90.87 | 94.77 |
| 8 | 6,863,773 | 2,017,949,262 | 42.15 | 90.65 | 94.61 |
| 9 | 24,011,009 | 7,011,214,628 | 43.17 | 89.77 | 94.03 |
| 10 | 7,605,910 | 2,223,826,798 | 40.84 | 88.49 | 93.7 |
| 11 | 52,564,193 | 15,453,872,742 | 44.45 | 86.02 | 93.14 |
| 12 | 8,940,587 | 2,619,823,794 | 39.24 | 90.41 | 95.41 |
| 13 | 51,647,128 | 15,184,255,632 | 44.67 | 86.36 | 93.41 |
| 14 | 12,470,585 | 3,674,408,479 | 42.54 | 88.03 | 93.84 |
| 15 | 6,273,437 | 1,844,390,478 | 42.39 | 89.55 | 93.83 |
| 16 | 21,307,793 | 6,264,491,142 | 45.67 | 85.91 | 93.15 |
| 17 | 5,865,096 | 1,718,473,128 | 39.79 | 91.13 | 95.98 |
| 18 | 7,622,196 | 2,234,611,143 | 38.42 | 88.6 | 94.67 |
| 19 | 7,980,091 | 2,346,146,754 | 42.92 | 89.49 | 93.8 |
| 20 | 8,120,512 | 2,379,886,283 | 39.36 | 89.83 | 95.3 |
| 21 | 10,206,331 | 2,993,531,036 | 41.84 | 84.67 | 92.83 |
| 22 | 10,881,480 | 3,187,885,718 | 40.86 | 86.09 | 93.38 |
| 23 | 7,254,200 | 2,139,259,709 | 44 | 84.34 | 92.15 |
| 24 | 7,511,433 | 2,208,361,302 | 41.17 | 89.55 | 93.81 |
| 25 | 20,172,990 | 5,895,125,108 | 41.85 | 84.89 | 92.37 |
| 26 | 9,449,569 | 2,778,173,286 | 40.64 | 93.14 | 96.95 |
| 27 | 5,888,577 | 1,731,132,497 | 40.54 | 85.92 | 93.68 |
| 28 | 7,956,853 | 2,335,383,283 | 41.66 | 86.52 | 93.62 |
| 29 | 10,433,396 | 3,077,114,286 | 40.97 | 87.16 | 94.03 |
| 30 | 7,706,275 | 2,281,057,400 | 43.17 | 83.15 | 91.49 |
| 31 | 10,037,306 | 2,945,969,111 | 40.23 | 87.44 | 94.03 |
| 32 | 8,418,355 | 2,474,996,370 | 42.63 | 90.74 | 94.69 |
| 33 | 6,922,325 | 2,023,778,539 | 41.69 | 86.26 | 92.81 |
| 34 | 16,790,167 | 4,969,889,432 | 42.65 | 89.68 | 93.95 |
| 35 | 6,054,762 | 1,774,045,266 | 42.22 | 84.24 | 92.08 |
| 36 | 9,223,678 | 2,715,979,076 | 44.01 | 84.53 | 92.3 |
| 37 | 8,905,413 | 2,626,465,814 | 41.91 | 84.54 | 92.81 |
| 38 | 7,382,347 | 2,162,594,248 | 40.15 | 88.07 | 94.33 |
| 39 | 8,200,757 | 2,411,022,558 | 43.9 | 81.57 | 90.66 |
| 40 | 12,682,387 | 3,728,621,778 | 45.47 | 79.8 | 89.57 |
| 41 | 10,409,533 | 3,081,221,768 | 44.33 | 89.44 | 93.79 |
| 42 | 6,150,493 | 1,796,853,936 | 38.31 | 90.82 | 95.65 |
| 43 | 13,384,632 | 3,961,851,072 | 41.54 | 88.3 | 94.13 |
| 44 | 13,566,571 | 3,979,757,838 | 43.07 | 86.2 | 93.41 |
| 45 | 10,037,426 | 2,944,926,174 | 44.31 | 85.53 | 92.92 |
| 46 | 17,372,701 | 5,142,319,496 | 42.61 | 89.6 | 93.9 |
| 47 | 7,148,564 | 2,101,923,891 | 43.34 | 87.83 | 94.11 |
| 48 | 21,678,663 | 6,395,205,585 | 42.86 | 89.45 | 93.78 |
| 49 | 5,710,011 | 1,678,594,460 | 41.76 | 85.89 | 93.7 |
| 50 | 22,323,254 | 6,585,359,930 | 43.1 | 89.78 | 94.02 |
| 51 | 7,243,281 | 2,123,165,679 | 42.45 | 84.53 | 92.66 |
| 52 | 13,628,144 | 4,006,674,336 | 42.17 | 90.81 | 94.73 |
| 53 | 6,481,941 | 1,918,654,536 | 42.02 | 90.62 | 94.59 |
| 54 | 6,131,495 | 1,802,503,581 | 43.51 | 86.65 | 93.39 |
| 55 | 11,486,360 | 3,387,739,088 | 41.29 | 84.66 | 93.29 |
| 56 | 17,236,841 | 5,050,394,413 | 42.51 | 89.44 | 93.76 |
| 57 | 15,909,762 | 4,661,560,266 | 44.47 | 89.58 | 93.91 |
| 58 | 15,559,120 | 4,574,381,280 | 45.05 | 80.4 | 89.92 |
| 59 | 13,358,943 | 3,953,628,792 | 42.57 | 93.41 | 97.1 |
| 60 | 6,058,794 | 1,791,912,832 | 43.56 | 84.25 | 92.13 |
| 61 | 6,100,264 | 1,793,386,730 | 43.44 | 87.51 | 93.89 |
| 62 | 19,224,726 | 5,613,619,992 | 43.1 | 89.6 | 93.89 |
| 63 | 7,469,946 | 2,189,407,692 | 40.99 | 87.89 | 94.22 |
| 64 | 7,379,036 | 2,169,159,623 | 45.23 | 86.83 | 93.53 |
| 65 | 7,065,493 | 2,065,612,874 | 41.12 | 88.62 | 94.66 |
| 66 | 17,187,816 | 5,018,842,272 | 43.03 | 89.65 | 93.93 |
| 67 | 16,075,893 | 4,758,464,328 | 44.46 | 79.84 | 89.63 |
| 68 | 20,511,134 | 5,989,251,128 | 42.7 | 89.72 | 93.98 |
| 69 | 8,209,717 | 2,398,299,325 | 40.49 | 89.61 | 95.2 |
| 70 | 6,660,303 | 1,947,358,620 | 40.14 | 89.77 | 95.24 |
| 71 | 11,044,488 | 3,247,003,518 | 41.53 | 85.8 | 93.38 |
| 72 | 12,022,528 | 3,546,645,760 | 43.2 | 89.5 | 93.82 |
| 73 | 6,271,833 | 1,843,878,386 | 42.42 | 91.14 | 95.65 |
| 74 | 20,667,251 | 6,096,839,045 | 42.65 | 89.38 | 93.72 |
| 75 | 5,735,203 | 1,685,720,538 | 42.62 | 88.75 | 94.73 |
| 76 | 12,748,161 | 3,773,455,656 | 42.44 | 90.81 | 94.74 |
| 77 | 5,925,963 | 1,742,233,122 | 42.51 | 78.95 | 89.53 |
| 78 | 6,753,025 | 1,985,518,560 | 39.89 | 90.79 | 95.77 |
| 79 | 7,864,150 | 2,322,477,994 | 43.2 | 89.92 | 95.34 |
| 80 | 15,918,780 | 4,695,948,074 | 41.81 | 84.57 | 93.23 |
| 81 | 9,422,237 | 2,770,137,678 | 47.36 | 93.16 | 97.02 |
| 82 | 17,780,487 | 5,263,024,152 | 44.1 | 80.34 | 89.9 |
| 83 | 5,619,504 | 1,663,373,184 | 41.7 | 90.74 | 94.67 |
| 84 | 4,833,109 | 1,420,206,481 | 40.06 | 90.85 | 95.77 |
| 85 | 10,135,916 | 2,998,835,696 | 40.49 | 86.23 | 93.85 |
| 86 | 11,071,911 | 3,277,285,656 | 42.01 | 90.75 | 94.69 |
| 87 | 7,811,441 | 2,312,186,536 | 42.59 | 80.95 | 90.86 |
| 88 | 7,601,713 | 2,235,092,742 | 41.11 | 91.3 | 96.05 |
| 89 | 7,475,403 | 2,210,487,477 | 43.07 | 80.82 | 90.73 |
| 90 | 11,620,294 | 3,427,986,730 | 42.17 | 89.71 | 93.94 |
| 91 | 4,990,891 | 1,476,800,586 | 42.01 | 93.08 | 96.96 |
| 92 | 7,600,630 | 2,238,523,944 | 42.44 | 88.64 | 94.79 |
| 93 | 5,792,386 | 1,713,262,892 | 43.26 | 86.06 | 93 |
| 94 | 8,934,875 | 2,635,788,125 | 42.02 | 90.62 | 94.6 |
| 95 | 5,819,776 | 1,711,118,407 | 40.75 | 91.3 | 96.07 |
| 96 | 5,705,833 | 1,688,926,568 | 42.45 | 79.09 | 89.63 |
| 97 | 6,083,924 | 1,800,780,180 | 42.7 | 84.13 | 92.91 |
| 98 | 8,570,892 | 2,519,372,874 | 40.48 | 89.24 | 94.97 |
| 99 | 7,332,392 | 2,156,298,770 | 40.41 | 89.39 | 95.08 |
| 100 | 18,980,924 | 5,599,372,580 | 41.47 | 80.04 | 90.31 |
| 101 | 5,491,392 | 1,614,222,115 | 47.14 | 90.71 | 95.59 |
| 102 | 9,824,887 | 2,878,691,891 | 44.74 | 89.49 | 93.85 |
| 103 | 40,914,968 | 12,029,000,592 | 39.8 | 91.87 | 96.35 |
| 104 | 5,283,052 | 1,555,259,630 | 39.49 | 84.76 | 92.87 |
| 105 | 8,352,351 | 2,466,695,263 | 41.85 | 94.08 | 97.42 |
| 106 | 5,489,587 | 1,624,917,752 | 42.09 | 91.39 | 96.13 |
| 107 | 7,621,654 | 2,255,821,670 | 42.08 | 84.53 | 93.19 |
| 108 | 15,566,647 | 4,561,027,571 | 42.1 | 79.14 | 89.87 |
| 109 | 17,313,058 | 5,072,725,994 | 41.98 | 79.92 | 90.22 |
| 110 | 8,121,094 | 2,390,130,199 | 41.29 | 87.5 | 94.02 |
| 111 | 17,173,806 | 5,031,925,158 | 44.56 | 80.09 | 89.75 |
| 112 | 7,275,805 | 2,152,267,856 | 41.17 | 89.21 | 95 |
| 113 | 6,747,550 | 1,977,032,150 | 47.96 | 87.56 | 93.04 |
| 114 | 16,927,016 | 4,959,615,688 | 42.75 | 89.36 | 93.72 |
| 115 | 10,565,538 | 3,095,702,634 | 44.18 | 89.58 | 93.89 |
| 116 | 41,786,642 | 12,285,272,748 | 44.97 | 86.95 | 93.76 |
| 117 | 15,097,640 | 4,453,803,800 | 42.55 | 90.5 | 94.51 |
| 118 | 9,979,685 | 2,944,007,075 | 42.13 | 90.92 | 94.8 |
| 119 | 13,285,274 | 3,879,300,008 | 43.83 | 89.44 | 93.8 |
| 120 | 9,432,907 | 2,763,841,751 | 42.22 | 90.98 | 94.86 |
| 121 | 6,275,535 | 1,856,470,680 | 38.59 | 89.25 | 94.9 |
| 122 | 8,663,306 | 2,546,907,764 | 41.01 | 83.86 | 92.68 |
| 123 | 10,423,713 | 3,066,765,472 | 41.56 | 90.29 | 95.62 |
| 124 | 15,498,205 | 4,525,475,860 | 41.92 | 79.89 | 90.22 |
| 125 | 9,677,210 | 2,854,448,414 | 41.28 | 84.43 | 93.13 |
| 126 | 11,284,683 | 3,306,412,119 | 41.98 | 90.76 | 94.69 |
| 127 | 31,322,394 | 9,146,139,048 | 42.26 | 79.5 | 90 |
| 128 | 6,811,747 | 2,016,100,331 | 39.37 | 89.99 | 95.44 |
| 129 | 6,196,200 | 1,830,262,742 | 43.54 | 93.68 | 97.24 |
| 130 | 11,951,290 | 3,524,415,520 | 40.63 | 85.69 | 93.54 |
| 131 | 23,549,940 | 6,923,682,360 | 42.74 | 80.82 | 91.02 |
| 132 | 10,894,681 | 3,181,246,852 | 47.93 | 88.67 | 93.34 |
| 133 | 21,660,544 | 6,324,878,848 | 43.03 | 89.52 | 93.84 |
| 134 | 40,176,216 | 11,811,807,504 | 45.4 | 87.33 | 94.04 |
| 135 | 10,626,490 | 3,144,618,098 | 42.65 | 89.93 | 95.43 |
| 136 | 9,341,162 | 2,764,525,610 | 42.92 | 92.63 | 96.77 |
| 137 | 9,314,247 | 2,719,760,124 | 42.25 | 89.57 | 93.84 |
| 138 | 13,572,024 | 3,990,175,056 | 46.16 | 86.75 | 93.71 |
| 139 | 7,622,831 | 2,225,866,652 | 46.06 | 89.23 | 93.67 |
| 140 | 5,590,180 | 1,643,512,920 | 40.45 | 85.86 | 93.65 |
| 141 | 8,522,706 | 2,505,675,564 | 41.13 | 85.74 | 93.59 |
| 142 | 8,682,158 | 2,543,872,294 | 42.03 | 90.56 | 94.54 |
| 143 | 7,569,694 | 2,210,350,648 | 39.14 | 90.7 | 95.73 |
| 144 | 20,135,158 | 5,909,516,428 | 40.84 | 94.56 | 97.64 |
| 145 | 21,582,797 | 6,345,342,318 | 44.42 | 89.53 | 93.86 |
| 146 | 7,102,936 | 2,102,469,056 | 39.28 | 91.52 | 96.22 |
| 147 | 11,215,653 | 3,297,401,982 | 42.93 | 89.58 | 93.88 |
| 148 | 7,182,211 | 2,123,964,327 | 46.17 | 93.97 | 97.41 |
| 149 | 13,388,149 | 3,962,892,104 | 46.78 | 79.23 | 89.94 |
| 150 | 6,474,481 | 1,908,453,086 | 44.75 | 88.16 | 93.52 |
| 151 | 5,901,680 | 1,729,192,240 | 42.13 | 90.87 | 94.77 |
| 152 | 6,212,682 | 1,837,911,020 | 40.86 | 90.3 | 95.51 |
| 153 | 10,030,707 | 2,928,966,444 | 42.19 | 90.74 | 94.68 |
| 154 | 25,840,775 | 7,597,187,850 | 42.82 | 89.64 | 93.93 |
| 155 | 6,948,511 | 2,049,695,939 | 40.73 | 88.5 | 93.61 |
| 156 | 5,655,625 | 1,672,908,476 | 42.62 | 85.95 | 93.16 |
| 157 | 16,517,083 | 4,889,056,568 | 45.76 | 89.51 | 93.86 |
| 158 | 13,262,446 | 3,897,322,784 | 41.15 | 86.27 | 93.42 |
| 159 | 9,638,982 | 2,845,697,306 | 40.66 | 89.54 | 95.18 |
| 160 | 21,735,151 | 6,433,604,696 | 39.35 | 85.44 | 93.42 |
| 161 | 25,073,859 | 7,421,862,264 | 42.05 | 81.38 | 91.36 |
| 162 | 6,030,745 | 1,779,069,775 | 39.17 | 90.29 | 95.58 |
| 163 | 12,702,303 | 3,758,312,131 | 42.72 | 93.78 | 97.3 |
| 164 | 6,429,241 | 1,884,929,551 | 39.11 | 89.51 | 95.17 |
| 165 | 8,103,902 | 2,398,383,043 | 40.12 | 89.98 | 95.43 |
| 166 | 23,384,843 | 6,921,913,528 | 43.3 | 80.8 | 90.99 |
| 167 | 8,562,383 | 2,500,215,836 | 44.11 | 79.75 | 89.54 |
| 168 | 6,449,893 | 1,889,818,649 | 39.24 | 90.45 | 95.69 |
| 169 | 13,707,390 | 4,044,429,167 | 44.27 | 84.18 | 92.11 |
| 170 | 5,803,853 | 1,701,990,468 | 40.63 | 88.68 | 93.7 |
| 171 | 28,485,019 | 8,403,080,605 | 42.59 | 80.94 | 91.12 |
| 172 | 9,760,667 | 2,864,580,257 | 43.22 | 84.99 | 92.55 |
| 173 | 6,489,061 | 1,914,272,995 | 46.42 | 87.97 | 93.25 |
| 174 | 22,579,805 | 6,661,042,475 | 45.5 | 89.28 | 93.7 |
| 175 | 6,795,804 | 2,004,762,180 | 43.83 | 88.58 | 93.7 |
| 176 | 8,478,208 | 2,504,838,400 | 40.98 | 88.7 | 94.7 |
| 177 | 11,488,454 | 3,354,628,568 | 42.4 | 90.7 | 94.67 |
| 178 | 11,574,036 | 3,379,618,512 | 42.1 | 90.68 | 94.63 |
| 179 | 30,235,056 | 8,919,341,520 | 42.23 | 81.18 | 91.27 |
| 180 | 6,831,734 | 2,016,628,425 | 39.88 | 88.92 | 94.88 |

Table S8. Statistics of data production by ddRAD sequencing for each individual in F1 population

| Segregation Patterns * | double-pseudo-test-cross model | SNP Number |
| --- | --- | --- |
| aa × bb | Not used | 2,750 |
| ab × cc | Not used | 109 |
| ab× cd | Not used | 3 |
| cc × ab | Not used | 123 |
| lm × ll | heterozygous (female) × homozygous/absent (male) | 100,453 |
| nn × np | homozygous/absent (female) × heterozygous (male) | 95,278 |
| hk × hk | heterozygous (female) × heterozygous (male) | 52,571 |
| ef × eg | heterozygous/absent (female) × heterozygous/absent (male) | 2,413 |
| Total |  | 253,700 |

Table S9. A summary of statistics of all SNP markers types in linkage population.

First column means predicted genotype segregation patterns which are labeled by “female parent genotype×male parent genotype”. Only the SNPs with the segregation patterns of lm×ll, nn×np, hk×hk and ef×eg were subjected to genetic linage construction using the double pseudo-testcross strategy. The details of SNPs genotype of individuals showed in Data S1 [20, 21].

| LGs | No. of loci | Length (cM) | Density (cM/locus) | Largest gap (cM) | No. of gaps >10 cM |
| --- | --- | --- | --- | --- | --- |
| LG01 | 19 | 113.27 | 5.96 | 15.411 | 2 |
| LG02 | 25 | 148.41 | 5.94 | 21.37 | 3 |
| LG03 | 19 | 194.15 | 10.22 | 27.59 | 8 |
| LG04 | 12 | 90.02 | 7.50 | 27.6 | 2 |
| LG05 | 14 | 92.41 | 6.60 | 14.28 | 3 |
| LG06 | 31 | 170.86 | 5.51 | 30.157 | 3 |
| LG07 | 23 | 200.09 | 8.70 | 25.051 | 7 |
| LG08 | 25 | 151.75 | 6.07 | 21.237 | 6 |
| LG09 | 20 | 88.76 | 4.44 | 9.281 | 0 |
| LG10 | 24 | 149.96 | 6.25 | 20.087 | 3 |
| LG11 | 14 | 86.00 | 6.14 | 19.385 | 3 |
| LG12 | 35 | 170.99 | 4.89 | 23.931 | 4 |
| LG13 | 13 | 138.57 | 10.66 | 40.897 | 3 |
| LG14 | 14 | 57.00 | 4.07 | 15.533 | 1 |
| LG15 | 12 | 85.00 | 7.08 | 28.741 | 3 |
| Total | 300 | 1937.22 | 6.46 | / | 51 |
| Mean | 20 | 129.15 | 6.46 | / |  |

Table S10. Features of the 15 linkage groups (LG) in linkage map of *C. oleifera*

| Accession | Origin | Group^*^ | Number of Clean reads | Total bases (Gb) | Read number mapped into reference genome |
| --- | --- | --- | --- | --- | --- |
| L1 | Maogang, Jiangxi, China | VII | 61768716 | 9.33 | 83.46% |
| L2 | Maogang, Jiangxi, China | V | 63574062 | 9.61 | 82.62% |
| L3 | Maogang, Jiangxi, China | I | 55879444 | 8.45 | 79.01% |
| L4 | Maogang, Jiangxi, China | IV | 50616026 | 7.66 | 80.96% |
| L8 | Maogang, Jiangxi, China | VII | 52226374 | 7.89 | 84.25% |
| L9 | Maogang, Jiangxi, China | II | 41922946 | 6.39 | 79.32% |
| L10 | Maogang, Jiangxi, China | I | 52625154 | 7.99 | 81.67% |
| L11 | Maogang, Jiangxi, China | II | 53248004 | 8.07 | 81.86% |
| L12 | Maogang, Jiangxi, China | II | 54489538 | 8.24 | 84.28% |
| L15 | Maogang, Jiangxi, China | V | 53940588 | 8.16 | 83.22% |
| L16 | Maogang, Jiangxi, China | III | 49948850 | 7.56 | 82.96% |
| L17 | Anji, Zhejiang, China | VI | 54289304 | 8.21 | 82.95% |
| L18 | Anji, Zhejiang, China | III | 48661810 | 7.36 | 81.20% |
| L19 | Yongxing, Hunan, China | V | 58702660 | 8.89 | 82.04% |
| L20 | Anji, Zhejiang, China | II | 54515142 | 8.23 | 82.90% |
| L21 | Anji, Zhejiang, China | IV | 51924690 | 7.84 | 83.26% |
| L22 | Anji, Zhejiang, China | I | 63196340 | 9.56 | 82.59% |
| L23 | Anji, Zhejiang, China | II | 63183784 | 9.55 | 82.25% |
| L24 | Anji, Zhejiang, China | I | 53804310 | 8.14 | 81.19% |
| L26 | Anji, Zhejiang, China | IV | 50981046 | 7.70 | 83.37% |
| L27 | Anji, Zhejiang, China | V | 56550612 | 8.56 | 81.42% |
| L28 | Yongxing, Hunan, China | V | 57829810 | 8.73 | 82.94% |
| L29 | Yongxing, Hunan, China | V | 50980492 | 7.71 | 83.02% |
| L30 | Yongxing, Hunan, China | I | 51252876 | 7.78 | 81.67% |
| L31 | Yongxing, Hunan, China | I | 58695546 | 8.87 | 83.51% |
| L32 | Yongxing, Hunan, China | I | 61437554 | 9.28 | 83.40% |
| L39 | Unknown, China | VI | 61485066 | 9.33 | 82.53% |
| L40 | Anji, Zhejiang, China | III | 48407452 | 7.31 | 82.74% |
| L42 | Hongguang, Hunan, China | I | 58132684 | 8.79 | 82.85% |
| L43 | Hongguang, Hunan, China | IV | 50611532 | 7.68 | 81.91% |
| L44 | Hongguang, Hunan, China | V | 54240492 | 8.20 | 81.74% |
| L45 | Hongguang, Hunan, China | I | 41328512 | 6.29 | 79.50% |
| L46 | Unknown, China | IV | 43402096 | 6.62 | 82.00% |
| L49 | Yongxing, Hunan, China | II | 56430826 | 8.53 | 83.07% |
| L52 | Anji, Zhejiang, China | IV | 49320630 | 7.46 | 83.48% |
| L53 | Anji, Zhejiang, China | IV | 52352552 | 7.92 | 83.10% |
| L54 | Anji, Zhejiang, China | II | 58086910 | 8.78 | 81.74% |
| L55 | Anji, Zhejiang, China | I | 50308154 | 7.59 | 83.31% |
| L56 | Anji, Zhejiang, China | IV | 51684484 | 7.82 | 81.50% |
| L58 | Anji, Zhejiang, China | III | 53485676 | 8.09 | 82.74% |
| L59 | Anji, Zhejiang, China | I | 46662462 | 7.04 | 82.40% |
| L60 | Anji, Zhejiang, China | I | 53841236 | 8.15 | 83.02% |
| L61 | Maogang, Jiangxi, China | VII | 54397688 | 8.22 | 82.01% |
| L63 | Anji, Zhejiang, China | VI | 52681062 | 7.96 | 82.49% |
| L64 | Juchong, Hunan, China | IV | 54667962 | 8.26 | 82.33% |
| L65 | Juchong, Hunan, China | IV | 53545676 | 8.09 | 82.63% |
| L66 | Juchong, Hunan, China | IV | 58726674 | 8.89 | 81.69% |
| L67 | Juchong, Hunan, China | VI | 74529288 | 11.27 | 78.89% |
| L69 | Unknown, China | I | 56181620 | 8.52 | 82.01% |
| L70 | Linqing, Jiangxi, China | II | 57719906 | 8.73 | 82.89% |
| L71 | Linqing, Jiangxi, China | VI | 56922242 | 8.61 | 82.60% |
| L72 | Unknown, China | IV | 58393306 | 8.82 | 82.02% |
| L73 | Linqing, Jiangxi, China | I | 42947708 | 6.54 | 79.05% |
| L76 | Lintong, Jiangxi, China | I | 58412822 | 8.83 | 76.22% |
| L77 | Lintong, Jiangxi, China | IV | 54640374 | 8.26 | 81.98% |
| L78 | Daqing, Zhejiang, China | I | 47802608 | 7.22 | 81.69% |
| L81 | Maogang, Jiangxi, China | II | 51624344 | 7.82 | 81.88% |
| L82 | Hongguang, Hunan, China | II | 55040466 | 8.33 | 81.65% |
| L86 | Daqing, Zhejiang, China | IV | 55424066 | 8.38 | 81.90% |
| L87 | Juchong, Hunan, China | I | 54367806 | 8.24 | 82.50% |
| L88 | Juchong, Hunan, China | I | 50768930 | 7.67 | 82.68% |
| L89 | Unknown, China | IV | 54246114 | 8.19 | 83.17% |
| L90 | Juchong, Hunan, China | III | 54253922 | 8.21 | 82.99% |
| L91 | Juchong, Hunan, China | I | 50293880 | 7.61 | 79.87% |
| L94 | Yichun, Jiangxi, China | III | 36016178 | 5.47 | 80.80% |
| L95 | Yichun, Jiangxi, China | IV | 54607148 | 8.25 | 82.99% |
| L96 | Yichun, Jiangxi, China | III | 48142630 | 7.29 | 82.94% |
| L97 | Yichun, Jiangxi, China | IV | 52336576 | 7.92 | 79.62% |
| L98 | Yichun, Jiangxi, China | I | 55645646 | 8.41 | 82.63% |
| L99 | Linqing, Jiangxi, China | IV | 54192156 | 8.20 | 81.94% |
| L100 | Linqing, Jiangxi, China | VII | 51981870 | 7.86 | 82.68% |
| L101 | Linqing, Jiangxi, China | III | 53526366 | 8.09 | 81.26% |
| L102 | Unknown, China | VII | 50735478 | 7.71 | 80.97% |
| L106 | Lintong, Jiangxi, China | I | 47917324 | 7.24 | 82.20% |
| L119 | Shaoguan, Guangdong, China | V | 80871908 | 12.24 | 78.64% |
| L145 | Yichun, Jiangxi, China | II | 55111938 | 8.36 | 82.38% |
| L150 | Unknown, China | IV | 52886764 | 7.99 | 81.51% |
| L151 | Yongfeng, Jiangxi, China | I | 54902542 | 8.30 | 82.95% |
| L152 | Yongfeng, Jiangxi, China | IV | 58350780 | 8.83 | 83.06% |
| L153 | Yongfeng, Jiangxi, China | III | 48886530 | 7.39 | 82.02% |
| L155 | Yongfeng, Jiangxi, China | IV | 53297658 | 8.05 | 82.57% |
| L160 | Maogang, Jiangxi, China | III | 55986150 | 8.46 | 82.23% |
| L161 | Maogang, Jiangxi, China | III | 46046936 | 6.95 | 82.39% |
| L162 | Maogang, Jiangxi, China | IV | 53110936 | 8.03 | 82.58% |
| L163 | Maogang, Jiangxi, China | II | 52897318 | 8.00 | 81.76% |
| L164 | Maogang, Jiangxi, China | IV | 50319934 | 7.60 | 82.87% |
| L165 | Maogang, Jiangxi, China | II | 47733716 | 7.22 | 82.41% |
| L166 | Maogang, Jiangxi, China | V | 59318394 | 8.96 | 83.40% |
| L167 | Maogang, Jiangxi, China | II | 55655016 | 8.41 | 82.92% |
| L168 | Maogang, Jiangxi, China | IV | 61749674 | 9.34 | 81.84% |
| L170 | Maogang, Jiangxi, China | V | 58719206 | 8.90 | 81.64% |
| L172 | Maogang, Jiangxi, China | I | 34361208 | 5.21 | 82.48% |
| L173 | Maogang, Jiangxi, China | IV | 40870674 | 6.24 | 80.22% |
| L174 | Maogang, Jiangxi, China | III | 59214498 | 8.93 | 82.60% |
| L175 | Maogang, Jiangxi, China | I | 64156060 | 9.71 | 79.28% |
| L176 | Maogang, Jiangxi, China | I | 59159426 | 8.94 | 83.33% |
| L177 | Maogang, Jiangxi, China | II | 51845442 | 7.83 | 83.13% |
| L178 | Maogang, Jiangxi, China | III | 64721904 | 9.77 | 82.24% |
| L180 | Maogang, Jiangxi, China | V | 46254546 | 6.99 | 82.53% |
| L182 | Maogang, Jiangxi, China | II | 55027716 | 8.33 | 81.61% |
| L185 | Maogang, Jiangxi, China | I | 56024984 | 8.48 | 79.53% |
| L186 | Unknown, China | V | 57900528 | 8.76 | 79.54% |
| L212 | Dongyang, Zhejiang, China | IV | 51693818 | 7.83 | 81.53% |
| L213 | Dongyang, Zhejiang, China | I | 46011792 | 6.95 | 83.27% |
| L214 | Dongyang, Zhejiang, China | III | 58751498 | 8.88 | 81.92% |
| L217 | Maogang, Jiangxi, China | VII | 54529248 | 8.24 | 81.23% |
| L219 | Maogang, Jiangxi, China | IV | 51843632 | 7.84 | 83.80% |
| L220 | Maogang, Jiangxi, China | IV | 61129640 | 9.26 | 81.47% |
| L222 | Maogang, Jiangxi, China | IV | 48110448 | 7.30 | 80.99% |
| L226 | Maogang, Jiangxi, China | III | 38303788 | 5.84 | 80.04% |
| L227 | Maogang, Jiangxi, China | II | 54136778 | 8.19 | 82.15% |
| L228 | Maogang, Jiangxi, China | II | 51997032 | 7.87 | 82.55% |
| L229 | Maogang, Jiangxi, China | II | 53522072 | 8.10 | 83.09% |
| L232 | Maogang, Jiangxi, China | II | 49744660 | 7.51 | 82.58% |
| L233 | Maogang, Jiangxi, China | I | 59038682 | 8.92 | 82.53% |
| L252 | Maogang, Jiangxi, China | I | 56703454 | 8.59 | 82.35% |
| L297 | Unknown, China | IV | 42020870 | 6.41 | 80.42% |
| L298 | Unknown, China | I | 49189818 | 7.43 | 82.06% |
| L299 | Unknown, China | II | 61186382 | 9.26 | 82.66% |
| L501 | Fuyang, Zhejiang, China | II | 57293440 | 8.65 | 82.36% |
| L504 | Fuyang, Zhejiang, China | I | 60671590 | 9.19 | 83.30% |
| L530 | Fuyang, Zhejiang, China | III | 47997944 | 7.25 | 82.36% |
| L531 | Fuyang, Zhejiang, China | III | 59763856 | 9.05 | 80.79% |
| L534 | Fuyang, Zhejiang, China | III | 46585314 | 7.04 | 83.03% |
| L536 | Fuyang, Zhejiang, China | III | 59984504 | 9.06 | 82.37% |
| L539 | Fuyang, Zhejiang, China | III | 56378542 | 8.54 | 81.94% |
| L540 | Fuyang, Zhejiang, China | II | 66081292 | 10.00 | 81.06% |
| L543 | Fuyang, Zhejiang, China | III | 58149444 | 8.78 | 82.29% |
| L545 | Fuyang, Zhejiang, China | I | 59838506 | 9.06 | 83.22% |
| L547 | Fuyang, Zhejiang, China | III | 37152390 | 5.66 | 78.84% |
| L548 | Fuyang, Zhejiang, China | III | 65692016 | 9.92 | 82.56% |
| L559 | Fuyang, Zhejiang, China | III | 63095382 | 9.52 | 83.52% |
| L561 | Fuyang, Zhejiang, China | I | 49980092 | 7.56 | 81.45% |
| L564 | Fuyang, Zhejiang, China | III | 43794998 | 6.62 | 82.30% |
| L3001 | Fuyang, Zhejiang, China | I | 61000120 | 9.22 | 82.40% |
| L3003 | Fuyang, Zhejiang, China | III | 55660688 | 8.41 | 83.06% |
| L3007 | Fuyang, Zhejiang, China | III | 41834962 | 6.32 | 82.92% |
| L3009 | Fuyang, Zhejiang, China | III | 99110242 | 15.00 | 82.64% |
| L3011 | Fuyang, Zhejiang, China | I | 44274294 | 6.69 | 80.98% |
| L3012 | Anji, Zhejiang, China | III | 41601458 | 6.34 | 80.03% |
| L3014 | Fuyang, Zhejiang, China | II | 40147948 | 6.07 | 83.89% |
| L3038 | Fuyang, Zhejiang, China | II | 65029526 | 9.82 | 81.59% |
| L164-1 | Maogang, Jiangxi, China | I | 60771416 | 9.19 | 80.44% |
| L213-1 | Dongyang, Zhejiang, China | I | 48872672 | 7.39 | 83.67% |
| Lfy1 | Fuyang, Zhejiang, China | I | 64977046 | 9.82 | 82.16% |
| Lfy2 | Fuyang, Zhejiang, China | II | 54483902 | 8.24 | 80.93% |
| Lfy5 | Fuyang, Zhejiang, China | I | 46005454 | 6.95 | 80.54% |
| Lfy7 | Fuyang, Zhejiang, China | III | 53225178 | 8.03 | 82.99% |
| Lfy9 | Fuyang, Zhejiang, China | III | 43440670 | 6.56 | 82.46% |
| LbendiCK | Fenyi, Jiangxi, China | III | 55645326 | 8.44 | 81.83% |
| Lde1 | Changsha, Hunan, China | II | 64794024 | 9.82 | 81.83% |
| Lgan1 | Nanchang, Jiangxi, China | VI | 61009646 | 9.22 | 82.74% |
| Lgan12 | Nanchang, Jiangxi, China | IV | 59799204 | 9.05 | 82.45% |
| Lgan2 | Nanchang, Jiangxi, China | V | 58385882 | 8.83 | 82.90% |
| Lgan3 | Nanchang, Jiangxi, China | I | 57936834 | 8.75 | 82.54% |
| Lgan4 | Nanchang, Jiangxi, China | I | 62482914 | 9.44 | 82.73% |
| Lgan5 | Nanchang, Jiangxi, China | V | 59116042 | 8.94 | 81.97% |
| Lgan9 | Nanchang, Jiangxi, China | V | 48361672 | 7.30 | 83.06% |
| Lganwu2 | Nanning, Guangxi, China | I | 55307376 | 8.37 | 81.86% |
| Lgui1 | Nanning, Guangxi, China | V | 49064460 | 7.40 | 82.23% |
| Lgui2 | Nanning, Guangxi, China | IV | 54646614 | 8.28 | 81.88% |
| Lgui3 | Nanning, Guangxi, China | V | 49904352 | 7.54 | 82.43% |
| Lgui4 | Nanning, Guangxi, China | VI | 58332458 | 8.81 | 83.42% |
| Lguiruan2 | Cenxi, Guangxi, China | VII | 58446890 | 8.83 | 82.97% |
| Lguiruan23 | Cenxi, Guangxi, China | VII | 56046070 | 8.48 | 81.74% |
| Lguiruan3 | Cenxi, Guangxi, China | VII | 55954308 | 8.46 | 82.91% |
| Ljiangxi23 | Fuyang, Zhejiang, China | III | 57416658 | 8.67 | 82.62% |
| Lmin43 | Fuan, Fujian, China | V | 53657242 | 8.11 | 82.69% |
| Lmin48 | Fuan, Fujian, China | III | 63058788 | 9.53 | 82.00% |
| Lmin60 | Fuan, Fujian, China | V | 54218794 | 8.19 | 83.20% |
| Lminyou1 | Fuan, Fujian, China | V | 56931406 | 8.62 | 82.07% |
| Lminyou10 | Fuan, Fujian, China | V | 53897306 | 8.15 | 82.95% |
| Lminyou11 | Fuan, Fujian, China | V | 61141356 | 9.27 | 81.87% |
| Lminyou2 | Fuan, Fujian, China | V | 58080814 | 8.78 | 81.38% |
| Lminyou4 | Fuan, Fujian, China | V | 52955060 | 8.02 | 80.91% |
| Lminyou43 | Fuan, Fujian, China | V | 68471778 | 10.34 | 83.54% |
| Lminyou48 | Fuan, Fujian, China | V | 60038496 | 9.08 | 81.78% |
| Lminyou5 | Fuan, Fujian, China | V | 55461362 | 8.38 | 82.67% |
| Lminyou6 | Fuan, Fujian, China | VI | 66871828 | 10.11 | 83.10% |
| Lminyou60 | Fuan, Fujian, China | VII | 59538914 | 8.99 | 81.64% |
| Lminyou8 | Fuan, Fujian, China | II | 51234886 | 7.74 | 83.06% |
| Lminyou9 | Fuan, Fujian, China | II | 66654924 | 10.07 | 81.97% |
| Ltong10 | Jinxiu, Guangxi, China | II | 52683138 | 7.96 | 79.81% |
| Ltong11 | Jinxiu, Guangxi, China | IV | 62590480 | 9.49 | 83.49% |
| Ltong12 | Jinxiu, Guangxi, China | I | 61879292 | 9.36 | 82.11% |
| Ltong18 | Jinxiu, Guangxi, China | I | 50833180 | 7.68 | 83.39% |
| Ltong29 | Jinxiu, Guangxi, China | VI | 57418712 | 8.68 | 82.73% |
| Ltong3 | Jinxiu, Guangxi, China | VI | 51748254 | 7.83 | 81.57% |
| Ltong30 | Jinxiu, Guangxi, China | VI | 61851944 | 9.34 | 82.04% |
| Ltong32 | Jinxiu, Guangxi, China | VI | 49544124 | 7.48 | 82.07% |
| Ltong38 | Jinxiu, Guangxi, China | II | 65883816 | 9.97 | 82.24% |
| Ltong39 | Jinxiu, Guangxi, China | V | 59097986 | 8.92 | 82.58% |
| Ltong4 | Jinxiu, Guangxi, China | V | 59283042 | 8.97 | 81.19% |
| Ltong40 | Jinxiu, Guangxi, China | VI | 63858188 | 9.65 | 81.73% |
| Ltong41 | Jinxiu, Guangxi, China | I | 63545242 | 9.61 | 81.80% |
| Ltong6 | Jinxiu, Guangxi, China | III | 43597512 | 6.59 | 82.52% |
| Ltong9 | Jinxiu, Guangxi, China | I | 60367984 | 9.12 | 82.16% |
| Lwu | Unknown, China | VI | 52970846 | 8.00 | 82.10% |
| Lwu-1 | Unknown, China | III | 55604522 | 8.45 | 81.80% |
| Lwu5-1 | Fuyang, Zhejiang, China | V | 57173764 | 8.65 | 82.82% |
| Lwu5-2 | Fuyang, Zhejiang, China | VII | 45366550 | 6.85 | 83.55% |
| Lwupai5 | Fuyang, Zhejiang, China | V | 59261670 | 8.96 | 82.23% |
| Lwuyiwu2 | Jinhua, Zhejiang, China | VI | 55945760 | 8.45 | 83.62% |
| Lxiang3 | Changsha, Hunan, China | V | 62320442 | 9.43 | 82.37% |
| Lxiang4 | Changsha, Hunan, China | V | 51747788 | 7.84 | 81.60% |
| Lxiang47 | Changsha, Hunan, China | V | 51587254 | 7.80 | 82.61% |
| Lxiang5 | Changsha, Hunan, China | III | 57731276 | 8.72 | 82.27% |
| Lxiang50 | Changsha, Hunan, China | V | 57169852 | 8.64 | 82.52% |
| Lxiang6 | Changsha, Hunan, China | IV | 62475388 | 9.45 | 81.75% |
| Lxiang7 | Changsha, Hunan, China | IV | 56869226 | 8.60 | 81.84% |
| Lxiang8 | Changsha, Hunan, China | V | 53833066 | 8.13 | 83.74% |
| Lxiang9 | Changsha, Hunan, China | VII | 56056662 | 8.47 | 82.68% |
| Lyajia1 | Fuyang, Zhejiang, China | VII | 46989282 | 7.11 | 83.04% |
| Lyajia2 | Fuyang, Zhejiang, China | V | 49358606 | 7.45 | 82.60% |
| Lyajia6 | Fuyang, Zhejiang, China | III | 54278780 | 8.19 | 82.56% |
| Lyajia8 | Fuyang, Zhejiang, China | V | 50736160 | 7.69 | 82.14% |
| Lyawu1 | Fuyang, Zhejiang, China | V | 62640970 | 9.46 | 82.65% |
| Lyawu2 | Fuyang, Zhejiang, China | V | 57582696 | 8.70 | 83.09% |
| Lyawu4 | Fuyang, Zhejiang, China | V | 61886266 | 9.36 | 82.64% |
| Lyawu6 | Fuyang, Zhejiang, China | III | 58510814 | 8.85 | 81.89% |
| Lyawu7 | Fuyang, Zhejiang, China | V | 58767350 | 8.87 | 83.41% |

Table S11. Origin of the 221 accessions in the association population and summary of their RNAseq data

Group*, the 221 accessions were clustered into seven distinct groups I, II, III, IV, V, VI and VII, based on the maximum likelihood phylogeny analysis.

Table S12-1

| Line | OC % | | |  | palmitic acid | | |  | palmitoleic acid | | |  | stearic acid | | |
| --- | --- | --- | --- | --- | --- | --- | --- | --- | --- | --- | --- | --- | --- | --- | --- |
|  | 2013 | 2014 | 2015 |  | 2013 | 2014 | 2015 |  | 2013 | 2014 | 2015 |  | 2013 | 2014 | 2015 |
| L1 | 43.11 | 37.80 | 48.74 |  | 8.48 | 9.33 | 9.00 |  | 0.05 | 0.03 | 0.03 |  | 2.35 | 1.60 | 2.30 |
| L2 | 38.74 | 35.12 | 44.91 |  | 7.70 | 8.45 | 7.87 |  | 0.00 | 0.05 | 0.00 |  | 2.20 | 1.60 | 1.63 |
| L3 | 42.70 | 29.56 | 46.39 |  | 8.04 | 9.89 | 8.08 |  | 0.03 | 0.06 | 0.03 |  | 2.73 | 1.37 | 2.23 |
| L4 | 40.97 | 38.91 | 46.94 |  | 8.05 | 8.98 | 8.07 |  | 0.02 | 0.03 | 0.03 |  | 2.30 | 1.62 | 1.80 |
| L8 | 42.20 | 36.82 | 44.35 |  | 9.21 | 9.75 | 9.13 |  | 0.03 | 0.10 | 0.00 |  | 1.98 | 1.58 | 1.97 |
| L9 | 42.56 | 42.90 | 48.71 |  | 8.10 | 8.60 | 8.57 |  | 0.00 | 0.03 | 0.07 |  | 1.90 | 1.60 | 1.83 |
| L10 | 35.30 | 40.30 | 46.89 |  | 7.67 | 7.77 | 7.20 |  | 0.00 | 0.07 | 0.00 |  | 2.29 | 2.67 | 2.83 |
| L11 | 32.93 | 44.94 | 48.25 |  | 7.45 | 6.97 | 7.83 |  | 0.08 | 0.07 | 0.03 |  | 2.05 | 2.37 | 1.97 |
| L12 | 37.56 | 41.46 | 39.18 |  | 8.55 | 7.37 | 7.90 |  | 0.05 | 0.03 | 0.00 |  | 2.10 | 2.47 | 1.95 |
| L15 | 34.61 | 33.78 | 41.69 |  | 7.77 | 7.90 | 7.60 |  | 0.00 | 0.07 | 0.00 |  | 2.47 | 1.97 | 2.03 |
| L16 | 44.51 | 44.13 | 50.00 |  | 7.57 | 7.40 | 7.97 |  | 0.00 | 0.05 | 0.07 |  | 1.83 | 2.40 | 2.33 |
| L17 | 35.24 | 36.69 | 37.19 |  | 8.91 | 7.90 | 8.50 |  | 0.07 | 0.10 | 0.05 |  | 2.34 | 2.20 | 2.20 |
| L18 | 39.64 | 42.39 | 48.59 |  | 8.09 | 8.77 | 7.85 |  | 0.04 | 0.09 | 0.00 |  | 2.50 | 1.89 | 2.20 |
| L19 | 42.18 | 39.52 | 46.80 |  | 8.70 | 8.47 | 9.70 |  | 0.00 | 0.10 | 0.05 |  | 2.20 | 1.70 | 1.70 |
| L20 | 43.17 | 29.05 | 37.93 |  | 8.10 | 8.45 | 8.00 |  | 0.03 | 0.10 | 0.05 |  | 1.83 | 1.70 | 1.95 |
| L21 | 40.22 | 48.28 | 47.61 |  | 8.03 | 7.93 | 8.60 |  | 0.00 | 0.08 | 0.10 |  | 2.47 | 2.55 | 2.40 |
| L22 | 34.60 | 38.87 | 50.02 |  | 7.90 | 9.93 | 8.25 |  | 0.03 | 0.10 | 0.05 |  | 2.57 | 1.57 | 1.65 |
| L23 | 37.03 | 42.74 | 50.62 |  | 8.30 | 8.23 | 7.75 |  | 0.00 | 0.07 | 0.00 |  | 2.27 | 2.40 | 2.65 |
| L24 | 35.50 | 32.16 | 42.92 |  | 7.80 | 8.73 | 7.45 |  | 0.00 | 0.07 | 0.00 |  | 2.17 | 1.97 | 2.20 |
| L26 | 31.93 | 42.13 | 43.63 |  | 8.47 | 7.73 | 7.55 |  | 0.03 | 0.07 | 0.03 |  | 2.10 | 2.07 | 2.15 |
| L27 | 36.77 | 43.04 | 37.89 |  | 8.70 | 7.80 | 8.95 |  | 0.00 | 0.06 | 0.05 |  | 2.40 | 2.02 | 2.05 |
| L28 | 33.43 | 32.55 | 42.32 |  | 7.60 | 8.33 | 7.40 |  | 0.00 | 0.10 | 0.05 |  | 2.60 | 1.47 | 2.10 |
| L29 | 36.41 | 36.58 | 42.42 |  | 6.80 | 6.77 | 6.55 |  | 0.00 | 0.07 | 0.00 |  | 2.30 | 2.37 | 2.50 |
| L30 | 36.84 | 39.76 | 45.09 |  | 8.44 | 8.98 | 7.80 |  | 0.01 | 0.05 | 0.00 |  | 2.40 | 2.05 | 2.35 |
| L31 | 28.51 | 33.90 | 45.28 |  | 8.64 | 7.85 | 7.90 |  | 0.10 | 0.10 | 0.05 |  | 1.69 | 1.75 | 2.15 |
| L32 | 41.56 | 42.99 | 46.51 |  | 7.80 | 9.00 | 7.65 |  | 0.10 | 0.00 | 0.00 |  | 2.40 | 2.20 | 1.95 |
| L39 | 23.51 | 35.66 | 46.81 |  | 9.47 | 8.80 | 7.45 |  | 0.11 | 0.07 | 0.05 |  | 1.14 | 1.33 | 2.15 |
| L40 | 39.06 | 36.46 | 48.13 |  | 7.77 | 8.83 | 8.40 |  | 0.00 | 0.08 | 0.10 |  | 2.60 | 1.85 | 2.30 |
| L42 | 36.17 | 39.11 | 42.73 |  | 8.37 | 7.72 | 8.25 |  | 0.07 | 0.08 | 0.02 |  | 1.93 | 1.93 | 1.62 |
| L43 | 33.31 | 39.99 | 44.42 |  | 8.27 | 7.85 | 8.70 |  | 0.10 | 0.08 | 0.00 |  | 1.80 | 2.08 | 2.05 |
| L44 | 35.92 | 33.26 | 42.85 |  | 8.28 | 8.03 | 8.35 |  | 0.00 | 0.00 | 0.05 |  | 2.35 | 2.00 | 2.00 |
| L45 | 33.01 | 38.60 | 41.47 |  | 7.64 | 7.05 | 6.80 |  | 0.01 | 0.10 | 0.00 |  | 2.52 | 2.65 | 2.80 |
| L46 | 35.38 | 47.38 | 51.31 |  | 7.37 | 7.65 | 6.65 |  | 0.00 | 0.08 | 0.00 |  | 1.63 | 1.95 | 1.75 |
| L49 | 27.50 | 33.30 | 40.90 |  | 8.44 | 7.55 | 7.40 |  | 0.09 | 0.10 | 0.05 |  | 1.52 | 2.05 | 2.15 |
| L52 | 32.37 | 44.18 | 43.88 |  | 8.54 | 8.30 | 8.60 |  | 0.03 | 0.10 | 0.05 |  | 1.64 | 1.80 | 1.90 |
| L53 | 38.97 | 29.45 | 41.66 |  | 7.85 | 8.38 | 7.98 |  | 0.04 | 0.04 | 0.03 |  | 2.23 | 1.66 | 1.85 |
| L54 | 37.21 | 49.99 | 54.43 |  | 7.93 | 7.85 | 7.75 |  | 0.07 | 0.05 | 0.00 |  | 2.07 | 2.00 | 1.95 |
| L55 | 30.58 | 46.18 | 51.93 |  | 8.20 | 7.40 | 8.35 |  | 0.00 | 0.10 | 0.05 |  | 1.75 | 2.40 | 2.20 |
| L56 | 36.77 | 38.58 | 42.80 |  | 9.47 | 9.00 | 9.15 |  | 0.03 | 0.10 | 0.00 |  | 1.50 | 1.70 | 1.70 |
| L58 | 36.95 | 42.12 | 47.84 |  | 8.17 | 8.60 | 8.70 |  | 0.01 | 0.05 | 0.05 |  | 2.32 | 1.85 | 1.90 |
| L59 | 35.52 | 42.08 | 49.89 |  | 8.17 | 7.87 | 8.30 |  | 0.08 | 0.07 | 0.05 |  | 2.38 | 2.60 | 2.30 |
| L60 | 38.02 | 37.75 | 45.77 |  | 9.36 | 8.45 | 8.95 |  | 0.09 | 0.10 | 0.05 |  | 1.80 | 2.45 | 2.25 |
| L61 | 39.05 | 43.59 | 50.27 |  | 8.26 | 8.77 | 8.95 |  | 0.00 | 0.10 | 0.00 |  | 2.83 | 2.40 | 2.40 |
| L63 | 40.53 | 38.74 | 50.30 |  | 8.10 | 8.87 | 8.55 |  | 0.02 | 0.07 | 0.05 |  | 2.20 | 1.43 | 2.00 |
| L64 | 40.76 | 42.98 | 45.77 |  | 7.60 | 8.43 | 7.70 |  | 0.00 | 0.03 | 0.00 |  | 2.10 | 1.83 | 1.80 |
| L65 | 43.54 | 40.51 | 44.55 |  | 8.99 | 8.07 | 7.40 |  | 0.00 | 0.07 | 0.00 |  | 2.31 | 2.23 | 2.75 |
| L66 | 38.64 | 28.50 | 43.27 |  | 8.45 | 8.25 | 7.85 |  | 0.00 | 0.10 | 0.10 |  | 2.50 | 1.90 | 2.05 |
| L67 | 27.02 | 37.79 | 43.83 |  | 8.30 | 7.87 | 6.50 |  | 0.00 | 0.07 | 0.00 |  | 2.03 | 2.53 | 2.70 |
| L69 | 36.55 | 39.04 | 40.74 |  | 6.95 | 7.10 | 7.70 |  | 0.00 | 0.03 | 0.00 |  | 1.90 | 2.33 | 2.40 |
| L70 | 42.48 | 39.52 | 53.50 |  | 9.03 | 9.33 | 9.05 |  | 0.10 | 0.10 | 0.05 |  | 2.17 | 1.83 | 2.10 |
| L71 | 41.31 | 44.60 | 49.92 |  | 8.45 | 8.48 | 8.50 |  | 0.03 | 0.10 | 0.00 |  | 2.40 | 2.10 | 2.23 |
| L72 | 40.32 | 34.14 | 44.15 |  | 8.29 | 8.90 | 8.20 |  | 0.02 | 0.06 | 0.00 |  | 2.80 | 1.96 | 2.25 |
| L73 | 35.73 | 40.17 | 47.25 |  | 7.64 | 7.67 | 7.55 |  | 0.04 | 0.10 | 0.05 |  | 2.62 | 2.13 | 2.55 |
| L76 | 36.24 | 29.07 | 42.62 |  | 7.83 | 7.50 | 7.23 |  | 0.05 | 0.08 | 0.03 |  | 2.58 | 2.35 | 2.99 |
| L77 | 30.55 | 27.62 | 35.77 |  | 9.90 | 8.67 | 8.40 |  | 0.00 | 0.10 | 0.00 |  | 1.55 | 1.43 | 1.75 |
| L78 | 28.99 | 37.65 | 42.57 |  | 7.80 | 8.27 | 7.10 |  | 0.07 | 0.10 | 0.00 |  | 2.00 | 1.77 | 1.75 |
| L81 | 40.72 | 42.52 | 50.36 |  | 7.80 | 8.71 | 7.50 |  | 0.03 | 0.07 | 0.03 |  | 3.29 | 1.89 | 2.05 |
| L82 | 34.52 | 32.67 | 40.04 |  | 7.70 | 8.40 | 7.30 |  | 0.00 | 0.10 | 0.00 |  | 2.23 | 1.35 | 2.00 |
| L86 | 33.22 | 41.83 | 47.44 |  | 9.77 | 8.57 | 9.47 |  | 0.00 | 0.07 | 0.00 |  | 1.17 | 1.83 | 1.60 |
| L87 | 35.18 | 35.83 | 35.51 |  | 8.70 | 8.30 | 8.10 |  | 0.00 | 0.10 | 0.00 |  | 1.90 | 1.65 | 2.30 |
| L88 | 41.34 | 39.56 | 47.63 |  | 7.99 | 8.43 | 7.70 |  | 0.02 | 0.10 | 0.00 |  | 2.44 | 1.97 | 2.17 |
| L89 | 37.34 | 40.07 | 44.72 |  | 7.46 | 6.87 | 6.77 |  | 0.02 | 0.10 | 0.00 |  | 2.26 | 2.30 | 2.13 |
| L90 | 36.31 | 50.07 | 52.18 |  | 8.10 | 7.03 | 7.83 |  | 0.00 | 0.07 | 0.07 |  | 2.27 | 2.43 | 2.07 |
| L91 | 37.16 | 37.29 | 44.43 |  | 8.03 | 6.97 | 7.40 |  | 0.00 | 0.03 | 0.00 |  | 2.40 | 2.60 | 2.87 |
| L94 | 31.91 | 43.05 | 49.40 |  | 8.10 | 7.07 | 8.47 |  | 0.10 | 0.07 | 0.00 |  | 1.63 | 2.47 | 2.07 |
| L95 | 28.45 | 38.25 | 44.49 |  | 8.15 | 8.28 | 8.20 |  | 0.10 | 0.10 | 0.05 |  | 1.85 | 1.95 | 2.03 |
| L96 | 44.14 | 42.23 | 49.44 |  | 8.38 | 9.34 | 8.50 |  | 0.04 | 0.08 | 0.07 |  | 2.28 | 1.50 | 1.77 |
| L97 | 31.46 | 36.46 | 47.06 |  | 8.53 | 8.20 | 7.85 |  | 0.07 | 0.07 | 0.00 |  | 1.95 | 2.10 | 2.65 |
| L98 | 29.61 | 38.80 | 42.11 |  | 9.75 | 8.45 | 8.70 |  | 0.05 | 0.10 | 0.00 |  | 1.35 | 1.85 | 1.50 |
| L99 | 38.22 | 27.39 | 38.30 |  | 8.90 | 10.37 | 9.47 |  | 0.00 | 0.13 | 0.03 |  | 1.90 | 1.17 | 1.47 |
| L100 | 38.08 | 31.62 | 34.67 |  | 7.30 | 8.15 | 8.33 |  | 0.00 | 0.10 | 0.00 |  | 2.20 | 1.45 | 1.50 |
| L101 | 34.79 | 41.78 | 46.31 |  | 8.60 | 8.85 | 7.65 |  | 0.00 | 0.05 | 0.00 |  | 2.00 | 2.00 | 1.80 |
| L102 | 32.38 | 35.27 | 46.30 |  | 7.50 | 8.21 | 8.30 |  | 0.00 | 0.10 | 0.05 |  | 2.20 | 2.19 | 1.95 |
| L106 | 31.21 | 45.53 | 49.09 |  | 7.90 | 7.75 | 9.10 |  | 0.10 | 0.03 | 0.03 |  | 2.30 | 1.98 | 1.87 |
| L119 | 37.38 | 41.80 | 48.40 |  | 8.40 | 9.53 | 9.00 |  | 0.00 | 0.03 | 0.05 |  | 1.83 | 1.57 | 1.90 |
| L145 | 43.18 | 44.79 | 49.83 |  | 9.04 | 9.44 | 8.70 |  | 0.04 | 0.04 | 0.05 |  | 2.24 | 1.88 | 2.05 |
| L150 | 40.32 | 39.95 | 45.46 |  | 7.75 | 7.97 | 8.27 |  | 0.05 | 0.10 | 0.03 |  | 2.20 | 1.93 | 1.70 |
| L151 | 37.53 | 42.29 | 44.75 |  | 7.86 | 8.30 | 7.33 |  | 0.00 | 0.07 | 0.00 |  | 2.11 | 2.60 | 2.80 |
| L152 | 43.67 | 46.53 | 46.13 |  | 7.58 | 8.10 | 8.27 |  | 0.00 | 0.10 | 0.03 |  | 1.84 | 1.93 | 1.70 |
| L153 | 42.47 | 48.26 | 50.09 |  | 8.23 | 7.85 | 7.33 |  | 0.07 | 0.10 | 0.00 |  | 2.43 | 3.05 | 3.17 |
| L155 | 34.35 | 31.52 | 39.92 |  | 8.40 | 7.75 | 8.15 |  | 0.00 | 0.10 | 0.05 |  | 1.95 | 1.95 | 2.00 |
| L160 | 36.35 | 38.68 | 41.31 |  | 9.15 | 8.75 | 9.05 |  | 0.00 | 0.07 | 0.05 |  | 1.90 | 2.07 | 2.20 |
| L161 | 36.38 | 48.38 | 49.55 |  | 7.20 | 8.00 | 8.25 |  | 0.00 | 0.10 | 0.05 |  | 2.00 | 2.70 | 2.15 |
| L162 | 37.26 | 41.11 | 47.48 |  | 7.47 | 7.60 | 7.53 |  | 0.07 | 0.05 | 0.00 |  | 2.70 | 2.35 | 1.80 |
| L163 | 38.22 | 36.27 | 47.65 |  | 7.27 | 7.40 | 7.07 |  | 0.00 | 0.03 | 0.00 |  | 2.17 | 2.83 | 3.27 |
| L164 | 31.60 | 44.10 | 40.93 |  | 7.83 | 7.25 | 7.43 |  | 0.03 | 0.00 | 0.00 |  | 1.90 | 2.30 | 2.28 |
| L165 | 42.01 | 37.52 | 48.28 |  | 8.21 | 9.00 | 8.27 |  | 0.00 | 0.07 | 0.00 |  | 2.32 | 1.57 | 2.03 |
| L166 | 41.82 | 43.50 | 47.95 |  | 7.97 | 8.33 | 7.55 |  | 0.03 | 0.05 | 0.05 |  | 2.34 | 1.85 | 2.28 |
| L167 | 44.54 | 43.50 | 45.43 |  | 8.85 | 9.45 | 8.40 |  | 0.00 | 0.10 | 0.00 |  | 2.00 | 1.80 | 2.00 |
| L168 | 34.85 | 41.85 | 47.86 |  | 7.93 | 7.63 | 7.77 |  | 0.07 | 0.08 | 0.00 |  | 1.83 | 2.63 | 2.40 |
| L170 | 41.86 | 43.35 | 45.16 |  | 8.03 | 7.93 | 8.07 |  | 0.07 | 0.03 | 0.07 |  | 2.43 | 1.77 | 2.00 |
| L172 | 27.72 | 31.35 | 32.50 |  | 8.30 | 8.42 | 8.92 |  | 0.09 | 0.04 | 0.04 |  | 1.30 | 1.70 | 1.32 |
| L173 | 35.98 | 36.68 | 48.76 |  | 8.50 | 8.65 | 8.72 |  | 0.03 | 0.10 | 0.02 |  | 2.17 | 1.75 | 1.94 |
| L174 | 38.48 | 37.13 | 42.85 |  | 8.50 | 8.63 | 8.45 |  | 0.05 | 0.00 | 0.08 |  | 1.73 | 1.61 | 1.90 |
| L175 | 40.74 | 39.60 | 44.70 |  | 8.35 | 8.75 | 8.15 |  | 0.00 | 0.05 | 0.03 |  | 1.43 | 1.53 | 1.67 |
| L176 | 37.68 | 38.74 | 44.69 |  | 7.33 | 10.07 | 8.43 |  | 0.03 | 0.07 | 0.03 |  | 2.30 | 1.53 | 2.00 |
| L177 | 37.56 | 38.03 | 45.59 |  | 7.55 | 9.14 | 7.24 |  | 0.05 | 0.02 | 0.03 |  | 1.80 | 1.67 | 2.09 |
| L178 | 36.61 | 40.15 | 41.38 |  | 7.88 | 8.58 | 7.82 |  | 0.02 | 0.07 | 0.02 |  | 1.76 | 1.55 | 1.62 |
| L180 | 31.08 | 33.81 | 43.44 |  | 8.74 | 10.55 | 8.70 |  | 0.04 | 0.05 | 0.03 |  | 2.28 | 1.50 | 2.10 |
| L182 | 42.40 | 44.51 | 46.46 |  | 7.83 | 8.90 | 8.40 |  | 0.00 | 0.07 | 0.00 |  | 1.90 | 1.77 | 1.83 |
| L185 | 36.85 | 39.70 | 47.58 |  | 7.64 | 7.93 | 7.65 |  | 0.03 | 0.07 | 0.00 |  | 2.72 | 2.07 | 2.35 |
| L186 | 35.78 | 39.70 | 37.10 |  | 8.17 | 7.95 | 7.50 |  | 0.00 | 0.08 | 0.00 |  | 2.10 | 2.00 | 1.80 |
| L212 | 41.74 | 35.94 | 47.35 |  | 8.35 | 8.79 | 7.30 |  | 0.00 | 0.06 | 0.00 |  | 2.45 | 2.21 | 2.85 |
| L213 | 36.95 | 40.90 | 46.60 |  | 7.45 | 8.37 | 7.68 |  | 0.00 | 0.10 | 0.00 |  | 2.53 | 2.12 | 2.28 |
| L214 | 42.38 | 32.04 | 40.91 |  | 8.60 | 8.77 | 8.15 |  | 0.00 | 0.10 | 0.00 |  | 2.30 | 1.67 | 1.50 |
| L217 | 41.67 | 42.65 | 51.03 |  | 7.30 | 7.90 | 7.87 |  | 0.00 | 0.10 | 0.00 |  | 2.73 | 2.33 | 2.23 |
| L219 | 30.99 | 37.11 | 40.47 |  | 7.60 | 7.74 | 7.95 |  | 0.00 | 0.06 | 0.00 |  | 1.80 | 2.07 | 2.00 |
| L220 | 29.76 | 37.73 | 43.20 |  | 8.30 | 8.83 | 7.50 |  | 0.05 | 0.13 | 0.00 |  | 2.20 | 1.70 | 2.33 |
| L222 | 36.14 | 38.22 | 47.41 |  | 7.65 | 7.00 | 7.50 |  | 0.01 | 0.10 | 0.00 |  | 2.78 | 3.10 | 3.00 |
| L226 | 38.81 | 39.59 | 48.83 |  | 7.73 | 8.10 | 8.30 |  | 0.00 | 0.07 | 0.05 |  | 2.27 | 1.73 | 2.10 |
| L227 | 38.81 | 46.01 | 47.06 |  | 8.70 | 8.90 | 7.60 |  | 0.00 | 0.05 | 0.00 |  | 1.90 | 1.60 | 2.00 |
| L228 | 40.77 | 39.03 | 40.76 |  | 8.42 | 8.10 | 7.93 |  | 0.00 | 0.07 | 0.00 |  | 1.80 | 1.53 | 1.50 |
| L229 | NA | 41.84 | 42.72 |  | NA | 6.80 | 7.80 |  | NA | 0.05 | 0.00 |  | NA | 2.05 | 1.70 |
| L232 | 38.27 | 35.90 | 51.44 |  | 8.20 | 7.68 | 8.13 |  | 0.07 | 0.10 | 0.00 |  | 2.03 | 2.15 | 2.10 |
| L233 | 35.98 | 40.42 | 39.88 |  | 7.35 | 7.50 | 6.95 |  | 0.00 | 0.05 | 0.00 |  | 2.75 | 2.40 | 2.85 |
| L252 | 38.58 | 35.82 | 38.24 |  | 7.45 | 9.63 | 7.45 |  | 0.00 | 0.07 | 0.00 |  | 2.30 | 1.53 | 2.15 |
| L297 | 44.12 | 37.45 | 49.41 |  | 7.57 | 8.37 | 7.70 |  | 0.00 | 0.07 | 0.00 |  | 1.70 | 1.57 | 1.80 |
| L298 | 41.37 | 39.45 | 39.50 |  | 7.90 | 7.80 | 7.90 |  | 0.00 | 0.00 | 0.00 |  | 2.10 | 2.05 | 1.85 |
| L299 | 37.68 | 35.14 | 47.60 |  | 8.95 | 10.23 | 9.00 |  | 0.10 | 0.07 | 0.05 |  | 1.95 | 1.40 | 2.20 |
| L501 | 25.04 | 37.87 | 35.76 |  | 7.00 | 7.93 | 7.55 |  | 0.00 | 0.05 | 0.00 |  | 2.25 | 1.95 | 1.60 |
| L504 | NA | 31.84 | NA |  | NA | 9.20 | NA |  | NA | 0.00 | NA |  | NA | 1.40 | NA |
| L530 | 39.69 | 42.74 | 47.23 |  | 8.35 | 8.00 | 8.35 |  | 0.05 | 0.08 | 0.10 |  | 2.23 | 2.22 | 2.30 |
| L531 | 34.04 | 45.25 | 42.46 |  | 7.75 | 7.80 | 7.03 |  | 0.00 | 0.05 | 0.00 |  | 1.73 | 2.00 | 2.43 |
| L534 | 37.85 | 43.47 | NA |  | 7.40 | 8.63 | NA |  | 0.00 | 0.10 | NA |  | 2.05 | 1.93 | NA |
| L536 | 39.29 | 38.22 | 38.70 |  | 7.35 | 8.07 | 6.40 |  | 0.10 | 0.10 | 0.00 |  | 3.15 | 2.30 | 2.70 |
| L539 | 41.52 | 45.19 | 48.30 |  | 7.58 | 8.50 | 8.50 |  | 0.00 | 0.10 | 0.00 |  | 1.95 | 1.97 | 2.40 |
| L540 | 36.53 | 44.16 | 44.47 |  | 7.98 | 7.55 | 8.58 |  | 0.03 | 0.05 | 0.04 |  | 2.05 | 2.10 | 1.92 |
| L543 | 39.94 | 42.28 | 52.51 |  | 7.90 | 9.74 | 7.75 |  | 0.08 | 0.04 | 0.05 |  | 2.45 | 1.66 | 2.65 |
| L545 | 40.15 | 44.73 | 54.97 |  | 7.48 | 10.08 | 7.85 |  | 0.03 | 0.05 | 0.00 |  | 3.00 | 1.77 | 2.60 |
| L547 | 38.50 | 45.28 | 42.11 |  | 8.63 | 7.67 | 7.90 |  | 0.00 | 0.07 | 0.00 |  | 1.60 | 1.97 | 1.60 |
| L548 | 42.04 | 51.03 | 46.97 |  | 8.43 | 8.00 | 7.95 |  | 0.10 | 0.00 | 0.00 |  | 2.17 | 2.60 | 2.50 |
| L559 | 32.84 | 30.79 | 41.47 |  | 8.70 | 10.15 | 8.00 |  | 0.10 | 0.00 | 0.00 |  | 2.07 | 1.50 | 2.00 |
| L561 | 39.53 | 43.58 | 48.50 |  | 8.68 | 8.80 | 9.68 |  | 0.06 | 0.07 | 0.06 |  | 2.64 | 2.00 | 2.16 |
| L564 | 41.52 | 31.80 | 46.19 |  | 8.05 | 10.60 | 7.40 |  | 0.00 | 0.00 | 0.00 |  | 1.55 | 1.25 | 1.65 |
| L3001 | 34.45 | 43.09 | 44.48 |  | 7.24 | 9.70 | 8.20 |  | 0.06 | 0.03 | 0.10 |  | 2.22 | 1.50 | 2.00 |
| L3003 | 41.41 | 37.32 | 40.99 |  | 7.93 | 10.63 | 8.10 |  | 0.03 | 0.03 | 0.10 |  | 2.07 | 1.60 | 2.20 |
| L3007 | 43.24 | 47.60 | 50.85 |  | 7.70 | 8.40 | 8.40 |  | 0.03 | 0.05 | 0.05 |  | 2.27 | 2.25 | 2.05 |
| L3009 | 38.73 | 46.64 | 51.48 |  | 7.56 | 7.73 | 7.70 |  | 0.04 | 0.07 | 0.05 |  | 2.14 | 2.13 | 2.10 |
| L3011 | 41.52 | 39.23 | 40.53 |  | 8.40 | 8.40 | 9.07 |  | 0.10 | 0.10 | 0.03 |  | 1.80 | 1.50 | 1.47 |
| L3012 | 45.39 | 48.30 | 50.79 |  | 7.43 | 8.23 | 7.65 |  | 0.00 | 0.03 | 0.00 |  | 2.70 | 1.93 | 2.35 |
| L3014 | 39.57 | 41.77 | 45.16 |  | 8.07 | 8.50 | 8.40 |  | 0.10 | 0.10 | 0.05 |  | 1.77 | 1.73 | 1.70 |
| L3038 | 37.70 | 43.65 | 41.38 |  | 8.60 | 7.80 | 8.65 |  | 0.05 | 0.07 | 0.05 |  | 2.25 | 2.10 | 2.15 |
| L164-1 | 38.65 | 35.43 | 45.61 |  | 7.80 | 8.33 | 7.95 |  | 0.00 | 0.10 | 0.00 |  | 1.25 | 1.60 | 1.55 |
| L213-1 | 31.77 | 46.05 | 42.98 |  | 7.45 | 8.90 | 7.00 |  | 0.00 | 0.10 | 0.00 |  | 2.25 | 1.45 | 2.00 |
| LbendiCK | NA | 26.09 | NA |  | NA | 8.95 | NA |  | NA | 0.07 | NA |  | NA | 1.65 | NA |
| LDe1 | 34.64 | 26.61 | 31.76 |  | 6.20 | 8.83 | 6.50 |  | 0.00 | 0.00 | 0.10 |  | 3.37 | 1.50 | 2.70 |
| LFy1 | 38.96 | 36.46 | 47.77 |  | 8.05 | 9.65 | 7.65 |  | 0.00 | 0.03 | 0.00 |  | 1.45 | 1.45 | 1.65 |
| LFy2 | 42.34 | 41.05 | 43.16 |  | 8.07 | 7.90 | 8.35 |  | 0.07 | 0.05 | 0.00 |  | 1.83 | 1.80 | 1.55 |
| LFy5 | 37.15 | 43.23 | 43.40 |  | 8.65 | 7.63 | 8.22 |  | 0.05 | 0.03 | 0.01 |  | 2.03 | 2.00 | 1.88 |
| LFy7 | 42.89 | 41.76 | 47.84 |  | 7.70 | 9.95 | 7.40 |  | 0.03 | 0.01 | 0.00 |  | 1.97 | 1.58 | 2.00 |
| LFy9 | 40.19 | 45.85 | 48.10 |  | 8.93 | 9.57 | 8.85 |  | 0.03 | 0.03 | 0.05 |  | 2.20 | 2.07 | 2.30 |
| Lgan1 | NA | 42.25 | 48.48 |  | NA | 8.20 | 8.40 |  | NA | 0.10 | 0.00 |  | NA | 1.80 | 2.30 |
| Lgan12 | 38.10 | 29.94 | NA |  | 7.53 | 8.75 | NA |  | 0.00 | 0.10 | NA |  | 1.97 | 1.25 | NA |
| Lgan2 | NA | NA | NA |  | NA | NA | NA |  | NA | NA | NA |  | NA | NA | NA |
| Lgan3 | 36.75 | 46.23 | NA |  | 7.80 | 7.40 | NA |  | 0.00 | 0.00 | NA |  | 3.30 | 3.00 | NA |
| Lgan4 | 43.49 | 34.16 | NA |  | 8.15 | 10.35 | NA |  | 0.05 | 0.10 | NA |  | 3.15 | 1.80 | NA |
| Lgan5 | 36.90 | 39.16 | NA |  | 8.20 | 8.63 | NA |  | 0.03 | 0.03 | NA |  | 1.97 | 1.83 | NA |
| Lgan9 | 40.11 | 32.43 | NA |  | 8.33 | 8.77 | NA |  | 0.00 | 0.07 | NA |  | 1.83 | 1.83 | NA |
| Lganwu2 | NA | 34.68 | NA |  | NA | 8.52 | NA |  | NA | 0.09 | NA |  | NA | 1.53 | NA |
| Lgui1 | NA | 39.31 | NA |  | NA | 7.98 | NA |  | NA | 0.11 | NA |  | NA | 2.89 | NA |
| Lgui2 | NA | 40.12 | NA |  | NA | 8.09 | NA |  | NA | 0.00 | NA |  | NA | 1.95 | NA |
| Lgui3 | NA | 39.03 | NA |  | NA | 8.58 | NA |  | NA | 0.00 | NA |  | NA | 2.48 | NA |
| Lgui4 | NA | 34.02 | NA |  | NA | 9.45 | NA |  | NA | 0.09 | NA |  | NA | 2.01 | NA |
| Lguiruan2 | 40.14 | 29.77 | 33.72 |  | 7.97 | 8.37 | 7.80 |  | 0.00 | 0.00 | 0.10 |  | 2.60 | 2.20 | 2.40 |
| Lguiruan23 | 37.49 | 25.33 | 35.42 |  | 7.90 | 9.90 | 8.40 |  | 0.00 | 0.00 | 0.10 |  | 2.63 | 1.30 | 1.90 |
| Lguiruan3 | 44.65 | 35.94 | 42.10 |  | 7.63 | 8.90 | 7.80 |  | 0.03 | 0.00 | 0.10 |  | 3.43 | 2.17 | 3.10 |
| Ljiangxi23 | 40.59 | 41.90 | 46.52 |  | 8.03 | 8.80 | 7.80 |  | 0.03 | 0.10 | 0.10 |  | 2.40 | 1.87 | 2.20 |
| Lmin43 | 41.57 | 20.42 | NA |  | 8.63 | 11.23 | NA |  | 0.07 | 0.00 | NA |  | 2.20 | 0.93 | NA |
| Lmin48 | 38.63 | 20.77 | NA |  | 8.30 | 10.83 | NA |  | 0.03 | 0.00 | NA |  | 2.43 | 0.98 | NA |
| Lmin60 | 41.87 | 19.70 | NA |  | 8.20 | 11.80 | NA |  | 0.00 | 0.00 | NA |  | 2.43 | 0.80 | NA |
| Lminyou1 | NA | 16.44 | NA |  | NA | 8.56 | NA |  | NA | 0.10 | NA |  | NA | 1.38 | NA |
| Lminyou10 | NA | 16.55 | NA |  | NA | 7.96 | NA |  | NA | 0.10 | NA |  | NA | 2.62 | NA |
| Lminyou11 | NA | 27.12 | NA |  | NA | 7.78 | NA |  | NA | 0.11 | NA |  | NA | 1.49 | NA |
| Lminyou2 | NA | 18.83 | NA |  | NA | 7.80 | NA |  | NA | 0.07 | NA |  | NA | 1.67 | NA |
| Lminyou4 | NA | 17.15 | NA |  | NA | 7.18 | NA |  | NA | 0.10 | NA |  | NA | 2.81 | NA |
| Lminyou43 | NA | 15.29 | NA |  | NA | 8.81 | NA |  | NA | 0.11 | NA |  | NA | 2.23 | NA |
| Lminyou48 | NA | 26.75 | NA |  | NA | 9.38 | NA |  | NA | 0.10 | NA |  | NA | 1.83 | NA |
| Lminyou5 | NA | 36.52 | NA |  | NA | 8.32 | NA |  | NA | 0.00 | NA |  | NA | 2.24 | NA |
| Lminyou6 | NA | 41.29 | NA |  | NA | 7.13 | NA |  | NA | 0.08 | NA |  | NA | 2.38 | NA |
| Lminyou60 | NA | 13.59 | NA |  | NA | 10.24 | NA |  | NA | 0.14 | NA |  | NA | 1.30 | NA |
| Lminyou8 | NA | 14.53 | NA |  | NA | 10.00 | NA |  | NA | 0.10 | NA |  | NA | 1.28 | NA |
| Lminyou9 | NA | 21.37 | NA |  | NA | 8.75 | NA |  | NA | 0.09 | NA |  | NA | 1.70 | NA |
| Ltong10 | 39.69 | 42.50 | 48.87 |  | 8.67 | 8.77 | 8.60 |  | 0.00 | 0.10 | 0.00 |  | 1.80 | 1.67 | 2.03 |
| Ltong11 | 37.11 | 34.43 | 42.14 |  | 7.77 | 9.73 | 8.07 |  | 0.03 | 0.05 | 0.07 |  | 1.83 | 1.38 | 1.83 |
| Ltong12 | 39.93 | 41.28 | 35.75 |  | 7.37 | 9.20 | 8.30 |  | 0.03 | 0.00 | 0.00 |  | 2.50 | 2.15 | 2.35 |
| Ltong18 | 42.41 | 39.67 | 51.25 |  | 8.97 | 10.07 | 8.90 |  | 0.00 | 0.07 | 0.03 |  | 2.17 | 1.87 | 2.50 |
| Ltong29 | 32.55 | 22.61 | 27.14 |  | 8.17 | 10.13 | 8.85 |  | 0.07 | 0.06 | 0.10 |  | 1.80 | 1.45 | 1.70 |
| Ltong3 | 38.64 | 39.70 | 45.33 |  | 8.17 | 9.43 | 8.85 |  | 0.00 | 0.00 | 0.03 |  | 2.63 | 1.90 | 2.08 |
| Ltong30 | 46.93 | 42.74 | 53.04 |  | 8.70 | 9.18 | 9.30 |  | 0.05 | 0.06 | 0.10 |  | 1.88 | 1.60 | 1.35 |
| Ltong32 | 35.10 | 41.85 | 45.34 |  | 8.60 | 7.93 | 7.70 |  | 0.06 | 0.03 | 0.05 |  | 1.54 | 1.87 | 2.35 |
| Ltong38 | 28.90 | 33.94 | 37.20 |  | 9.87 | 11.04 | 8.75 |  | 0.10 | 0.07 | 0.05 |  | 1.43 | 1.44 | 1.90 |
| Ltong39 | 28.31 | 27.42 | 32.10 |  | 8.00 | 9.65 | 8.80 |  | 0.10 | 0.05 | 0.00 |  | 2.57 | 1.80 | 2.20 |
| Ltong4 | 32.69 | 35.49 | 46.93 |  | 8.07 | 9.58 | 8.00 |  | 0.10 | 0.08 | 0.03 |  | 2.40 | 1.80 | 2.13 |
| Ltong40 | 38.08 | 41.07 | 38.20 |  | 8.43 | 10.80 | 8.40 |  | 0.10 | 0.07 | 0.10 |  | 2.80 | 1.90 | 2.60 |
| Ltong41 | 42.18 | 38.72 | 47.82 |  | 6.96 | 8.85 | 7.28 |  | 0.06 | 0.03 | 0.05 |  | 2.87 | 1.40 | 2.08 |
| Ltong6 | 38.51 | 44.09 | 50.13 |  | 8.27 | 9.00 | 7.95 |  | 0.10 | 0.05 | 0.00 |  | 3.37 | 2.25 | 2.05 |
| Ltong9 | 44.97 | 45.45 | 53.05 |  | 8.00 | 9.00 | 7.80 |  | 0.00 | 0.10 | 0.00 |  | 1.80 | 1.88 | 2.00 |
| Lwu | 29.96 | 36.61 | 39.88 |  | 9.80 | 8.40 | 9.60 |  | 0.00 | 0.10 | 0.00 |  | 1.60 | 1.80 | 1.25 |
| Lwu-1 | 21.22 | 34.09 | 43.72 |  | 8.60 | 8.47 | 7.43 |  | 0.03 | 0.03 | 0.03 |  | 1.50 | 1.67 | 1.87 |
| Lwu5-2 | NA | 20.34 | NA |  | NA | 10.04 | NA |  | NA | 0.13 | NA |  | NA | 1.45 | NA |
| Lwupai5 | NA | 22.33 | 41.82 |  | NA | 10.05 | 7.90 |  | NA | 0.14 | 0.00 |  | NA | 1.50 | 1.80 |
| Lwuyiwu2 | NA | 23.70 | NA |  | NA | 6.73 | NA |  | NA | 0.00 | NA |  | NA | 2.13 | NA |
| Lxiang3 | 33.56 | 12.82 | 24.53 |  | 8.17 | 10.00 | 8.10 |  | 0.03 | 0.03 | 0.10 |  | 1.67 | 1.00 | 1.70 |
| Lxiang4 | 42.30 | 27.74 | 35.11 |  | 8.13 | 9.67 | 6.70 |  | 0.03 | 0.03 | 0.10 |  | 2.13 | 1.43 | 2.40 |
| Lxiang47 | 33.87 | 31.05 | 41.04 |  | 6.93 | 9.23 | 9.40 |  | 0.03 | 0.00 | 0.10 |  | 2.83 | 1.77 | 1.60 |
| Lxiang5 | 45.37 | 24.25 | 27.88 |  | 8.07 | 9.19 | 7.90 |  | 0.03 | 0.03 | 0.10 |  | 2.57 | 1.47 | 2.10 |
| Lxiang50 | 33.57 | 16.81 | 26.63 |  | 8.17 | 9.30 | 7.90 |  | 0.00 | 0.00 | 0.10 |  | 1.57 | 1.13 | 1.80 |
| Lxiang6 | 40.72 | 37.24 | 42.26 |  | 7.63 | 9.67 | 8.90 |  | 0.00 | 0.00 | 0.10 |  | 2.47 | 1.37 | 1.60 |
| Lxiang7 | NA | 24.51 | NA |  | NA | 9.12 | NA |  | NA | 0.10 | NA |  | NA | 1.46 | NA |
| Lxiang8 | 39.97 | 33.45 | 45.66 |  | 7.23 | 9.30 | 7.60 |  | 0.03 | 0.00 | 0.10 |  | 3.70 | 2.07 | 2.70 |
| Lxiang9 | NA | 31.33 | 50.55 |  | NA | 8.10 | 9.00 |  | NA | 0.00 | 0.10 |  | NA | 2.37 | 1.20 |
| Lyajia1 | NA | 24.04 | NA |  | NA | 7.68 | NA |  | NA | 0.10 | NA |  | NA | 2.16 | NA |
| Lyajia2 | NA | 25.72 | NA |  | NA | 9.58 | NA |  | NA | 0.13 | NA |  | NA | 1.64 | NA |
| Lyajia6 | NA | 32.93 | NA |  | NA | 8.33 | NA |  | NA | 0.00 | NA |  | NA | 1.81 | NA |
| Lyajia8 | NA | 15.21 | NA |  | NA | 9.12 | NA |  | NA | 0.11 | NA |  | NA | 1.49 | NA |
| Lyawu1 | NA | 34.86 | NA |  | NA | 8.62 | NA |  | NA | 0.12 | NA |  | NA | 1.66 | NA |
| Lyawu2 | NA | 18.79 | NA |  | NA | 8.51 | NA |  | NA | 0.11 | NA |  | NA | 1.54 | NA |
| Lyawu4 | NA | 13.53 | NA |  | NA | 10.50 | NA |  | NA | 0.14 | NA |  | NA | 1.09 | NA |
| Lwu5-1 | NA | 15.16 | NA |  | NA | 9.34 | NA |  | NA | 0.12 | NA |  | NA | 1.59 | NA |
| Lyawu6 | NA | 26.97 | NA |  | NA | 8.41 | NA |  | NA | 0.13 | NA |  | NA | 1.61 | NA |
| Lyawu7 | NA | 14.39 | NA |  | NA | 10.51 | NA |  | NA | 0.15 | NA |  | NA | 1.12 | NA |

Table S12-2

| Line | oleic acid | | |  | linoleic acid | | |  | linolenic acid | | |  | cis-11-eicosenoic acid | | |
| --- | --- | --- | --- | --- | --- | --- | --- | --- | --- | --- | --- | --- | --- | --- | --- |
|  | 2013 | 2014 | 2015 |  | 2013 | 2014 | 2015 |  | 2013 | 2014 | 2015 |  | 2013 | 2014 | 2015 |
| L1 | 80.98 | 76.83 | 79.73 |  | 7.40 | 11.43 | 8.20 |  | 0.23 | 0.30 | 0.20 |  | 0.50 | 0.53 | 0.57 |
| L2 | 81.10 | 79.50 | 79.70 |  | 8.40 | 9.75 | 10.13 |  | 0.20 | 0.20 | 0.30 |  | 0.40 | 0.45 | 0.47 |
| L3 | 81.05 | 72.91 | 78.98 |  | 7.41 | 14.91 | 9.83 |  | 0.28 | 0.39 | 0.33 |  | 0.49 | 0.46 | 0.55 |
| L4 | 81.20 | 79.35 | 80.93 |  | 7.67 | 9.33 | 8.37 |  | 0.27 | 0.30 | 0.30 |  | 0.50 | 0.42 | 0.53 |
| L8 | 80.33 | 75.43 | 79.83 |  | 7.84 | 12.40 | 8.37 |  | 0.20 | 0.25 | 0.20 |  | 0.43 | 0.50 | 0.50 |
| L9 | 82.60 | 79.70 | 79.47 |  | 6.70 | 9.33 | 9.23 |  | 0.30 | 0.23 | 0.27 |  | 0.50 | 0.50 | 0.50 |
| L10 | 83.15 | 82.43 | 83.33 |  | 6.18 | 6.40 | 5.80 |  | 0.25 | 0.20 | 0.20 |  | 0.47 | 0.53 | 0.57 |
| L11 | 82.73 | 83.07 | 81.30 |  | 6.83 | 6.77 | 8.07 |  | 0.30 | 0.20 | 0.23 |  | 0.60 | 0.57 | 0.57 |
| L12 | 80.45 | 81.03 | 80.50 |  | 8.10 | 8.33 | 8.70 |  | 0.20 | 0.23 | 0.30 |  | 0.55 | 0.53 | 0.55 |
| L15 | 82.13 | 79.57 | 80.87 |  | 6.83 | 9.63 | 8.67 |  | 0.27 | 0.27 | 0.23 |  | 0.50 | 0.57 | 0.53 |
| L16 | 83.17 | 81.85 | 80.47 |  | 6.73 | 7.50 | 8.40 |  | 0.30 | 0.25 | 0.30 |  | 0.37 | 0.55 | 0.50 |
| L17 | 78.87 | 80.10 | 78.65 |  | 8.99 | 8.80 | 9.75 |  | 0.28 | 0.30 | 0.30 |  | 0.51 | 0.50 | 0.50 |
| L18 | 81.19 | 77.74 | 80.80 |  | 7.39 | 10.67 | 8.35 |  | 0.33 | 0.30 | 0.30 |  | 0.49 | 0.51 | 0.50 |
| L19 | 80.70 | 78.17 | 77.70 |  | 7.50 | 10.67 | 9.90 |  | 0.30 | 0.30 | 0.30 |  | 0.50 | 0.60 | 0.55 |
| L20 | 80.13 | 77.70 | 79.05 |  | 9.00 | 11.20 | 10.15 |  | 0.30 | 0.30 | 0.30 |  | 0.50 | 0.60 | 0.50 |
| L21 | 82.27 | 80.28 | 79.20 |  | 6.50 | 8.40 | 8.85 |  | 0.30 | 0.20 | 0.30 |  | 0.40 | 0.53 | 0.55 |
| L22 | 81.17 | 77.03 | 80.60 |  | 7.47 | 10.67 | 8.65 |  | 0.30 | 0.27 | 0.30 |  | 0.50 | 0.43 | 0.45 |
| L23 | 79.83 | 79.97 | 80.30 |  | 8.90 | 8.67 | 8.60 |  | 0.27 | 0.20 | 0.25 |  | 0.47 | 0.43 | 0.45 |
| L24 | 83.30 | 78.77 | 82.35 |  | 5.87 | 9.70 | 7.10 |  | 0.30 | 0.27 | 0.30 |  | 0.60 | 0.53 | 0.60 |
| L26 | 81.50 | 81.40 | 82.33 |  | 7.00 | 7.90 | 7.18 |  | 0.40 | 0.30 | 0.30 |  | 0.53 | 0.53 | 0.50 |
| L27 | 79.60 | 79.90 | 76.75 |  | 8.45 | 9.38 | 11.35 |  | 0.33 | 0.28 | 0.30 |  | 0.48 | 0.56 | 0.55 |
| L28 | 81.00 | 78.83 | 82.60 |  | 8.05 | 10.50 | 7.15 |  | 0.20 | 0.27 | 4.15 |  | 0.50 | 0.50 | 0.55 |
| L29 | 84.00 | 84.13 | 84.80 |  | 6.05 | 6.00 | 5.35 |  | 0.30 | 0.23 | 0.25 |  | 0.55 | 0.50 | 0.55 |
| L30 | 80.56 | 79.05 | 82.45 |  | 7.82 | 9.10 | 6.60 |  | 0.23 | 0.25 | 0.20 |  | 0.50 | 0.53 | 0.60 |
| L31 | 80.77 | 80.15 | 81.90 |  | 8.01 | 9.45 | 7.30 |  | 0.27 | 0.20 | 0.20 |  | 0.54 | 0.55 | 0.45 |
| L32 | 84.00 | 84.20 | 83.60 |  | 4.93 | 4.10 | 6.10 |  | 0.30 | 0.20 | 0.20 |  | 0.50 | 0.40 | 0.45 |
| L39 | 72.40 | 78.47 | 82.65 |  | 11.97 | 10.50 | 6.85 |  | 0.53 | 0.37 | 0.30 |  | 0.54 | 0.47 | 0.50 |
| L40 | 82.58 | 77.35 | 80.80 |  | 6.40 | 11.16 | 7.75 |  | 0.23 | 0.27 | 0.20 |  | 0.42 | 0.47 | 0.45 |
| L42 | 80.27 | 80.27 | 78.72 |  | 8.60 | 9.12 | 10.53 |  | 0.27 | 0.28 | 0.30 |  | 0.47 | 0.55 | 0.57 |
| L43 | 80.73 | 80.00 | 78.90 |  | 8.33 | 9.23 | 9.35 |  | 0.30 | 0.23 | 0.30 |  | 0.60 | 0.55 | 0.65 |
| L44 | 80.95 | 79.37 | 80.50 |  | 7.65 | 9.80 | 8.30 |  | 0.30 | 0.30 | 0.30 |  | 0.50 | 0.50 | 0.50 |
| L45 | 82.05 | 80.00 | 81.70 |  | 7.01 | 9.45 | 7.85 |  | 0.26 | 0.20 | 0.30 |  | 0.53 | 0.55 | 0.55 |
| L46 | 81.50 | 80.58 | 82.75 |  | 8.60 | 9.05 | 8.10 |  | 0.30 | 0.20 | 0.20 |  | 0.60 | 0.53 | 0.55 |
| L49 | 78.46 | 81.65 | 81.75 |  | 10.29 | 7.78 | 7.70 |  | 0.55 | 0.30 | 0.35 |  | 0.66 | 0.58 | 0.65 |
| L52 | 78.01 | 78.00 | 77.85 |  | 10.88 | 11.05 | 10.85 |  | 0.38 | 0.25 | 0.20 |  | 0.55 | 0.55 | 0.55 |
| L53 | 81.58 | 77.38 | 79.43 |  | 7.60 | 11.76 | 9.83 |  | 0.28 | 0.32 | 0.25 |  | 0.49 | 0.48 | 0.55 |
| L54 | 80.83 | 81.15 | 82.15 |  | 8.30 | 8.25 | 7.40 |  | 0.33 | 0.20 | 0.20 |  | 0.50 | 0.50 | 0.50 |
| L55 | 79.95 | 82.60 | 80.05 |  | 9.30 | 6.60 | 8.65 |  | 0.35 | 0.20 | 0.20 |  | 0.50 | 0.60 | 0.50 |
| L56 | 76.50 | 75.25 | 76.45 |  | 11.60 | 13.10 | 11.85 |  | 0.37 | 0.35 | 0.30 |  | 0.53 | 0.60 | 0.60 |
| L58 | 80.59 | 78.35 | 80.20 |  | 8.14 | 10.30 | 8.40 |  | 0.24 | 0.30 | 0.20 |  | 0.50 | 0.55 | 0.50 |
| L59 | 80.50 | 80.23 | 79.30 |  | 8.00 | 8.43 | 9.15 |  | 0.32 | 0.27 | 0.30 |  | 0.52 | 0.57 | 0.55 |
| L60 | 78.51 | 78.60 | 78.65 |  | 9.41 | 9.65 | 9.30 |  | 0.30 | 0.20 | 0.25 |  | 0.52 | 0.50 | 0.55 |
| L61 | 81.09 | 78.87 | 79.35 |  | 7.06 | 9.13 | 8.55 |  | 0.25 | 0.20 | 0.20 |  | 0.51 | 0.53 | 0.50 |
| L63 | 81.73 | 78.90 | 79.80 |  | 7.21 | 10.00 | 8.90 |  | 0.24 | 0.23 | 0.20 |  | 0.50 | 0.50 | 0.50 |
| L64 | 84.00 | 82.17 | 82.75 |  | 5.70 | 6.80 | 6.85 |  | 0.00 | 0.20 | 0.20 |  | 0.60 | 0.50 | 0.60 |
| L65 | 81.27 | 81.67 | 82.80 |  | 6.66 | 7.27 | 6.25 |  | 0.28 | 0.23 | 0.25 |  | 0.48 | 0.53 | 0.55 |
| L66 | 80.55 | 78.75 | 81.25 |  | 7.80 | 10.15 | 7.95 |  | 0.20 | 0.30 | 0.25 |  | 0.50 | 0.60 | 0.55 |
| L67 | 79.73 | 80.67 | 84.30 |  | 9.03 | 8.13 | 5.70 |  | 0.37 | 0.27 | 0.20 |  | 0.53 | 0.43 | 0.60 |
| L69 | 84.25 | 81.80 | 80.40 |  | 6.20 | 7.90 | 8.60 |  | 0.20 | 0.20 | 0.20 |  | 0.50 | 0.60 | 0.70 |
| L70 | 79.90 | 76.07 | 78.35 |  | 8.10 | 11.83 | 9.75 |  | 0.20 | 0.23 | 0.25 |  | 0.53 | 0.50 | 0.45 |
| L71 | 80.75 | 78.27 | 78.77 |  | 7.68 | 10.27 | 9.72 |  | 0.23 | 0.25 | 0.23 |  | 0.45 | 0.50 | 0.50 |
| L72 | 80.99 | 78.54 | 81.20 |  | 7.14 | 9.80 | 7.50 |  | 0.29 | 0.27 | 0.25 |  | 0.51 | 0.46 | 0.65 |
| L73 | 82.20 | 80.93 | 81.73 |  | 6.70 | 8.37 | 7.40 |  | 0.32 | 0.27 | 0.28 |  | 0.48 | 0.50 | 0.48 |
| L76 | 81.55 | 81.32 | 82.77 |  | 7.23 | 7.92 | 6.20 |  | 0.30 | 0.35 | 0.27 |  | 0.48 | 0.48 | 0.51 |
| L77 | 76.95 | 77.60 | 79.05 |  | 10.70 | 11.33 | 10.00 |  | 0.35 | 0.33 | 0.35 |  | 0.55 | 0.53 | 0.55 |
| L78 | 80.03 | 77.47 | 81.35 |  | 9.23 | 11.60 | 9.00 |  | 0.30 | 0.30 | 0.20 |  | 0.60 | 0.50 | 0.55 |
| L81 | 82.06 | 81.01 | 82.98 |  | 6.10 | 7.71 | 6.70 |  | 0.21 | 0.21 | 0.20 |  | 0.47 | 0.43 | 0.57 |
| L82 | 82.18 | 75.00 | 82.23 |  | 6.98 | 14.00 | 7.47 |  | 0.30 | 0.40 | 0.30 |  | 0.59 | 0.70 | 0.60 |
| L86 | 75.17 | 79.07 | 75.87 |  | 13.13 | 9.77 | 12.23 |  | 0.33 | 0.27 | 0.30 |  | 0.40 | 0.43 | 0.57 |
| L87 | 80.40 | 78.55 | 79.45 |  | 8.30 | 10.55 | 9.20 |  | 0.30 | 0.30 | 0.35 |  | 0.40 | 0.50 | 0.60 |
| L88 | 82.31 | 78.40 | 81.80 |  | 6.53 | 10.30 | 7.57 |  | 0.23 | 0.27 | 0.23 |  | 0.49 | 0.53 | 0.53 |
| L89 | 84.08 | 83.23 | 85.03 |  | 5.36 | 6.70 | 5.23 |  | 0.23 | 0.23 | 0.20 |  | 0.57 | 0.60 | 0.60 |
| L90 | 80.77 | 83.50 | 82.63 |  | 7.87 | 6.20 | 6.63 |  | 0.27 | 0.20 | 0.20 |  | 0.50 | 0.53 | 0.50 |
| L91 | 81.93 | 82.47 | 82.03 |  | 6.87 | 7.07 | 6.83 |  | 0.30 | 0.27 | 0.33 |  | 0.50 | 0.60 | 0.57 |
| L94 | 79.00 | 82.40 | 78.23 |  | 10.37 | 7.27 | 10.50 |  | 0.30 | 0.23 | 0.23 |  | 0.50 | 0.50 | 0.47 |
| L95 | 79.51 | 77.73 | 78.50 |  | 9.50 | 11.20 | 10.48 |  | 0.33 | 0.25 | 0.23 |  | 0.57 | 0.55 | 0.53 |
| L96 | 81.74 | 79.24 | 80.33 |  | 6.84 | 9.14 | 8.53 |  | 0.22 | 0.20 | 0.20 |  | 0.48 | 0.46 | 0.53 |
| L97 | 77.13 | 79.17 | 81.45 |  | 7.43 | 9.63 | 7.25 |  | 0.36 | 0.30 | 0.30 |  | 0.54 | 0.53 | 0.55 |
| L98 | 75.55 | 78.90 | 78.70 |  | 12.30 | 9.90 | 10.30 |  | 0.40 | 0.30 | 0.30 |  | 0.50 | 0.50 | 0.50 |
| L99 | 80.40 | 70.93 | 76.83 |  | 8.00 | 16.10 | 11.37 |  | 0.30 | 0.50 | 0.30 |  | 0.50 | 0.50 | 0.50 |
| L100 | 82.40 | 75.80 | 74.33 |  | 7.40 | 13.60 | 14.90 |  | 0.10 | 0.30 | 0.37 |  | 0.60 | 0.60 | 0.57 |
| L101 | 80.60 | 77.05 | 83.20 |  | 8.00 | 11.30 | 6.55 |  | 0.30 | 0.25 | 0.25 |  | 0.60 | 0.45 | 0.55 |
| L102 | 82.70 | 80.28 | 80.80 |  | 7.00 | 8.42 | 8.10 |  | 0.30 | 0.31 | 0.25 |  | 0.30 | 0.53 | 0.55 |
| L106 | 81.30 | 81.35 | 80.13 |  | 7.50 | 8.13 | 8.17 |  | 0.50 | 0.25 | 0.23 |  | 0.50 | 0.55 | 0.50 |
| L119 | 81.77 | 77.50 | 78.05 |  | 7.27 | 10.70 | 10.10 |  | 0.27 | 0.30 | 0.30 |  | 0.50 | 0.43 | 0.55 |
| L145 | 79.78 | 79.30 | 79.90 |  | 8.18 | 8.70 | 8.55 |  | 0.21 | 0.18 | 0.20 |  | 0.49 | 0.46 | 0.50 |
| L150 | 82.20 | 79.97 | 79.83 |  | 7.05 | 9.30 | 9.47 |  | 0.25 | 0.20 | 0.23 |  | 0.50 | 0.53 | 0.57 |
| L151 | 82.16 | 79.00 | 83.07 |  | 7.16 | 4.80 | 6.07 |  | 0.23 | 0.13 | 0.20 |  | 0.43 | 0.37 | 0.50 |
| L152 | 83.15 | 80.00 | 80.17 |  | 6.79 | 9.17 | 9.03 |  | 0.26 | 0.23 | 0.30 |  | 0.37 | 0.50 | 0.53 |
| L153 | 81.87 | 80.15 | 82.63 |  | 6.70 | 8.15 | 6.17 |  | 0.23 | 0.20 | 0.20 |  | 0.47 | 0.50 | 0.50 |
| L155 | 79.45 | 81.30 | 80.35 |  | 9.35 | 8.20 | 8.70 |  | 0.30 | 0.25 | 0.30 |  | 0.50 | 0.50 | 0.50 |
| L160 | 78.80 | 78.33 | 79.50 |  | 9.35 | 10.04 | 8.50 |  | 0.30 | 0.26 | 0.30 |  | 0.50 | 0.51 | 0.50 |
| L161 | 83.43 | 81.10 | 81.50 |  | 6.73 | 7.60 | 7.40 |  | 0.20 | 0.20 | 0.20 |  | 0.40 | 0.50 | 0.50 |
| L162 | 82.70 | 81.90 | 83.30 |  | 6.27 | 7.30 | 6.50 |  | 0.33 | 0.25 | 0.20 |  | 0.53 | 0.55 | 0.50 |
| L163 | 84.10 | 83.97 | 84.33 |  | 5.80 | 4.97 | 4.53 |  | 0.23 | 0.20 | 0.20 |  | 0.43 | 0.53 | 0.57 |
| L164 | 80.73 | 82.10 | 82.43 |  | 8.70 | 7.55 | 7.13 |  | 0.30 | 0.20 | 0.25 |  | 0.47 | 0.50 | 0.53 |
| L165 | 81.27 | 76.33 | 80.77 |  | 7.47 | 12.27 | 8.13 |  | 0.24 | 0.30 | 0.20 |  | 0.49 | 0.57 | 0.57 |
| L166 | 82.50 | 79.92 | 82.28 |  | 6.51 | 9.17 | 7.08 |  | 0.23 | 0.22 | 0.20 |  | 0.47 | 0.47 | 0.55 |
| L167 | 80.65 | 74.00 | 78.43 |  | 7.80 | 13.90 | 10.37 |  | 0.20 | 0.30 | 0.20 |  | 0.40 | 0.50 | 0.50 |
| L168 | 80.10 | 80.20 | 80.80 |  | 9.17 | 8.73 | 8.23 |  | 0.33 | 0.23 | 0.23 |  | 0.57 | 0.53 | 0.53 |
| L170 | 80.07 | 79.37 | 79.40 |  | 8.70 | 10.00 | 9.67 |  | 0.20 | 0.30 | 0.23 |  | 0.50 | 0.60 | 0.53 |
| L172 | 78.21 | 80.08 | 76.53 |  | 11.00 | 9.06 | 12.28 |  | 0.45 | 0.30 | 0.36 |  | 0.61 | 0.48 | 0.58 |
| L173 | 80.15 | 78.33 | 79.00 |  | 8.35 | 10.38 | 9.52 |  | 0.27 | 0.27 | 0.28 |  | 0.50 | 0.53 | 0.52 |
| L174 | 81.28 | 81.11 | 80.00 |  | 7.70 | 7.97 | 8.80 |  | 0.23 | 0.23 | 0.25 |  | 0.50 | 0.43 | 0.53 |
| L175 | 81.05 | 78.57 | 80.77 |  | 8.40 | 10.33 | 8.57 |  | 0.32 | 0.25 | 0.22 |  | 0.42 | 0.53 | 0.60 |
| L176 | 84.00 | 78.23 | 80.63 |  | 5.47 | 9.40 | 7.97 |  | 0.23 | 0.20 | 0.27 |  | 0.57 | 0.50 | 0.67 |
| L177 | 81.88 | 79.34 | 81.77 |  | 7.95 | 9.12 | 8.03 |  | 0.25 | 0.26 | 0.23 |  | 0.52 | 0.45 | 0.57 |
| L178 | 81.10 | 80.38 | 81.15 |  | 8.46 | 8.82 | 8.53 |  | 0.26 | 0.22 | 0.27 |  | 0.50 | 0.43 | 0.62 |
| L180 | 79.26 | 75.05 | 79.60 |  | 8.78 | 12.15 | 8.80 |  | 0.38 | 0.33 | 0.30 |  | 0.50 | 0.40 | 0.53 |
| L182 | 81.73 | 78.30 | 79.10 |  | 7.87 | 10.23 | 9.77 |  | 0.23 | 0.23 | 0.23 |  | 0.40 | 0.53 | 0.57 |
| L185 | 82.95 | 80.63 | 82.70 |  | 5.91 | 8.53 | 6.55 |  | 0.27 | 0.27 | 0.20 |  | 0.49 | 2.03 | 0.50 |
| L186 | 80.57 | 79.30 | 82.00 |  | 8.37 | 9.90 | 7.70 |  | 0.30 | 0.25 | 0.25 |  | 0.50 | 0.58 | 0.60 |
| L212 | 80.90 | 79.09 | 83.55 |  | 7.50 | 9.16 | 5.60 |  | 0.30 | 0.25 | 0.30 |  | 0.50 | 0.43 | 0.50 |
| L213 | 83.35 | 78.47 | 82.00 |  | 5.85 | 10.14 | 7.23 |  | 0.28 | 0.31 | 0.20 |  | 0.58 | 0.55 | 0.58 |
| L214 | 80.70 | 76.63 | 79.30 |  | 7.60 | 12.07 | 10.35 |  | 0.30 | 0.33 | 0.25 |  | 0.50 | 0.50 | 0.50 |
| L217 | 83.37 | 80.00 | 80.50 |  | 5.90 | 8.93 | 8.57 |  | 0.20 | 0.20 | 0.23 |  | 0.53 | 0.60 | 0.57 |
| L219 | 83.00 | 82.40 | 81.45 |  | 7.00 | 7.06 | 7.75 |  | 0.20 | 0.22 | 0.30 |  | 0.50 | 0.51 | 0.60 |
| L220 | 80.53 | 75.73 | 82.57 |  | 8.13 | 12.83 | 6.80 |  | 0.33 | 0.30 | 0.23 |  | 0.50 | 0.50 | 0.50 |
| L222 | 83.45 | 83.35 | 83.30 |  | 5.48 | 5.75 | 5.50 |  | 0.22 | 0.20 | 0.25 |  | 0.43 | 0.50 | 0.50 |
| L226 | 82.17 | 79.80 | 80.35 |  | 7.07 | 9.57 | 8.65 |  | 0.23 | 0.27 | 0.10 |  | 0.50 | 0.43 | 0.45 |
| L227 | 78.10 | 78.55 | 81.00 |  | 10.30 | 10.15 | 8.40 |  | 0.40 | 0.25 | 0.20 |  | 0.50 | 0.45 | 0.60 |
| L228 | 81.89 | 82.03 | 81.80 |  | 7.06 | 7.53 | 7.80 |  | 0.34 | 0.30 | 0.33 |  | 0.51 | 0.50 | 0.57 |
| L229 | NA | 80.20 | 80.30 |  | NA | 10.00 | 9.40 |  | NA | 0.25 | 0.30 |  | NA | 0.65 | 0.60 |
| L232 | 80.80 | 79.48 | 79.80 |  | 8.20 | 9.88 | 9.30 |  | 0.23 | 0.25 | 0.20 |  | 0.50 | 0.50 | 0.50 |
| L233 | 83.15 | 80.25 | 83.45 |  | 5.85 | 9.05 | 5.70 |  | 0.30 | 0.20 | 0.35 |  | 0.55 | 0.55 | 0.60 |
| L252 | 83.65 | 76.30 | 82.70 |  | 5.90 | 11.80 | 6.90 |  | 0.20 | 0.23 | 0.20 |  | 0.50 | 0.47 | 0.60 |
| L297 | 83.10 | 78.23 | 81.95 |  | 7.00 | 11.00 | 7.80 |  | 0.27 | 0.23 | 0.30 |  | 0.40 | 0.53 | 0.50 |
| L298 | 81.40 | 80.70 | 81.75 |  | 7.80 | 8.65 | 7.65 |  | 0.30 | 0.25 | 0.25 |  | 0.50 | 0.60 | 0.60 |
| L299 | 79.90 | 76.57 | 78.35 |  | 8.45 | 11.07 | 9.70 |  | 0.20 | 0.27 | 0.20 |  | 0.50 | 0.43 | 0.50 |
| L501 | 81.65 | 79.80 | 79.40 |  | 8.00 | 9.40 | 10.50 |  | 0.30 | 0.28 | 0.30 |  | 0.70 | 0.58 | 0.60 |
| L504 | NA | 80.80 | NA |  | NA | 8.00 | NA |  | NA | 0.20 | NA |  | NA | 0.40 | NA |
| L530 | 79.03 | 78.60 | 78.65 |  | 9.68 | 10.26 | 9.70 |  | 0.30 | 0.30 | 0.30 |  | 0.45 | 0.52 | 0.50 |
| L531 | 81.55 | 80.45 | 82.60 |  | 8.10 | 8.85 | 7.00 |  | 0.30 | 0.25 | 0.30 |  | 0.55 | 0.60 | 0.60 |
| L534 | 82.13 | 76.63 | NA |  | 7.65 | 11.87 | NA |  | 0.23 | 0.27 | NA |  | 0.53 | 0.57 | NA |
| L536 | 83.35 | 80.13 | 84.30 |  | 5.25 | 8.60 | 5.70 |  | 0.30 | 0.27 | 0.30 |  | 0.50 | 0.57 | 0.60 |
| L539 | 83.73 | 77.67 | 79.10 |  | 6.13 | 11.07 | 9.20 |  | 0.20 | 0.23 | 0.20 |  | 0.43 | 0.50 | 0.50 |
| L540 | 81.30 | 80.85 | 78.63 |  | 7.95 | 8.60 | 10.03 |  | 0.25 | 0.30 | 0.20 |  | 0.48 | 0.55 | 0.59 |
| L543 | 81.40 | 79.04 | 80.60 |  | 7.40 | 8.92 | 8.35 |  | 0.30 | 0.25 | 0.25 |  | 0.48 | 0.32 | 0.45 |
| L545 | 83.00 | 80.14 | 83.00 |  | 5.78 | 7.45 | 6.00 |  | 0.25 | 0.20 | 0.20 |  | 0.43 | 0.30 | 0.40 |
| L547 | 78.95 | 79.37 | 80.00 |  | 9.95 | 10.10 | 9.60 |  | 0.38 | 0.23 | 0.30 |  | 0.50 | 0.60 | 0.60 |
| L548 | 78.83 | 78.10 | 79.40 |  | 9.77 | 10.60 | 9.40 |  | 0.20 | 0.20 | 0.20 |  | 0.50 | 0.50 | 0.50 |
| L559 | 77.30 | 76.20 | 78.00 |  | 11.03 | 11.50 | 11.30 |  | 0.30 | 0.30 | 0.30 |  | 0.50 | 0.35 | 0.40 |
| L561 | 79.42 | 78.67 | 77.22 |  | 8.54 | 9.73 | 10.16 |  | 0.30 | 0.23 | 0.24 |  | 0.42 | 0.53 | 0.42 |
| L564 | 80.95 | 74.95 | 81.05 |  | 8.55 | 12.55 | 9.15 |  | 0.20 | 0.30 | 0.20 |  | 0.60 | 0.35 | 0.55 |
| L3001 | 83.74 | 80.40 | 78.10 |  | 6.00 | 7.80 | 10.80 |  | 0.28 | 0.20 | 0.30 |  | 0.50 | 0.37 | 0.60 |
| L3003 | 81.43 | 76.17 | 81.60 |  | 7.93 | 10.90 | 7.30 |  | 0.20 | 0.27 | 0.20 |  | 0.43 | 0.37 | 0.60 |
| L3007 | 82.47 | 78.00 | 78.35 |  | 6.87 | 10.55 | 10.35 |  | 0.27 | 0.20 | 0.25 |  | 0.40 | 0.50 | 0.50 |
| L3009 | 81.72 | 79.37 | 81.50 |  | 7.76 | 9.93 | 7.90 |  | 0.34 | 0.23 | 0.30 |  | 0.46 | 0.50 | 0.45 |
| L3011 | 81.10 | 78.00 | 77.83 |  | 7.90 | 11.10 | 10.80 |  | 0.30 | 0.30 | 0.30 |  | 0.50 | 0.60 | 0.53 |
| L3012 | 83.33 | 79.43 | 80.75 |  | 5.73 | 9.57 | 8.50 |  | 0.30 | 0.30 | 0.25 |  | 0.50 | 0.53 | 0.55 |
| L3014 | 80.93 | 77.15 | 78.20 |  | 8.37 | 11.73 | 10.85 |  | 0.33 | 0.25 | 0.25 |  | 0.50 | 0.53 | 0.50 |
| L3038 | 80.15 | 79.93 | 80.60 |  | 8.20 | 9.33 | 7.80 |  | 0.30 | 0.27 | 0.25 |  | 0.48 | 0.53 | 0.50 |
| L164-1 | 83.55 | 79.00 | 81.55 |  | 6.85 | 10.10 | 8.05 |  | 0.20 | 0.23 | 0.20 |  | 0.40 | 0.57 | 0.60 |
| L213-1 | 83.15 | 74.40 | 82.90 |  | 6.35 | 14.35 | 7.20 |  | 0.30 | 0.30 | 0.20 |  | 0.50 | 0.55 | 0.50 |
| LbendiCK | NA | 79.12 | NA |  | NA | 9.41 | NA |  | NA | 0.29 | NA |  | NA | 0.52 | NA |
| LDe1 | 85.30 | 79.37 | 83.40 |  | 4.27 | 9.60 | 6.40 |  | 0.27 | 0.30 | 0.30 |  | 0.53 | 0.40 | 0.60 |
| LFy1 | 80.60 | 79.60 | 80.90 |  | 9.20 | 8.70 | 9.00 |  | 0.30 | 0.30 | 0.30 |  | 0.40 | 0.35 | 0.55 |
| LFy2 | 80.60 | 80.25 | 77.50 |  | 8.77 | 9.20 | 11.85 |  | 0.30 | 0.25 | 0.30 |  | 0.47 | 0.50 | 0.45 |
| LFy5 | 79.98 | 81.10 | 79.20 |  | 8.55 | 8.43 | 9.88 |  | 0.35 | 0.23 | 0.30 |  | 0.43 | 0.50 | 0.51 |
| LFy7 | 82.07 | 78.05 | 82.30 |  | 7.50 | 9.74 | 7.45 |  | 0.30 | 0.30 | 0.30 |  | 0.43 | 0.35 | 0.50 |
| LFy9 | 80.00 | 76.93 | 79.85 |  | 8.20 | 10.60 | 8.25 |  | 0.30 | 0.30 | 0.30 |  | 0.40 | 0.50 | 0.45 |
| Lgan1 | NA | 78.50 | 79.90 |  | NA | 10.50 | 8.70 |  | NA | 0.30 | 0.20 |  | NA | 0.60 | 0.60 |
| Lgan12 | 83.40 | 80.00 | NA |  | 6.47 | 9.10 | NA |  | 0.20 | 0.30 | NA |  | 0.47 | 0.55 | NA |
| Lgan2 | NA | NA | NA |  | NA | NA | NA |  | NA | NA | NA |  | NA | NA | NA |
| Lgan3 | 81.10 | 83.10 | NA |  | 7.10 | 5.80 | NA |  | 0.30 | 0.30 | NA |  | 0.50 | 0.50 | NA |
| Lgan4 | 81.90 | 74.55 | NA |  | 6.10 | 12.48 | NA |  | 0.25 | 0.35 | NA |  | 0.50 | 0.43 | NA |
| Lgan5 | 79.97 | 79.37 | NA |  | 9.07 | 9.37 | NA |  | 0.27 | 0.27 | NA |  | 0.50 | 0.53 | NA |
| Lgan9 | 79.97 | 79.10 | NA |  | 9.17 | 9.57 | NA |  | 0.23 | 0.23 | NA |  | 0.43 | 0.43 | NA |
| Lganwu2 | NA | 78.90 | NA |  | NA | 10.16 | NA |  | NA | 0.25 | NA |  | NA | 0.54 | NA |
| Lgui1 | NA | 81.66 | NA |  | NA | 6.57 | NA |  | NA | 0.21 | NA |  | NA | 0.51 | NA |
| Lgui2 | NA | 80.90 | NA |  | NA | 8.39 | NA |  | NA | 0.24 | NA |  | NA | 0.43 | NA |
| Lgui3 | NA | 80.73 | NA |  | NA | 7.58 | NA |  | NA | 0.20 | NA |  | NA | 0.43 | NA |
| Lgui4 | NA | 78.44 | NA |  | NA | 9.22 | NA |  | NA | 0.26 | NA |  | NA | 0.52 | NA |
| Lguiruan2 | 80.50 | 82.07 | 80.00 |  | 8.13 | 6.70 | 8.60 |  | 0.33 | 0.30 | 0.40 |  | 0.50 | 0.37 | 0.60 |
| Lguiruan23 | 81.47 | 77.43 | 79.20 |  | 7.20 | 10.67 | 9.40 |  | 0.30 | 0.30 | 0.40 |  | 0.53 | 0.33 | 0.60 |
| Lguiruan3 | 83.07 | 82.90 | 83.10 |  | 5.07 | 5.47 | 5.00 |  | 0.30 | 0.20 | 0.40 |  | 0.43 | 0.30 | 0.50 |
| Ljiangxi23 | 82.70 | 78.47 | 82.70 |  | 6.17 | 10.10 | 6.60 |  | 0.27 | 0.27 | 0.20 |  | 0.43 | 0.53 | 0.40 |
| Lmin43 | 80.77 | 73.17 | NA |  | 7.50 | 13.77 | NA |  | 0.40 | 0.53 | NA |  | 0.57 | 0.40 | NA |
| Lmin48 | 80.30 | 74.98 | NA |  | 8.17 | 12.30 | NA |  | 0.30 | 0.50 | NA |  | 0.53 | 0.40 | NA |
| Lmin60 | 82.23 | 71.35 | NA |  | 6.20 | 14.95 | NA |  | 0.30 | 0.60 | NA |  | 0.57 | 0.45 | NA |
| Lminyou1 | NA | 77.85 | NA |  | NA | 11.10 | NA |  | NA | 0.36 | NA |  | NA | 0.66 | NA |
| Lminyou10 | NA | 81.10 | NA |  | NA | 7.27 | NA |  | NA | 0.31 | NA |  | NA | 0.58 | NA |
| Lminyou11 | NA | 82.33 | NA |  | NA | 7.41 | NA |  | NA | 0.31 | NA |  | NA | 0.57 | NA |
| Lminyou2 | NA | 78.66 | NA |  | NA | 10.84 | NA |  | NA | 0.31 | NA |  | NA | 0.66 | NA |
| Lminyou4 | NA | 83.64 | NA |  | NA | 5.44 | NA |  | NA | 0.28 | NA |  | NA | 0.54 | NA |
| Lminyou43 | NA | 79.75 | NA |  | NA | 8.21 | NA |  | NA | 0.33 | NA |  | NA | 0.52 | NA |
| Lminyou48 | NA | 78.11 | NA |  | NA | 9.72 | NA |  | NA | 0.31 | NA |  | NA | 0.53 | NA |
| Lminyou5 | NA | 81.20 | NA |  | NA | 7.59 | NA |  | NA | 0.21 | NA |  | NA | 0.45 | NA |
| Lminyou6 | NA | 83.84 | NA |  | NA | 5.83 | NA |  | NA | 0.22 | NA |  | NA | 0.52 | NA |
| Lminyou60 | NA | 71.81 | NA |  | NA | 15.47 | NA |  | NA | 0.48 | NA |  | NA | 0.55 | NA |
| Lminyou8 | NA | 75.46 | NA |  | NA | 12.11 | NA |  | NA | 0.50 | NA |  | NA | 0.54 | NA |
| Lminyou9 | NA | 77.91 | NA |  | NA | 10.64 | NA |  | NA | 0.31 | NA |  | NA | 0.60 | NA |
| Ltong10 | 78.63 | 74.80 | 78.27 |  | 10.10 | 13.77 | 10.30 |  | 0.30 | 0.37 | 0.27 |  | 0.43 | 0.50 | 0.57 |
| Ltong11 | 81.53 | 77.65 | 79.60 |  | 8.03 | 10.48 | 9.67 |  | 0.30 | 0.30 | 0.30 |  | 0.53 | 0.43 | 0.53 |
| Ltong12 | 84.00 | 80.50 | 81.40 |  | 5.30 | 7.45 | 7.10 |  | 0.23 | 0.25 | 0.25 |  | 0.53 | 0.50 | 0.55 |
| Ltong18 | 77.20 | 74.80 | 76.63 |  | 11.00 | 12.57 | 11.13 |  | 0.30 | 0.27 | 0.30 |  | 0.37 | 0.37 | 0.47 |
| Ltong29 | 80.37 | 74.33 | 75.80 |  | 8.60 | 13.15 | 12.35 |  | 0.33 | 0.38 | 0.35 |  | 0.67 | 0.52 | 0.70 |
| Ltong3 | 81.20 | 76.80 | 76.88 |  | 7.17 | 11.10 | 11.35 |  | 0.27 | 0.23 | 0.30 |  | 0.60 | 0.53 | 0.58 |
| Ltong30 | 81.10 | 77.40 | 78.40 |  | 7.55 | 10.96 | 9.65 |  | 0.25 | 0.28 | 0.20 |  | 0.48 | 0.52 | 0.50 |
| Ltong32 | 77.98 | 79.00 | 80.75 |  | 10.88 | 10.30 | 8.30 |  | 0.38 | 0.23 | 0.30 |  | 0.56 | 0.57 | 0.60 |
| Ltong38 | 73.43 | 74.44 | 76.40 |  | 14.17 | 12.32 | 12.00 |  | 0.43 | 0.32 | 0.35 |  | 0.53 | 0.41 | 0.50 |
| Ltong39 | 81.57 | 75.75 | 77.45 |  | 6.73 | 11.85 | 10.55 |  | 0.40 | 0.40 | 0.45 |  | 0.60 | 0.50 | 0.65 |
| Ltong4 | 81.30 | 77.88 | 80.03 |  | 7.37 | 9.98 | 8.93 |  | 0.30 | 0.25 | 0.23 |  | 0.53 | 0.45 | 0.60 |
| Ltong40 | 81.67 | 77.63 | 81.70 |  | 6.13 | 9.03 | 6.30 |  | 0.33 | 0.30 | 0.40 |  | 0.53 | 0.40 | 0.60 |
| Ltong41 | 84.66 | 81.48 | 84.13 |  | 4.76 | 7.64 | 5.75 |  | 0.23 | 0.21 | 0.25 |  | 0.50 | 0.38 | 0.58 |
| Ltong6 | 81.93 | 81.15 | 82.25 |  | 5.60 | 6.90 | 6.95 |  | 0.27 | 0.20 | 0.20 |  | 0.50 | 0.45 | 0.55 |
| Ltong9 | 82.05 | 77.75 | 81.35 |  | 7.43 | 10.55 | 8.00 |  | 0.28 | 0.25 | 0.30 |  | 0.42 | 0.50 | 0.50 |
| Lwu | 76.00 | 76.90 | 74.95 |  | 11.90 | 11.90 | 13.20 |  | 0.30 | 0.30 | 0.35 |  | 0.50 | 0.50 | 0.50 |
| Lwu-1 | 78.53 | 79.30 | 81.63 |  | 10.30 | 9.77 | 8.13 |  | 0.43 | 0.27 | 0.27 |  | 0.60 | 0.50 | 0.57 |
| Lwu5-2 | NA | 72.29 | NA |  | NA | 15.29 | NA |  | NA | 0.28 | NA |  | NA | 0.53 | NA |
| Lwupai5 | NA | 72.46 | 81.15 |  | NA | 14.96 | 8.40 |  | NA | 0.41 | 0.20 |  | NA | 0.49 | 0.55 |
| Lwuyiwu2 | NA | 84.70 | NA |  | NA | 5.56 | NA |  | NA | 0.31 | NA |  | NA | 0.58 | NA |
| Lxiang3 | 80.23 | 75.37 | 77.90 |  | 8.93 | 12.73 | 11.10 |  | 0.37 | 0.47 | 0.50 |  | 0.60 | 0.47 | 0.60 |
| Lxiang4 | 80.60 | 78.70 | 84.20 |  | 8.40 | 9.50 | 5.90 |  | 0.27 | 0.27 | 0.30 |  | 0.43 | 0.37 | 0.60 |
| Lxiang47 | 83.47 | 80.03 | 76.70 |  | 5.93 | 8.33 | 11.20 |  | 0.27 | 0.20 | 0.30 |  | 0.47 | 0.33 | 0.50 |
| Lxiang5 | 82.10 | 78.50 | 80.20 |  | 6.50 | 10.05 | 8.70 |  | 0.23 | 0.34 | 0.50 |  | 0.47 | 0.45 | 0.60 |
| Lxiang50 | 80.27 | 77.90 | 80.30 |  | 9.00 | 10.83 | 8.70 |  | 0.37 | 0.37 | 0.50 |  | 0.60 | 0.40 | 0.70 |
| Lxiang6 | 81.93 | 79.00 | 77.30 |  | 7.23 | 9.40 | 11.30 |  | 0.30 | 0.27 | 0.30 |  | 0.47 | 0.33 | 0.50 |
| Lxiang7 | NA | 77.11 | NA |  | NA | 11.33 | NA |  | NA | 0.32 | NA |  | NA | 0.57 | NA |
| Lxiang8 | 82.73 | 80.87 | 82.10 |  | 5.57 | 7.17 | 6.80 |  | 0.20 | 0.20 | 0.20 |  | 0.50 | 0.37 | 0.50 |
| Lxiang9 | NA | 82.13 | 78.50 |  | NA | 6.72 | 10.40 |  | NA | 0.21 | 0.30 |  | NA | 0.47 | 0.60 |
| Lyajia1 | NA | 81.92 | NA |  | NA | 7.33 | NA |  | NA | 0.26 | NA |  | NA | 0.55 | NA |
| Lyajia2 | NA | 77.21 | NA |  | NA | 10.63 | NA |  | NA | 0.27 | NA |  | NA | 0.54 | NA |
| Lyajia6 | NA | 82.01 | NA |  | NA | 7.10 | NA |  | NA | 0.21 | NA |  | NA | 0.54 | NA |
| Lyajia8 | NA | 76.80 | NA |  | NA | 11.45 | NA |  | NA | 0.43 | NA |  | NA | 0.60 | NA |
| Lyawu1 | NA | 80.79 | NA |  | NA | 7.97 | NA |  | NA | 0.32 | NA |  | NA | 0.51 | NA |
| Lyawu2 | NA | 80.80 | NA |  | NA | 8.10 | NA |  | NA | 0.39 | NA |  | NA | 0.55 | NA |
| Lyawu4 | NA | 73.81 | NA |  | NA | 13.13 | NA |  | NA | 0.66 | NA |  | NA | 0.67 | NA |
| Lwu5-1 | NA | 78.53 | NA |  | NA | 9.32 | NA |  | NA | 0.46 | NA |  | NA | 0.64 | NA |
| Lyawu6 | NA | 77.90 | NA |  | NA | 11.11 | NA |  | NA | 0.37 | NA |  | NA | 0.47 | NA |
| Lyawu7 | NA | 72.72 | NA |  | NA | 14.11 | NA |  | NA | 0.68 | NA |  | NA | 0.65 | NA |

Table S12. Details of eight important oil traits in mature kernel of C. oleifera all accessions for three consecutive years (2013, 2014 and 2015). NA indicates a missing value.

| Sources of variation | | Year | Genotypes | Error |
| --- | --- | --- | --- | --- |
| *df* | | 2 | 220 | 450 |
| OC | MS | 1771.853 | 470.171 | 82.057 |
|  | *F* value | 21.593** | 5.73** |  |
| palmitic acid | MS | 304.6827 | 12.7433 | 4.5496 |
|  | *F* value | 66.97** | 2.801** |  |
| palmitoleic acid | MS | 0.1255 | 0.0011 | 0.0012 |
|  | *F* value | 101.095** | 0.89 |  |
| stearic acid | MS | 2.4784 | 1.1477 | 0.347 |
|  | *F* value | 7.142** | 3.307** |  |
| oleic acid | MS | 12866.344 | 1240.2258 | 389.86 |
|  | *F* value | 33.002** | 3.181** |  |
| linoleic acid | MS | 674.3882 | 17.9125 | 6.0907 |
|  | *F* value | 110.724** | 2.941** |  |
| linolenic acid | MS | 0.148 | 0.0396 | 0.0326 |
|  | *F* value | 4.546* | 1.217* |  |
| cis-11-eicosenoic acid | MS | 0.561 | 0.0553 | 0.0246 |
|  | *F* value | 22.78** | 2.246** |  |

Table S13. Analysis of variance for eight oil traits in the association population of *C. oleifera*.

MS, Mean squares for phenotype.

*, *P* < 0.05; **, *P* < 0.01.

| **Accession** | **SNP Number** | **Transition** | **Transversion** | **Ti/Tv** | **Heterozy-gosity** | **Homozygo-sity** | **Het-ratio** | **Function** | | | |
| --- | --- | --- | --- | --- | --- | --- | --- | --- | --- | --- | --- |
|  |  |  |  |  |  |  |  | **Synonymous SNV** | **Nonsynony-mous SNV** | **Stopgain** | **Stoploss** |
| L1 | 1,552,686 | 1,081,766 | 470,920 | 2.30 | 471,724 | 1,080,962 | 30.38% | 171,176 | 163,154 | 1,713 | 162 |
| L2 | 1,593,588 | 1,109,667 | 483,921 | 2.29 | 484,346 | 1,109,242 | 30.39% | 171,141 | 163,332 | 1,729 | 160 |
| L3 | 1,577,712 | 1,098,282 | 479,430 | 2.29 | 486,445 | 1,091,267 | 30.83% | 172,462 | 164,306 | 1,707 | 158 |
| L4 | 1,592,255 | 1,107,339 | 484,916 | 2.28 | 472,868 | 1,119,387 | 29.70% | 172,912 | 164,461 | 1,709 | 161 |
| L8 | 1,513,695 | 1,055,692 | 458,003 | 2.30 | 450,215 | 1,063,480 | 29.74% | 169,905 | 161,931 | 1,689 | 156 |
| L9 | 1,535,910 | 1,069,081 | 466,829 | 2.29 | 471,339 | 1,064,508 | 30.69% | 168,947 | 161,062 | 1,685 | 161 |
| L10 | 1,610,218 | 1,119,363 | 490,855 | 2.28 | 472,516 | 1,137,702 | 29.34% | 176,060 | 168,217 | 1,744 | 159 |
| L11 | 1,562,839 | 1,088,273 | 474,566 | 2.29 | 457,007 | 1,105,832 | 29.24% | 170,146 | 162,126 | 1,675 | 152 |
| L12 | 1,602,630 | 1,117,644 | 484,986 | 2.30 | 498,268 | 1,104,362 | 31.09% | 172,457 | 164,524 | 1,721 | 155 |
| L15 | 1,574,030 | 1,095,883 | 478,147 | 2.29 | 467,566 | 1,106,468 | 29.71% | 171,875 | 163,434 | 1,688 | 164 |
| L16 | 1,517,450 | 1,058,527 | 458,923 | 2.31 | 453,051 | 1,064,399 | 29.86% | 168,216 | 160,655 | 1,689 | 155 |
| L17 | 1,586,162 | 1,105,071 | 481,091 | 2.30 | 495,525 | 1,060,637 | 31.24% | 170,228 | 162,961 | 1,738 | 161 |
| L18 | 1,583,108 | 1,101,630 | 481,478 | 2.29 | 496,980 | 1,086,428 | 31.39% | 171,458 | 164,323 | 1,751 | 157 |
| L19 | 1,606,589 | 1,118,506 | 488,083 | 2.29 | 493,619 | 1,112,970 | 30.72% | 174,992 | 167,443 | 1,725 | 165 |
| L20 | 1,627,497 | 1,133,418 | 494,079 | 2.29 | 525,695 | 1,101,802 | 32.30% | 173,973 | 166,659 | 1,774 | 162 |
| L21 | 1,567,571 | 1,091,198 | 476,373 | 2.29 | 482,282 | 1,082,259 | 30.77% | 166,998 | 159,367 | 1,697 | 154 |
| L22 | 1,598,316 | 1,113,624 | 484,692 | 2.30 | 508,138 | 1,090,178 | 31.79% | 172,320 | 164,921 | 1,754 | 162 |
| L23 | 1,606,981 | 1,120,337 | 486,644 | 2.30 | 495,900 | 1,111,081 | 30.86% | 171,229 | 163,651 | 1,748 | 154 |
| L24 | 1,553,444 | 1,083,516 | 469,928 | 2.31 | 458,003 | 1,095,441 | 29.48% | 167,001 | 159,394 | 1,684 | 153 |
| L26 | 1,435,720 | 999,187 | 436,533 | 2.29 | 412,213 | 1,053,507 | 28.71% | 168,127 | 159,135 | 1,602 | 154 |
| L27 | 1,595,908 | 1,112,022 | 483,886 | 2.30 | 488,792 | 1,107,116 | 30.63% | 173,957 | 166,235 | 1,766 | 157 |
| L28 | 1,591,606 | 1,108,386 | 483,220 | 2.29 | 469,969 | 1,121,637 | 29.53% | 172,466 | 164,363 | 1,721 | 160 |
| L29 | 1,550,037 | 1,079,398 | 470,639 | 2.29 | 451,899 | 1,098,138 | 29.15% | 169,762 | 161,939 | 1,690 | 155 |
| L30 | 1,595,664 | 1,111,367 | 484,297 | 2.29 | 501,468 | 1,094,196 | 31.43% | 176,700 | 169,243 | 1,784 | 159 |
| L31 | 1,549,146 | 1,078,162 | 470,984 | 2.29 | 469,202 | 1,079,944 | 30.29% | 173,331 | 165,183 | 1,723 | 162 |
| L32 | 1,582,329 | 1,102,602 | 479,727 | 2.30 | 483,405 | 1,098,921 | 30.55% | 170,903 | 163,357 | 1,724 | 162 |
| L39 | 1,576,918 | 1,098,346 | 478,572 | 2.30 | 480,818 | 1,096,100 | 30.49% | 175,686 | 167,800 | 1,739 | 162 |
| L40 | 1,537,274 | 1,069,959 | 467,315 | 2.29 | 469,221 | 1,068,052 | 30.52% | 167,790 | 159,885 | 1,696 | 157 |
| L42 | 1,642,981 | 1,143,951 | 499,030 | 2.29 | 529,876 | 1,113,105 | 32.25% | 173,422 | 166,253 | 1,758 | 155 |
| L43 | 1,574,148 | 1,096,684 | 477,464 | 2.30 | 487,521 | 1,086,627 | 30.97% | 175,148 | 167,308 | 1,709 | 157 |
| L44 | 1,599,674 | 1,115,358 | 484,316 | 2.30 | 505,261 | 1,094,413 | 31.59% | 172,758 | 165,590 | 1,752 | 164 |
| L45 | 1,568,324 | 1,091,660 | 476,664 | 2.29 | 499,113 | 1,069,211 | 31.82% | 174,628 | 166,831 | 1,731 | 163 |
| L46 | 1,291,004 | 894,733 | 396,271 | 2.26 | 380,728 | 910,276 | 29.49% | 160,817 | 150,031 | 1,563 | 157 |
| L49 | 1,541,709 | 1,070,906 | 470,803 | 2.27 | 461,361 | 1,080,348 | 29.93% | 174,808 | 166,492 | 1,727 | 165 |
| L52 | 1,526,227 | 1,061,087 | 465,140 | 2.28 | 440,911 | 1,085,316 | 28.89% | 168,852 | 160,224 | 1,652 | 157 |
| L53 | 1,583,799 | 1,102,969 | 480,830 | 2.29 | 488,565 | 1,095,234 | 30.85% | 170,799 | 163,777 | 1,710 | 159 |
| L54 | 1,594,086 | 1,110,689 | 483,397 | 2.30 | 491,773 | 1,102,313 | 30.85% | 171,013 | 163,641 | 1,727 | 157 |
| L55 | 1,533,231 | 1,065,559 | 467,672 | 2.28 | 463,269 | 1,069,962 | 30.22% | 169,020 | 160,510 | 1,684 | 156 |
| L56 | 1,523,617 | 1,061,905 | 461,712 | 2.30 | 457,590 | 1,021,477 | 30.03% | 169,237 | 160,723 | 1,663 | 153 |
| L58 | 1,530,836 | 1,065,619 | 465,217 | 2.29 | 462,437 | 1,068,399 | 30.21% | 168,267 | 160,731 | 1,688 | 157 |
| L59 | 1,548,924 | 1,077,225 | 471,699 | 2.28 | 475,873 | 1,073,051 | 30.72% | 168,994 | 161,068 | 1,686 | 156 |
| L60 | 1,562,615 | 1,088,217 | 474,398 | 2.29 | 473,167 | 1,079,448 | 30.28% | 171,860 | 164,217 | 1,708 | 163 |
| L61 | 1,571,312 | 1,094,605 | 476,707 | 2.30 | 472,850 | 1,088,489 | 30.09% | 171,326 | 163,732 | 1,725 | 156 |
| L63 | 1,585,813 | 1,103,133 | 482,680 | 2.29 | 487,591 | 1,098,222 | 30.75% | 173,446 | 165,817 | 1,724 | 163 |
| L64 | 1,622,717 | 1,129,808 | 492,909 | 2.29 | 511,534 | 1,111,183 | 31.52% | 174,978 | 167,229 | 1,775 | 161 |
| L65 | 1,574,775 | 1,096,966 | 477,809 | 2.30 | 482,140 | 1,092,635 | 30.62% | 174,483 | 166,579 | 1,724 | 165 |
| L66 | 1,617,324 | 1,126,050 | 491,274 | 2.29 | 510,546 | 1,106,778 | 31.57% | 174,307 | 166,991 | 1,771 | 157 |
| L67 | 1,690,048 | 1,176,835 | 513,213 | 2.29 | 549,718 | 1,140,330 | 32.53% | 177,222 | 170,144 | 1,807 | 161 |
| L69 | 1,594,503 | 1,110,745 | 483,758 | 2.30 | 470,336 | 1,124,167 | 29.50% | 175,878 | 167,676 | 1,713 | 165 |
| L70 | 1,606,091 | 1,118,643 | 487,448 | 2.29 | 493,966 | 1,112,125 | 30.76% | 171,669 | 164,129 | 1,741 | 156 |
| L71 | 1,543,378 | 1,076,905 | 466,473 | 2.31 | 465,825 | 1,077,553 | 30.18% | 168,740 | 161,061 | 1,696 | 159 |
| L72 | 1,607,177 | 1,118,466 | 488,711 | 2.29 | 506,114 | 1,101,063 | 31.49% | 175,661 | 168,170 | 1,784 | 162 |
| L73 | 1,476,265 | 1,028,160 | 448,105 | 2.29 | 444,706 | 1,031,559 | 30.12% | 165,921 | 157,513 | 1,647 | 150 |
| L76 | 1,566,804 | 1,091,090 | 475,714 | 2.29 | 477,117 | 1,089,687 | 30.45% | 175,170 | 167,399 | 1,737 | 157 |
| L77 | 1,624,982 | 1,131,562 | 493,420 | 2.29 | 507,644 | 1,117,338 | 31.24% | 173,599 | 166,194 | 1,712 | 160 |
| L78 | 1,601,133 | 1,116,673 | 484,460 | 2.30 | 497,229 | 1,103,904 | 31.05% | 171,596 | 163,660 | 1,709 | 162 |
| L81 | 1,570,368 | 1,092,530 | 477,838 | 2.29 | 474,508 | 1,095,860 | 30.22% | 169,144 | 161,461 | 1,684 | 152 |
| L82 | 1,583,015 | 1,100,867 | 482,148 | 2.28 | 465,227 | 1,117,788 | 29.39% | 175,918 | 167,700 | 1,743 | 160 |
| L86 | 1,647,009 | 1,146,733 | 500,276 | 2.29 | 531,807 | 1,115,649 | 32.29% | 174,338 | 166,895 | 1,766 | 153 |
| L87 | 1,637,803 | 1,140,130 | 497,673 | 2.29 | 533,121 | 1,104,682 | 32.55% | 177,298 | 169,853 | 1,795 | 161 |
| L88 | 1,563,799 | 1,090,445 | 473,354 | 2.30 | 471,088 | 1,092,711 | 30.12% | 171,954 | 163,999 | 1,721 | 160 |
| L89 | 1,562,812 | 1,088,777 | 474,035 | 2.30 | 475,646 | 1,087,166 | 30.44% | 171,777 | 164,293 | 1,713 | 160 |
| L90 | 1,547,901 | 1,077,677 | 470,224 | 2.29 | 457,703 | 1,090,198 | 29.57% | 169,315 | 161,208 | 1,652 | 154 |
| L91 | 1,448,145 | 1,004,269 | 443,876 | 2.26 | 445,615 | 1,002,530 | 30.77% | 174,003 | 165,407 | 1,684 | 164 |
| L94 | 1,453,149 | 1,012,389 | 440,760 | 2.30 | 424,979 | 1,028,170 | 29.25% | 170,326 | 161,685 | 1,653 | 154 |
| L95 | 1,593,613 | 1,110,560 | 483,053 | 2.30 | 492,338 | 1,101,275 | 30.89% | 171,346 | 163,971 | 1,716 | 153 |
| L96 | 1,544,682 | 1,076,467 | 468,215 | 2.30 | 461,088 | 1,083,594 | 29.85% | 169,339 | 161,572 | 1,678 | 157 |
| L97 | 1,532,553 | 1,067,289 | 465,264 | 2.29 | 470,969 | 1,061,584 | 30.73% | 171,468 | 163,062 | 1,696 | 158 |
| L98 | 1,608,350 | 1,120,987 | 487,363 | 2.30 | 487,994 | 1,120,356 | 30.34% | 175,639 | 168,004 | 1,767 | 160 |
| L99 | 1,595,938 | 1,111,240 | 484,698 | 2.29 | 504,199 | 1,091,739 | 31.59% | 176,808 | 169,421 | 1,774 | 161 |
| L100 | 1,618,311 | 1,129,228 | 489,083 | 2.31 | 506,684 | 1,111,227 | 31.31% | 174,425 | 166,861 | 1,750 | 162 |
| L101 | 1,580,034 | 1,099,897 | 480,137 | 2.29 | 490,114 | 6,036,920 | 31.02% | 171,937 | 164,640 | 1,766 | 160 |
| L102 | 1,557,098 | 1,085,182 | 471,916 | 2.30 | 477,792 | 6,029,306 | 30.68% | 172,596 | 164,215 | 1,709 | 158 |
| L106 | 1,535,863 | 1,070,738 | 465,125 | 2.30 | 462,784 | 6,023,079 | 30.13% | 173,021 | 165,092 | 1,728 | 158 |
| L119 | 1,684,741 | 1,173,750 | 510,991 | 2.30 | 542,677 | 1,142,064 | 32.21% | 177,333 | 170,067 | 1,789 | 163 |
| L145 | 1,573,987 | 1,096,820 | 477,167 | 2.30 | 480,626 | 1,093,361 | 30.54% | 174,127 | 166,368 | 1,746 | 158 |
| L150 | 1,532,277 | 1,068,349 | 463,928 | 2.30 | 457,045 | 1,075,232 | 29.83% | 171,001 | 163,097 | 1,675 | 161 |
| L151 | 1,583,100 | 1,103,836 | 479,264 | 2.30 | 481,505 | 1,101,545 | 30.42% | 172,528 | 165,112 | 1,733 | 158 |
| L152 | 1,526,028 | 1,063,431 | 462,597 | 2.30 | 460,587 | 1,065,441 | 30.18% | 170,620 | 162,766 | 1,676 | 154 |
| L153 | 1,563,295 | 1,087,959 | 475,336 | 2.29 | 458,781 | 1,104,514 | 29.35% | 168,112 | 160,749 | 1,694 | 154 |
| L155 | 1,630,227 | 1,134,568 | 495,659 | 2.29 | 518,895 | 1,111,332 | 31.83% | 173,402 | 166,030 | 1,765 | 156 |
| L160 | 1,593,091 | 1,109,071 | 484,020 | 2.29 | 508,107 | 1,084,984 | 31.89% | 173,994 | 166,659 | 1,762 | 161 |
| L161 | 1,540,805 | 1,070,937 | 469,868 | 2.28 | 442,945 | 1,097,860 | 28.75% | 168,844 | 160,623 | 1,658 | 154 |
| L162 | 1,567,609 | 1,091,969 | 475,640 | 2.30 | 482,876 | 1,084,733 | 30.80% | 174,092 | 166,390 | 1,736 | 161 |
| L163 | 1,609,986 | 1,120,497 | 489,489 | 2.29 | 505,108 | 1,104,878 | 31.37% | 175,462 | 168,461 | 1,763 | 163 |
| L164 | 1,568,232 | 1,092,235 | 475,997 | 2.29 | 473,906 | 1,094,326 | 30.22% | 175,561 | 167,502 | 1,720 | 159 |
| L165 | 1,538,293 | 1,070,084 | 468,209 | 2.29 | 466,573 | 1,071,723 | 30.33% | 169,917 | 161,643 | 1,682 | 159 |
| L166 | 1,583,326 | 1,103,430 | 479,896 | 2.30 | 478,258 | 1,105,068 | 30.21% | 173,008 | 165,685 | 1,748 | 165 |
| L167 | 1,587,612 | 1,104,957 | 482,655 | 2.29 | 492,641 | 1,094,971 | 31.03% | 172,156 | 164,833 | 1,734 | 156 |
| L168 | 1,639,986 | 1,143,604 | 496,382 | 2.30 | 527,475 | 1,112,511 | 32.16% | 174,397 | 167,528 | 1,781 | 161 |
| L170 | 1,578,317 | 1,100,128 | 478,189 | 2.30 | 470,777 | 1,107,540 | 29.83% | 171,040 | 162,998 | 1,699 | 162 |
| L172 | 1,453,215 | 1,013,516 | 439,699 | 2.31 | 415,590 | 1,037,625 | 28.60% | 166,158 | 157,626 | 1,618 | 150 |
| L173 | 1,483,516 | 1,032,826 | 450,690 | 2.29 | 445,174 | 1,038,342 | 30.01% | 167,920 | 159,528 | 1,655 | 149 |
| L174 | 1,616,456 | 1,125,706 | 490,750 | 2.29 | 500,898 | 1,114,558 | 30.99% | 173,407 | 166,293 | 1,780 | 163 |
| L175 | 1,624,472 | 1,131,707 | 492,765 | 2.30 | 531,186 | 1,093,286 | 32.70% | 175,245 | 168,068 | 1,766 | 158 |
| L176 | 1,603,342 | 1,117,111 | 486,231 | 2.30 | 483,166 | 1,120,176 | 30.13% | 174,174 | 166,616 | 1,761 | 162 |
| L177 | 1,493,971 | 1,040,428 | 453,543 | 2.29 | 418,636 | 1,075,335 | 28.02% | 172,427 | 164,024 | 1,679 | 162 |
| L178 | 1,652,112 | 1,152,208 | 499,904 | 2.30 | 508,974 | 1,143,078 | 30.81% | 175,431 | 168,053 | 1,774 | 164 |
| L180 | 1,537,631 | 1,069,852 | 467,779 | 2.29 | 466,335 | 1,071,296 | 30.33% | 171,753 | 163,777 | 1,719 | 160 |
| L182 | 1,583,248 | 1,102,161 | 481,087 | 2.29 | 481,789 | 1,101,459 | 30.43% | 173,177 | 165,486 | 1,736 | 159 |
| L185 | 1,550,959 | 1,079,958 | 471,001 | 2.29 | 438,075 | 1,112,884 | 28.25% | 172,368 | 163,658 | 1,676 | 159 |
| L186 | 1,616,441 | 1,126,649 | 489,792 | 2.30 | 514,988 | 1,101,453 | 31.86% | 174,459 | 166,690 | 1,745 | 161 |
| L212 | 1,624,821 | 1,130,623 | 494,198 | 2.29 | 510,269 | 1,114,552 | 31.40% | 176,488 | 168,998 | 1,789 | 163 |
| L213 | 1,446,486 | 1,007,519 | 438,967 | 2.30 | 372,115 | 1,074,371 | 25.73% | 163,692 | 154,568 | 1,579 | 150 |
| L214 | 1,664,346 | 1,158,746 | 505,600 | 2.29 | 541,702 | 1,122,644 | 32.55% | 177,733 | 170,768 | 1,810 | 166 |
| L217 | 1,526,589 | 1,063,588 | 463,001 | 2.30 | 462,320 | 1,064,269 | 30.28% | 166,000 | 158,195 | 1,661 | 156 |
| L219 | 1,483,422 | 1,031,663 | 451,759 | 2.28 | 641,615 | 1,048,807 | 43.25% | 172,083 | 163,792 | 1,695 | 159 |
| L220 | 1,648,143 | 1,146,358 | 501,785 | 2.28 | 531,331 | 1,116,812 | 32.24% | 175,658 | 168,395 | 1,772 | 162 |
| L222 | 1,592,107 | 1,108,332 | 483,775 | 2.29 | 491,164 | 1,100,943 | 30.85% | 172,643 | 164,711 | 1,725 | 159 |
| L226 | 1,416,959 | 985,360 | 431,599 | 2.28 | 402,643 | 1,014,316 | 28.42% | 167,035 | 158,187 | 1,615 | 155 |
| L227 | 1,500,582 | 1,044,441 | 456,141 | 2.29 | 389,242 | 1,111,340 | 25.94% | 165,052 | 155,966 | 1,622 | 150 |
| L228 | 1,575,496 | 1,096,265 | 479,231 | 2.29 | 480,512 | 1,094,984 | 30.50% | 173,289 | 165,485 | 1,715 | 159 |
| L229 | 1,516,104 | 1,055,853 | 460,251 | 2.29 | 425,254 | 1,090,850 | 28.05% | 167,134 | 158,734 | 1,662 | 154 |
| L232 | 1,545,194 | 1,074,343 | 470,851 | 2.28 | 465,551 | 1,079,643 | 30.13% | 171,780 | 164,209 | 1,714 | 161 |
| L233 | 1,640,373 | 1,141,041 | 499,332 | 2.29 | 536,188 | 1,104,185 | 32.69% | 178,256 | 171,164 | 1,788 | 165 |
| L252 | 1,556,984 | 1,084,085 | 472,899 | 2.29 | 474,532 | 1,612,452 | 30.48% | 168,134 | 160,144 | 1,655 | 158 |
| L297 | 1,447,388 | 1,007,679 | 439,709 | 2.29 | 428,385 | 1,019,003 | 29.60% | 168,207 | 159,322 | 1,632 | 148 |
| L298 | 1,544,536 | 1,075,642 | 468,894 | 2.29 | 456,276 | 1,088,260 | 29.54% | 165,810 | 157,967 | 1,661 | 153 |
| L299 | 1,613,313 | 1,124,001 | 489,312 | 2.30 | 500,912 | 1,112,401 | 31.05% | 174,304 | 166,658 | 1,749 | 163 |
| L501 | 1,642,437 | 1,142,777 | 499,660 | 2.29 | 525,673 | 1,116,764 | 32.01% | 175,903 | 168,251 | 1,774 | 159 |
| L504 | 1,598,620 | 1,114,543 | 484,077 | 2.30 | 493,328 | 1,105,292 | 30.86% | 173,681 | 166,102 | 1,739 | 155 |
| L530 | 1,582,057 | 1,102,499 | 479,558 | 2.30 | 473,444 | 1,108,613 | 29.93% | 170,489 | 162,828 | 1,677 | 154 |
| L531 | 1,634,415 | 1,137,817 | 496,598 | 2.29 | 509,961 | 1,124,454 | 31.20% | 177,777 | 170,697 | 1,788 | 162 |
| L534 | 1,520,757 | 1,058,338 | 462,419 | 2.29 | 432,007 | 1,088,750 | 28.41% | 166,748 | 158,859 | 1,675 | 157 |
| L536 | 1,605,799 | 1,118,079 | 487,720 | 2.29 | 470,332 | 1,135,467 | 29.29% | 172,134 | 165,014 | 1,756 | 161 |
| L539 | 1,542,709 | 1,072,991 | 469,718 | 2.28 | 442,389 | 1,100,320 | 28.68% | 171,283 | 163,263 | 1,681 | 157 |
| L540 | 1,668,257 | 1,161,710 | 506,547 | 2.29 | 530,361 | 1,137,896 | 31.79% | 175,243 | 167,803 | 1,793 | 164 |
| L543 | 1,600,847 | 1,115,007 | 485,840 | 2.30 | 451,981 | 1,148,866 | 28.23% | 172,167 | 164,567 | 1,705 | 162 |
| L545 | 1,585,266 | 1,104,861 | 480,405 | 2.30 | 485,574 | 1,091,692 | 30.63% | 172,843 | 165,289 | 1,747 | 166 |
| L547 | 1,440,588 | 1,003,667 | 436,921 | 2.30 | 412,260 | 1,028,328 | 28.62% | 167,420 | 158,638 | 1,609 | 153 |
| L548 | 1,598,134 | 1,113,459 | 484,675 | 2.30 | 465,731 | 1,132,403 | 29.14% | 169,853 | 162,536 | 1,733 | 156 |
| L559 | 1,615,016 | 1,124,303 | 490,713 | 2.29 | 490,106 | 1,124,910 | 30.35% | 172,563 | 165,542 | 1,757 | 160 |
| L561 | 1,539,802 | 1,073,789 | 466,013 | 2.30 | 465,010 | 1,074,792 | 30.20% | 168,704 | 160,952 | 1,681 | 160 |
| L564 | 1,514,039 | 1,054,581 | 459,458 | 2.30 | 382,973 | 1,131,066 | 25.29% | 167,859 | 159,403 | 1,661 | 157 |
| L3001 | 1,537,686 | 1,071,880 | 465,806 | 2.30 | 466,779 | 1,070,907 | 30.36% | 169,303 | 161,374 | 1,698 | 163 |
| L3003 | 1,579,449 | 1,099,015 | 480,434 | 2.29 | 478,071 | 1,101,378 | 30.27% | 171,386 | 163,759 | 1,721 | 161 |
| L3007 | 1,511,315 | 1,053,031 | 458,284 | 2.30 | 435,572 | 1,075,743 | 28.82% | 164,878 | 156,908 | 1,644 | 147 |
| L3009 | 1,700,305 | 1,184,946 | 515,359 | 2.30 | 556,816 | 1,133,489 | 32.75% | 176,916 | 170,402 | 1,837 | 168 |
| L3011 | 1,538,859 | 1,071,455 | 467,404 | 2.29 | 465,692 | 1,073,167 | 30.26% | 170,749 | 162,636 | 1,697 | 154 |
| L3012 | 1,510,848 | 1,052,058 | 458,790 | 2.29 | 455,476 | 1,055,372 | 30.15% | 169,530 | 160,896 | 1,669 | 149 |
| L3014 | 1,509,336 | 1,050,044 | 459,292 | 2.29 | 437,886 | 1,071,450 | 29.01% | 167,690 | 159,509 | 1,659 | 154 |
| L3038 | 1,702,956 | 1,186,948 | 516,008 | 2.30 | 573,919 | 1,129,037 | 33.70% | 178,039 | 171,205 | 1,818 | 168 |
| L164-1 | 1,565,033 | 1,092,235 | 475,997 | 2.29 | 482,914 | 1,082,119 | 30.86% | 172,200 | 164,794 | 1,745 | 160 |
| L213-1 | 1,522,793 | 1,060,951 | 461,842 | 2.30 | 456,928 | 1,065,865 | 30.01% | 167,249 | 159,146 | 1,645 | 154 |
| LbendiCK | 1,594,668 | 1,109,914 | 484,754 | 2.29 | 458,570 | 1,136,098 | 28.76% | 172,985 | 165,430 | 1,721 | 159 |
| LDe1 | 1,639,936 | 1,141,639 | 498,297 | 2.29 | 522,752 | 1,117,184 | 31.88% | 176,192 | 168,896 | 1,800 | 160 |
| LFy1 | 1,633,133 | 1,137,450 | 495,683 | 2.29 | 528,250 | 1,154,883 | 32.35% | 175,803 | 168,819 | 1,812 | 166 |
| LFy2 | 1,580,832 | 1,102,297 | 478,535 | 2.30 | 503,193 | 1,077,639 | 31.83% | 171,887 | 164,677 | 1,742 | 163 |
| LFy5 | 1,497,240 | 1,042,932 | 454,308 | 2.30 | 443,364 | 1,053,876 | 29.61% | 165,787 | 157,558 | 1,651 | 154 |
| LFy7 | 1,543,186 | 1,074,917 | 468,269 | 2.30 | 468,724 | 1,074,462 | 30.37% | 168,082 | 160,379 | 1,679 | 158 |
| LFy9 | 1,525,534 | 1,063,465 | 462,069 | 2.30 | 444,533 | 1,081,001 | 29.14% | 169,837 | 161,762 | 1,673 | 154 |
| Lgan1 | 1,608,379 | 1,119,191 | 489,188 | 2.29 | 501,394 | 1,106,985 | 31.17% | 171,927 | 164,276 | 1,738 | 161 |
| Lgan12 | 1,623,843 | 1,130,783 | 493,060 | 2.29 | 513,503 | 1,110,340 | 31.62% | 176,464 | 168,934 | 1,777 | 165 |
| Lgan2 | 1,626,078 | 1,131,269 | 494,809 | 2.29 | 516,262 | 1,109,816 | 31.75% | 175,969 | 168,560 | 1,772 | 161 |
| Lgan3 | 1,627,084 | 1,134,416 | 492,668 | 2.30 | 517,414 | 1,109,670 | 31.80% | 174,231 | 167,288 | 1,788 | 164 |
| Lgan4 | 1,628,404 | 1,134,162 | 494,242 | 2.29 | 520,241 | 1,108,163 | 31.95% | 174,490 | 167,475 | 1,789 | 161 |
| Lgan5 | 1,607,792 | 1,118,843 | 488,949 | 2.29 | 502,638 | 1,105,154 | 31.26% | 175,076 | 167,664 | 1,772 | 165 |
| Lgan9 | 1,546,526 | 1,076,469 | 470,057 | 2.29 | 461,136 | 1,085,390 | 29.82% | 172,866 | 164,858 | 1,736 | 167 |
| Lganwu2 | 1,609,157 | 1,119,892 | 489,265 | 2.29 | 496,345 | 1,112,812 | 30.85% | 173,511 | 166,420 | 1,765 | 159 |
| Lgui1 | 1,591,359 | 1,108,525 | 482,834 | 2.30 | 485,027 | 1,106,332 | 30.48% | 171,922 | 164,300 | 1,718 | 156 |
| Lgui2 | 1,576,491 | 1,096,015 | 480,476 | 2.28 | 481,976 | 1,094,515 | 30.57% | 173,027 | 165,407 | 1,738 | 155 |
| Lgui3 | 1,566,503 | 1,090,122 | 476,381 | 2.29 | 485,221 | 1,081,282 | 30.97% | 175,627 | 167,980 | 1,737 | 162 |
| Lgui4 | 1,570,536 | 1,093,884 | 476,652 | 2.29 | 467,254 | 1,103,282 | 29.75% | 168,937 | 161,459 | 1,703 | 160 |
| Lguiruan2 | 1,537,693 | 1,068,403 | 469,290 | 2.28 | 465,706 | 1,071,987 | 30.29% | 175,237 | 167,590 | 1,756 | 167 |
| Lguiruan23 | 1,561,499 | 1,085,784 | 475,715 | 2.28 | 415,287 | 1,146,212 | 26.60% | 174,294 | 166,402 | 1,685 | 161 |
| Lguiruan3 | 1,548,982 | 1,075,951 | 473,031 | 2.27 | 450,241 | 1,098,651 | 29.07% | 176,010 | 168,307 | 1,757 | 166 |
| Ljiangxi23 | 1,574,940 | 1,097,078 | 477,862 | 2.30 | 487,867 | 1,087,073 | 30.98% | 170,734 | 163,334 | 1,725 | 157 |
| Lmin43 | 1,620,818 | 1,127,571 | 493,247 | 2.29 | 527,872 | 1,092,946 | 32.57% | 177,991 | 170,744 | 1,783 | 161 |
| Lmin48 | 1,660,601 | 1,154,897 | 505,704 | 2.28 | 538,356 | 1,122,245 | 32.42% | 177,816 | 170,870 | 1,804 | 163 |
| Lmin60 | 1,594,082 | 1,107,007 | 487,075 | 2.27 | 502,191 | 1,091,891 | 31.50% | 176,429 | 168,651 | 1,763 | 164 |
| Lminyou1 | 1,594,929 | 1,110,224 | 484,705 | 2.29 | 495,246 | 1,099,683 | 31.05% | 176,287 | 168,769 | 1,772 | 165 |
| Lminyou10 | 1,581,942 | 1,099,407 | 482,535 | 2.28 | 497,093 | 1,084,849 | 31.42% | 176,265 | 168,932 | 1,776 | 159 |
| Lminyou11 | 1,625,674 | 1,130,428 | 495,246 | 2.28 | 508,248 | 1,166,426 | 31.26% | 177,313 | 170,035 | 1,756 | 162 |
| Lminyou2 | 1,662,457 | 1,156,410 | 506,047 | 2.29 | 534,405 | 1,129,052 | 32.15% | 178,422 | 171,679 | 1,821 | 167 |
| Lminyou4 | 1,621,154 | 1,128,059 | 493,095 | 2.29 | 516,173 | 1,104,981 | 31.84% | 176,130 | 168,655 | 1,762 | 163 |
| Lminyou43 | 1,645,646 | 1,143,614 | 502,032 | 2.28 | 509,582 | 1,136,064 | 30.97% | 175,781 | 168,153 | 1,755 | 165 |
| Lminyou48 | 1,617,895 | 1,126,359 | 491,536 | 2.29 | 510,190 | 1,107,705 | 31.53% | 171,118 | 163,813 | 1,744 | 160 |
| Lminyou5 | 1,592,968 | 1,107,331 | 485,637 | 2.28 | 486,537 | 1,106,431 | 30.54% | 175,724 | 168,251 | 1,759 | 165 |
| Lminyou6 | 1,669,505 | 1,161,553 | 507,952 | 2.29 | 825,960 | 1,128,245 | 49.47% | 176,806 | 170,369 | 1,805 | 161 |
| Lminyou60 | 1,639,851 | 1,141,878 | 497,973 | 2.29 | 524,917 | 1,114,934 | 32.01% | 174,548 | 167,786 | 1,797 | 159 |
| Lminyou8 | 1,602,981 | 1,115,749 | 487,232 | 2.29 | 499,853 | 1,103,128 | 31.18% | 177,039 | 169,739 | 1,747 | 164 |
| Lminyou9 | 1,668,470 | 1,161,575 | 506,895 | 2.29 | 539,817 | 1,128,653 | 32.35% | 177,118 | 170,314 | 1,814 | 163 |
| Ltong10 | 1,593,081 | 1,109,706 | 483,375 | 2.30 | 483,306 | 1,109,775 | 30.34% | 169,861 | 162,052 | 1,725 | 158 |
| Ltong11 | 1,429,576 | 999,579 | 429,997 | 2.32 | 422,103 | 977,473 | 29.53% | 169,230 | 160,155 | 1,673 | 152 |
| Ltong12 | 1,614,168 | 1,123,885 | 490,283 | 2.29 | 507,698 | 1,106,470 | 31.45% | 175,443 | 167,801 | 1,773 | 163 |
| Ltong18 | 1,500,537 | 1,044,781 | 455,756 | 2.29 | 444,566 | 1,055,971 | 29.63% | 169,768 | 161,660 | 1,668 | 156 |
| Ltong29 | 1,600,337 | 1,113,658 | 486,679 | 2.29 | 505,412 | 1,084,925 | 31.58% | 175,830 | 168,138 | 1,743 | 163 |
| Ltong3 | 1,513,145 | 1,054,490 | 458,655 | 2.30 | 450,778 | 1,062,367 | 29.79% | 167,294 | 159,545 | 1,642 | 152 |
| Ltong30 | 1,617,512 | 1,126,564 | 490,948 | 2.29 | 513,347 | 1,104,165 | 31.74% | 174,302 | 167,449 | 1,763 | 164 |
| Ltong32 | 1,601,692 | 1,116,675 | 485,017 | 2.30 | 497,146 | 1,104,546 | 31.04% | 174,241 | 166,825 | 1,749 | 165 |
| Ltong38 | 1,622,068 | 1,130,229 | 491,839 | 2.30 | 515,634 | 1,106,434 | 31.79% | 173,563 | 166,362 | 1,759 | 160 |
| Ltong39 | 1,599,048 | 1,113,805 | 485,243 | 2.30 | 501,303 | 1,097,745 | 31.35% | 174,885 | 167,453 | 1,744 | 162 |
| Ltong4 | 1,563,482 | 1,089,831 | 473,651 | 2.30 | 465,820 | 1,097,662 | 29.79% | 168,931 | 161,309 | 1,688 | 161 |
| Ltong40 | 1,579,769 | 1,100,126 | 479,643 | 2.29 | 479,296 | 1,100,473 | 30.34% | 171,725 | 163,926 | 1,727 | 159 |
| Ltong41 | 1,615,476 | 1,125,252 | 490,224 | 2.30 | 487,628 | 1,117,848 | 30.18% | 173,994 | 166,827 | 1,767 | 165 |
| Ltong6 | 1,479,779 | 1,029,882 | 449,897 | 2.29 | 439,289 | 1,041,490 | 29.69% | 167,352 | 159,363 | 1,668 | 157 |
| Ltong9 | 1,619,394 | 1,128,774 | 490,620 | 2.30 | 531,153 | 1,088,241 | 32.80% | 174,100 | 167,108 | 1,787 | 164 |
| Lwu | 1,598,542 | 1,115,234 | 483,308 | 2.31 | 493,455 | 1,105,087 | 30.87% | 174,945 | 167,383 | 1,739 | 163 |
| Lwu-1 | 1,606,598 | 1,119,006 | 487,592 | 2.29 | 515,961 | 1,091,637 | 32.12% | 178,020 | 170,744 | 1,793 | 167 |
| Lwu5-1 | 1,626,263 | 1,132,335 | 493,928 | 2.29 | 518,284 | 1,107,969 | 31.87% | 174,289 | 166,656 | 1,765 | 162 |
| Lwu5-2 | 1,553,932 | 1,081,261 | 472,671 | 2.29 | 464,940 | 1,088,989 | 29.92% | 171,249 | 163,504 | 1,704 | 160 |
| Lwupai5 | 1,613,738 | 1,122,830 | 490,908 | 2.29 | 506,899 | 1,106,839 | 31.41% | 177,548 | 170,406 | 1,786 | 168 |
| Lwuyiwu2 | 1,605,227 | 1,116,987 | 488,240 | 2.29 | 495,802 | 1,109,425 | 30.89% | 170,864 | 163,773 | 1,759 | 154 |
| Lxiang3 | 1,620,200 | 1,126,964 | 493,236 | 2.28 | 511,336 | 1,108,828 | 31.56% | 176,673 | 169,103 | 1,768 | 163 |
| Lxiang4 | 1,541,612 | 1,073,484 | 468,128 | 2.29 | 453,387 | 1,088,235 | 29.41% | 170,678 | 162,772 | 1,700 | 152 |
| Lxiang47 | 1,584,425 | 1,103,525 | 480,900 | 2.29 | 485,189 | 1,099,236 | 30.62% | 173,760 | 166,009 | 1,752 | 157 |
| Lxiang5 | 1,625,315 | 1,130,445 | 494,870 | 2.28 | 526,390 | 1,098,475 | 32.39% | 177,621 | 170,528 | 1,802 | 163 |
| Lxiang50 | 1,616,682 | 1,124,469 | 492,213 | 2.28 | 516,069 | 1,100,613 | 31.92% | 176,220 | 168,956 | 1,780 | 162 |
| Lxiang6 | 1,650,640 | 1,149,734 | 500,906 | 2.30 | 515,440 | 1,135,200 | 31.23% | 174,705 | 167,460 | 1,778 | 159 |
| Lxiang7 | 1,642,376 | 1,143,284 | 499,092 | 2.29 | 517,018 | 1,115,358 | 31.48% | 177,087 | 170,055 | 1,795 | 164 |
| Lxiang8 | 1,560,198 | 1,085,582 | 474,616 | 2.29 | 472,484 | 1,087,714 | 30.28% | 173,221 | 165,498 | 1,685 | 161 |
| Lxiang9 | 1,604,141 | 1,117,016 | 487,125 | 2.29 | 500,149 | 1,103,992 | 31.18% | 172,862 | 165,594 | 1,760 | 163 |
| Lyajia1 | 1,437,841 | 1,001,135 | 436,706 | 2.29 | 408,712 | 994,489 | 28.43% | 164,079 | 156,066 | 1,605 | 151 |
| Lyajia2 | 1,587,098 | 1,104,083 | 483,015 | 2.29 | 496,714 | 1,090,384 | 31.30% | 174,898 | 166,792 | 1,760 | 162 |
| Lyajia6 | 1,604,751 | 1,116,721 | 488,030 | 2.29 | 489,269 | 1,114,482 | 30.49% | 172,998 | 165,570 | 1,734 | 155 |
| Lyajia8 | 1,544,307 | 1,074,003 | 470,304 | 2.28 | 477,267 | 1,067,040 | 30.90% | 176,352 | 168,988 | 1,774 | 161 |
| Lyawu1 | 1,612,117 | 1,121,879 | 490,238 | 2.29 | 496,964 | 1,115,153 | 30.83% | 173,581 | 166,147 | 1,758 | 159 |
| Lyawu2 | 1,614,252 | 1,122,486 | 491,766 | 2.28 | 514,689 | 1,099,563 | 31.88% | 177,124 | 170,034 | 1,777 | 155 |
| Lyawu4 | 1,629,802 | 1,132,897 | 496,905 | 2.28 | 533,357 | 1,096,445 | 32.73% | 177,986 | 170,771 | 1,797 | 167 |
| Lyawu6 | 1,603,292 | 1,116,290 | 487,002 | 2.29 | 470,489 | 1,131,803 | 29.35% | 172,402 | 164,934 | 1,757 | 161 |
| Lyawu7 | 1,547,889 | 1,073,986 | 473,903 | 2.27 | 493,850 | 1,054,039 | 31.90% | 176,617 | 169,190 | 1,775 | 163 |
| all | 1,849,953 | 1,288,570 | 561,383 | 2.30 | / | / | / | 183,483 | 178,198 | 1,959 | 172 |

Table S14. Statistics of SNPs of *C. oleifera* association population in this study

| **sample** | **CDS-Insertion** | **CDS-Deletion** | **CDS-Het** | **CDS-Homo** | **CDS-Total** | **Genome-Insertion** | **Genome-Deletion** | **Genome-Het** | **Genome-Homo** | **Genome-Total** |
| --- | --- | --- | --- | --- | --- | --- | --- | --- | --- | --- |
| L1 | 220 | 214 | 198 | 236 | 434 | 28,613 | 41,388 | 26,217 | 43,784 | 70,001 |
| L2 | 223 | 219 | 188 | 254 | 442 | 29,675 | 39,699 | 27,560 | 41,814 | 69,374 |
| L3 | 220 | 218 | 190 | 248 | 438 | 28,795 | 41,790 | 25,173 | 45,412 | 70,585 |
| L4 | 225 | 219 | 194 | 250 | 444 | 29,688 | 42,883 | 26,908 | 45,663 | 72,571 |
| L8 | 213 | 214 | 192 | 235 | 427 | 27,576 | 39,864 | 24,000 | 43,440 | 67,440 |
| L9 | 217 | 211 | 186 | 242 | 428 | 28,013 | 40,288 | 24,015 | 44,286 | 68,301 |
| L10 | 223 | 217 | 184 | 256 | 440 | 20,274 | 43,584 | 18,019 | 45,839 | 63,858 |
| L11 | 224 | 215 | 172 | 267 | 439 | 29,001 | 41,849 | 26,128 | 44,722 | 70,850 |
| L12 | 221 | 219 | 178 | 262 | 440 | 29,466 | 42,663 | 26,656 | 45,473 | 72,129 |
| L15 | 223 | 209 | 182 | 250 | 432 | 29,221 | 39,168 | 25,950 | 42,439 | 68,389 |
| L16 | 222 | 208 | 174 | 256 | 430 | 27,788 | 39,804 | 23,999 | 43,593 | 67,592 |
| L17 | 222 | 211 | 183 | 250 | 433 | 29,297 | 42,012 | 26,227 | 45,082 | 71,309 |
| L18 | 226 | 214 | 172 | 268 | 440 | 29,230 | 42,202 | 26,710 | 44,722 | 71,432 |
| L19 | 224 | 214 | 175 | 263 | 438 | 30,016 | 43,120 | 27,812 | 45,324 | 73,136 |
| L20 | 221 | 219 | 198 | 242 | 440 | 30,278 | 43,637 | 28,629 | 45,286 | 73,915 |
| L21 | 215 | 209 | 179 | 245 | 424 | 29,096 | 41,747 | 26,211 | 44,632 | 70,843 |
| L22 | 221 | 214 | 182 | 253 | 435 | 29,785 | 42,688 | 27,951 | 44,522 | 72,473 |
| L23 | 220 | 210 | 183 | 247 | 430 | 29,703 | 42,741 | 27,420 | 45,024 | 72,444 |
| L24 | 218 | 216 | 179 | 255 | 434 | 28,668 | 41,258 | 25,162 | 44,764 | 69,926 |
| L26 | 220 | 210 | 175 | 255 | 430 | 26,507 | 38,221 | 22,428 | 42,300 | 64,728 |
| L27 | 227 | 219 | 187 | 259 | 446 | 29,542 | 42,415 | 26,760 | 45,197 | 71,957 |
| L28 | 224 | 215 | 185 | 254 | 439 | 29,614 | 42,731 | 26,568 | 45,777 | 72,345 |
| L29 | 222 | 212 | 181 | 253 | 434 | 28,724 | 41,460 | 24,972 | 45,212 | 70,184 |
| L30 | 230 | 217 | 205 | 242 | 447 | 29,651 | 42,598 | 27,118 | 45,131 | 72,249 |
| L31 | 222 | 215 | 176 | 261 | 437 | 28,695 | 41,158 | 25,431 | 44,422 | 69,853 |
| L32 | 219 | 217 | 184 | 252 | 436 | 29,252 | 42,110 | 26,792 | 44,570 | 71,362 |
| L39 | 219 | 222 | 173 | 268 | 441 | 29,181 | 41,848 | 26,317 | 44,712 | 71,029 |
| L40 | 220 | 209 | 175 | 254 | 429 | 28,329 | 40,863 | 25,099 | 44,093 | 69,192 |
| L42 | 225 | 218 | 203 | 240 | 443 | 30,754 | 44,338 | 29,390 | 45,702 | 75,092 |
| L43 | 223 | 217 | 192 | 248 | 440 | 29,120 | 41,969 | 26,722 | 44,367 | 71,089 |
| L44 | 222 | 211 | 179 | 254 | 433 | 29,319 | 42,388 | 26,471 | 45,236 | 71,707 |
| L45 | 229 | 218 | 193 | 254 | 447 | 28,743 | 41,472 | 25,328 | 44,887 | 70,215 |
| L46 | 204 | 199 | 160 | 243 | 403 | 23,292 | 33,396 | 18,987 | 37,701 | 56,688 |
| L49 | 224 | 218 | 194 | 248 | 442 | 28,840 | 41,344 | 25,935 | 44,249 | 70,184 |
| L52 | 216 | 210 | 195 | 231 | 426 | 28,482 | 40,954 | 24,841 | 44,595 | 69,436 |
| L53 | 223 | 223 | 187 | 259 | 446 | 29,321 | 42,286 | 26,513 | 45,094 | 71,607 |
| L54 | 225 | 214 | 191 | 248 | 439 | 29,521 | 42,456 | 26,723 | 45,254 | 71,977 |
| L55 | 221 | 211 | 182 | 250 | 432 | 28,524 | 41,167 | 25,479 | 44,212 | 69,691 |
| L56 | 220 | 215 | 192 | 243 | 435 | 27,897 | 40,171 | 24,502 | 43,566 | 68,068 |
| L58 | 222 | 210 | 171 | 261 | 432 | 28,260 | 40,738 | 25,144 | 43,854 | 68,998 |
| L59 | 220 | 199 | 168 | 251 | 419 | 28,701 | 41,330 | 26,005 | 44,026 | 70,031 |
| L60 | 224 | 209 | 184 | 249 | 433 | 28,946 | 41,598 | 26,369 | 44,175 | 70,544 |
| L61 | 217 | 210 | 173 | 254 | 427 | 28,781 | 41,500 | 25,754 | 44,527 | 70,281 |
| L63 | 222 | 219 | 178 | 263 | 441 | 29,539 | 42,479 | 26,825 | 45,193 | 72,018 |
| L64 | 226 | 214 | 197 | 243 | 440 | 30,252 | 43,659 | 28,089 | 45,822 | 73,911 |
| L65 | 224 | 212 | 194 | 242 | 436 | 29,006 | 42,048 | 26,598 | 44,456 | 71,054 |
| L66 | 224 | 219 | 194 | 249 | 443 | 29,984 | 43,060 | 27,755 | 45,289 | 73,044 |
| L67 | 228 | 223 | 195 | 256 | 451 | 31,351 | 45,301 | 29,137 | 47,515 | 76,652 |
| L69 | 227 | 217 | 196 | 248 | 444 | 29,561 | 42,649 | 26,766 | 45,444 | 72,210 |
| L70 | 228 | 218 | 181 | 265 | 446 | 29,804 | 42,917 | 27,236 | 45,485 | 72,721 |
| L71 | 221 | 217 | 180 | 258 | 438 | 28,353 | 40,802 | 24,833 | 44,322 | 69,155 |
| L72 | 225 | 221 | 190 | 256 | 446 | 30,108 | 43,141 | 28,050 | 45,199 | 73,249 |
| L73 | 213 | 208 | 174 | 247 | 421 | 26,829 | 38,622 | 22,695 | 42,756 | 65,451 |
| L76 | 225 | 218 | 191 | 252 | 443 | 28,969 | 41,562 | 25,693 | 44,838 | 70,531 |
| L77 | 221 | 216 | 194 | 243 | 437 | 30,331 | 43,637 | 28,311 | 45,657 | 73,968 |
| L78 | 220 | 211 | 177 | 254 | 431 | 29,450 | 42,636 | 26,980 | 45,106 | 72,086 |
| L81 | 215 | 208 | 167 | 256 | 423 | 29,344 | 41,659 | 26,097 | 44,906 | 71,003 |
| L82 | 222 | 218 | 179 | 261 | 440 | 29,501 | 42,415 | 26,518 | 45,398 | 71,916 |
| L86 | 226 | 210 | 195 | 241 | 436 | 30,654 | 44,213 | 28,954 | 45,913 | 74,867 |
| L87 | 228 | 223 | 205 | 246 | 451 | 30,588 | 43,982 | 29,356 | 45,214 | 74,570 |
| L88 | 226 | 212 | 191 | 247 | 438 | 28,769 | 41,432 | 25,276 | 44,925 | 70,201 |
| L89 | 217 | 213 | 188 | 242 | 430 | 28,790 | 41,641 | 25,994 | 44,437 | 70,431 |
| L90 | 222 | 209 | 176 | 255 | 431 | 28,489 | 41,049 | 25,170 | 44,368 | 69,538 |
| L91 | 227 | 208 | 185 | 250 | 435 | 26,672 | 38,251 | 23,017 | 41,906 | 64,923 |
| L94 | 221 | 212 | 170 | 263 | 433 | 25,869 | 37,454 | 20,963 | 42,360 | 63,323 |
| L95 | 217 | 213 | 178 | 252 | 430 | 29,250 | 42,128 | 26,425 | 44,953 | 71,378 |
| L96 | 220 | 211 | 185 | 246 | 431 | 28,268 | 40,877 | 24,655 | 44,490 | 69,145 |
| L97 | 224 | 208 | 184 | 248 | 432 | 27,759 | 40,171 | 23,963 | 43,967 | 67,930 |
| L98 | 226 | 220 | 186 | 260 | 446 | 29,609 | 42,868 | 26,546 | 45,931 | 72,477 |
| L99 | 224 | 221 | 213 | 232 | 445 | 29,703 | 42,731 | 27,475 | 44,959 | 72,434 |
| L100 | 225 | 217 | 200 | 242 | 442 | 29,877 | 42,943 | 27,495 | 45,325 | 72,820 |
| L101 | 226 | 219 | 197 | 248 | 445 | 29,426 | 42,046 | 26,934 | 44,538 | 71,472 |
| L102 | 221 | 210 | 190 | 241 | 431 | 28,678 | 41,404 | 25,847 | 44,235 | 70,082 |
| L106 | 223 | 215 | 177 | 261 | 438 | 27,958 | 40,460 | 24,636 | 43,782 | 68,418 |
| L119 | 227 | 219 | 188 | 258 | 446 | 31,169 | 45,167 | 29,044 | 47,292 | 76,336 |
| L145 | 223 | 213 | 190 | 246 | 436 | 29,136 | 41,785 | 26,114 | 44,807 | 70,921 |
| L150 | 215 | 209 | 182 | 242 | 424 | 27,893 | 40,393 | 24,216 | 44,070 | 68,286 |
| L151 | 224 | 221 | 192 | 253 | 445 | 29,027 | 41,883 | 25,552 | 45,358 | 70,910 |
| L152 | 223 | 215 | 182 | 256 | 438 | 28,030 | 40,248 | 24,693 | 43,585 | 68,278 |
| L153 | 217 | 213 | 173 | 257 | 430 | 28,927 | 41,907 | 25,547 | 45,287 | 70,834 |
| L155 | 225 | 223 | 196 | 252 | 448 | 30,302 | 43,644 | 28,479 | 45,467 | 73,946 |
| L160 | 226 | 224 | 188 | 262 | 450 | 29,467 | 42,481 | 27,068 | 44,880 | 71,948 |
| L161 | 214 | 211 | 169 | 256 | 425 | 28,574 | 41,368 | 25,351 | 44,591 | 69,942 |
| L162 | 226 | 210 | 201 | 235 | 436 | 28,865 | 41,649 | 26,431 | 44,083 | 70,514 |
| L163 | 228 | 220 | 199 | 249 | 448 | 29,901 | 43,240 | 27,785 | 45,356 | 73,141 |
| L164 | 229 | 223 | 189 | 263 | 452 | 29,004 | 41,756 | 25,579 | 45,181 | 70,760 |
| L165 | 217 | 214 | 196 | 235 | 431 | 28,449 | 41,130 | 25,431 | 44,148 | 69,579 |
| L166 | 228 | 216 | 198 | 246 | 444 | 29,087 | 42,115 | 26,091 | 45,111 | 71,202 |
| L167 | 223 | 219 | 206 | 236 | 442 | 29,428 | 42,486 | 27,069 | 44,845 | 71,914 |
| L168 | 221 | 213 | 206 | 228 | 434 | 30,333 | 43,660 | 28,217 | 45,776 | 73,993 |
| L170 | 212 | 213 | 170 | 255 | 425 | 28,987 | 41,803 | 26,309 | 44,481 | 70,790 |
| L172 | 219 | 208 | 182 | 245 | 427 | 26,319 | 37,835 | 22,297 | 41,857 | 64,154 |
| L173 | 216 | 206 | 183 | 239 | 422 | 26,748 | 38,578 | 22,462 | 42,864 | 65,326 |
| L174 | 222 | 220 | 190 | 252 | 442 | 30,048 | 43,365 | 27,838 | 45,575 | 73,413 |
| L175 | 226 | 218 | 201 | 243 | 444 | 29,777 | 42,976 | 26,824 | 45,929 | 72,753 |
| L176 | 228 | 218 | 198 | 248 | 446 | 29,724 | 42,815 | 27,536 | 45,003 | 72,539 |
| L177 | 224 | 212 | 192 | 244 | 436 | 56,485 | 39,452 | 23,222 | 72,715 | 95,937 |
| L178 | 229 | 217 | 195 | 251 | 446 | 30,638 | 44,204 | 28,257 | 46,585 | 74,842 |
| L180 | 226 | 212 | 189 | 249 | 438 | 28,439 | 40,836 | 25,356 | 43,919 | 69,275 |
| L182 | 220 | 216 | 188 | 248 | 436 | 29,171 | 42,040 | 26,126 | 45,085 | 71,211 |
| L185 | 222 | 213 | 176 | 259 | 435 | 28,459 | 41,173 | 24,269 | 45,363 | 69,632 |
| L186 | 225 | 218 | 188 | 255 | 443 | 29,728 | 42,683 | 26,370 | 46,041 | 72,411 |
| L212 | 229 | 219 | 191 | 257 | 448 | 30,353 | 43,793 | 28,470 | 45,676 | 74,146 |
| L213 | 216 | 202 | 154 | 264 | 418 | 26,433 | 38,298 | 21,856 | 42,875 | 64,731 |
| L214 | 226 | 223 | 217 | 232 | 449 | 30,986 | 44,667 | 29,758 | 45,895 | 75,653 |
| L217 | 216 | 209 | 185 | 240 | 425 | 28,105 | 40,457 | 24,888 | 43,674 | 68,562 |
| L219 | 219 | 210 | 175 | 254 | 429 | 27,331 | 39,100 | 23,166 | 43,265 | 66,431 |
| L220 | 228 | 224 | 195 | 257 | 452 | 30,759 | 44,415 | 29,214 | 45,960 | 75,174 |
| L222 | 224 | 214 | 187 | 251 | 438 | 29,550 | 42,691 | 26,953 | 45,288 | 72,241 |
| L226 | 221 | 198 | 162 | 257 | 419 | 25,629 | 36,847 | 20,710 | 41,766 | 62,476 |
| L227 | 215 | 208 | 155 | 268 | 423 | 27,716 | 40,063 | 23,487 | 44,292 | 67,779 |
| L228 | 219 | 216 | 192 | 243 | 435 | 29,220 | 42,036 | 26,514 | 44,742 | 71,256 |
| L229 | 217 | 212 | 170 | 259 | 429 | 28,006 | 40,285 | 24,323 | 43,968 | 68,291 |
| L232 | 219 | 213 | 169 | 263 | 432 | 28,672 | 41,306 | 25,547 | 44,431 | 69,978 |
| L233 | 226 | 226 | 195 | 257 | 452 | 30,667 | 44,059 | 29,308 | 45,418 | 74,726 |
| L252 | 219 | 221 | 172 | 268 | 440 | 29,013 | 41,705 | 26,550 | 44,168 | 70,718 |
| L297 | 219 | 203 | 170 | 252 | 422 | 25,971 | 37,594 | 21,554 | 42,011 | 63,565 |
| L298 | 217 | 213 | 168 | 262 | 430 | 28,329 | 41,164 | 25,027 | 44,466 | 69,493 |
| L299 | 225 | 215 | 207 | 233 | 440 | 30,048 | 43,110 | 27,934 | 45,224 | 73,158 |
| L501 | 225 | 221 | 206 | 240 | 446 | 30,659 | 44,373 | 29,558 | 45,474 | 75,032 |
| L504 | 221 | 220 | 185 | 256 | 441 | 29,443 | 42,415 | 26,827 | 45,031 | 71,858 |
| L530 | 224 | 210 | 186 | 248 | 434 | 28,982 | 42,203 | 25,850 | 45,335 | 71,185 |
| L531 | 229 | 217 | 215 | 231 | 446 | 30,439 | 43,569 | 28,460 | 45,548 | 74,008 |
| L534 | 215 | 210 | 178 | 247 | 425 | 27,991 | 40,259 | 24,059 | 44,191 | 68,250 |
| L536 | 220 | 214 | 188 | 246 | 434 | 29,848 | 43,153 | 27,490 | 45,511 | 73,001 |
| L539 | 225 | 216 | 178 | 263 | 441 | 28,645 | 41,255 | 25,346 | 44,554 | 69,900 |
| L540 | 228 | 215 | 202 | 241 | 443 | 31,050 | 44,872 | 29,909 | 46,013 | 75,922 |
| L543 | 226 | 216 | 178 | 264 | 442 | 29,542 | 42,649 | 26,007 | 46,184 | 72,191 |
| L545 | 223 | 219 | 210 | 232 | 442 | 29,264 | 42,092 | 26,709 | 44,647 | 71,356 |
| L547 | 216 | 203 | 177 | 242 | 419 | 25,660 | 37,245 | 21,045 | 41,860 | 62,905 |
| L548 | 217 | 213 | 196 | 234 | 430 | 29,514 | 42,538 | 26,269 | 45,783 | 72,052 |
| L559 | 223 | 214 | 201 | 236 | 437 | 30,017 | 43,324 | 27,857 | 45,484 | 73,341 |
| L561 | 224 | 212 | 171 | 265 | 436 | 28,169 | 40,638 | 24,858 | 43,949 | 68,807 |
| L564 | 219 | 210 | 163 | 266 | 429 | 27,731 | 40,095 | 22,541 | 45,285 | 67,826 |
| L3001 | 221 | 212 | 196 | 237 | 433 | 28,316 | 40,870 | 25,764 | 43,422 | 69,186 |
| L3003 | 219 | 218 | 177 | 260 | 437 | 29,106 | 42,084 | 26,208 | 44,982 | 71,190 |
| L3007 | 217 | 209 | 179 | 247 | 426 | 27,655 | 39,925 | 23,579 | 44,001 | 67,580 |
| L3009 | 229 | 221 | 202 | 248 | 450 | 31,814 | 45,878 | 31,881 | 45,811 | 77,692 |
| L3011 | 223 | 213 | 183 | 253 | 436 | 28,483 | 40,798 | 24,962 | 44,319 | 69,281 |
| L3012 | 220 | 207 | 167 | 260 | 427 | 27,390 | 39,709 | 23,607 | 43,492 | 67,099 |
| L3014 | 212 | 211 | 173 | 250 | 423 | 27,689 | 40,194 | 24,099 | 43,784 | 67,883 |
| L3038 | 231 | 215 | 200 | 246 | 446 | 31,696 | 45,686 | 31,585 | 45,797 | 77,382 |
| L164-1 | 224 | 220 | 177 | 267 | 444 | 28,573 | 41,161 | 24,432 | 45,302 | 69,734 |
| L213-1 | 215 | 210 | 168 | 257 | 425 | 27,975 | 40,370 | 24,382 | 43,963 | 68,345 |
| LbendiCK | 219 | 213 | 179 | 253 | 432 | 29,567 | 42,716 | 26,088 | 46,195 | 72,283 |
| LDe1 | 230 | 221 | 180 | 271 | 451 | 30,535 | 44,033 | 29,265 | 45,303 | 74,568 |
| LFy1 | 230 | 220 | 198 | 252 | 450 | 30,375 | 43,715 | 28,978 | 45,112 | 74,090 |
| LFy2 | 222 | 211 | 192 | 241 | 433 | 29,114 | 41,775 | 26,712 | 44,177 | 70,889 |
| LFy5 | 222 | 205 | 165 | 262 | 427 | 27,463 | 39,426 | 23,762 | 43,127 | 66,889 |
| LFy7 | 222 | 217 | 182 | 257 | 439 | 28,375 | 40,744 | 24,897 | 44,222 | 69,119 |
| LFy9 | 218 | 213 | 176 | 255 | 431 | 27,873 | 40,315 | 23,916 | 44,272 | 68,188 |
| Lgan1 | 225 | 217 | 175 | 267 | 442 | 30,120 | 43,385 | 28,369 | 45,136 | 73,505 |
| Lgan12 | 225 | 219 | 204 | 240 | 444 | 30,035 | 43,647 | 28,752 | 44,930 | 73,682 |
| Lgan2 | 229 | 216 | 212 | 233 | 445 | 30,241 | 43,690 | 28,697 | 45,234 | 73,931 |
| Lgan3 | 226 | 219 | 202 | 243 | 445 | 30,309 | 43,571 | 28,599 | 45,281 | 73,880 |
| Lgan4 | 229 | 222 | 192 | 259 | 451 | 30,271 | 43,549 | 28,286 | 45,534 | 73,820 |
| Lgan5 | 228 | 216 | 182 | 262 | 444 | 29,998 | 43,122 | 28,154 | 44,966 | 73,120 |
| Lgan9 | 222 | 214 | 178 | 258 | 436 | 28,452 | 41,087 | 24,988 | 44,551 | 69,539 |
| Lganwu2 | 227 | 223 | 197 | 253 | 450 | 29,914 | 43,064 | 27,278 | 45,700 | 72,978 |
| Lgui1 | 224 | 218 | 190 | 252 | 442 | 29,514 | 42,533 | 26,836 | 45,211 | 72,047 |
| Lgui2 | 226 | 225 | 202 | 249 | 451 | 29,398 | 42,221 | 26,619 | 45,000 | 71,619 |
| Lgui3 | 226 | 215 | 193 | 248 | 441 | 28,932 | 41,614 | 26,202 | 44,344 | 70,546 |
| Lgui4 | 221 | 211 | 174 | 258 | 432 | 28,886 | 41,696 | 25,444 | 45,138 | 70,582 |
| Lguiruan2 | 229 | 216 | 200 | 245 | 445 | 29,755 | 41,281 | 26,236 | 44,800 | 71,036 |
| Lguiruan23 | 226 | 214 | 173 | 267 | 440 | 28,926 | 41,743 | 24,285 | 46,384 | 70,669 |
| Lguiruan3 | 220 | 217 | 201 | 236 | 437 | 28,938 | 41,558 | 25,538 | 44,958 | 70,496 |
| Ljiangxi23 | 221 | 207 | 205 | 223 | 428 | 28,885 | 41,893 | 26,362 | 44,416 | 70,778 |
| Lmin43 | 227 | 221 | 224 | 224 | 448 | 30,316 | 40,514 | 28,778 | 42,052 | 70,830 |
| Lmin48 | 227 | 221 | 196 | 252 | 448 | 31,057 | 44,755 | 29,769 | 46,043 | 75,812 |
| Lmin60 | 222 | 222 | 203 | 241 | 444 | 29,924 | 42,931 | 28,307 | 44,548 | 72,855 |
| Lminyou1 | 229 | 216 | 191 | 254 | 445 | 29,777 | 42,777 | 27,742 | 44,812 | 72,554 |
| Lminyou10 | 227 | 224 | 205 | 246 | 451 | 29,521 | 42,343 | 27,512 | 44,352 | 71,864 |
| Lminyou11 | 227 | 222 | 198 | 251 | 449 | 30,417 | 43,713 | 28,486 | 45,644 | 74,130 |
| Lminyou2 | 225 | 220 | 199 | 246 | 445 | 31,175 | 44,800 | 29,889 | 46,086 | 75,975 |
| Lminyou4 | 224 | 217 | 198 | 243 | 441 | 30,177 | 43,531 | 28,458 | 45,250 | 73,708 |
| Lminyou43 | 225 | 223 | 196 | 252 | 448 | 30,875 | 44,446 | 29,444 | 45,877 | 75,321 |
| Lminyou48 | 224 | 218 | 205 | 237 | 442 | 30,164 | 43,438 | 28,132 | 45,470 | 73,602 |
| Lminyou5 | 221 | 221 | 189 | 253 | 442 | 29,673 | 42,869 | 27,387 | 45,155 | 72,542 |
| Lminyou6 | 226 | 226 | 190 | 262 | 452 | 31,234 | 45,038 | 30,251 | 46,021 | 76,272 |
| Lminyou60 | 225 | 217 | 202 | 240 | 442 | 30,496 | 43,995 | 29,049 | 45,442 | 74,491 |
| Lminyou8 | 225 | 215 | 207 | 233 | 440 | 29,778 | 42,791 | 27,289 | 45,280 | 72,569 |
| Lminyou9 | 223 | 222 | 192 | 253 | 445 | 31,151 | 44,846 | 30,148 | 45,849 | 75,997 |
| Ltong10 | 215 | 207 | 177 | 245 | 422 | 29,348 | 42,561 | 26,573 | 45,336 | 71,909 |
| Ltong11 | 219 | 200 | 171 | 248 | 419 | 24,630 | 35,676 | 20,507 | 39,799 | 60,306 |
| Ltong12 | 227 | 219 | 190 | 256 | 446 | 30,120 | 43,225 | 27,940 | 45,405 | 73,345 |
| Ltong18 | 219 | 213 | 170 | 262 | 432 | 27,549 | 39,654 | 24,185 | 43,018 | 67,203 |
| Ltong29 | 229 | 222 | 196 | 255 | 451 | 29,724 | 43,002 | 28,310 | 44,416 | 72,726 |
| Ltong3 | 218 | 210 | 190 | 238 | 428 | 27,638 | 39,800 | 24,478 | 42,960 | 67,438 |
| Ltong30 | 226 | 218 | 188 | 256 | 444 | 30,071 | 43,270 | 28,321 | 45,020 | 73,341 |
| Ltong32 | 228 | 221 | 199 | 250 | 449 | 29,625 | 42,576 | 26,749 | 45,452 | 72,201 |
| Ltong38 | 224 | 212 | 200 | 236 | 436 | 30,115 | 43,343 | 28,402 | 45,056 | 73,458 |
| Ltong39 | 224 | 212 | 195 | 241 | 436 | 29,754 | 42,731 | 27,699 | 44,786 | 72,485 |
| Ltong4 | 221 | 207 | 176 | 252 | 428 | 28,743 | 41,355 | 25,847 | 44,251 | 70,098 |
| Ltong40 | 227 | 214 | 186 | 255 | 441 | 29,352 | 42,288 | 26,930 | 44,710 | 71,640 |
| Ltong41 | 229 | 221 | 191 | 259 | 450 | 29,957 | 43,218 | 27,560 | 45,615 | 73,175 |
| Ltong6 | 218 | 204 | 165 | 257 | 422 | 26,974 | 38,883 | 23,014 | 42,843 | 65,857 |
| Ltong9 | 224 | 220 | 193 | 251 | 444 | 29,864 | 42,999 | 28,204 | 44,659 | 72,863 |
| Lwu | 223 | 217 | 192 | 248 | 440 | 29,354 | 42,409 | 26,556 | 45,207 | 71,763 |
| Lwu-1 | 231 | 221 | 204 | 248 | 452 | 29,675 | 42,632 | 27,804 | 44,503 | 72,307 |
| Lwu5-1 | 229 | 217 | 191 | 255 | 446 | 30,228 | 43,501 | 28,537 | 45,192 | 73,729 |
| Lwu5-2 | 220 | 209 | 184 | 245 | 429 | 28,600 | 41,287 | 25,109 | 44,778 | 69,887 |
| Lwupai5 | 227 | 220 | 199 | 248 | 447 | 29,997 | 43,218 | 28,235 | 44,980 | 73,215 |
| Lwuyiwu2 | 227 | 214 | 182 | 259 | 441 | 29,818 | 43,093 | 27,429 | 45,482 | 72,911 |
| Lxiang3 | 228 | 220 | 198 | 250 | 448 | 30,167 | 43,391 | 28,451 | 45,107 | 73,558 |
| Lxiang4 | 224 | 215 | 189 | 250 | 439 | 28,422 | 40,807 | 24,943 | 44,286 | 69,229 |
| Lxiang47 | 227 | 222 | 182 | 267 | 449 | 29,175 | 42,037 | 26,269 | 44,943 | 71,212 |
| Lxiang5 | 228 | 220 | 193 | 255 | 448 | 30,256 | 43,592 | 28,727 | 45,121 | 73,848 |
| Lxiang50 | 225 | 222 | 212 | 235 | 447 | 30,190 | 43,368 | 28,382 | 45,176 | 73,558 |
| Lxiang6 | 224 | 215 | 197 | 242 | 439 | 30,901 | 44,426 | 28,820 | 46,507 | 75,327 |
| Lxiang7 | 231 | 222 | 187 | 266 | 453 | 30,590 | 43,936 | 28,783 | 45,743 | 74,526 |
| Lxiang8 | 224 | 212 | 185 | 251 | 436 | 28,883 | 41,359 | 25,577 | 44,665 | 70,242 |
| Lxiang9 | 221 | 217 | 190 | 248 | 438 | 29,846 | 42,829 | 27,470 | 45,205 | 72,675 |
| Lyajia1 | 219 | 208 | 165 | 262 | 427 | 26,276 | 37,854 | 22,534 | 41,596 | 64,130 |
| Lyajia2 | 229 | 215 | 197 | 247 | 444 | 29,525 | 42,449 | 27,331 | 44,643 | 71,974 |
| Lyajia6 | 223 | 215 | 183 | 255 | 438 | 29,811 | 42,903 | 27,460 | 45,254 | 72,714 |
| Lyajia8 | 224 | 211 | 196 | 239 | 435 | 28,593 | 40,965 | 25,958 | 43,600 | 69,558 |
| Lyawu1 | 226 | 215 | 201 | 240 | 441 | 30,139 | 43,205 | 27,982 | 45,362 | 73,344 |
| Lyawu2 | 230 | 221 | 211 | 240 | 451 | 30,285 | 43,366 | 28,394 | 45,257 | 73,651 |
| Lyawu4 | 228 | 222 | 206 | 244 | 450 | 30,441 | 43,850 | 29,551 | 44,740 | 74,291 |
| Lyawu6 | 221 | 212 | 196 | 237 | 433 | 29,853 | 43,124 | 27,162 | 45,815 | 72,977 |
| Lyawu7 | 225 | 220 | 199 | 246 | 445 | 28,960 | 41,585 | 27,108 | 43,437 | 70,545 |
| all | 233 | 229 | / | / | 462 | 35,086 | 50,354 | / | / | 85,440 |

Table S15. Statistics of InDels of *C. oleifera* association population in this study

| Sample | yield (kg) | Sample | yield (kg) | Sample | yield (kg) | Sample | yield (kg) | Sample | yield (kg) |
| --- | --- | --- | --- | --- | --- | --- | --- | --- | --- |
| L1 | 4.69 | L182 | 3.79 | L3009 | 4.15 | L60 | 5.06 | Lgan1 | 3.22 |
| L10 | 4.39 | L185 | 6.32 | L3011 | 2.82 | L61 | 9.23 | Lgan12 | 4.99 |
| L100 | 8.87 | L186 | 6.85 | L3012 | 3.52 | L63 | 5.52 | Lgan3 | 3.44 |
| L101 | 2.89 | L19 | 1.56 | L3014 | 3.52 | L64 | 4.32 | Lgan4 | 3.25 |
| L102 | 5.07 | L2 | 5.46 | L3038 | 9.83 | L65 | 3.79 | Lgan5 | 3.65 |
| L106 | 8.05 | L20 | 6.28 | L31 | 5.97 | L66 | 4.32 | Lgan9 | 2.59 |
| L11 | 2.95 | L21 | 4.82 | L32 | 3.66 | L67 | 2.93 | Lguiruan2 | 1.68 |
| L119 | 2.71 | L212 | 2.31 | L39 | 5.29 | L69 | 5.14 | Lguiruan23 | 2.87 |
| L12 | 4.58 | L213 | 5.14 | L4 | 6.07 | L70 | 5.52 | Lguiruan3 | 1.62 |
| L145 | 3.17 | L213-1 | 5.18 | L40 | 7.36 | L71 | 5.47 | Ljiangxi23 | 2.40 |
| L15 | 5.89 | L214 | 7.82 | L42 | 5.79 | L72 | 5.34 | Lmin43 | 1.93 |
| L150 | 5.42 | L217 | 2.16 | L43 | 6.04 | L73 | 7.57 | Lmin48 | 2.05 |
| L151 | 7.23 | L219 | 6.57 | L44 | 2.79 | L76 | 3.60 | Lmin60 | 0.85 |
| L152 | 3.87 | L22 | 4.04 | L45 | 8.40 | L77 | 4.34 | Ltong10 | 4.11 |
| L153 | 3.43 | L220 | 6.74 | L46 | 6.52 | L78 | 4.53 | Ltong11 | 2.99 |
| L155 | 3.87 | L222 | 2.27 | L49 | 6.17 | L8 | 4.20 | Ltong12 | 2.23 |
| L16 | 4.29 | L226 | 4.45 | L501 | 5.76 | L81 | 3.96 | Ltong18 | 3.17 |
| L160 | 1.95 | L227 | 0.36 | L52 | 7.09 | L82 | 4.36 | Ltong29 | 2.21 |
| L161 | 4.06 | L228 | 2.18 | L53 | 4.84 | L86 | 8.07 | Ltong3 | 2.86 |
| L162 | 2.36 | L229 | 1.67 | L530 | 4.69 | L87 | 6.75 | Ltong30 | 2.87 |
| L163 | 5.16 | L23 | 6.37 | L531 | 6.18 | L88 | 5.20 | Ltong32 | 7.70 |
| L164 | 8.74 | L232 | 5.38 | L534 | 5.05 | L89 | 5.39 | Ltong38 | 4.58 |
| L164-1 | 5.85 | L233 | 5.65 | L536 | 5.02 | L9 | 5.05 | Ltong39 | 4.28 |
| L165 | 5.36 | L24 | 4.77 | L539 | 3.31 | L90 | 3.28 | Ltong4 | 2.11 |
| L166 | 4.08 | L252 | 3.49 | L54 | 4.19 | L91 | 6.59 | Ltong40 | 1.04 |
| L167 | 5.08 | L26 | 2.86 | L540 | 2.26 | L94 | 5.32 | Ltong41 | 4.14 |
| L168 | 5.87 | L27 | 2.79 | L543 | 3.56 | L95 | 5.61 | Ltong6 | 3.45 |
| L17 | 4.30 | L28 | 3.96 | L545 | 1.87 | L96 | 4.88 | Ltong9 | 3.44 |
| L170 | 5.82 | L29 | 1.83 | L547 | 1.20 | L97 | 6.25 | Lwu | 6.75 |
| L172 | 8.84 | L297 | 5.39 | L548 | 3.12 | L98 | 5.07 | Lwu-1 | 5.07 |
| L173 | 4.86 | L298 | 7.10 | L55 | 7.71 | L99 | 6.96 | Lxiang3 | 4.04 |
| L174 | 3.65 | L299 | 4.75 | L559 | 4.58 | LDe1 | 1.93 | Lxiang4 | 3.21 |
| L175 | 6.74 | L3 | 6.79 | L56 | 5.31 | LFy1 | 2.89 | Lxiang47 | 3.53 |
| L176 | 4.47 | L30 | 7.26 | L561 | 3.86 | LFy2 | 4.04 | Lxiang5 | 1.77 |
| L177 | 8.31 | L3001 | 3.18 | L564 | 3.24 | LFy5 | 1.48 | Lxiang50 | 3.76 |
| L178 | 5.88 | L3003 | 2.22 | L58 | 5.49 | LFy7 | 2.72 | Lxiang6 | 2.18 |
| L18 | 3.03 | L3007 | 2.11 | L59 | 2.15 | LFy9 | 1.88 | Lxiang8 | 3.67 |
| L180 | 2.78 |  |  |  |  |  |  |  |  |

Table S16-1

| Sample | fruit weight (g) | seed number per fruit | Sample | fruit weight (g) | seed number per fruit | Sample | fruit weight (g) | seed number per fruit | Sample | fruit weight (g) | seed number per fruit |
| --- | --- | --- | --- | --- | --- | --- | --- | --- | --- | --- | --- |
| L100 | 17.92 | 4.27 | L186 | 16.68 | 1.73 | L49 | 44.23 | 5.30 | L89 | 22.23 | 5.17 |
| L102 | 18.45 | 4.37 | L20 | 20.86 | 5.52 | L52 | 16.27 | 1.98 | L9 | 16.95 | 4.33 |
| L106 | 20.20 | 4.10 | L21 | 18.75 | 4.42 | L53 | 18.72 | 3.51 | L90 | 15.86 | 4.82 |
| L11 | 19.89 | 3.86 | L212 | 12.86 | 3.56 | L54 | 17.72 | 2.25 | L91 | 23.68 | 4.59 |
| L12 | 19.35 | 3.91 | L213 | 19.75 | 2.63 | L55 | 12.21 | 4.98 | L94 | 16.47 | 3.59 |
| L145 | 21.42 | 2.97 | L213-1 | 14.84 | 2.30 | L56 | 18.93 | 4.41 | L95 | 24.94 | 3.20 |
| L15 | 26.62 | 3.86 | L214 | 18.49 | 3.33 | L58 | 16.13 | 4.60 | L96 | 17.32 | 4.92 |
| L150 | 17.61 | 1.85 | L217 | 11.29 | 2.90 | L59 | 13.02 | 2.48 | L97 | 13.12 | 3.23 |
| L151 | 19.53 | 5.27 | L22 | 20.89 | 5.85 | L60 | 8.38 | 1.58 | L98 | 24.50 | 5.47 |
| L152 | 7.73 | 2.12 | L220 | 16.66 | 2.41 | L61 | 15.38 | 2.94 | L99 | 28.19 | 5.12 |
| L153 | 9.46 | 3.48 | L226 | 16.33 | 2.51 | L63 | 14.16 | 2.57 | LDe1 | 34.37 | 7.25 |
| L155 | 46.86 | 9.14 | L228 | 17.18 | 2.24 | L64 | 21.74 | 3.03 | Lguiruan2 | 23.21 | 5.13 |
| L16 | 17.58 | 3.67 | L23 | 18.51 | 4.01 | L65 | 13.84 | 5.21 | Lguiruan23 | 20.43 | 4.20 |
| L161 | 16.95 | 1.99 | L232 | 27.35 | 4.11 | L66 | 23.61 | 3.80 | Lguiruan3 | 26.71 | 4.25 |
| L163 | 15.82 | 2.62 | L233 | 29.02 | 3.15 | L67 | 27.29 | 2.96 | Ljiangxi23 | 16.54 | 3.90 |
| L164 | 17.68 | 3.17 | L24 | 21.24 | 3.40 | L69 | 20.81 | 2.68 | Lmin48 | 24.34 | 4.83 |
| L164-1 | 13.89 | 3.14 | L26 | 20.40 | 2.60 | L70 | 16.74 | 3.25 | Lmin60 | 22.42 | 4.02 |
| L165 | 14.38 | 1.99 | L27 | 18.23 | 2.92 | L71 | 18.21 | 1.98 | Lminyou43 | 16.66 | 2.93 |
| L166 | 13.00 | 2.23 | L29 | 22.20 | 3.38 | L72 | 12.02 | 3.33 | Ltong30 | 17.39 | 4.55 |
| L167 | 16.40 | 2.85 | L297 | 7.80 | 2.31 | L73 | 12.84 | 2.02 | Ltong41 | 11.29 | 2.19 |
| L168 | 13.47 | 2.81 | L3 | 11.15 | 3.71 | L76 | 16.65 | 3.91 | Ltong9 | 17.75 | 3.28 |
| L17 | 16.12 | 3.38 | L30 | 23.90 | 3.69 | L77 | 28.22 | 2.88 | Lwu | 22.18 | 5.95 |
| L172 | 18.28 | 3.66 | L31 | 27.83 | 4.01 | L78 | 14.25 | 3.22 | Lxiang3 | 25.41 | 5.40 |
| L173 | 17.54 | 3.97 | L39 | 24.52 | 3.86 | L8 | 24.86 | 4.25 | Lxiang4 | 17.76 | 3.46 |
| L175 | 14.01 | 3.16 | L4 | 19.52 | 4.00 | L81 | 14.08 | 2.20 | Lxiang47 | 29.98 | 5.57 |
| L177 | 19.95 | 2.72 | L40 | 13.23 | 3.32 | L82 | 33.44 | 3.74 | Lxiang5 | 20.93 | 4.17 |
| L178 | 21.48 | 4.42 | L42 | 19.72 | 3.26 | L86 | 10.93 | 3.02 | Lxiang50 | 31.10 | 5.70 |
| L180 | 20.63 | 3.96 | L43 | 24.08 | 2.74 | L87 | 34.95 | 6.43 | Lxiang6 | 21.27 | 4.93 |
| L182 | 16.31 | 2.28 | L45 | 26.17 | 2.23 | L88 | 12.43 | 1.65 | Lxiang8 | 28.39 | 4.43 |
| L185 | 21.67 | 4.44 | L46 | 12.95 | 2.52 |  |  |  |  |  |  |

Table S16-2

Table S16. Mean of fruit traits of *C. oleifera* association population

Table S16-1 mean of fruit yield (kg) per tree, Table S16-2 mean fruit weight (g) and seed number of per fruit in the *C. oleifera* association population.

| GO term | p-value | FDR | GO annotation |
| --- | --- | --- | --- |
| GO:0008553 | 1.63E-13 | 3.76E-12 | hydrogen-exporting ATPase activity, phosphorylative mechanism |
| GO:0006754 | 1.63E-13 | 1.88E-12 | ATP biosynthetic process |
| GO:0051453 | 9.25E-11 | 7.10E-10 | regulation of intracellular pH |
| GO:0030686 | 2.16E-10 | 1.24E-09 | 90S preribosome |
| GO:0000462 | 1.02E-08 | 4.70E-08 | maturation of SSU-rRNA from tricistronic rRNA transcript (SSU-rRNA, 5.8S rRNA, LSU-rRNA) |
| GO:1902600 | 6.59E-08 | 2.53E-07 | hydrogen ion transmembrane transport |
| GO:0052739 | 1.11E-07 | 3.65E-07 | phosphatidylserine 1-acylhydrolase activity |
| GO:0052740 | 1.11E-07 | 3.20E-07 | 1-acyl-2-lysophosphatidylserine acylhydrolase activity |
| GO:0008970 | 2.46E-07 | 6.28E-07 | phosphatidylcholine 1-acylhydrolase activity |
| GO:0032040 | 3.27E-07 | 7.51E-07 | small-subunit processome |
| GO:0031683 | 1.65E-06 | 3.46E-06 | G-protein beta gamma-subunit complex binding |
| GO:0007186 | 7.48E-06 | 1.43E-05 | G-protein coupled receptor signaling pathway |
| GO:0005887 | 8.44E-06 | 1.49E-05 | integral component of plasma membrane |
| GO:0005730 | 3.74E-05 | 6.15E-05 | nucleolus |
| GO:0008198 | 6.00E-05 | 9.20E-05 | ferrous iron binding |
| GO:0004871 | 9.51E-05 | 0.000137 | signal transducer activity |
| GO:0006396 | 0.000152 | 0.000206 | RNA processing |
| GO:0017145 | 0.000202 | 0.000258 | stem cell division |
| GO:0097428 | 0.000202 | 0.000245 | protein maturation by iron-sulfur cluster transfer |
| GO:2000069 | 0.000202 | 0.000233 | regulation of post-embryonic root development |
| GO:0043231 | 0.00028 | 0.000306 | intracellular membrane-bounded organelle |
| GO:0030674 | 0.000318 | 0.000332 | protein binding, bridging |
| GO:0005198 | 0.000339 | 0.000339 | structural molecule activity |
| GO:0010364 | 0.000656 | 0.000629 | regulation of ethylene biosynthetic process |
| GO:0006810 | 0.000667 | 0.000614 | transport |
| GO:0005338 | 0.000997 | 0.000882 | nucleotide-sugar transmembrane transporter activity |
| GO:0015780 | 0.001958 | 0.001668 | nucleotide-sugar transport |
| GO:0045053 | 0.001958 | 0.001608 | protein retention in Golgi apparatus |
| GO:1901679 | 0.001958 | 0.001553 | nucleotide transmembrane transport |
| GO:0000814 | 0.001958 | 0.001501 | ESCRT II complex |
| GO:0042285 | 0.001958 | 0.001453 | xylosyltransferase activity |
| GO:0043328 | 0.001958 | 0.001407 | protein targeting to vacuole involved in ubiquitin-dependent protein catabolic process via the multivesicular body sorting pathway |
| GO:0005643 | 0.002181 | 0.00152 | nuclear pore |
| GO:0016709 | 0.002678 | 0.001812 | oxidoreductase activity, acting on paired donors, with incorporation or reduction of molecular oxygen, NAD(P)H as one donor, and incorporation of one atom of oxygen |
| GO:0043161 | 0.002892 | 0.001901 | proteasome-mediated ubiquitin-dependent protein catabolic process |
| GO:0007030 | 0.003203 | 0.002046 | Golgi organization |
| GO:0016226 | 0.003203 | 0.001991 | iron-sulfur cluster assembly |
| GO:0044318 | 0.003203 | 0.001939 | L-aspartate:fumarate oxidoreductase activity |
| GO:0007059 | 0.003203 | 0.001889 | chromosome segregation |
| GO:0008734 | 0.003203 | 0.001842 | L-aspartate oxidase activity |
| GO:0005198 | 0.00337 | 0.001891 | structural molecule activity |
| GO:0008541 | 0.004716 | 0.002583 | proteasome regulatory particle, lid subcomplex |
| GO:0010016 | 0.004716 | 0.002523 | shoot system morphogenesis |
| GO:0010417 | 0.004716 | 0.002465 | glucuronoxylan biosynthetic process |
| GO:0009815 | 0.004716 | 0.002411 | 1-aminocyclopropane-1-carboxylate oxidase activity |
| GO:0097159 | 0.005862 | 0.002932 | organic cyclic compound binding |
| GO:0016036 | 0.005929 | 0.002902 | cellular response to phosphate starvation |
| GO:0006075 | 0.005929 | 0.002841 | (1->3)-beta-D-glucan biosynthetic process |
| GO:0006629 | 0.006147 | 0.002886 | lipid metabolic process |
| GO:0051537 | 0.006328 | 0.002911 | 2 iron, 2 sulfur cluster binding |
| GO:0006730 | 0.006481 | 0.002923 | one-carbon metabolic process |
| GO:0009933 | 0.006481 | 0.002867 | meristem structural organization |
| GO:0006556 | 0.006481 | 0.002813 | S-adenosylmethionine biosynthetic process |
| GO:0046872 | 0.006505 | 0.002721 | metal ion binding |
| GO:0000145 | 0.006739 | 0.002768 | exocyst |
| GO:0009506 | 0.006976 | 0.002815 | plasmodesma |
| GO:0046029 | 0.008482 | 0.003364 | mannitol dehydrogenase activity |
| GO:0051601 | 0.008482 | 0.003307 | exocyst localization |
| GO:0009723 | 0.008482 | 0.003252 | response to ethylene |
| GO:0010114 | 0.008482 | 0.003199 | response to red light |
| GO:0000148 | 0.008534 | 0.003166 | 1,3-beta-D-glucan synthase complex |
| GO:0003843 | 0.008534 | 0.003116 | 1,3-beta-D-glucan synthase activity |
| GO:0016226 | 0.00952 | 0.003422 | iron-sulfur cluster assembly |
| GO:0006633 | 0.009905 | 0.003505 | fatty acid biosynthetic process |
| GO:0017119 | 0.010705 | 0.003731 | Golgi transport complex |
| GO:0031463 | 0.010705 | 0.003675 | Cul3-RING ubiquitin ligase complex |
| GO:0090447 | 0.010705 | 0.003621 | glycerol-3-phosphate 2-O-acyltransferase activity |
| GO:0004478 | 0.010705 | 0.003569 | methionine adenosyltransferase activity |
| GO:0016301 | 0.011481 | 0.003773 | kinase activity |
| GO:0016049 | 0.011666 | 0.00378 | cell growth |
| GO:0003676 | 0.012524 | 0.004001 | nucleic acid binding |
| GO:0004743 | 0.013135 | 0.004139 | pyruvate kinase activity |
| GO:0030955 | 0.013135 | 0.004083 | potassium ion binding |
| GO:0009435 | 0.013135 | 0.004029 | NAD biosynthetic process |
| GO:0010143 | 0.013135 | 0.003976 | cutin biosynthetic process |
| GO:0004004 | 0.014043 | 0.004195 | ATP-dependent RNA helicase activity |
| GO:0016021 | 0.014482 | 0.004271 | integral component of membrane |
| GO:0005886 | 0.015091 | 0.004394 | plasma membrane |
| GO:0004497 | 0.015759 | 0.004531 | monooxygenase activity |
| GO:0006355 | 0.017657 | 0.005015 | regulation of transcription, DNA-templated |
| GO:0004822 | 0.018407 | 0.005164 | isoleucine-tRNA ligase activity |
| GO:0006893 | 0.018407 | 0.005102 | Golgi to plasma membrane transport |
| GO:0010817 | 0.018407 | 0.005041 | regulation of hormone levels |
| GO:0017049 | 0.018407 | 0.004982 | GTP-Rho binding |
| GO:0030334 | 0.018407 | 0.004924 | regulation of cell migration |
| GO:0031407 | 0.018407 | 0.004867 | oxylipin metabolic process |
| GO:0032041 | 0.018407 | 0.004812 | NAD-dependent histone deacetylase activity (H3-K14 specific) |
| GO:0033721 | 0.018407 | 0.004758 | aldehyde dehydrogenase (NADP+) activity |
| GO:0043021 | 0.018407 | 0.004705 | ribonucleoprotein complex binding |
| GO:0043682 | 0.018407 | 0.004653 | copper-transporting ATPase activity |
| GO:0046520 | 0.018407 | 0.004603 | sphingoid biosynthetic process |
| GO:0046976 | 0.018407 | 0.004553 | histone methyltransferase activity (H3-K27 specific) |
| GO:0048017 | 0.018407 | 0.004505 | inositol lipid-mediated signaling |
| GO:0050764 | 0.018407 | 0.004457 | regulation of phagocytosis |
| GO:0060321 | 0.018407 | 0.004411 | acceptance of pollen |
| GO:0070734 | 0.018407 | 0.004365 | histone H3-K27 methylation |
| GO:0070932 | 0.018407 | 0.004321 | histone H3 deacetylation |
| GO:0071139 | 0.018407 | 0.004277 | resolution of recombination intermediates |
| GO:0071277 | 0.018407 | 0.004234 | cellular response to calcium ion |
| GO:1903600 | 0.018407 | 0.004192 | glutaminase complex |
| GO:2000232 | 0.018407 | 0.004151 | regulation of rRNA processing |
| GO:0003998 | 0.018407 | 0.004111 | acylphosphatase activity |
| GO:0004359 | 0.018407 | 0.004072 | glutaminase activity |
| GO:0004535 | 0.018407 | 0.004033 | poly(A)-specific ribonuclease activity |
| GO:0005546 | 0.018407 | 0.003995 | phosphatidylinositol-4,5-bisphosphate binding |
| GO:0006428 | 0.018407 | 0.003957 | isoleucyl-tRNA aminoacylation |
| GO:0006572 | 0.018407 | 0.003921 | tyrosine catabolic process |
| GO:0006654 | 0.018407 | 0.003885 | phosphatidic acid biosynthetic process |
| GO:0008614 | 0.018407 | 0.003849 | pyridoxine metabolic process |
| GO:0015267 | 0.018407 | 0.003815 | channel activity |
| GO:0090503 | 0.018407 | 0.003781 | RNA phosphodiester bond hydrolysis, exonucleolytic |
| GO:0008643 | 0.02096 | 0.004267 | carbohydrate transport |
| GO:0009835 | 0.021535 | 0.004345 | fruit ripening |
| GO:0030687 | 0.021535 | 0.004308 | preribosome, large subunit precursor |
| GO:0008271 | 0.021535 | 0.00427 | secondary active sulfate transmembrane transporter activity |
| GO:1902358 | 0.021535 | 0.004234 | sulfate transmembrane transport |
| GO:1901363 | 0.023773 | 0.004635 | heterocyclic compound binding |
| GO:0005488 | 0.025533 | 0.004936 | binding |
| GO:0006607 | 0.027933 | 0.005355 | NLS-bearing protein import into nucleus |
| GO:0009693 | 0.027933 | 0.00531 | ethylene biosynthetic process |
| GO:0005975 | 0.028302 | 0.005337 | carbohydrate metabolic process |
| GO:0005759 | 0.030963 | 0.005791 | mitochondrial matrix |
| GO:0003824 | 0.031336 | 0.005813 | catalytic activity |
| GO:0009834 | 0.031336 | 0.005767 | plant-type secondary cell wall biogenesis |
| GO:0005829 | 0.033251 | 0.006071 | cytosol |
| GO:0051539 | 0.034667 | 0.006279 | 4 iron, 4 sulfur cluster binding |
| GO:0016020 | 0.034833 | 0.00626 | membrane |
| GO:0006890 | 0.034862 | 0.006217 | retrograde vesicle-mediated transport, Golgi to ER |
| GO:0006508 | 0.035458 | 0.006274 | proteolysis |
| GO:0000460 | 0.036138 | 0.006346 | maturation of 5.8S rRNA |
| GO:0009925 | 0.036138 | 0.006298 | basal plasma membrane |
| GO:0030915 | 0.036138 | 0.00625 | Smc5-Smc6 complex |
| GO:0031325 | 0.036138 | 0.006204 | positive regulation of cellular metabolic process |
| GO:0034388 | 0.036138 | 0.006158 | Pwp2p-containing subcomplex of 90S preribosome |
| GO:0035434 | 0.036138 | 0.006113 | copper ion transmembrane transport |
| GO:0071731 | 0.036138 | 0.006068 | response to nitric oxide |
| GO:1902559 | 0.036138 | 0.006024 | 3'-phospho-5'-adenylyl sulfate transmembrane transport |
| GO:0000323 | 0.036138 | 0.005981 | lytic vacuole |
| GO:0004132 | 0.036138 | 0.005938 | dCMP deaminase activity |
| GO:0004712 | 0.036138 | 0.005896 | protein serine |
| GO:0004838 | 0.036138 | 0.005854 | L-tyrosine:2-oxoglutarate aminotransferase activity |
| GO:0005901 | 0.036138 | 0.005813 | caveola |
| GO:0006275 | 0.036138 | 0.005773 | regulation of DNA replication |
| GO:0009877 | 0.036138 | 0.005733 | nodulation |
| GO:0009927 | 0.036138 | 0.005694 | histidine phosphotransfer kinase activity |
| GO:0016471 | 0.036138 | 0.005655 | vacuolar proton-transporting V-type ATPase complex |
| GO:0016820 | 0.036138 | 0.005617 | hydrolase activity, acting on acid anhydrides, catalyzing transmembrane movement of substances |
| GO:0030173 | 0.036138 | 0.005579 | integral component of Golgi membrane |
| GO:0046964 | 0.036138 | 0.005542 | 3'-phosphoadenosine 5'-phosphosulfate transmembrane transporter activity |
| GO:0048868 | 0.036138 | 0.005505 | pollen tube development |
| GO:0090544 | 0.036138 | 0.005469 | BAF-type complex |
| GO:0044550 | 0.037742 | 0.005675 | secondary metabolite biosynthetic process |
| GO:0016491 | 0.03964 | 0.005921 | oxidoreductase activity |
| GO:0009793 | 0.044628 | 0.006623 | embryo development ending in seed dormancy |
| GO:0006310 | 0.046067 | 0.006793 | DNA recombination |
| GO:0006096 | 0.046067 | 0.00675 | glycolytic process |
| GO:0005524 | 0.048514 | 0.007063 | ATP binding |
| GO:0005576 | 0.04853 | 0.007021 | extracellular region |
| GO:0004674 | 0.048593 | 0.006986 | protein serine |
| GO:0016021 | 0.048645 | 0.00695 | integral component of membrane |
| GO:0005654 | 0.04998 | 0.007097 | nucleoplasm |
| GO:0048731 | 0.04998 | 0.007053 | system development |

Table S17. The enriched GO terms based on the genes from selective sweep analysis.

| Traits | SNP | Chr | Pos | P | R^2^ |
| --- | --- | --- | --- | --- | --- |
| palmitic acid | HiC_scaffold_1__139141751 | HiC_scaffold_1 | 139141751 | 8.5E-06 | 0.18 |
|  | HiC_scaffold_1__170821534 | HiC_scaffold_1 | 170821534 | 1.95E-06 | 0.18 |
|  | HiC_scaffold_1__209448757 | HiC_scaffold_1 | 209448757 | 7.95E-06 | 0.14 |
|  | HiC_scaffold_10__157109354 | HiC_scaffold_10 | 157109354 | 4.15E-06 | 0.12 |
|  | HiC_scaffold_10__210678038 | HiC_scaffold_10 | 210678038 | 9.06E-06 | 0.15 |
|  | HiC_scaffold_10__42908796 | HiC_scaffold_10 | 42908796 | 7.86E-06 | 0.11 |
|  | HiC_scaffold_10__42908832 | HiC_scaffold_10 | 42908832 | 2.59E-07 | 0.14 |
|  | HiC_scaffold_10__69521639 | HiC_scaffold_10 | 69521639 | 1.27E-06 | 0.13 |
|  | HiC_scaffold_11__100471147 | HiC_scaffold_11 | 100471147 | 3.07E-06 | 0.10 |
|  | HiC_scaffold_11__136799610 | HiC_scaffold_11 | 136799610 | 1.31E-06 | 0.13 |
|  | HiC_scaffold_11__136799612 | HiC_scaffold_11 | 136799612 | 8.15E-06 | 0.11 |
|  | HiC_scaffold_12__105976256 | HiC_scaffold_12 | 105976256 | 4.12E-06 | 0.15 |
|  | HiC_scaffold_12__105976316 | HiC_scaffold_12 | 105976316 | 2.67E-06 | 0.17 |
|  | HiC_scaffold_13__160284616 | HiC_scaffold_13 | 160284616 | 5.14E-06 | 0.14 |
|  | HiC_scaffold_13__47393700 | HiC_scaffold_13 | 47393700 | 6.24E-06 | 0.14 |
|  | HiC_scaffold_13__70383654 | HiC_scaffold_13 | 70383654 | 4.4E-06 | 0.11 |
|  | HiC_scaffold_14__34709403 | HiC_scaffold_14 | 34709403 | 9.68E-06 | 0.13 |
|  | HiC_scaffold_14__82157396 | HiC_scaffold_14 | 82157396 | 8.88E-06 | 0.16 |
|  | HiC_scaffold_15__17317574 | HiC_scaffold_15 | 17317574 | 6.99E-05 | 0.09 |
|  | HiC_scaffold_2__146858080 | HiC_scaffold_2 | 146858080 | 9E-06 | 0.11 |
|  | HiC_scaffold_2__177033680 | HiC_scaffold_2 | 177033680 | 6.41E-06 | 0.09 |
|  | HiC_scaffold_2__190301169 | HiC_scaffold_2 | 190301169 | 1.48E-06 | 0.16 |
|  | HiC_scaffold_3__168317526 | HiC_scaffold_3 | 168317526 | 9.43E-06 | 0.10 |
|  | HiC_scaffold_3__185416919 | HiC_scaffold_3 | 185416919 | 7.11E-04 | 0.06 |
|  | HiC_scaffold_3__55890 | HiC_scaffold_3 | 55890 | 4.37E-06 | 0.18 |
|  | HiC_scaffold_3__75852190 | HiC_scaffold_3 | 75852190 | 1.79E-06 | 0.14 |
|  | HiC_scaffold_3__77478528 | HiC_scaffold_3 | 77478528 | 7.52E-06 | 0.15 |
|  | HiC_scaffold_4__135256397 | HiC_scaffold_4 | 135256397 | 6.31E-06 | 0.15 |
|  | HiC_scaffold_4__39673709 | HiC_scaffold_4 | 39673709 | 3.31E-06 | 0.11 |
|  | HiC_scaffold_4__59543449 | HiC_scaffold_4 | 59543449 | 9.71E-06 | 0.16 |
|  | HiC_scaffold_5__126415884 | HiC_scaffold_5 | 126415884 | 2.58E-07 | 0.16 |
|  | HiC_scaffold_5__126415891 | HiC_scaffold_5 | 126415891 | 1.12E-06 | 0.14 |
|  | HiC_scaffold_5__96181064 | HiC_scaffold_5 | 96181064 | 6.6E-06 | 0.14 |
|  | HiC_scaffold_6__114241182 | HiC_scaffold_6 | 114241182 | 3.25E-06 | 0.14 |
|  | HiC_scaffold_6__135296315 | HiC_scaffold_6 | 135296315 | 3.93E-06 | 0.12 |
|  | HiC_scaffold_6__80466585 | HiC_scaffold_6 | 80466585 | 2.68E-06 | 0.13 |
|  | HiC_scaffold_7__140936608 | HiC_scaffold_7 | 140936608 | 4.31E-06 | 0.14 |
|  | HiC_scaffold_7__17870989 | HiC_scaffold_7 | 17870989 | 4.96E-06 | 0.18 |
|  | HiC_scaffold_8__59191065 | HiC_scaffold_8 | 59191065 | 9.39E-06 | 0.13 |
|  | HiC_scaffold_8__92183183 | HiC_scaffold_8 | 92183183 | 1.31E-06 | 0.14 |
|  | HiC_scaffold_9__82568084 | HiC_scaffold_9 | 82568084 | 4.5E-06 | 0.18 |
| palmitoleic acid | HiC_scaffold_1__3062838 | HiC_scaffold_1 | 3062838 | 2.35E-06 | 0.12 |
|  | HiC_scaffold_1__7714070 | HiC_scaffold_1 | 7714070 | 4.17E-06 | 0.11 |
|  | HiC_scaffold_1__98218723 | HiC_scaffold_1 | 98218723 | 9.73E-06 | 0.12 |
|  | HiC_scaffold_1__119655203 | HiC_scaffold_1 | 119655203 | 2.86E-06 | 0.15 |
|  | HiC_scaffold_1__128297678 | HiC_scaffold_1 | 128297678 | 9.48E-06 | 0.11 |
|  | HiC_scaffold_2__190000895 | HiC_scaffold_2 | 190000895 | 2.19E-06 | 0.13 |
|  | HiC_scaffold_2__191589382 | HiC_scaffold_2 | 191589382 | 4.62E-07 | 0.21 |
|  | HiC_scaffold_3__67921224 | HiC_scaffold_3 | 67921224 | 3.01E-06 | 0.14 |
|  | HiC_scaffold_3__130244479 | HiC_scaffold_3 | 130244479 | 2.86E-06 | 0.15 |
|  | HiC_scaffold_4__34241929 | HiC_scaffold_4 | 34241929 | 4.83E-06 | 0.18 |
|  | HiC_scaffold_5__91063298 | HiC_scaffold_5 | 91063298 | 6.47E-06 | 0.13 |
|  | HiC_scaffold_5__148570007 | HiC_scaffold_5 | 148570007 | 4.4E-06 | 0.14 |
|  | HiC_scaffold_6__31933969 | HiC_scaffold_6 | 31933969 | 6.25E-06 | 0.16 |
|  | HiC_scaffold_7__27852147 | HiC_scaffold_7 | 27852147 | 4.45E-06 | 0.12 |
|  | HiC_scaffold_7__36470653 | HiC_scaffold_7 | 36470653 | 6.09E-06 | 0.10 |
|  | HiC_scaffold_7__48624802 | HiC_scaffold_7 | 48624802 | 2.67E-06 | 0.10 |
|  | HiC_scaffold_7__105000262 | HiC_scaffold_7 | 105000262 | 2E-06 | 0.18 |
|  | HiC_scaffold_9__26142854 | HiC_scaffold_9 | 26142854 | 6.03E-07 | 0.13 |
|  | HiC_scaffold_10__9782744 | HiC_scaffold_10 | 9782744 | 6.63E-06 | 0.13 |
|  | HiC_scaffold_10__13817659 | HiC_scaffold_10 | 13817659 | 1.94E-06 | 0.11 |
|  | HiC_scaffold_10__22095521 | HiC_scaffold_10 | 22095521 | 1.91E-06 | 0.11 |
|  | HiC_scaffold_10__22095527 | HiC_scaffold_10 | 22095527 | 3.66E-06 | 0.10 |
|  | HiC_scaffold_10__28875308 | HiC_scaffold_10 | 28875308 | 4.26E-06 | 0.11 |
|  | HiC_scaffold_10__158994147 | HiC_scaffold_10 | 158994147 | 5.89E-06 | 0.16 |
|  | HiC_scaffold_11__48759083 | HiC_scaffold_11 | 48759083 | 9.92E-06 | 0.13 |
|  | HiC_scaffold_11__49010326 | HiC_scaffold_11 | 49010326 | 1.35E-06 | 0.12 |
|  | HiC_scaffold_11__49010329 | HiC_scaffold_11 | 49010329 | 2.09E-06 | 0.12 |
|  | HiC_scaffold_12__42622634 | HiC_scaffold_12 | 42622634 | 5.44E-06 | 0.11 |
|  | HiC_scaffold_12__71313007 | HiC_scaffold_12 | 71313007 | 6.84E-06 | 0.08 |
|  | HiC_scaffold_12__71313025 | HiC_scaffold_12 | 71313025 | 2.07E-07 | 0.14 |
|  | HiC_scaffold_12__71313033 | HiC_scaffold_12 | 71313033 | 4.3E-08 | 0.16 |
|  | HiC_scaffold_14__63536260 | HiC_scaffold_14 | 63536260 | 5.13E-06 | 0.11 |
|  | HiC_scaffold_14__110191894 | HiC_scaffold_14 | 110191894 | 4.82E-06 | 0.12 |
|  | HiC_scaffold_14__123466662 | HiC_scaffold_14 | 123466662 | 7.78E-06 | 0.11 |
|  | HiC_scaffold_14__133881125 | HiC_scaffold_14 | 133881125 | 5.12E-06 | 0.14 |
|  | HiC_scaffold_15__71687870 | HiC_scaffold_15 | 71687870 | 1.5E-06 | 0.11 |
| stearic acid | HiC_scaffold_2__77227287 | HiC_scaffold_2 | 77227287 | 2.75E-06 | 0.15 |
|  | HiC_scaffold_10__1067616 | HiC_scaffold_10 | 1067616 | 1.02E-04 | 0.08 |
|  | HiC_scaffold_10__112357685 | HiC_scaffold_10 | 112357685 | 5.42E-06 | 0.15 |
|  | HiC_scaffold_10__120325316 | HiC_scaffold_10 | 120325316 | 3.16E-06 | 0.18 |
|  | HiC_scaffold_10__120325321 | HiC_scaffold_10 | 120325321 | 8.54E-06 | 0.18 |
|  | HiC_scaffold_10__26345992 | HiC_scaffold_10 | 26345992 | 9.17E-06 | 0.16 |
|  | HiC_scaffold_11__21703094 | HiC_scaffold_11 | 21703094 | 6.45E-06 | 0.10 |
|  | HiC_scaffold_11__21703154 | HiC_scaffold_11 | 21703154 | 1.42E-06 | 0.10 |
|  | HiC_scaffold_11__43619536 | HiC_scaffold_11 | 43619536 | 0.000537 | 0.06 |
|  | HiC_scaffold_12__140556943 | HiC_scaffold_12 | 140556943 | 6.82E-06 | 0.12 |
|  | HiC_scaffold_12__18761442 | HiC_scaffold_12 | 18761442 | 7.16E-07 | 0.15 |
|  | HiC_scaffold_12__50117726 | HiC_scaffold_12 | 50117726 | 4.7E-06 | 0.11 |
|  | HiC_scaffold_15__97257674 | HiC_scaffold_15 | 97257674 | 8.86E-06 | 0.14 |
|  | HiC_scaffold_2__134141712 | HiC_scaffold_2 | 134141712 | 9.79E-06 | 0.11 |
|  | HiC_scaffold_3__154177839 | HiC_scaffold_3 | 154177839 | 4.4E-06 | 0.12 |
|  | HiC_scaffold_3__182382981 | HiC_scaffold_3 | 182382981 | 9.76E-07 | 0.14 |
|  | HiC_scaffold_3__72856430 | HiC_scaffold_3 | 72856430 | 3.55E-06 | 0.13 |
|  | HiC_scaffold_4__132471070 | HiC_scaffold_4 | 132471070 | 4.41E-06 | 0.13 |
|  | HiC_scaffold_4__27463027 | HiC_scaffold_4 | 27463027 | 8.13E-07 | 0.10 |
|  | HiC_scaffold_4__31039147 | HiC_scaffold_4 | 31039147 | 3.87E-06 | 0.11 |
|  | HiC_scaffold_5__112205021 | HiC_scaffold_5 | 112205021 | 2.59E-06 | 0.08 |
|  | HiC_scaffold_5__15908259 | HiC_scaffold_5 | 15908259 | 3.63E-06 | 0.11 |
|  | HiC_scaffold_6__11405013 | HiC_scaffold_6 | 11405013 | 8.53E-09 | 0.21 |
|  | HiC_scaffold_6__54469978 | HiC_scaffold_6 | 54469978 | 1.10E-04 | 0.08 |
|  | HiC_scaffold_6__553113 | HiC_scaffold_6 | 553113 | 5.34E-06 | 0.18 |
|  | HiC_scaffold_6__58047726 | HiC_scaffold_6 | 58047726 | 9.39E-06 | 0.07 |
|  | HiC_scaffold_7__112232428 | HiC_scaffold_7 | 112232428 | 2.46E-07 | 0.11 |
|  | HiC_scaffold_7__71298145 | HiC_scaffold_7 | 71298145 | 3.36E-04 | 0.05 |
|  | HiC_scaffold_8__28015344 | HiC_scaffold_8 | 28015344 | 2.41E-06 | 0.11 |
|  | HiC_scaffold_8__42575259 | HiC_scaffold_8 | 42575259 | 1.86E-04 | 0.08 |
|  | HiC_scaffold_9__110225418 | HiC_scaffold_9 | 110225418 | 7.8E-06 | 0.13 |
|  | HiC_scaffold_9__68874187 | HiC_scaffold_9 | 68874187 | 2.57E-06 | 0.09 |
|  | HiC_scaffold_9__98483162 | HiC_scaffold_9 | 98483162 | 9.97E-06 | 0.13 |
| oleic acid | HiC_scaffold_1__2422174 | HiC_scaffold_1 | 2422174 | 3.14E-06 | 0.13 |
|  | HiC_scaffold_1__87654508 | HiC_scaffold_1 | 87654508 | 3.22E-06 | 0.17 |
|  | HiC_scaffold_2__117321119 | HiC_scaffold_2 | 117321119 | 4.93E-06 | 0.12 |
|  | HiC_scaffold_2__160158458 | HiC_scaffold_2 | 160158458 | 4.19E-06 | 0.16 |
|  | HiC_scaffold_2__166475022 | HiC_scaffold_2 | 166475022 | 5.82E-06 | 0.11 |
|  | HiC_scaffold_3__47532011 | HiC_scaffold_3 | 47532011 | 5.68E-07 | 0.14 |
|  | HiC_scaffold_3__126379333 | HiC_scaffold_3 | 126379333 | 8.29E-06 | 0.13 |
|  | HiC_scaffold_4__53253123 | HiC_scaffold_4 | 53253123 | 8.79E-06 | 0.14 |
|  | HiC_scaffold_6__56916998 | HiC_scaffold_6 | 56916998 | 9.35E-06 | 0.16 |
|  | HiC_scaffold_6__80466585 | HiC_scaffold_6 | 80466585 | 7.87E-06 | 0.12 |
|  | HiC_scaffold_8__92183183 | HiC_scaffold_8 | 92183183 | 4.18E-06 | 0.13 |
|  | HiC_scaffold_10__1067616 | HiC_scaffold_10 | 1067616 | 9.55E-06 | 0.12 |
|  | HiC_scaffold_11__4456881 | HiC_scaffold_11 | 4456881 | 8.01E-06 | 0.10 |
|  | HiC_scaffold_12__26107719 | HiC_scaffold_12 | 26107719 | 8.91E-07 | 0.12 |
|  | HiC_scaffold_12__87028174 | HiC_scaffold_12 | 87028174 | 2.4E-08 | 0.27 |
|  | HiC_scaffold_12__189124962 | HiC_scaffold_12 | 189124962 | 9.93E-06 | 0.22 |
| linoleic acid | HiC_scaffold_1__2422174 | HiC_scaffold_1 | 2422174 | 6.79E-06 | 0.11 |
|  | HiC_scaffold_1__123847201 | HiC_scaffold_1 | 123847201 | 6.51E-06 | 0.13 |
|  | HiC_scaffold_2__85117427 | HiC_scaffold_2 | 85117427 | 2.45E-06 | 0.13 |
|  | HiC_scaffold_2__115211740 | HiC_scaffold_2 | 115211740 | 3.92E-06 | 0.14 |
|  | HiC_scaffold_2__117321119 | HiC_scaffold_2 | 117321119 | 5.44E-06 | 0.12 |
|  | HiC_scaffold_2__160158458 | HiC_scaffold_2 | 160158458 | 2.64E-06 | 0.16 |
|  | HiC_scaffold_2__165156651 | HiC_scaffold_2 | 165156651 | 7.88E-06 | 0.10 |
|  | HiC_scaffold_3__47532011 | HiC_scaffold_3 | 47532011 | 3.06E-07 | 0.14 |
|  | HiC_scaffold_6__17316862 | HiC_scaffold_6 | 17316862 | 6.34E-06 | 0.08 |
|  | HiC_scaffold_6__18938170 | HiC_scaffold_6 | 18938170 | 8.7E-06 | 0.09 |
|  | HiC_scaffold_7__120701530 | HiC_scaffold_7 | 120701530 | 3.33E-06 | 0.14 |
|  | HiC_scaffold_7__154675419 | HiC_scaffold_7 | 154675419 | 5.16E-06 | 0.09 |
|  | HiC_scaffold_9__2775915 | HiC_scaffold_9 | 2775915 | 3.57E-06 | 0.19 |
|  | HiC_scaffold_9__2775917 | HiC_scaffold_9 | 2775917 | 5.75E-06 | 0.18 |
|  | HiC_scaffold_9__7517585 | HiC_scaffold_9 | 7517585 | 1.87E-06 | 0.12 |
|  | HiC_scaffold_10__8904591 | HiC_scaffold_10 | 8904591 | 6.41E-06 | 0.10 |
|  | HiC_scaffold_10__193028875 | HiC_scaffold_10 | 193028875 | 6.37E-06 | 0.10 |
|  | HiC_scaffold_11__81332772 | HiC_scaffold_11 | 81332772 | 3.36E-06 | 0.13 |
|  | HiC_scaffold_11__87780092 | HiC_scaffold_11 | 87780092 | 6.32E-06 | 0.09 |
|  | HiC_scaffold_11__101138887 | HiC_scaffold_11 | 101138887 | 9.08E-06 | 0.12 |
|  | HiC_scaffold_12__26107719 | HiC_scaffold_12 | 26107719 | 2.38E-06 | 0.11 |
|  | HiC_scaffold_12__87028174 | HiC_scaffold_12 | 87028174 | 2.86E-08 | 0.27 |
|  | HiC_scaffold_12__189124962 | HiC_scaffold_12 | 189124962 | 5.28E-06 | 0.22 |
|  | HiC_scaffold_13__70354415 | HiC_scaffold_13 | 70354415 | 9.71E-06 | 0.12 |
|  | HiC_scaffold_13__108209574 | HiC_scaffold_13 | 108209574 | 5.23E-06 | 0.10 |
|  | HiC_scaffold_14__18195468 | HiC_scaffold_14 | 18195468 | 4.78E-06 | 0.10 |
|  | HiC_scaffold_14__136030424 | HiC_scaffold_14 | 136030424 | 1.5E-06 | 0.11 |
| linolenic acid | HiC_scaffold_1__12548307 | HiC_scaffold_1 | 12548307 | 1.49E-06 | 0.12 |
|  | HiC_scaffold_1__25006263 | HiC_scaffold_1 | 25006263 | 3.47E-06 | 0.13 |
|  | HiC_scaffold_1__112231664 | HiC_scaffold_1 | 112231664 | 2.95E-06 | 0.15 |
|  | HiC_scaffold_1__136392223 | HiC_scaffold_1 | 136392223 | 3.77E-07 | 0.12 |
|  | HiC_scaffold_1__139448115 | HiC_scaffold_1 | 139448115 | 1.3E-08 | 0.17 |
|  | HiC_scaffold_2__119620299 | HiC_scaffold_2 | 119620299 | 7.64E-06 | 0.11 |
|  | HiC_scaffold_2__166156607 | HiC_scaffold_2 | 166156607 | 5.88E-06 | 0.13 |
|  | HiC_scaffold_2__177092028 | HiC_scaffold_2 | 177092028 | 1.39E-06 | 0.11 |
|  | HiC_scaffold_2__181559520 | HiC_scaffold_2 | 181559520 | 4.29E-07 | 0.11 |
|  | HiC_scaffold_3__10462802 | HiC_scaffold_3 | 10462802 | 4.31E-06 | 0.12 |
|  | HiC_scaffold_3__47544109 | HiC_scaffold_3 | 47544109 | 9.45E-06 | 0.09 |
|  | HiC_scaffold_3__108736875 | HiC_scaffold_3 | 108736875 | 2.77E-06 | 0.09 |
|  | HiC_scaffold_3__179768686 | HiC_scaffold_3 | 179768686 | 7.8E-06 | 0.11 |
|  | HiC_scaffold_4__50041197 | HiC_scaffold_4 | 50041197 | 7.16E-08 | 0.11 |
|  | HiC_scaffold_4__130723426 | HiC_scaffold_4 | 130723426 | 7.21E-06 | 0.11 |
|  | HiC_scaffold_5__20193346 | HiC_scaffold_5 | 20193346 | 7.89E-07 | 0.09 |
|  | HiC_scaffold_5__37890482 | HiC_scaffold_5 | 37890482 | 5.9E-06 | 0.09 |
|  | HiC_scaffold_5__39289574 | HiC_scaffold_5 | 39289574 | 8.84E-08 | 0.11 |
|  | HiC_scaffold_5__50911604 | HiC_scaffold_5 | 50911604 | 9.04E-07 | 0.10 |
|  | HiC_scaffold_5__64846942 | HiC_scaffold_5 | 64846942 | 2.64E-06 | 0.10 |
|  | HiC_scaffold_5__80158408 | HiC_scaffold_5 | 80158408 | 7.4E-09 | 0.20 |
|  | HiC_scaffold_5__157084790 | HiC_scaffold_5 | 157084790 | 9.25E-06 | 0.12 |
|  | HiC_scaffold_6__73968120 | HiC_scaffold_6 | 73968120 | 1.55E-06 | 0.14 |
|  | HiC_scaffold_6__84071424 | HiC_scaffold_6 | 84071424 | 1.83E-06 | 0.16 |
|  | HiC_scaffold_7__9670131 | HiC_scaffold_7 | 9670131 | 8.01E-06 | 0.07 |
|  | HiC_scaffold_7__9906832 | HiC_scaffold_7 | 9906832 | 2.22E-06 | 0.14 |
|  | HiC_scaffold_7__45234869 | HiC_scaffold_7 | 45234869 | 5.63E-08 | 0.14 |
|  | HiC_scaffold_7__45234875 | HiC_scaffold_7 | 45234875 | 1.98E-06 | 0.10 |
|  | HiC_scaffold_7__116310905 | HiC_scaffold_7 | 116310905 | 2.22E-06 | 0.12 |
|  | HiC_scaffold_8__13996888 | HiC_scaffold_8 | 13996888 | 9.14E-06 | 0.08 |
|  | HiC_scaffold_9__4815504 | HiC_scaffold_9 | 4815504 | 9.41E-07 | 0.15 |
|  | HiC_scaffold_9__45333368 | HiC_scaffold_9 | 45333368 | 4.3E-06 | 0.09 |
|  | HiC_scaffold_9__73468022 | HiC_scaffold_9 | 73468022 | 5.4E-07 | 0.14 |
|  | HiC_scaffold_9__105756718 | HiC_scaffold_9 | 105756718 | 3.64E-06 | 0.16 |
|  | HiC_scaffold_10__72818928 | HiC_scaffold_10 | 72818928 | 3.85E-06 | 0.12 |
|  | HiC_scaffold_10__130356983 | HiC_scaffold_10 | 130356983 | 5.52E-06 | 0.08 |
|  | HiC_scaffold_10__162190002 | HiC_scaffold_10 | 162190002 | 1.94E-06 | 0.14 |
|  | HiC_scaffold_10__165358269 | HiC_scaffold_10 | 165358269 | 3.51E-06 | 0.09 |
|  | HiC_scaffold_11__154060448 | HiC_scaffold_11 | 154060448 | 9.76E-07 | 0.13 |
|  | HiC_scaffold_11__154529645 | HiC_scaffold_11 | 154529645 | 8.57E-06 | 0.09 |
|  | HiC_scaffold_12__24956058 | HiC_scaffold_12 | 24956058 | 5.12E-06 | 0.13 |
|  | HiC_scaffold_12__89572596 | HiC_scaffold_12 | 89572596 | 5.34E-06 | 0.11 |
|  | HiC_scaffold_12__172802978 | HiC_scaffold_12 | 172802978 | 4.33E-07 | 0.10 |
|  | HiC_scaffold_12__172822539 | HiC_scaffold_12 | 172822539 | 7.07E-06 | 0.08 |
|  | HiC_scaffold_12__186351572 | HiC_scaffold_12 | 186351572 | 1.33E-06 | 0.09 |
|  | HiC_scaffold_12__191801707 | HiC_scaffold_12 | 191801707 | 4.76E-06 | 0.09 |
|  | HiC_scaffold_12__206722506 | HiC_scaffold_12 | 206722506 | 2.06E-06 | 0.12 |
|  | HiC_scaffold_13__32554330 | HiC_scaffold_13 | 32554330 | 2.24E-06 | 0.12 |
|  | HiC_scaffold_13__42002077 | HiC_scaffold_13 | 42002077 | 3.25E-06 | 0.13 |
|  | HiC_scaffold_13__92314634 | HiC_scaffold_13 | 92314634 | 2.6E-06 | 0.12 |
|  | HiC_scaffold_13__123543823 | HiC_scaffold_13 | 123543823 | 5.33E-06 | 0.11 |
|  | HiC_scaffold_13__149193084 | HiC_scaffold_13 | 149193084 | 3.84E-06 | 0.15 |
|  | HiC_scaffold_13__162317211 | HiC_scaffold_13 | 162317211 | 4.55E-06 | 0.09 |
|  | HiC_scaffold_13__172759275 | HiC_scaffold_13 | 172759275 | 3.81E-06 | 0.08 |
|  | HiC_scaffold_14__52625541 | HiC_scaffold_14 | 52625541 | 7.03E-06 | 0.08 |
|  | HiC_scaffold_15__111042248 | HiC_scaffold_15 | 111042248 | 5.19E-06 | 0.10 |
|  | HiC_scaffold_15__144281657 | HiC_scaffold_15 | 144281657 | 8.21E-06 | 0.08 |
|  | scaffold_1017__145277 | scaffold_1017 | 145277 | 8.44E-07 | 0.15 |
| cis-11-eicosenoic acid | HiC_scaffold_2__98766767 | HiC_scaffold_2 | 98766767 | 7.11E-06 | 0.15 |
|  | HiC_scaffold_3__19984007 | HiC_scaffold_3 | 19984007 | 1.26E-06 | 0.14 |
|  | HiC_scaffold_3__90236621 | HiC_scaffold_3 | 90236621 | 5.6E-07 | 0.15 |
|  | HiC_scaffold_3__124247920 | HiC_scaffold_3 | 124247920 | 1.96E-06 | 0.10 |
|  | HiC_scaffold_3__171573664 | HiC_scaffold_3 | 171573664 | 1.38E-06 | 0.10 |
|  | HiC_scaffold_4__74443212 | HiC_scaffold_4 | 74443212 | 6.17E-07 | 0.11 |
|  | HiC_scaffold_4__126502032 | HiC_scaffold_4 | 126502032 | 8.59E-06 | 0.12 |
|  | HiC_scaffold_6__26205191 | HiC_scaffold_6 | 26205191 | 9.12E-07 | 0.12 |
|  | HiC_scaffold_8__3475564 | HiC_scaffold_8 | 3475564 | 8.44E-07 | 0.14 |
|  | HiC_scaffold_8__23660435 | HiC_scaffold_8 | 23660435 | 1.54E-07 | 0.28 |
|  | HiC_scaffold_9__61912769 | HiC_scaffold_9 | 61912769 | 8E-07 | 0.23 |
|  | HiC_scaffold_9__99085302 | HiC_scaffold_9 | 99085302 | 5.46E-06 | 0.09 |
|  | HiC_scaffold_10__169581288 | HiC_scaffold_10 | 169581288 | 9.71E-06 | 0.10 |
|  | HiC_scaffold_10__169581308 | HiC_scaffold_10 | 169581308 | 6.59E-06 | 0.11 |
|  | HiC_scaffold_10__198077523 | HiC_scaffold_10 | 198077523 | 7.16E-07 | 0.14 |
|  | HiC_scaffold_11__138102056 | HiC_scaffold_11 | 138102056 | 2.33E-06 | 0.11 |
|  | HiC_scaffold_12__16542833 | HiC_scaffold_12 | 16542833 | 1.48E-06 | 0.10 |
|  | HiC_scaffold_12__78038506 | HiC_scaffold_12 | 78038506 | 9.21E-06 | 0.12 |
|  | HiC_scaffold_12__154971061 | HiC_scaffold_12 | 154971061 | 3.35E-07 | 0.12 |
|  | HiC_scaffold_12__194887121 | HiC_scaffold_12 | 194887121 | 6.81E-06 | 0.10 |
|  | HiC_scaffold_13__118028762 | HiC_scaffold_13 | 118028762 | 4.11E-07 | 0.13 |
|  | HiC_scaffold_14__14821196 | HiC_scaffold_14 | 14821196 | 7.42E-06 | 0.08 |
|  | HiC_scaffold_14__40025519 | HiC_scaffold_14 | 40025519 | 9.32E-06 | 0.09 |
|  | HiC_scaffold_14__108978302 | HiC_scaffold_14 | 108978302 | 7.81E-06 | 0.12 |
|  | HiC_scaffold_15__2147280 | HiC_scaffold_15 | 2147280 | 8.65E-06 | 0.09 |
| OC | HiC_scaffold_15__65445682 | HiC_scaffold_15 | 65445682 | 5.22E-06 | 0.12 |
|  | HiC_scaffold_15__118682214 | HiC_scaffold_15 | 118682214 | 4.95E-06 | 0.10 |
|  | HiC_scaffold_1__7604473 | HiC_scaffold_1 | 7604473 | 5.73E-07 | 0.08 |
|  | HiC_scaffold_1__104302391 | HiC_scaffold_1 | 104302391 | 4.48E-06 | 0.07 |
|  | HiC_scaffold_1__130177706 | HiC_scaffold_1 | 130177706 | 4.67E-06 | 0.10 |
|  | HiC_scaffold_1__130177713 | HiC_scaffold_1 | 130177713 | 6.36E-06 | 0.10 |
|  | HiC_scaffold_1__169761177 | HiC_scaffold_1 | 169761177 | 2.46E-06 | 0.10 |
|  | HiC_scaffold_1__71336197 | HiC_scaffold_1 | 71336197 | 4.4E-06 | 0.07 |
|  | HiC_scaffold_1__74909761 | HiC_scaffold_1 | 74909761 | 8.54E-06 | 0.06 |
|  | HiC_scaffold_10__11667171 | HiC_scaffold_10 | 11667171 | 8.08E-07 | 0.07 |
|  | HiC_scaffold_10__135557077 | HiC_scaffold_10 | 135557077 | 5.85E-07 | 0.14 |
|  | HiC_scaffold_10__165731641 | HiC_scaffold_10 | 165731641 | 8.03E-06 | 0.08 |
|  | HiC_scaffold_10__175318316 | HiC_scaffold_10 | 175318316 | 1.34E-06 | 0.11 |
|  | HiC_scaffold_10__21981975 | HiC_scaffold_10 | 21981975 | 3.79E-04 | 0.04 |
|  | HiC_scaffold_10__27821823 | HiC_scaffold_10 | 27821823 | 5.97E-06 | 0.08 |
|  | HiC_scaffold_10__29454430 | HiC_scaffold_10 | 29454430 | 5.47E-04 | 0.04 |
|  | HiC_scaffold_10__50395485 | HiC_scaffold_10 | 50395485 | 8.17E-08 | 0.15 |
|  | HiC_scaffold_10__55897886 | HiC_scaffold_10 | 55897886 | 5.25E-04 | 0.04 |
|  | HiC_scaffold_11__138614777 | HiC_scaffold_11 | 138614777 | 2.47E-06 | 0.09 |
|  | HiC_scaffold_11__149746705 | HiC_scaffold_11 | 149746705 | 7.58E-06 | 0.07 |
|  | HiC_scaffold_11__167356847 | HiC_scaffold_11 | 167356847 | 7.16E-06 | 0.11 |
|  | HiC_scaffold_11__169383997 | HiC_scaffold_11 | 169383997 | 1.47E-08 | 0.13 |
|  | HiC_scaffold_11__42215447 | HiC_scaffold_11 | 42215447 | 3.25E-05 | 0.06 |
|  | HiC_scaffold_12__135271259 | HiC_scaffold_12 | 135271259 | 6.35E-07 | 0.12 |
|  | HiC_scaffold_12__14016822 | HiC_scaffold_12 | 14016822 | 8.69E-06 | 0.11 |
|  | HiC_scaffold_12__14369729 | HiC_scaffold_12 | 14369729 | 3.81E-06 | 0.07 |
|  | HiC_scaffold_12__65124871 | HiC_scaffold_12 | 65124871 | 7.66E-07 | 0.10 |
|  | HiC_scaffold_12__67653598 | HiC_scaffold_12 | 67653598 | 4.34E-06 | 0.07 |
|  | HiC_scaffold_12__73536013 | HiC_scaffold_12 | 73536013 | 4.24E-06 | 0.07 |
|  | HiC_scaffold_12__87853437 | HiC_scaffold_12 | 87853437 | 4.94E-06 | 0.13 |
|  | HiC_scaffold_12__9546732 | HiC_scaffold_12 | 9546732 | 9.51E-06 | 0.08 |
|  | HiC_scaffold_12__9644861 | HiC_scaffold_12 | 9644861 | 8.96E-04 | 0.04 |
|  | HiC_scaffold_13__131034884 | HiC_scaffold_13 | 131034884 | 4.68E-06 | 0.07 |
|  | HiC_scaffold_13__146918175 | HiC_scaffold_13 | 146918175 | 2.38E-06 | 0.08 |
|  | HiC_scaffold_13__179016485 | HiC_scaffold_13 | 179016485 | 6.73E-04 | 0.04 |
|  | HiC_scaffold_13__44130690 | HiC_scaffold_13 | 44130690 | 5.59E-06 | 0.09 |
|  | HiC_scaffold_13__49351550 | HiC_scaffold_13 | 49351550 | 7.7E-06 | 0.05 |
|  | HiC_scaffold_13__62754028 | HiC_scaffold_13 | 62754028 | 9.59E-06 | 0.07 |
|  | HiC_scaffold_13__65382590 | HiC_scaffold_13 | 65382590 | 6.6E-06 | 0.07 |
|  | HiC_scaffold_15__131619216 | HiC_scaffold_15 | 131619216 | 7.46E-06 | 0.08 |
|  | HiC_scaffold_15__40618972 | HiC_scaffold_15 | 40618972 | 7E-06 | 0.08 |
|  | HiC_scaffold_15__42241872 | HiC_scaffold_15 | 42241872 | 2.21E-06 | 0.10 |
|  | HiC_scaffold_15__4624317 | HiC_scaffold_15 | 4624317 | 1.92E-05 | 0.09 |
|  | HiC_scaffold_15__60604936 | HiC_scaffold_15 | 60604936 | 9.62E-06 | 0.07 |
|  | HiC_scaffold_15__67320828 | HiC_scaffold_15 | 67320828 | 1.39E-06 | 0.07 |
|  | HiC_scaffold_2__1004670 | HiC_scaffold_2 | 1004670 | 1.3E-06 | 0.14 |
|  | HiC_scaffold_2__105785364 | HiC_scaffold_2 | 105785364 | 5.86E-06 | 0.08 |
|  | HiC_scaffold_2__127512966 | HiC_scaffold_2 | 127512966 | 5.88E-06 | 0.07 |
|  | HiC_scaffold_2__187448379 | HiC_scaffold_2 | 187448379 | 4.07E-06 | 0.07 |
|  | HiC_scaffold_2__30098439 | HiC_scaffold_2 | 30098439 | 6.92E-06 | 0.07 |
|  | HiC_scaffold_2__30098439 | HiC_scaffold_2 | 30098439 | 6.92E-06 | 0.07 |
|  | HiC_scaffold_2__62899469 | HiC_scaffold_2 | 62899469 | 8.57E-06 | 0.10 |
|  | HiC_scaffold_2__6956451 | HiC_scaffold_2 | 6956451 | 4.4E-06 | 0.10 |
|  | HiC_scaffold_2__6956453 | HiC_scaffold_2 | 6956453 | 4.14E-06 | 0.10 |
|  | HiC_scaffold_3__130272842 | HiC_scaffold_3 | 130272842 | 4.81E-06 | 0.09 |
|  | HiC_scaffold_3__184949171 | HiC_scaffold_3 | 184949171 | 1.29E-06 | 0.11 |
|  | HiC_scaffold_3__3193421 | HiC_scaffold_3 | 3193421 | 8.65E-06 | 0.09 |
|  | HiC_scaffold_3__7318743 | HiC_scaffold_3 | 7318743 | 5.18E-06 | 0.07 |
|  | HiC_scaffold_3__76145034 | HiC_scaffold_3 | 76145034 | 9.81E-06 | 0.08 |
|  | HiC_scaffold_3__76145034 | HiC_scaffold_3 | 76145034 | 9.81E-06 | 0.08 |
|  | HiC_scaffold_4__116619193 | HiC_scaffold_4 | 116619193 | 1.64E-06 | 0.10 |
|  | HiC_scaffold_4__150305079 | HiC_scaffold_4 | 150305079 | 1.83E-06 | 0.14 |
|  | HiC_scaffold_4__158119122 | HiC_scaffold_4 | 158119122 | 9.93E-06 | 0.06 |
|  | HiC_scaffold_4__19039395 | HiC_scaffold_4 | 19039395 | 3.38E-07 | 0.13 |
|  | HiC_scaffold_4__19044341 | HiC_scaffold_4 | 19044341 | 1.59E-06 | 0.08 |
|  | HiC_scaffold_4__20906898 | HiC_scaffold_4 | 20906898 | 2.28E-06 | 0.08 |
|  | HiC_scaffold_4__29852175 | HiC_scaffold_4 | 29852175 | 8.39E-06 | 0.09 |
|  | HiC_scaffold_4__53257456 | HiC_scaffold_4 | 53257456 | 4.44E-06 | 0.08 |
|  | HiC_scaffold_4__5466954 | HiC_scaffold_4 | 5466954 | 7.95E-06 | 0.09 |
|  | HiC_scaffold_4__87076823 | HiC_scaffold_4 | 87076823 | 5.01E-06 | 0.10 |
|  | HiC_scaffold_5__103934514 | HiC_scaffold_5 | 103934514 | 3.09E-06 | 0.10 |
|  | HiC_scaffold_5__116227945 | HiC_scaffold_5 | 116227945 | 7.80E-04 | 0.05 |
|  | HiC_scaffold_5__121562414 | HiC_scaffold_5 | 121562414 | 7.96E-06 | 0.10 |
|  | HiC_scaffold_5__6744165 | HiC_scaffold_5 | 6744165 | 1.13E-06 | 0.08 |
|  | HiC_scaffold_5__9739452 | HiC_scaffold_5 | 9739452 | 2.52E-06 | 0.08 |
|  | HiC_scaffold_6__102506987 | HiC_scaffold_6 | 102506987 | 4.75E-08 | 0.16 |
|  | HiC_scaffold_6__106589158 | HiC_scaffold_6 | 106589158 | 2.91E-08 | 0.16 |
|  | HiC_scaffold_6__21962595 | HiC_scaffold_6 | 21962595 | 1.35E-05 | 0.08 |
|  | HiC_scaffold_6__28369773 | HiC_scaffold_6 | 28369773 | 4.36E-06 | 0.07 |
|  | HiC_scaffold_6__82621337 | HiC_scaffold_6 | 82621337 | 1.43E-06 | 0.10 |
|  | HiC_scaffold_6__90435668 | HiC_scaffold_6 | 90435668 | 3.4E-06 | 0.08 |
|  | HiC_scaffold_6__92709763 | HiC_scaffold_6 | 92709763 | 1.7E-06 | 0.11 |
|  | HiC_scaffold_6__93495812 | HiC_scaffold_6 | 93495812 | 1.03E-07 | 0.12 |
|  | HiC_scaffold_6__96625728 | HiC_scaffold_6 | 96625728 | 5.25E-06 | 0.08 |
|  | HiC_scaffold_7__130853869 | HiC_scaffold_7 | 130853869 | 8.54E-07 | 0.15 |
|  | HiC_scaffold_7__146983922 | HiC_scaffold_7 | 146983922 | 4.53E-07 | 0.08 |
|  | HiC_scaffold_7__16691639 | HiC_scaffold_7 | 16691639 | 2.93E-06 | 0.09 |
|  | HiC_scaffold_7__190010256 | HiC_scaffold_7 | 190010256 | 7.4E-06 | 0.10 |
|  | HiC_scaffold_7__51390013 | HiC_scaffold_7 | 51390013 | 3.14E-05 | 0.07 |
|  | HiC_scaffold_7__56914769 | HiC_scaffold_7 | 56914769 | 9.34E-06 | 0.07 |
|  | HiC_scaffold_7__58100332 | HiC_scaffold_7 | 58100332 | 9.5E-06 | 0.09 |
|  | HiC_scaffold_7__69712244 | HiC_scaffold_7 | 69712244 | 9.22E-07 | 0.10 |
|  | HiC_scaffold_7__87381277 | HiC_scaffold_7 | 87381277 | 4.98E-06 | 0.08 |
|  | HiC_scaffold_7__87613974 | HiC_scaffold_7 | 87613974 | 2.38E-06 | 0.07 |
|  | HiC_scaffold_7__9531706 | HiC_scaffold_7 | 9531706 | 2.58E-06 | 0.08 |
|  | HiC_scaffold_8__104289593 | HiC_scaffold_8 | 104289593 | 3.22E-06 | 0.11 |
|  | HiC_scaffold_8__120840884 | HiC_scaffold_8 | 120840884 | 3.03E-04 | 0.10 |
|  | HiC_scaffold_8__28828820 | HiC_scaffold_8 | 28828820 | 1.92E-06 | 0.13 |
|  | HiC_scaffold_8__31302202 | HiC_scaffold_8 | 31302202 | 4.87E-06 | 0.09 |
|  | HiC_scaffold_8__39600463 | HiC_scaffold_8 | 39600463 | 9.43E-04 | 0.07 |
|  | HiC_scaffold_8__41283847 | HiC_scaffold_8 | 41283847 | 8.02E-07 | 0.11 |
|  | HiC_scaffold_8__50604982 | HiC_scaffold_8 | 50604982 | 1.33E-06 | 0.09 |
|  | HiC_scaffold_9__109169986 | HiC_scaffold_9 | 109169986 | 4.84E-06 | 0.07 |
|  | HiC_scaffold_9__52057942 | HiC_scaffold_9 | 52057942 | 3.13E-06 | 0.13 |
|  | HiC_scaffold_9__69682564 | HiC_scaffold_9 | 69682564 | 9.89E-06 | 0.11 |
|  | HiC_scaffold_9__84556397 | HiC_scaffold_9 | 84556397 | 9.27E-06 | 0.12 |

Table S18. Loci significantly associated with oil traits in GWAS

| Trait | gene | p-value | FDR | Symbol |
| --- | --- | --- | --- | --- |
| C181 | augustus_masked-HiC_scaffold_10-processed-gene-1410.25 | 0.0046335 | 0.03846516 | ERF060 |
| C183 | augustus_masked-HiC_scaffold_10-processed-gene-548.10 | 0.0046625 | 0.03863589 | MALD3 |
| C160 | augustus_masked-HiC_scaffold_10-processed-gene-561.6 | 0.0001769 | 0.00325777 | ERF5 |
| C161 | augustus_masked-HiC_scaffold_10-processed-gene-561.6 | 0.0001434 | 0.00275469 | ERF5 |
| C181 | augustus_masked-HiC_scaffold_10-processed-gene-561.6 | 0.000766 | 0.01017026 | ERF5 |
| C182 | augustus_masked-HiC_scaffold_10-processed-gene-561.6 | 0.0050401 | 0.04088458 | ERF5 |
| OC | augustus_masked-HiC_scaffold_10-processed-gene-561.6 | 0.000766 | 0.01017026 | ERF5 |
| C161 | augustus_masked-HiC_scaffold_10-processed-gene-765.10 | 0.002375 | 0.0236609 | ERF026 |
| C183 | augustus_masked-HiC_scaffold_11-processed-gene-310.25 | 1.14E-06 | 4.57E-05 | oleosin |
| OC | augustus_masked-HiC_scaffold_11-processed-gene-310.25 | 1.46E-08 | 9.94E-07 | oleosin2 |
| OC | augustus_masked-HiC_scaffold_11-processed-gene-422.60 | 0.0005333 | 0.00768145 | ERF4 |
| C161 | augustus_masked-HiC_scaffold_12-processed-gene-1192.17 | 6.89E-05 | 0.00153339 | ERF034 |
| C181 | augustus_masked-HiC_scaffold_12-processed-gene-425.52 | 0.0009582 | 0.01202008 | GAI1 |
| C183 | augustus_masked-HiC_scaffold_13-processed-gene-1319.2 | 0.0043033 | 0.03642445 | CAC3 |
| OC | augustus_masked-HiC_scaffold_14-processed-gene-857.32 | 1.22E-06 | 4.85E-05 | oleosin1 |
| OC | augustus_masked-HiC_scaffold_15-processed-gene-46.25 | 1.36E-07 | 7.05E-06 | At4g10955 |
| C161 | augustus_masked-HiC_scaffold_1-processed-gene-131.29 | 0.001146 | 0.01374986 | KCS4 |
| C183 | augustus_masked-HiC_scaffold_1-processed-gene-131.29 | 0.0003895 | 0.00602956 | KCS4 |
| C201 | augustus_masked-HiC_scaffold_1-processed-gene-419.7 | 0.0005997 | 0.0084127 | At5g64080 |
| C160 | augustus_masked-HiC_scaffold_3-processed-gene-1044.6 | 2.50E-06 | 9.16E-05 | ERF109 |
| C161 | augustus_masked-HiC_scaffold_3-processed-gene-1044.6 | 4.66E-06 | 0.00015764 | ERF109 |
| C181 | augustus_masked-HiC_scaffold_3-processed-gene-1044.6 | 5.91E-05 | 0.00134812 | ERF109 |
| C182 | augustus_masked-HiC_scaffold_3-processed-gene-1044.6 | 0.0010629 | 0.01299267 | ERF109 |
| C183 | augustus_masked-HiC_scaffold_3-processed-gene-114.18 | 0.0027693 | 0.02649443 | KCS4 |
| C201 | augustus_masked-HiC_scaffold_3-processed-gene-114.18 | 0.0024538 | 0.02427169 | KCS4 |
| OC | augustus_masked-HiC_scaffold_3-processed-gene-114.18 | 8.68E-06 | 0.00026947 | KCS4 |
| C181 | augustus_masked-HiC_scaffold_3-processed-gene-143.21 | 8.60E-05 | 0.00183894 | AOS1 |
| C182 | augustus_masked-HiC_scaffold_3-processed-gene-143.21 | 0.0008468 | 0.01095124 | AOS1 |
| C160 | augustus_masked-HiC_scaffold_4-processed-gene-1257.1 | 5.71E-06 | 0.00018762 | ERF4 |
| C161 | augustus_masked-HiC_scaffold_4-processed-gene-1257.1 | 8.78E-05 | 0.00186893 | ERF4 |
| C181 | augustus_masked-HiC_scaffold_4-processed-gene-1257.1 | 0.0004261 | 0.00646129 | ERF4 |
| C183 | augustus_masked-HiC_scaffold_5-processed-gene-276.48 | 0.0021983 | 0.02233037 | ECI2 |
| C183 | augustus_masked-HiC_scaffold_7-processed-gene-1443.30 | 0.0003035 | 0.00496033 | ERF008 |
| C201 | augustus_masked-HiC_scaffold_7-processed-gene-1443.30 | 6.99E-07 | 2.99E-05 | ERF008 |
| OC | genemark-HiC_scaffold_10-processed-gene-219.24 | 6.76E-07 | 2.90E-05 | LPCAT |
| C183 | genemark-HiC_scaffold_14-processed-gene-756.48 | 0.002587 | 0.02521177 | SMO1-1 |
| C201 | genemark-HiC_scaffold_14-processed-gene-756.48 | 1.65E-05 | 0.00047017 | SMO1-1 |
| C181 | genemark-HiC_scaffold_3-processed-gene-549.43 | 0.0026924 | 0.02596524 | SAD6 |
| C182 | genemark-HiC_scaffold_3-processed-gene-549.43 | 0.0015208 | 0.01697158 | SAD6 |
| C181 | genemark-HiC_scaffold_3-processed-gene-558.40 | 0.0019259 | 0.02027949 | SAD6 |
| C182 | genemark-HiC_scaffold_3-processed-gene-558.40 | 0.0011538 | 0.01381924 | SAD6 |
| C180 | maker-HiC_scaffold_10-snap-gene-11.26 | 0.0058588 | 0.04541323 | SAD |
| C201 | maker-HiC_scaffold_10-snap-gene-11.26 | 4.99E-05 | 0.00117752 | SAD |
| OC | maker-HiC_scaffold_10-snap-gene-11.26 | 1.02E-06 | 4.17E-05 | SAD |
| C201 | maker-HiC_scaffold_10-snap-gene-1294.24 | 6.87E-07 | 2.94E-05 | PAS2A |
| C160 | maker-HiC_scaffold_10-snap-gene-1475.14 | 0.0002025 | 0.00361942 | DGK2 |
| C181 | maker-HiC_scaffold_10-snap-gene-1475.14 | 0.001322 | 0.01527585 | DGK2 |
| C182 | maker-HiC_scaffold_10-snap-gene-1475.14 | 0.0054456 | 0.04315431 | DGK2 |
| C183 | maker-HiC_scaffold_10-snap-gene-1475.14 | 2.67E-05 | 0.00070302 | DGK2 |
| C180 | maker-HiC_scaffold_10-snap-gene-1697.32 | 0.0045587 | 0.03802949 | MAGL |
| C201 | maker-HiC_scaffold_10-snap-gene-1776.4 | 0.0012239 | 0.01443606 | GH3.1 |
| C183 | maker-HiC_scaffold_10-snap-gene-424.19 | 0.0004511 | 0.0067587 | CPRD49 |
| C201 | maker-HiC_scaffold_10-snap-gene-424.19 | 0.0013183 | 0.01524417 | CPRD49 |
| C183 | maker-HiC_scaffold_10-snap-gene-428.0 | 0.0016023 | 0.01765419 | PLC2 |
| OC | maker-HiC_scaffold_10-snap-gene-435.32 | 0.0003764 | 0.00587486 | FAD7 |
| C201 | maker-HiC_scaffold_11-snap-gene-1234.0 | 0.0015985 | 0.01762326 | DGK5 |
| C182 | maker-HiC_scaffold_11-snap-gene-300.4 | 0.0019718 | 0.0206356 | CERK |
| C183 | maker-HiC_scaffold_11-snap-gene-336.5 | 0.0045235 | 0.03780613 | GLY1 |
| C160 | maker-HiC_scaffold_11-snap-gene-436.24 | 0.0008062 | 0.01056134 | PLC6 |
| C180 | maker-HiC_scaffold_11-snap-gene-436.24 | 0.0025257 | 0.02477309 | PLC6 |
| C181 | maker-HiC_scaffold_11-snap-gene-436.24 | 0.0015523 | 0.01722476 | PLC6 |
| C183 | maker-HiC_scaffold_11-snap-gene-436.24 | 0.0010231 | 0.01261345 | PLC6 |
| C161 | maker-HiC_scaffold_11-snap-gene-740.10 | 0.0009463 | 0.0119055 | PGPS1 |
| C181 | maker-HiC_scaffold_12-snap-gene-1001.39 | 0.0004883 | 0.00718124 | DGD2 |
| C182 | maker-HiC_scaffold_12-snap-gene-1001.39 | 0.0009716 | 0.01213954 | DGD2 |
| C201 | maker-HiC_scaffold_12-snap-gene-1454.23 | 0.0016444 | 0.01799764 | PDAT1 |
| C201 | maker-HiC_scaffold_12-snap-gene-1988.5 | 0.0005115 | 0.0074353 | FAD6 |
| OC | maker-HiC_scaffold_12-snap-gene-1988.5 | 2.68E-09 | 2.25E-07 | FAD6 |
| OC | maker-HiC_scaffold_12-snap-gene-582.15 | 0.001994 | 0.02081503 | FATB1 |
| C201 | maker-HiC_scaffold_12-snap-gene-642.8 | 1.57E-05 | 0.00044906 | MGD2 |
| C181 | maker-HiC_scaffold_12-snap-gene-813.0 | 0.0017875 | 0.01918136 | EIN3 |
| C183 | maker-HiC_scaffold_12-snap-gene-928.2 | 2.28E-05 | 0.00061601 | GPAT8 |
| C183 | maker-HiC_scaffold_12-snap-gene-992.20 | 0.0015087 | 0.01686546 | DGD2 |
| C181 | maker-HiC_scaffold_13-snap-gene-1073.0 | 0.0008994 | 0.01146797 | UGT86A1 |
| C183 | maker-HiC_scaffold_13-snap-gene-113.3 | 0.0016212 | 0.01780816 | KCR1 |
| C161 | maker-HiC_scaffold_13-snap-gene-157.5 | 3.45E-05 | 0.00086786 | VPS34 |
| C161 | maker-HiC_scaffold_13-snap-gene-1713.19 | 0.0056605 | 0.04434598 | GPDHC1 |
| C183 | maker-HiC_scaffold_13-snap-gene-1713.19 | 0.0003995 | 0.00615251 | GPDHC1 |
| C201 | maker-HiC_scaffold_13-snap-gene-1713.19 | 1.29E-07 | 6.74E-06 | GPDHC1 |
| C180 | maker-HiC_scaffold_13-snap-gene-901.0 | 0.0060076 | 0.04620594 | At5g03610 |
| C181 | maker-HiC_scaffold_14-snap-gene-1155.26 | 0.0006183 | 0.0086237 | -- |
| C183 | maker-HiC_scaffold_14-snap-gene-170.11 | 6.95E-05 | 0.0015449 | PLD1 |
| C201 | maker-HiC_scaffold_14-snap-gene-602.13 | 0.0011613 | 0.01388634 | KAS1 |
| C180 | maker-HiC_scaffold_14-snap-gene-602.15 | 0.0023255 | 0.02330462 | ARF9 |
| C183 | maker-HiC_scaffold_14-snap-gene-602.15 | 0.0006895 | 0.00939219 | ARF9 |
| C201 | maker-HiC_scaffold_14-snap-gene-602.15 | 0.0026981 | 0.02599972 | ARF9 |
| C183 | maker-HiC_scaffold_14-snap-gene-770.20 | 9.13E-06 | 0.00028171 | -- |
| C183 | maker-HiC_scaffold_14-snap-gene-9.29 | 0.0001028 | 0.00212558 | PLA2-ALPHA |
| C201 | maker-HiC_scaffold_14-snap-gene-9.29 | 0.0005185 | 0.00751391 | PLA2-ALPHA |
| C183 | maker-HiC_scaffold_15-snap-gene-124.25 | 0.000581 | 0.00820955 | LPLAT1 |
| C201 | maker-HiC_scaffold_15-snap-gene-124.25 | 5.99E-05 | 0.00136462 | LPLAT1 |
| C183 | maker-HiC_scaffold_15-snap-gene-1381.48 | 0.0004584 | 0.00683674 | DIM |
| C160 | maker-HiC_scaffold_15-snap-gene-173.30 | 0.0016448 | 0.01800024 | KAS3B |
| C161 | maker-HiC_scaffold_15-snap-gene-173.30 | 0.0002131 | 0.00376241 | KAS3B |
| C180 | maker-HiC_scaffold_15-snap-gene-173.30 | 0.0049342 | 0.04026272 | KAS3B |
| C181 | maker-HiC_scaffold_15-snap-gene-173.30 | 0.0043004 | 0.03640707 | KAS3B |
| C183 | maker-HiC_scaffold_1-snap-gene-1657.18 | 0.0001387 | 0.00267862 | NPC3 |
| C201 | maker-HiC_scaffold_1-snap-gene-1942.50 | 0.0010827 | 0.01318089 | PLDBETA1 |
| C160 | maker-HiC_scaffold_1-snap-gene-2124.37 | 3.14E-05 | 0.00080363 | PYL9 |
| C181 | maker-HiC_scaffold_1-snap-gene-2124.37 | 0.0019941 | 0.02081529 | PYL9 |
| C181 | maker-HiC_scaffold_2-snap-gene-1256.13 | 0.0005961 | 0.00837769 | CSLH1 |
| C183 | maker-HiC_scaffold_2-snap-gene-1261.39 | 0.001894 | 0.02003795 | HPL |
| OC | maker-HiC_scaffold_2-snap-gene-300.57 | 4.12E-07 | 1.87E-05 | IAA14 |
| C160 | maker-HiC_scaffold_2-snap-gene-696.31 | 0.0014178 | 0.01609103 | IN2-2 |
| C183 | maker-HiC_scaffold_2-snap-gene-813.20 | 0.0056968 | 0.04453017 | SAD |
| C201 | maker-HiC_scaffold_2-snap-gene-813.20 | 0.0052409 | 0.04200139 | SAD |
| C201 | maker-HiC_scaffold_3-snap-gene-1098.40 | 0.0018071 | 0.01934358 | ERF |
| C201 | maker-HiC_scaffold_3-snap-gene-1790.2 | 0.0038225 | 0.03346341 | ACCase |
| C201 | maker-HiC_scaffold_3-snap-gene-1860.8 | 0.0001838 | 0.00335481 | PLC2 |
| C183 | maker-HiC_scaffold_3-snap-gene-1895.4 | 0.0002426 | 0.00416084 | LTP2 |
| C183 | maker-HiC_scaffold_3-snap-gene-256.23 | 0.0003957 | 0.00610601 | SQE1 |
| C180 | maker-HiC_scaffold_3-snap-gene-390.5 | 0.0032167 | 0.0294944 | RAP2-4 |
| C182 | maker-HiC_scaffold_3-snap-gene-403.30 | 7.46E-05 | 0.00163604 | ACC1 |
| OC | maker-HiC_scaffold_3-snap-gene-761.26 | 0.0047126 | 0.03892274 | ARF17 |
| C201 | maker-HiC_scaffold_4-snap-gene-1318.7 | 0.0009642 | 0.01206902 | At4g04930 |
| C201 | maker-HiC_scaffold_4-snap-gene-1587.26 | 0.0028096 | 0.02676617 | PLA1 |
| C181 | maker-HiC_scaffold_4-snap-gene-181.28 | 0.0005115 | 0.0074353 | -- |
| C181 | maker-HiC_scaffold_4-snap-gene-194.25 | 0.0004911 | 0.00721365 | -- |
| C201 | maker-HiC_scaffold_4-snap-gene-597.17 | 0.0048986 | 0.0400597 | PLA1 |
| C181 | maker-HiC_scaffold_5-snap-gene-1008.1 | 0.0004359 | 0.00657273 | UGT80B1 |
| C182 | maker-HiC_scaffold_5-snap-gene-1008.1 | 0.0002162 | 0.00380442 | UGT80B1 |
| C160 | maker-HiC_scaffold_5-snap-gene-1816.15 | 0.0015008 | 0.0167993 | PLDDELTA |
| C161 | maker-HiC_scaffold_5-snap-gene-1816.15 | 4.84E-06 | 0.00016271 | PLDDELTA |
| C181 | maker-HiC_scaffold_5-snap-gene-1816.15 | 5.65E-05 | 0.0012997 | PLDDELTA |
| C182 | maker-HiC_scaffold_5-snap-gene-1816.15 | 0.0001987 | 0.00356426 | PLDDELTA |
| C183 | maker-HiC_scaffold_5-snap-gene-1816.15 | 0.0063091 | 0.04783098 | PLDDELTA |
| C160 | maker-HiC_scaffold_5-snap-gene-405.41 | 0.0012331 | 0.01451568 | DBR |
| C181 | maker-HiC_scaffold_5-snap-gene-412.25 | 0.0035087 | 0.03143687 | PED1 |
| C183 | maker-HiC_scaffold_5-snap-gene-412.25 | 0.0010011 | 0.01240596 | PED1 |
| C181 | maker-HiC_scaffold_5-snap-gene-544.27 | 0.0055064 | 0.04349999 | AAE16 |
| C160 | maker-HiC_scaffold_5-snap-gene-65.14 | 0.0027329 | 0.02624157 | MFP2 |
| C180 | maker-HiC_scaffold_5-snap-gene-65.14 | 0.0007056 | 0.00956555 | MFP2 |
| C181 | maker-HiC_scaffold_5-snap-gene-65.14 | 0.0011093 | 0.01342498 | MFP2 |
| C182 | maker-HiC_scaffold_5-snap-gene-65.14 | 0.0015345 | 0.01708363 | MFP2 |
| C183 | maker-HiC_scaffold_5-snap-gene-65.14 | 0.0002892 | 0.00478658 | MFP2 |
| C201 | maker-HiC_scaffold_5-snap-gene-717.4 | 0.0044531 | 0.03736355 | SAD |
| C160 | maker-HiC_scaffold_5-snap-gene-842.29 | 0.006601 | 0.0493806 | DBR |
| C180 | maker-HiC_scaffold_5-snap-gene-842.29 | 0.0025066 | 0.02464179 | DBR |
| C160 | maker-HiC_scaffold_5-snap-gene-844.40 | 0.0006977 | 0.00947941 | DBR |
| C180 | maker-HiC_scaffold_5-snap-gene-844.40 | 0.0046628 | 0.03863589 | DBR |
| C160 | maker-HiC_scaffold_5-snap-gene-849.1 | 2.77E-05 | 0.00072438 | DBR |
| C160 | maker-HiC_scaffold_5-snap-gene-850.40 | 6.78E-08 | 3.80E-06 | DBR |
| C201 | maker-HiC_scaffold_6-snap-gene-1330.2 | 0.0004426 | 0.0066523 | fabZ |
| OC | maker-HiC_scaffold_6-snap-gene-1330.2 | 0.0066684 | 0.04974712 | fabZ |
| C201 | maker-HiC_scaffold_6-snap-gene-1330.4 | 0.0005201 | 0.00753139 | At5g64080 |
| C183 | maker-HiC_scaffold_6-snap-gene-1375.20 | 2.26E-05 | 0.00061151 | At1g71691 |
| C201 | maker-HiC_scaffold_6-snap-gene-1375.20 | 0.0021188 | 0.02174317 | At1g71691 |
| C183 | maker-HiC_scaffold_6-snap-gene-1430.5 | 6.58E-05 | 0.00147835 | At1g71691 |
| C201 | maker-HiC_scaffold_6-snap-gene-1430.5 | 0.0004929 | 0.00723145 | At1g71691 |
| C201 | maker-HiC_scaffold_6-snap-gene-219.33 | 7.24E-05 | 0.00159653 | SDP1 |
| OC | maker-HiC_scaffold_6-snap-gene-219.33 | 9.74E-08 | 5.25E-06 | SDP1 |
| C183 | maker-HiC_scaffold_7-snap-gene-1041.7 | 0.0020986 | 0.02159782 | GDPD6 |
| C201 | maker-HiC_scaffold_7-snap-gene-1041.7 | 0.0023346 | 0.02337727 | GDPD6 |
| C161 | maker-HiC_scaffold_7-snap-gene-1123.47 | 0.0001028 | 0.00212558 | -- |
| C201 | maker-HiC_scaffold_7-snap-gene-1123.47 | 0.0006436 | 0.00890243 | -- |
| C183 | maker-HiC_scaffold_7-snap-gene-1801.1 | 0.0002775 | 0.00463121 | DGK7 |
| C161 | maker-HiC_scaffold_7-snap-gene-30.0 | 0.0009662 | 0.0120857 | KCR1 |
| C201 | maker-HiC_scaffold_7-snap-gene-30.0 | 0.0001096 | 0.00223648 | KCR1 |
| C161 | maker-HiC_scaffold_7-snap-gene-514.48 | 0.000438 | 0.00659739 | IAA26 |
| C183 | maker-HiC_scaffold_7-snap-gene-514.48 | 0.0006739 | 0.00923005 | IAA26 |
| C201 | maker-HiC_scaffold_7-snap-gene-514.48 | 4.94E-05 | 0.00116704 | IAA26 |
| OC | maker-HiC_scaffold_7-snap-gene-514.48 | 6.49E-18 | 3.34E-14 | IAA26 |
| C180 | maker-HiC_scaffold_7-snap-gene-713.15 | 0.0011558 | 0.01383385 | GLPK |
| C183 | maker-HiC_scaffold_7-snap-gene-713.15 | 3.99E-05 | 0.00097894 | GLPK |
| C183 | maker-HiC_scaffold_7-snap-gene-726.10 | 6.44E-05 | 0.00145094 | GLPK |
| C201 | maker-HiC_scaffold_7-snap-gene-726.10 | 0.0033017 | 0.03005806 | GLPK |
| C183 | maker-HiC_scaffold_7-snap-gene-809.20 | 0.0005574 | 0.00794983 | ARF2B |
| C180 | maker-HiC_scaffold_8-snap-gene-425.5 | 0.0012782 | 0.01490424 | NPC1 |
| C161 | maker-HiC_scaffold_8-snap-gene-655.6 | 0.0012908 | 0.01500503 | PDAT1 |
| C183 | maker-HiC_scaffold_8-snap-gene-75.16 | 0.003141 | 0.02898736 | ERF071 |
| C201 | maker-HiC_scaffold_9-snap-gene-1087.0 | 0.0021995 | 0.02233705 | ERF118 |
| C161 | maker-HiC_scaffold_9-snap-gene-161.37 | 0.0010332 | 0.01271496 | IAA26 |
| C180 | maker-HiC_scaffold_9-snap-gene-161.37 | 0.0018036 | 0.01931187 | IAA26 |
| C182 | maker-HiC_scaffold_9-snap-gene-161.37 | 0.0032013 | 0.02939503 | IAA26 |
| C183 | maker-HiC_scaffold_9-snap-gene-161.37 | 0.0028218 | 0.02685112 | IAA26 |
| C160 | maker-HiC_scaffold_9-snap-gene-41.0 | 0.0064473 | 0.04856175 | RAP2-2 |
| C180 | maker-HiC_scaffold_9-snap-gene-41.0 | 0.0009938 | 0.0123451 | RAP2-2 |
| C181 | maker-HiC_scaffold_9-snap-gene-41.0 | 0.0062571 | 0.04755505 | RAP2-2 |
| C182 | maker-HiC_scaffold_9-snap-gene-41.0 | 0.0063424 | 0.04801192 | RAP2-2 |
| C160 | maker-HiC_scaffold_9-snap-gene-44.12 | 0.0028113 | 0.02678115 | RAP2-2 |
| C161 | maker-HiC_scaffold_9-snap-gene-44.12 | 0.0001478 | 0.00281818 | RAP2-2 |
| C180 | maker-HiC_scaffold_9-snap-gene-44.12 | 0.0002945 | 0.00485545 | RAP2-2 |
| C181 | maker-HiC_scaffold_9-snap-gene-44.12 | 0.0002936 | 0.00484392 | RAP2-2 |
| C182 | maker-HiC_scaffold_9-snap-gene-44.12 | 0.000398 | 0.00613344 | RAP2-2 |
| C183 | maker-HiC_scaffold_9-snap-gene-44.12 | 0.0008329 | 0.01082676 | RAP2-2 |
| C201 | maker-HiC_scaffold_9-snap-gene-44.12 | 0.0014248 | 0.01613943 | RAP2-2 |
| C180 | maker-HiC_scaffold_9-snap-gene-46.28 | 0.0045876 | 0.03818175 | GLIP1 |
| C201 | maker-HiC_scaffold_9-snap-gene-590.17 | 0.0005753 | 0.00814667 | NPC6 |
| C183 | maker-HiC_scaffold_9-snap-gene-60.26 | 0.0021045 | 0.021649 | ALDH7B4 |
| C181 | maker-HiC_scaffold_9-snap-gene-79.20 | 0.0042292 | 0.03596981 | PDAT1 |
| C182 | maker-HiC_scaffold_9-snap-gene-79.20 | 0.0046706 | 0.03867679 | PDAT1 |
| C183 | maker-HiC_scaffold_9-snap-gene-79.20 | 0.0014474 | 0.01633186 | PDAT1 |
| C183 | maker-HiC_scaffold_9-snap-gene-86.0 | 0.0003025 | 0.00494796 | PDAT1 |
| C183 | maker-HiC_scaffold_9-snap-gene-935.24 | 0.0060774 | 0.04659673 | DGK1 |
| C183 | MSTRG.100674 | 0.0001482 | 0.00282331 | RAP2-7 |
| OC | MSTRG.102137 | 9.72E-10 | 9.32E-08 | PLA |
| C180 | MSTRG.102138 | 0.0008604 | 0.01109764 | -- |
| OC | MSTRG.102996 | 6.64E-09 | 4.95E-07 | FAD2 |
| C160 | MSTRG.103039 | 0.0025806 | 0.02516982 | KAS2 |
| OC | MSTRG.103039 | 0.0004655 | 0.00691795 | KAS2 |
| C201 | MSTRG.10568 | 0.0010496 | 0.01286081 | ECR |
| C201 | MSTRG.106929 | 0.0003391 | 0.00541381 | BCCP1 |
| C180 | MSTRG.108536 | 0.0034072 | 0.03077338 | SAD |
| C183 | MSTRG.11521 | 0.00119 | 0.01414603 | LTP1 |
| C201 | MSTRG.11521 | 0.0044454 | 0.03731222 | LTP1 |
| OC | MSTRG.12225 | 0.0012459 | 0.01462474 | FATB1 |
| C160 | MSTRG.15888 | 0.0045712 | 0.03810148 | IAA9 |
| C161 | MSTRG.15888 | 8.19E-05 | 0.00176552 | IAA9 |
| C181 | MSTRG.15888 | 0.0011257 | 0.01357899 | IAA9 |
| C182 | MSTRG.15888 | 0.0033935 | 0.03069313 | IAA9 |
| C183 | MSTRG.15888 | 0.0010867 | 0.01322012 | IAA9 |
| C201 | MSTRG.15888 | 0.0013336 | 0.0153649 | IAA9 |
| OC | MSTRG.15888 | 0.0011257 | 0.01357899 | IAA9 |
| C201 | MSTRG.17819 | 1.11E-06 | 4.47E-05 | At5g14450 |
| C183 | MSTRG.22189 | 0.0050546 | 0.04095174 | GPAT1 |
| C183 | MSTRG.26225 | 0.0038978 | 0.03394741 | DPL1 |
| OC | MSTRG.26275 | 0.0053764 | 0.04280123 | LTPG1 |
| C201 | MSTRG.26828 | 2.56E-06 | 9.35E-05 | At1g13580 |
| C180 | MSTRG.29061 | 0.0013985 | 0.01592102 | PLDZETA1 |
| C183 | MSTRG.29061 | 0.005633 | 0.04419532 | PLDZETA1 |
| OC | MSTRG.29061 | 0.0007441 | 0.0099513 | PLDZETA1 |
| C183 | MSTRG.29790 | 0.0020524 | 0.02124467 | ARF9 |
| C201 | MSTRG.29790 | 0.0025769 | 0.02514655 | ARF9 |
| OC | MSTRG.29868 | 7.92E-12 | 1.58E-09 | PYL3 |
| C201 | MSTRG.30196 | 0.003206 | 0.02943342 | tesB |
| C161 | MSTRG.3101 | 0.0017396 | 0.01878286 | ARF1 |
| C183 | MSTRG.3101 | 5.17E-05 | 0.00121022 | ARF1 |
| C180 | MSTRG.31678 | 0.0037758 | 0.03320373 | LTP8 |
| OC | MSTRG.33340 | 0.0004477 | 0.00671629 | DGAT1 |
| C183 | MSTRG.36247 | 1.73E-06 | 6.60E-05 | LPP2 |
| C160 | MSTRG.39668 | 0.0058554 | 0.04539558 | ACX4 |
| C161 | MSTRG.39668 | 0.0028658 | 0.02713758 | ACX4 |
| C160 | MSTRG.39910 | 0.0009963 | 0.01236854 | FAX3 |
| OC | MSTRG.4063 | 0.0011283 | 0.0135942 | LACS6 |
| C183 | MSTRG.4064 | 0.0004597 | 0.00684786 | ARF4 |
| C201 | MSTRG.4064 | 0.0010078 | 0.01246913 | ARF4 |
| OC | MSTRG.4064 | 4.35E-10 | 4.66E-08 | ARF4 |
| C183 | MSTRG.41142 | 0.0012597 | 0.01474177 | FAD2 |
| C201 | MSTRG.41142 | 0.0008249 | 0.01075221 | FAD2 |
| OC | MSTRG.41142 | 3.17E-06 | 0.00011285 | FAD2 |
| C183 | MSTRG.41249 | 0.0019304 | 0.02031052 | FAD2 |
| C201 | MSTRG.41249 | 0.0029083 | 0.02742948 | FAD2 |
| OC | MSTRG.41249 | 1.88E-05 | 0.00052299 | FAD2 |
| C201 | MSTRG.41250 | 0.0023542 | 0.02351266 | MOD1 |
| C183 | MSTRG.42275 | 6.36E-06 | 0.00020584 | DDB |
| C183 | MSTRG.42618 | 0.00023 | 0.00399551 | FATB1 |
| C201 | MSTRG.42618 | 0.004428 | 0.03720858 | FATB1 |
| OC | MSTRG.42618 | 2.65E-06 | 9.63E-05 | FATB1 |
| C180 | MSTRG.42901 | 0.0006978 | 0.00947973 | IAA27 |
| C183 | MSTRG.42901 | 5.12E-06 | 0.00017041 | IAA27 |
| C201 | MSTRG.42901 | 0.0002546 | 0.00431736 | IAA27 |
| C201 | MSTRG.43361 | 0.0016594 | 0.01811024 | DGAT1 |
| OC | MSTRG.43361 | 0.0001664 | 0.00309632 | DGAT1 |
| OC | MSTRG.43539 | 0.0001275 | 0.00250655 | LACS4 |
| C201 | MSTRG.43544 | 0.0001961 | 0.00352948 | TGD2 |
| C201 | MSTRG.44426 | 0.0013108 | 0.01517843 | KAS3A |
| C183 | MSTRG.45460 | 2.35E-05 | 0.00063209 | GPAT |
| OC | MSTRG.45460 | 6.02E-08 | 3.42E-06 | GPAT |
| OC | MSTRG.46426 | 4.89E-06 | 0.0001638 | oleosin1 |
| C180 | MSTRG.46427 | 0.0013765 | 0.01573078 | oleosin1 |
| OC | MSTRG.46427 | 2.56E-05 | 0.00067727 | oleosin1 |
| C201 | MSTRG.46820 | 0.0001558 | 0.00293779 | KCS11 |
| C201 | MSTRG.46846 | 0.0033492 | 0.03038499 | KCS11 |
| C183 | MSTRG.48801 | 0.0008133 | 0.01062901 | ppt-1 |
| C183 | MSTRG.53789 | 0.0018372 | 0.01959285 | MFP2 |
| C183 | MSTRG.53882 | 0.0047734 | 0.03931078 | MFP2 |
| C161 | MSTRG.53892 | 0.0008925 | 0.01139915 | PLDZETA1 |
| C201 | MSTRG.53892 | 9.59E-07 | 3.94E-05 | PLDZETA1 |
| OC | MSTRG.53892 | 1.16E-06 | 4.65E-05 | PLDZETA1 |
| C182 | MSTRG.61561 | 0.0011282 | 0.0135939 | At5g42250 |
| C181 | MSTRG.64869 | 0.0008903 | 0.01137566 | ECH2 |
| C182 | MSTRG.64869 | 0.0003689 | 0.00578825 | ECH2 |
| C161 | MSTRG.66546 | 0.0058297 | 0.04524779 | FAD7A-1 |
| C183 | MSTRG.66546 | 1.32E-06 | 5.20E-05 | FAD7A-1 |
| C201 | MSTRG.66546 | 0.0002016 | 0.00360682 | FAD7A-1 |
| OC | MSTRG.66546 | 9.10E-16 | 1.08E-12 | FAD7A-1 |
| C160 | MSTRG.69298 | 0.0017446 | 0.01882735 | SAC8 |
| C201 | MSTRG.69305 | 0.0009034 | 0.01150099 | ACX4 |
| C201 | MSTRG.69865 | 0.0005199 | 0.00752931 | FAD6 |
| OC | MSTRG.69865 | 0.000444 | 0.00667009 | FAD6 |
| C183 | MSTRG.70539 | 0.0002483 | 0.00423753 | FAH1 |
| C201 | MSTRG.70539 | 0.0001512 | 0.0028703 | FAH1 |
| OC | MSTRG.77111 | 5.43E-05 | 0.00125529 | LIP |
| C181 | MSTRG.79325 | 0.0020261 | 0.02105613 | AAE16 |
| C182 | MSTRG.79325 | 0.0010637 | 0.01300112 | AAE16 |
| OC | MSTRG.79956 | 0.0020598 | 0.02129613 | SAD |
| C183 | MSTRG.79957 | 0.0026958 | 0.02598537 | SAD |
| OC | MSTRG.79957 | 0.0008373 | 0.01086119 | SAD |
| C160 | MSTRG.80063 | 0.004585 | 0.03816744 | DBR |
| C160 | MSTRG.80233 | 0.0025924 | 0.02525061 | ERF021 |
| C160 | MSTRG.80571 | 0.0032374 | 0.0296265 | DBR |
| C201 | MSTRG.80704 | 0.0001256 | 0.00247839 | LACS9 |
| OC | MSTRG.80704 | 9.99E-06 | 0.00030431 | LACS9 |
| C183 | MSTRG.80812 | 0.0029453 | 0.02767706 | SAD |
| OC | MSTRG.81837 | 0.000946 | 0.0119027 | MGL |
| C180 | MSTRG.86106 | 0.0033277 | 0.03024758 | At3g48460 |
| C180 | MSTRG.89907 | 0.0008905 | 0.01137724 | DGK7 |
| C183 | MSTRG.89907 | 1.17E-06 | 4.67E-05 | DGK7 |
| C201 | MSTRG.89907 | 0.0020762 | 0.02142093 | DGK7 |
| C161 | MSTRG.90095 | 0.0018839 | 0.01995898 | LIP2 |
| C183 | MSTRG.90095 | 0.005964 | 0.0459644 | LIP2 |
| C201 | MSTRG.90095 | 0.0001707 | 0.00316038 | LIP2 |
| OC | MSTRG.90095 | 9.35E-11 | 1.26E-08 | LIP2 |
| C201 | MSTRG.90529 | 0.0059044 | 0.04564659 | WRI1 |
| C180 | MSTRG.9053 | 0.0057846 | 0.04499524 | ARF6 |
| C181 | MSTRG.9053 | 0.001691 | 0.01836013 | ARF6 |
| C182 | MSTRG.9053 | 0.0005198 | 0.00752887 | ARF6 |
| C180 | MSTRG.91015 | 0.0048138 | 0.03954862 | SQE3 |
| C183 | MSTRG.93964 | 0.0005623 | 0.00799888 | GDPD6 |
| C201 | MSTRG.93964 | 0.0001642 | 0.00306392 | GDPD6 |
| C181 | MSTRG.94255 | 0.0051978 | 0.04176255 | IAA11 |
| C182 | MSTRG.94255 | 0.0031604 | 0.0291172 | IAA11 |
| C183 | MSTRG.94587 | 0.0022463 | 0.02270303 | AIL6 |
| C161 | MSTRG.94931 | 0.0001057 | 0.00217361 | DGAT2D |
| OC | MSTRG.94931 | 6.30E-08 | 3.56E-06 | DGAT2 |
| C183 | MSTRG.97245 | 0.0002346 | 0.00405834 | PECT1 |
| C201 | MSTRG.99856 | 0.004787 | 0.03938541 | fabD |
| OC | MSTRG.99856 | 1.67E-06 | 6.38E-05 | fabD |
| C201 | MSTRG.99951 | 3.47E-05 | 0.00087217 | PLC4 |
| OC | MSTRG.99951 | 3.45E-05 | 0.00086786 | PLC4 |
| C181 | snap_masked-HiC_scaffold_10-processed-gene-206.20 | 0.0046398 | 0.03850462 | SDP6 |
| C201 | snap_masked-HiC_scaffold_10-processed-gene-558.29 | 0.0002123 | 0.00375182 | oleosin3 |
| OC | snap_masked-HiC_scaffold_10-processed-gene-558.29 | 9.12E-08 | 4.95E-06 | oleosin3 |
| C160 | snap_masked-HiC_scaffold_11-processed-gene-112.19 | 0.0063373 | 0.04798085 | CRF4 |
| C181 | snap_masked-HiC_scaffold_11-processed-gene-112.19 | 0.0006996 | 0.009502 | CRF4 |
| C182 | snap_masked-HiC_scaffold_11-processed-gene-112.19 | 0.0040205 | 0.03468749 | ERF4 |
| C160 | snap_masked-HiC_scaffold_12-processed-gene-1085.15 | 0.0055715 | 0.04388068 | SAUR36 |
| C181 | snap_masked-HiC_scaffold_13-processed-gene-1091.11 | 0.0027309 | 0.02622846 | ARF18 |
| C182 | snap_masked-HiC_scaffold_13-processed-gene-1091.11 | 0.0028552 | 0.02707274 | ARF18 |
| C180 | snap_masked-HiC_scaffold_15-processed-gene-99.14 | 0.0020187 | 0.02099836 | -- |
| C183 | snap_masked-HiC_scaffold_2-processed-gene-1570.2 | 0.0014558 | 0.01641166 | ERF4 |
| C201 | snap_masked-HiC_scaffold_3-processed-gene-449.16 | 0.0001044 | 0.00215216 | ERF110 |
| C181 | snap_masked-HiC_scaffold_4-processed-gene-1236.8 | 0.0009581 | 0.01201915 | ARF15 |
| C182 | snap_masked-HiC_scaffold_4-processed-gene-1236.8 | 0.0007201 | 0.00971066 | ARF15 |
| C160 | snap_masked-HiC_scaffold_5-processed-gene-438.20 | 0.0007906 | 0.01040989 | DBR |
| C183 | snap_masked-HiC_scaffold_9-processed-gene-47.14 | 0.0014465 | 0.0163264 | SAUR77 |

Table S19. The key candidate genes mined by qGWAS in *C. oleifera* association population

|  | id | | num | per | ratio | class | ID | Descrption | Pvalue | qvalue |
| --- | --- | --- | --- | --- | --- | --- | --- | --- | --- | --- |
| Genes with cis-eQTLs | Ribosome | | 152 | 6.487 | 0.311 | Genetic Information Processing | ko03010 | Ribosome | 8.93E-07 | 7.05E-05 |
|  | Spliceosome | | 128 | 5.463 | 0.321 | Genetic Information Processing | ko03040 | Spliceosome | 1.08E-06 | 7.05E-05 |
|  | Purine metabolism | | 99 | 4.225 | 0.296 | Metabolism | ko00230 | Purine metabolism | 4.91E-04 | 2.15E-02 |
|  | Oxidative phosphorylation | 84 | | 3.585 | 0.289 | Metabolism | ko00190 | Oxidative phosphorylation | 2.91E-03 | 7.32E-02 |
|  | Glycosylphosphatidylinositol(GPI)-anchor biosynthesis | | 17 | 0.726 | 0.415 | Metabolism | ko00563 | Glycosylphosphatidylinositol(GPI)-anchor biosynthesis | 3.79E-03 | 7.32E-02 |
|  | Pyruvate metabolism | | 66 | 2.817 | 0.296 | Metabolism | ko00620 | Pyruvate metabolism | 4.13E-03 | 7.32E-02 |
|  | Glyoxylate and dicarboxylate metabolism | | 57 | 2.433 | 0.303 | Metabolism | ko00630 | Glyoxylate and dicarboxylate metabolism | 4.20E-03 | 7.32E-02 |
|  | Carbon metabolism | | 162 | 6.914 | 0.263 | Metabolism | ko01200 | Carbon metabolism | 4.47E-03 | 7.32E-02 |
|  | Biosynthesis of amino acids | | 135 | 5.762 | 0.266 | Metabolism | ko01230 | Biosynthesis of amino acids | 6.19E-03 | 9.00E-02 |
|  | Glycosphingolipid biosynthesis - ganglio series | | 6 | 0.256 | 0.6 | Metabolism | ko00604 | Glycosphingolipid biosynthesis - ganglio series | 1.02E-02 | 1.33E-01 |
|  | Peroxisome | | 52 | 2.219 | 0.287 | Cellular Processes | ko04146 | Peroxisome | 1.81E-02 | 2.08E-01 |
|  | Fatty acid biosynthesis | | 32 | 1.366 | 0.311 | Metabolism | ko00061 | Fatty acid biosynthesis | 1.91E-02 | 2.08E-01 |
|  | Aminoacyl-tRNA biosynthesis | | 57 | 2.433 | 0.279 | Genetic Information Processing | ko00970 | Aminoacyl-tRNA biosynthesis | 2.41E-02 | 2.43E-01 |
|  | Ribosome biogenesis in eukaryotes | | 52 | 2.219 | 0.278 | Genetic Information Processing | ko03008 | Ribosome biogenesis in eukaryotes | 3.28E-02 | 2.91E-01 |
|  | Ascorbate and aldarate metabolism | | 23 | 0.982 | 0.315 | Metabolism | ko00053 | Ascorbate and aldarate metabolism | 3.63E-02 | 2.91E-01 |
|  | Fatty acid metabolism | | 46 | 1.963 | 0.28 | Metabolism | ko01212 | Fatty acid metabolism | 3.72E-02 | 2.91E-01 |
|  | Glycine, serine and threonine metabolism | | 41 | 1.75 | 0.285 | Metabolism | ko00260 | Glycine, serine and threonine metabolism | 3.77E-02 | 2.91E-01 |
| Trans-eQTLs target genes | Ribosome | | 331 | 6.391 | 0.677 | Genetic Information Processing | ko03010 | Ribosome | 1.12E-18 | 1.49E-16 |
|  | Spliceosome | | 265 | 5.117 | 0.664 | Genetic Information Processing | ko03040 | Spliceosome | 1.22E-13 | 8.11E-12 |
|  | mRNA surveillance pathway | | 185 | 3.572 | 0.611 | Genetic Information Processing | ko03015 | mRNA surveillance pathway | 5.15E-06 | 2.28E-04 |
|  | Purine metabolism | | 201 | 3.881 | 0.602 | Metabolism | ko00230 | Purine metabolism | 7.97E-06 | 2.65E-04 |
|  | Protein processing in endoplasmic reticulum | | 297 | 5.735 | 0.574 | Genetic Information Processing | ko04141 | Protein processing in endoplasmic reticulum | 1.59E-05 | 4.24E-04 |
|  | Ribosome biogenesis in eukaryotes | | 116 | 2.24 | 0.62 | Genetic Information Processing | ko03008 | Ribosome biogenesis in eukaryotes | 1.12E-04 | 2.37E-03 |
|  | Autophagy - other eukaryotes | | 56 | 1.081 | 0.691 | Cellular Processes | ko04136 | Autophagy - other eukaryotes | 1.25E-04 | 2.37E-03 |
|  | Fatty acid biosynthesis | | 67 | 1.294 | 0.65 | Metabolism | ko00061 | Fatty acid biosynthesis | 4.72E-04 | 7.85E-03 |
|  | RNA transport | | 249 | 4.808 | 0.558 | Genetic Information Processing | ko03013 | RNA transport | 8.36E-04 | 1.24E-02 |
|  | Basal transcription factors | | 51 | 0.985 | 0.662 | Genetic Information Processing | ko03022 | Basal transcription factors | 1.19E-03 | 1.58E-02 |
|  | Circadian rhythm - plant | | 55 | 1.062 | 0.64 | Organismal Systems | ko04712 | Circadian rhythm - plant | 2.61E-03 | 3.16E-02 |
|  | Glycosylphosphatidylinositol(GPI)-anchor biosynthesis | | 29 | 0.56 | 0.707 | Metabolism | ko00563 | Glycosylphosphatidylinositol(GPI)-anchor biosynthesis | 3.16E-03 | 3.50E-02 |
|  | Fatty acid metabolism | | 97 | 1.873 | 0.591 | Metabolism | ko01212 | Fatty acid metabolism | 3.57E-03 | 3.66E-02 |
|  | Pyruvate metabolism | | 128 | 2.472 | 0.574 | Metabolism | ko00620 | Pyruvate metabolism | 4.15E-03 | 3.82E-02 |
|  | Arginine biosynthesis | | 35 | 0.676 | 0.673 | Metabolism | ko00220 | Arginine biosynthesis | 4.58E-03 | 3.82E-02 |
|  | Phagosome | | 124 | 2.394 | 0.574 | Cellular Processes | ko04145 | Phagosome | 4.69E-03 | 3.82E-02 |
|  | Carbon metabolism | | 330 | 6.372 | 0.536 | Metabolism | ko01200 | Carbon metabolism | 4.88E-03 | 3.82E-02 |
| Genes covered the trans-eQTLs | Ribosome | | 433 | 5.154 | 0.885 | Genetic Information Processing | ko03010 | Ribosome | 3.04E-09 | 4.07E-07 |
|  | Spliceosome | | 352 | 4.19 | 0.882 | Genetic Information Processing | ko03040 | Spliceosome | 2.47E-07 | 1.66E-05 |
|  | Purine metabolism | | 294 | 3.5 | 0.88 | Metabolism | ko00230 | Purine metabolism | 3.91E-06 | 1.74E-04 |
|  | Peroxisome | | 161 | 1.916 | 0.89 | Cellular Processes | ko04146 | Peroxisome | 1.80E-04 | 4.96E-03 |
|  | Ribosome biogenesis in eukaryotes | | 166 | 1.976 | 0.888 | Genetic Information Processing | ko03008 | Ribosome biogenesis in eukaryotes | 1.85E-04 | 4.96E-03 |
|  | Biosynthesis of amino acids | | 430 | 5.118 | 0.846 | Metabolism | ko01230 | Biosynthesis of amino acids | 2.53E-04 | 5.65E-03 |
|  | RNA transport | | 378 | 4.499 | 0.848 | Genetic Information Processing | ko03013 | RNA transport | 4.81E-04 | 9.21E-03 |
|  | Autophagy - other eukaryotes | | 75 | 0.893 | 0.926 | Cellular Processes | ko04136 | Autophagy - other eukaryotes | 5.81E-04 | 9.68E-03 |
|  | Ubiquitin mediated proteolysis | | 257 | 3.059 | 0.86 | Genetic Information Processing | ko04120 | Ubiquitin mediated proteolysis | 6.50E-04 | 9.68E-03 |
|  | SNARE interactions in vesicular transport | | 78 | 0.928 | 0.918 | Genetic Information Processing | ko04130 | SNARE interactions in vesicular transport | 9.63E-04 | 1.29E-02 |
|  | Endocytosis | | 319 | 3.797 | 0.848 | Cellular Processes | ko04144 | Endocytosis | 1.13E-03 | 1.37E-02 |
|  | Glyoxylate and dicarboxylate metabolism | | 164 | 1.952 | 0.872 | Metabolism | ko00630 | Glyoxylate and dicarboxylate metabolism | 1.46E-03 | 1.56E-02 |
|  | Fatty acid metabolism | | 144 | 1.714 | 0.878 | Metabolism | ko01212 | Fatty acid metabolism | 1.52E-03 | 1.56E-02 |
|  | Pyrimidine metabolism | | 243 | 2.893 | 0.853 | Metabolism | ko00240 | Pyrimidine metabolism | 2.42E-03 | 2.32E-02 |
|  | Glycine, serine and threonine metabolism | | 126 | 1.5 | 0.875 | Metabolism | ko00260 | Glycine, serine and threonine metabolism | 3.95E-03 | 3.53E-02 |
|  | Carbon metabolism | | 510 | 6.071 | 0.828 | Metabolism | ko01200 | Carbon metabolism | 4.24E-03 | 3.55E-02 |
|  | Alanine, aspartate and glutamate metabolism | | 84 | 1 | 0.894 | Metabolism | ko00250 | Alanine, aspartate and glutamate metabolism | 4.78E-03 | 3.77E-02 |
|  | Aminoacyl-tRNA biosynthesis | | 175 | 2.083 | 0.858 | Genetic Information Processing | ko00970 | Aminoacyl-tRNA biosynthesis | 5.48E-03 | 4.08E-02 |
|  | Protein processing in endoplasmic reticulum | | 429 | 5.107 | 0.83 | Genetic Information Processing | ko04141 | Protein processing in endoplasmic reticulum | 6.20E-03 | 4.37E-02 |
|  | RNA degradation | | 235 | 2.797 | 0.845 | Genetic Information Processing | ko03018 | RNA degradation | 7.09E-03 | 4.75E-02 |

Table S20. Summary of the significantly enriched KEGG pathways of the genes with cis-eQTLs, trans-eQTLs targeted genes and genes covered the trans-eQTLs.

| SNP loci | Total number | Verified | Verificati-on rate | Geno-type1 | RNA-seq | Sanger | Geno-  type2 | RNA-seq | Sanger | Geno-  type3 | RNA-seq | Sanger |
| --- | --- | --- | --- | --- | --- | --- | --- | --- | --- | --- | --- | --- |
| HiC_scaffold_6_21921279 | 213 | 211 | 99.06% | GG | 186 | 185 | GA | 27 | 26 | - | - | - |
| HiC_scaffold_6_21921481 | 212 | 196 | 92.45% | CC | 47 | 46 | CT | 149 | 143 | TT | 16 | 7 |
| HiC_scaffold_6_21921487 | 211 | 194 | 91.94% | AA | 17 | 4 | AG | 147 | 144 | GG | 47 | 46 |
| HiC_scaffold_6_21921722 | 213 | 211 | 99.06% | CC | 145 | 145 | CT | 68 | 66 | - | - | - |
| HiC_scaffold_7_51393500 | 126 | 122 | 96.83% | AA | 109 | 108 | AT | 17 | 14 | - | - | - |
| HiC_scaffold_7_51393143 | 212 | 210 | 99.06% | TT | 189 | 189 | CT | 23 | 21 | - | - | - |
| HiC_scaffold_7_51390025 | 211 | 211 | 100.00% | CT | 78 | 78 | TT | 133 | 133 | - | - | - |
| HiC_scaffold_8_39586734 | 199 | 197 | 98.99% | CC | 151 | 151 | CT | 48 | 46 | - | - | - |
| HiC_scaffold_8_39589896 | 212 | 212 | 100.00% | AT | 206 | 206 | TT | 6 | 6 | - | - | - |
| HiC_scaffold_8_39590966 | 213 | 212 | 99.53% | CC | 134 | 134 | CT | 79 | 78 | - | - | - |
| HiC_scaffold_8_39590985 | 213 | 211 | 99.06% | AT | 205 | 203 | TT | 8 | 8 | - | - | - |
| HiC_scaffold_3_185417048 | 213 | 211 | 99.06% | GC | 31 | 30 | GG | 182 | 181 | - | - | - |
| HiC_scaffold_3_185416953 | 213 | 213 | 100.00% | GA | 34 | 34 | GG | 179 | 179 | - | - | - |
| HiC_scaffold_3_185416913 | 213 | 206 | 96.71% | CC | 171 | 168 | CA | 42 | 38 | - | - | - |
| HiC_scaffold_3_185411128 | 210 | 209 | 99.52% | CC | 174 | 174 | CT | 36 | 35 | - | - | - |
| HiC_scaffold_15_17317824 | 213 | 213 | 100.00% | CC | 184 | 184 | CT | 29 | 29 | - | - | - |
| HiC_scaffold_10_1115361 | 186 | 178 | 95.70% | GT | 156 | 151 | TT | 30 | 27 | - | - | - |
| HiC_scaffold_10_1115176 | 213 | 211 | 99.06% | CA | 32 | 30 | CC | 181 | 181 | - | - | - |
| HiC_scaffold_10_1113856 | 213 | 212 | 99.53% | TT | 125 | 124 | TA | 88 | 88 | - | - | - |
| HiC_scaffold_10_1111762 | 149 | 132 | 88.59% | GG | 112 | 112 | GT | 37 | 20 | - | - | - |
| HiC_scaffold_3_55811950 | 212 | 211 | 99.53% | CC | 112 | 111 | CT | 100 | 100 | - | - | - |
| HiC_scaffold_3_55809902 | 213 | 212 | 99.53% | AA | 185 | 184 | AC | 28 | 28 | - | - | - |
| HiC_scaffold_3_55809903 | 213 | 196 | 92.02% | GT | 29 | 29 | TT | 184 | 167 | - | - | - |
| Total | 4696 | 4591 | 97.76% | - | - | - | - | - | - | - | - | - |

Table S21. The summary of Sanger sequencing validation of SNPs identified by the RNA-seq analysis.

| sample | Group | PC1 | PC2 | PC3 | PC4 | PC5 | PC6 | PC7 | PC8 | PC9 | PC10 |
| --- | --- | --- | --- | --- | --- | --- | --- | --- | --- | --- | --- |
| L1 | MJX | -0.019 | 0.042 | 0.063 | 0.036 | -0.026 | -0.046 | -0.041 | -0.057 | 0.002 | 0.338 |
| L10 | MJX | 0.032 | -0.003 | 0.018 | -0.013 | 0.006 | 0.025 | 0.005 | 0.024 | 0.044 | 0.000 |
| L100 | MJX | 0.001 | 0.031 | 0.040 | 0.005 | 0.003 | -0.003 | -0.004 | -0.017 | 0.010 | 0.063 |
| L101 | MJX | -0.011 | -0.014 | 0.033 | -0.012 | -0.056 | 0.008 | 0.053 | -0.024 | -0.061 | -0.009 |
| L102 | unknown | -0.012 | 0.024 | 0.073 | 0.018 | -0.023 | -0.003 | -0.013 | -0.032 | 0.009 | 0.339 |
| L106 | MJX | 0.000 | 0.034 | 0.045 | 0.002 | 0.052 | 0.013 | -0.026 | 0.091 | 0.118 | 0.000 |
| L11 | MJX | 0.005 | 0.016 | 0.049 | 0.031 | -0.001 | 0.020 | 0.010 | 0.023 | -0.008 | -0.010 |
| L119 | SEF | 0.015 | 0.006 | 0.067 | -0.061 | 0.043 | 0.028 | 0.033 | -0.112 | 0.001 | 0.092 |
| L12 | MJX | -0.042 | 0.029 | 0.067 | 0.180 | -0.036 | -0.021 | -0.027 | -0.016 | 0.009 | -0.037 |
| L145 | MJX | 0.002 | 0.014 | 0.035 | -0.003 | 0.004 | -0.013 | 0.014 | -0.035 | -0.017 | -0.013 |
| L15 | MJX | 0.009 | 0.009 | 0.044 | -0.020 | 0.008 | 0.002 | 0.008 | -0.010 | 0.030 | 0.090 |
| L150 | unknown | -0.024 | 0.039 | 0.059 | 0.096 | -0.044 | 0.015 | -0.007 | 0.066 | 0.084 | -0.055 |
| L151 | MJX | 0.002 | 0.023 | 0.055 | -0.013 | 0.005 | 0.047 | 0.013 | 0.099 | 0.060 | -0.011 |
| L152 | MJX | 0.014 | 0.002 | 0.047 | -0.062 | -0.056 | 0.043 | 0.070 | 0.005 | -0.038 | 0.066 |
| L153 | MJX | -0.015 | -0.002 | 0.033 | -0.049 | -0.017 | -0.024 | 0.103 | -0.116 | -0.055 | -0.057 |
| L155 | MJX | 0.030 | -0.002 | 0.048 | -0.043 | -0.059 | 0.057 | 0.042 | 0.048 | 0.013 | -0.005 |
| L16 | unknown | -0.176 | -0.147 | -0.096 | -0.043 | 0.009 | 0.122 | -0.061 | -0.008 | -0.002 | 0.075 |
| L160 | MJX | -0.143 | -0.167 | -0.102 | -0.014 | 0.043 | 0.209 | -0.136 | -0.061 | 0.030 | -0.026 |
| L161 | MJX | 0.006 | 0.022 | 0.039 | -0.060 | -0.047 | 0.033 | 0.064 | -0.008 | 0.040 | 0.027 |
| L162 | MJX | 0.022 | 0.108 | -0.087 | -0.020 | -0.019 | 0.052 | -0.004 | -0.010 | -0.060 | 0.068 |
| L163 | MJX | 0.014 | 0.008 | 0.043 | -0.088 | -0.069 | 0.077 | 0.127 | -0.076 | -0.185 | -0.106 |
| L164 | MJX | 0.023 | 0.028 | 0.045 | -0.001 | -0.031 | 0.049 | 0.014 | 0.095 | 0.090 | -0.039 |
| L164-1 | MJX | -0.004 | 0.033 | 0.064 | 0.030 | -0.023 | 0.034 | 0.003 | 0.101 | 0.156 | -0.056 |
| L165 | MJX | -0.023 | -0.009 | 0.033 | -0.026 | -0.076 | 0.043 | 0.067 | -0.067 | -0.107 | -0.076 |
| L166 | MJX | 0.015 | 0.007 | 0.069 | -0.031 | 0.154 | -0.036 | 0.007 | -0.256 | 0.129 | -0.028 |
| L167 | MJX | 0.020 | 0.090 | -0.058 | -0.043 | -0.013 | 0.029 | 0.012 | -0.035 | -0.050 | 0.057 |
| L168 | MJX | -0.002 | 0.026 | 0.066 | -0.044 | 0.048 | 0.012 | 0.059 | -0.038 | -0.181 | -0.123 |
| L17 | MZJHN | 0.004 | 0.008 | 0.034 | -0.032 | -0.012 | 0.023 | 0.045 | -0.018 | -0.027 | 0.011 |
| L170 | MJX | -0.030 | -0.004 | 0.016 | -0.020 | 0.067 | -0.023 | -0.044 | -0.061 | 0.042 | 0.016 |
| L172 | MJX | -0.088 | 0.053 | 0.058 | 0.267 | -0.109 | -0.028 | -0.012 | -0.075 | -0.033 | 0.044 |
| L173 | MJX | 0.004 | 0.020 | 0.070 | -0.036 | -0.048 | 0.064 | 0.056 | 0.044 | 0.017 | 0.022 |
| L174 | MJX | -0.061 | -0.072 | -0.041 | -0.020 | -0.017 | 0.055 | -0.029 | -0.035 | -0.015 | 0.027 |
| L175 | MJX | 0.003 | 0.073 | 0.042 | 0.019 | -0.067 | 0.110 | 0.031 | 0.119 | 0.197 | -0.106 |
| L176 | MJX | 0.000 | 0.010 | 0.021 | -0.012 | 0.043 | -0.045 | 0.003 | 0.010 | 0.032 | 0.011 |
| L177 | MJX | 0.013 | 0.076 | -0.069 | -0.011 | 0.002 | 0.017 | 0.007 | -0.021 | -0.036 | 0.012 |
| L178 | MJX | 0.008 | 0.014 | 0.049 | -0.008 | 0.010 | 0.014 | 0.021 | 0.000 | -0.042 | 0.005 |
| L18 | MZJHN | -0.198 | -0.189 | -0.135 | -0.021 | 0.011 | 0.166 | -0.106 | -0.030 | 0.014 | 0.018 |
| L180 | MJX | -0.012 | -0.068 | 0.000 | -0.011 | 0.088 | 0.094 | -0.067 | -0.171 | 0.086 | -0.077 |
| L182 | MJX | 0.018 | 0.003 | 0.038 | -0.030 | -0.042 | 0.002 | 0.039 | -0.044 | -0.034 | 0.003 |
| L185 | MJX | 0.010 | 0.020 | 0.046 | -0.006 | -0.013 | 0.029 | 0.017 | 0.026 | 0.055 | 0.046 |
| L186 | unknown | 0.014 | 0.020 | 0.054 | -0.045 | 0.015 | 0.029 | 0.031 | -0.044 | 0.015 | 0.045 |
| L19 | MZJHN | 0.013 | -0.001 | 0.070 | -0.040 | 0.186 | -0.038 | 0.008 | -0.263 | 0.094 | 0.001 |
| L2 | MJX | 0.007 | 0.007 | 0.072 | -0.024 | 0.198 | -0.053 | -0.007 | -0.233 | 0.091 | -0.042 |
| L20 | MZJHN | 0.003 | 0.214 | -0.199 | 0.030 | -0.036 | 0.037 | 0.008 | -0.087 | -0.121 | 0.049 |
| L21 | MZJHN | -0.025 | 0.002 | 0.050 | -0.009 | -0.030 | 0.034 | 0.030 | -0.020 | -0.061 | 0.026 |
| L212 | MZJHN | 0.038 | -0.014 | 0.039 | -0.043 | -0.039 | 0.028 | 0.029 | 0.026 | -0.024 | 0.016 |
| L213 | MZJHN | -0.006 | 0.022 | 0.039 | -0.025 | 0.013 | -0.021 | 0.026 | 0.026 | 0.059 | 0.045 |
| L213-1 | MZJHN | -0.013 | 0.135 | -0.068 | 0.019 | 0.076 | 0.017 | -0.058 | 0.114 | 0.102 | -0.026 |
| L214 | MZJHN | 0.003 | -0.004 | 0.032 | -0.012 | -0.042 | 0.047 | 0.006 | 0.013 | -0.038 | 0.080 |
| L217 | MJX | -0.018 | 0.010 | 0.067 | 0.000 | -0.027 | 0.006 | -0.011 | -0.082 | 0.038 | 0.232 |
| L219 | MJX | 0.005 | -0.004 | 0.035 | -0.046 | 0.008 | 0.001 | 0.008 | 0.027 | -0.069 | 0.005 |
| L22 | MZJHN | -0.058 | 0.050 | -0.091 | -0.011 | 0.012 | 0.092 | -0.026 | 0.020 | 0.154 | -0.029 |
| L220 | MJX | -0.008 | 0.015 | 0.054 | 0.027 | 0.002 | -0.014 | 0.017 | -0.033 | -0.197 | -0.126 |
| L222 | MJX | 0.011 | 0.068 | -0.020 | 0.004 | -0.055 | 0.069 | 0.035 | 0.032 | -0.036 | -0.008 |
| L226 | MJX | -0.008 | 0.011 | 0.068 | -0.057 | -0.047 | 0.033 | 0.069 | -0.014 | 0.003 | 0.077 |
| L227 | MJX | -0.015 | 0.010 | 0.064 | 0.059 | -0.024 | -0.026 | 0.016 | 0.007 | 0.054 | 0.014 |
| L228 | MJX | 0.020 | 0.007 | 0.052 | -0.057 | -0.049 | 0.053 | 0.041 | -0.032 | -0.055 | 0.116 |
| L229 | MJX | -0.016 | 0.016 | 0.069 | 0.057 | -0.005 | -0.020 | 0.004 | 0.021 | 0.063 | 0.020 |
| L23 | MZJHN | -0.005 | 0.001 | 0.045 | -0.041 | -0.046 | 0.033 | 0.040 | -0.071 | -0.060 | 0.032 |
| L232 | MJX | 0.031 | 0.059 | -0.031 | -0.025 | -0.016 | 0.022 | -0.003 | -0.030 | -0.068 | -0.002 |
| L233 | unknown | 0.022 | 0.045 | -0.010 | 0.068 | -0.050 | 0.040 | -0.019 | -0.015 | 0.057 | -0.078 |
| L24 | MZJHN | -0.006 | 0.024 | 0.049 | -0.024 | 0.016 | 0.000 | 0.017 | 0.008 | 0.085 | 0.062 |
| L252 | MJX | -0.024 | 0.033 | 0.035 | 0.017 | 0.112 | -0.037 | -0.056 | 0.159 | 0.098 | -0.028 |
| L26 | MZJHN | 0.007 | 0.013 | 0.053 | -0.033 | 0.096 | -0.050 | -0.024 | 0.036 | -0.235 | -0.084 |
| L27 | MZJHN | -0.010 | 0.006 | 0.056 | -0.007 | 0.160 | -0.035 | -0.076 | 0.092 | -0.132 | 0.069 |
| L28 | MZJHN | 0.021 | -0.001 | 0.025 | -0.036 | 0.035 | -0.003 | 0.014 | -0.125 | 0.065 | -0.013 |
| L29 | MZJHN | -0.006 | 0.004 | 0.037 | -0.021 | 0.040 | -0.038 | 0.031 | -0.066 | 0.052 | 0.029 |
| L297 | unknown | 0.009 | 0.014 | 0.070 | -0.044 | -0.072 | 0.077 | 0.081 | 0.075 | -0.001 | 0.015 |
| L298 | unknown | -0.085 | 0.011 | 0.036 | 0.198 | -0.072 | 0.010 | -0.046 | -0.016 | 0.007 | -0.056 |
| L299 | unknown | 0.012 | 0.009 | 0.052 | -0.021 | 0.126 | -0.049 | -0.034 | -0.018 | -0.031 | -0.047 |
| L3 | MJX | -0.050 | 0.029 | 0.065 | 0.193 | -0.087 | 0.019 | -0.018 | -0.014 | 0.023 | -0.063 |
| L30 | MZJHN | 0.004 | 0.017 | 0.030 | 0.016 | 0.052 | -0.009 | -0.031 | 0.087 | 0.078 | -0.018 |
| L3001 | MZJHN | -0.107 | 0.049 | 0.063 | 0.319 | -0.062 | -0.077 | -0.029 | -0.071 | -0.045 | -0.086 |
| L3003 | MZJHN | -0.123 | -0.079 | -0.069 | 0.027 | -0.044 | -0.148 | 0.104 | 0.028 | 0.031 | -0.040 |
| L3007 | MZJHN | -0.169 | -0.126 | -0.116 | -0.045 | -0.013 | -0.046 | 0.061 | 0.033 | 0.025 | 0.022 |
| L3009 | MZJHN | -0.220 | -0.165 | -0.162 | -0.050 | 0.046 | 0.004 | -0.014 | 0.039 | -0.004 | 0.023 |
| L3011 | MZJHN | 0.029 | 0.236 | -0.251 | -0.033 | -0.025 | 0.094 | -0.018 | -0.037 | 0.084 | -0.036 |
| L3012 | MZJHN | 0.017 | 0.093 | -0.109 | -0.019 | -0.034 | -0.001 | 0.018 | -0.023 | -0.020 | 0.024 |
| L3014 | MZJHN | 0.011 | 0.130 | -0.139 | -0.016 | -0.005 | -0.002 | 0.005 | -0.031 | -0.039 | 0.045 |
| L3038 | MZJHN | 0.022 | 0.152 | -0.151 | -0.004 | 0.048 | -0.011 | -0.038 | -0.020 | -0.056 | 0.033 |
| L31 | MZJHN | 0.009 | 0.056 | -0.012 | -0.052 | -0.014 | 0.087 | 0.055 | -0.024 | 0.020 | -0.137 |
| L32 | MZJHN | -0.015 | 0.031 | 0.029 | 0.023 | 0.094 | -0.001 | -0.053 | 0.095 | 0.035 | -0.046 |
| L39 | unknown | -0.006 | 0.004 | 0.019 | -0.015 | 0.033 | -0.043 | 0.030 | 0.031 | 0.016 | 0.004 |
| L4 | MJX | 0.006 | -0.004 | 0.045 | -0.034 | -0.047 | 0.027 | 0.033 | 0.045 | 0.012 | 0.038 |
| L40 | MZJHN | -0.009 | -0.054 | 0.013 | -0.042 | -0.021 | 0.155 | 0.049 | -0.083 | -0.140 | -0.164 |
| L42 | MZJHN | 0.000 | 0.073 | -0.056 | -0.028 | 0.022 | 0.126 | -0.043 | -0.009 | 0.224 | -0.103 |
| L43 | MZJHN | 0.024 | 0.023 | 0.056 | -0.001 | -0.030 | 0.053 | 0.018 | 0.081 | 0.060 | -0.024 |
| L44 | MZJHN | -0.026 | -0.026 | 0.012 | -0.032 | -0.011 | 0.031 | -0.008 | 0.019 | 0.013 | 0.108 |
| L45 | MZJHN | 0.013 | 0.020 | 0.069 | 0.049 | -0.066 | 0.079 | 0.029 | 0.035 | -0.007 | -0.030 |
| L46 | unknown | -0.009 | 0.002 | 0.054 | -0.026 | -0.069 | 0.076 | 0.046 | 0.060 | 0.019 | 0.026 |
| L49 | MZJHN | 0.038 | -0.017 | 0.030 | -0.037 | 0.011 | 0.001 | -0.016 | -0.051 | 0.007 | -0.026 |
| L501 | MZJHN | -0.028 | 0.126 | -0.069 | 0.158 | 0.031 | -0.065 | -0.038 | -0.085 | -0.039 | -0.047 |
| L504 | MZJHN | -0.083 | 0.021 | 0.011 | 0.140 | -0.010 | -0.125 | 0.045 | 0.017 | 0.022 | -0.055 |
| L52 | MZJHN | 0.014 | 0.002 | 0.056 | -0.038 | 0.018 | 0.002 | 0.010 | -0.047 | 0.012 | 0.041 |
| L53 | MZJHN | -0.005 | 0.027 | 0.081 | -0.039 | -0.034 | 0.099 | 0.092 | 0.044 | -0.060 | -0.091 |
| L530 | MZJHN | -0.093 | -0.032 | -0.134 | -0.034 | -0.021 | -0.013 | 0.032 | -0.005 | -0.008 | 0.033 |
| L531 | MZJHN | -0.096 | -0.078 | -0.074 | -0.039 | 0.025 | -0.041 | 0.032 | 0.048 | 0.024 | 0.013 |
| L534 | MZJHN | -0.034 | -0.017 | -0.031 | -0.054 | -0.034 | -0.064 | 0.103 | -0.002 | -0.012 | -0.033 |
| L536 | MZJHN | -0.085 | -0.038 | -0.062 | -0.040 | 0.022 | -0.242 | 0.175 | 0.062 | 0.068 | -0.041 |
| L539 | MZJHN | -0.035 | 0.003 | -0.035 | -0.035 | 0.019 | -0.114 | 0.081 | 0.030 | 0.024 | -0.005 |
| L54 | MZJHN | -0.020 | -0.005 | 0.046 | -0.005 | -0.014 | 0.022 | 0.002 | -0.057 | -0.008 | 0.069 |
| L540 | MZJHN | -0.027 | 0.060 | -0.053 | 0.008 | 0.075 | -0.114 | 0.026 | 0.039 | -0.029 | -0.010 |
| L543 | MZJHN | -0.070 | -0.036 | -0.047 | -0.072 | -0.035 | -0.168 | 0.208 | 0.005 | -0.024 | -0.086 |
| L545 | MZJHN | -0.037 | 0.047 | -0.027 | -0.006 | 0.004 | -0.035 | 0.053 | 0.022 | 0.061 | -0.088 |
| L547 | MZJHN | -0.174 | -0.144 | -0.123 | -0.058 | -0.027 | -0.030 | 0.057 | 0.020 | 0.015 | 0.030 |
| L548 | MZJHN | -0.153 | -0.086 | -0.100 | 0.005 | -0.035 | -0.244 | 0.186 | 0.043 | 0.051 | -0.051 |
| L55 | MZJHN | -0.107 | 0.017 | 0.056 | 0.301 | -0.111 | -0.018 | -0.036 | -0.064 | -0.034 | -0.085 |
| L559 | MZJHN | -0.046 | -0.001 | -0.024 | -0.028 | 0.025 | -0.148 | 0.094 | 0.034 | 0.031 | -0.016 |
| L56 | MZJHN | 0.010 | 0.005 | 0.037 | -0.025 | 0.027 | 0.017 | -0.007 | 0.073 | -0.064 | 0.040 |
| L561 | MZJHN | -0.010 | 0.164 | -0.177 | -0.020 | 0.021 | -0.017 | 0.023 | -0.004 | 0.059 | -0.022 |
| L564 | MZJHN | -0.099 | -0.056 | -0.084 | -0.060 | -0.021 | -0.263 | 0.215 | 0.048 | 0.064 | -0.047 |
| L58 | MZJHN | -0.019 | -0.003 | 0.052 | -0.046 | -0.066 | 0.026 | 0.101 | -0.044 | -0.113 | -0.053 |
| L59 | MZJHN | -0.118 | -0.099 | -0.043 | 0.013 | -0.049 | 0.081 | -0.043 | -0.050 | -0.015 | 0.034 |
| L60 | MZJHN | -0.039 | 0.011 | 0.023 | 0.047 | 0.001 | -0.022 | 0.003 | 0.028 | 0.044 | 0.049 |
| L61 | MJX | -0.046 | 0.032 | 0.069 | 0.126 | -0.097 | -0.044 | -0.001 | -0.104 | -0.011 | 0.290 |
| L63 | MZJHN | 0.010 | 0.020 | 0.016 | -0.042 | -0.028 | 0.022 | 0.006 | 0.009 | 0.015 | -0.009 |
| L64 | MZJHN | 0.023 | -0.001 | 0.045 | -0.027 | 0.028 | -0.002 | -0.012 | 0.025 | -0.098 | -0.029 |
| L65 | MZJHN | 0.017 | 0.030 | 0.041 | -0.028 | -0.044 | 0.094 | 0.036 | 0.070 | -0.020 | 0.043 |
| L66 | MZJHN | 0.024 | 0.011 | 0.052 | -0.049 | -0.072 | 0.068 | 0.067 | 0.057 | -0.011 | -0.012 |
| L67 | MZJHN | 0.018 | -0.002 | 0.036 | -0.034 | 0.003 | -0.018 | 0.041 | 0.011 | 0.007 | -0.018 |
| L69 | unknown | 0.018 | 0.008 | 0.046 | -0.016 | 0.019 | 0.013 | 0.008 | 0.048 | 0.060 | 0.018 |
| L70 | MJX | 0.018 | 0.083 | -0.052 | -0.007 | 0.008 | 0.027 | -0.008 | 0.008 | 0.002 | 0.001 |
| L71 | MJX | -0.008 | 0.018 | 0.043 | 0.000 | 0.002 | 0.004 | 0.010 | 0.011 | 0.012 | 0.015 |
| L72 | unknown | 0.040 | 0.012 | 0.049 | -0.036 | -0.006 | 0.053 | -0.014 | 0.083 | 0.117 | 0.005 |
| L73 | MJX | -0.091 | 0.042 | 0.084 | 0.302 | -0.113 | -0.029 | -0.017 | -0.037 | 0.021 | -0.074 |
| L76 | MJX | 0.012 | 0.011 | 0.050 | 0.009 | 0.006 | 0.000 | 0.004 | -0.010 | 0.043 | 0.010 |
| L77 | MJX | 0.012 | 0.002 | 0.042 | -0.027 | -0.017 | 0.015 | 0.023 | -0.028 | -0.046 | -0.008 |
| L78 | MZJHN | -0.063 | 0.011 | 0.046 | 0.176 | -0.058 | -0.008 | -0.019 | -0.037 | -0.037 | -0.038 |
| L8 | MJX | -0.007 | 0.029 | 0.081 | -0.002 | -0.018 | -0.009 | -0.006 | -0.061 | 0.015 | 0.325 |
| L81 | MJX | 0.020 | -0.001 | 0.051 | -0.036 | 0.011 | -0.004 | 0.006 | -0.091 | -0.011 | -0.035 |
| L82 | MZJHN | 0.043 | 0.061 | -0.074 | -0.013 | -0.014 | 0.013 | -0.010 | -0.024 | -0.025 | 0.016 |
| L86 | MZJHN | -0.011 | 0.010 | 0.039 | 0.058 | -0.072 | 0.095 | 0.039 | 0.021 | -0.063 | -0.052 |
| L87 | MZJHN | 0.037 | 0.047 | -0.002 | -0.025 | -0.008 | 0.095 | 0.008 | 0.059 | 0.108 | -0.049 |
| L88 | MZJHN | -0.009 | 0.017 | 0.046 | 0.005 | -0.011 | 0.007 | 0.010 | 0.017 | 0.036 | -0.002 |
| L89 | unknown | 0.012 | 0.016 | 0.028 | -0.039 | -0.040 | 0.046 | 0.046 | -0.012 | -0.059 | -0.017 |
| L9 | MJX | 0.020 | 0.062 | -0.006 | -0.054 | -0.072 | 0.079 | 0.063 | -0.009 | -0.052 | 0.022 |
| L90 | MZJHN | -0.128 | -0.120 | -0.078 | -0.061 | -0.017 | 0.061 | -0.033 | 0.003 | -0.012 | 0.057 |
| L91 | unknown | 0.028 | 0.017 | 0.006 | -0.026 | -0.005 | 0.049 | 0.009 | 0.033 | 0.078 | -0.017 |
| L94 | MJX | -0.005 | 0.005 | 0.050 | -0.042 | -0.052 | 0.020 | 0.063 | 0.004 | 0.014 | 0.037 |
| L95 | MJX | 0.007 | 0.035 | 0.087 | -0.045 | -0.055 | 0.106 | 0.049 | 0.115 | 0.007 | -0.034 |
| L96 | MJX | 0.004 | -0.002 | 0.043 | -0.027 | -0.010 | 0.028 | 0.026 | -0.016 | -0.017 | 0.015 |
| L97 | MJX | 0.009 | 0.085 | -0.012 | -0.003 | 0.012 | 0.051 | -0.022 | 0.102 | 0.130 | -0.004 |
| L98 | MJX | -0.011 | 0.028 | 0.043 | 0.023 | 0.059 | -0.008 | -0.036 | 0.131 | 0.128 | -0.057 |
| L99 | MJX | 0.019 | 0.004 | 0.056 | -0.039 | -0.074 | 0.115 | 0.070 | 0.058 | -0.034 | -0.061 |
| LbendiCK | unknown | -0.005 | -0.021 | -0.021 | -0.018 | -0.022 | -0.101 | 0.070 | -0.023 | 0.033 | 0.019 |
| LDe1 | MZJHN | 0.032 | -0.013 | 0.041 | -0.026 | -0.006 | 0.002 | 0.001 | -0.038 | 0.015 | -0.046 |
| LFy1 | MZJHN | -0.028 | 0.143 | -0.122 | 0.115 | 0.001 | -0.041 | -0.032 | -0.040 | -0.044 | -0.005 |
| LFy2 | MZJHN | 0.010 | 0.234 | -0.231 | 0.001 | 0.064 | -0.001 | -0.048 | -0.022 | -0.095 | 0.065 |
| LFy5 | MZJHN | 0.015 | 0.235 | -0.260 | -0.035 | -0.024 | 0.075 | -0.007 | -0.041 | 0.039 | -0.014 |
| LFy7 | MZJHN | -0.180 | -0.155 | -0.109 | -0.050 | -0.030 | 0.126 | -0.068 | -0.021 | -0.069 | 0.041 |
| LFy9 | MZJHN | -0.017 | -0.003 | -0.002 | -0.050 | -0.028 | 0.005 | 0.068 | -0.016 | -0.064 | -0.058 |
| Lgan1 | MJX | -0.004 | 0.023 | 0.048 | -0.006 | 0.103 | -0.050 | -0.032 | 0.040 | -0.078 | -0.015 |
| Lgan12 | MJX | 0.024 | 0.019 | 0.040 | -0.004 | 0.094 | -0.021 | -0.031 | 0.047 | -0.158 | -0.072 |
| Lgan2 | MJX | 0.065 | -0.034 | 0.005 | -0.126 | -0.214 | -0.163 | -0.315 | -0.030 | 0.000 | -0.079 |
| Lgan3 | MJX | -0.012 | 0.014 | 0.033 | 0.007 | 0.077 | 0.028 | -0.021 | 0.071 | 0.005 | -0.042 |
| Lgan4 | MJX | -0.009 | 0.034 | 0.042 | -0.022 | 0.101 | 0.007 | -0.049 | 0.107 | 0.013 | -0.033 |
| Lgan5 | MJX | 0.022 | 0.005 | 0.056 | -0.029 | 0.165 | -0.046 | -0.003 | -0.233 | 0.117 | -0.052 |
| Lgan9 | MJX | 0.028 | -0.014 | 0.043 | -0.039 | 0.111 | -0.024 | 0.011 | -0.279 | 0.128 | -0.042 |
| Lganwu2 | MJX | 0.012 | -0.020 | 0.016 | 0.007 | -0.045 | -0.039 | 0.027 | 0.021 | 0.058 | -0.023 |
| Lgui1 | SWG | 0.059 | -0.038 | 0.005 | -0.118 | -0.214 | -0.153 | -0.292 | -0.038 | 0.007 | -0.080 |
| Lgui2 | SWG | 0.021 | -0.015 | 0.017 | -0.033 | -0.038 | -0.018 | 0.025 | 0.020 | 0.020 | 0.022 |
| Lgui3 | SWG | 0.055 | -0.020 | 0.009 | -0.078 | -0.130 | -0.116 | -0.209 | 0.006 | 0.007 | -0.044 |
| Lgui4 | SWG | -0.014 | 0.002 | 0.038 | -0.007 | 0.118 | -0.042 | -0.075 | 0.123 | -0.093 | 0.049 |
| Lguiruan2 | SWG | 0.036 | -0.028 | -0.007 | -0.020 | -0.012 | -0.064 | 0.024 | 0.037 | 0.023 | -0.001 |
| Lguiruan23 | SWG | 0.014 | -0.017 | -0.010 | -0.023 | -0.003 | -0.096 | 0.045 | 0.027 | 0.032 | 0.001 |
| Lguiruan3 | SWG | 0.029 | -0.022 | -0.008 | -0.019 | 0.009 | -0.091 | 0.036 | 0.044 | 0.024 | 0.005 |
| Ljiangxi23 | MZJHN | -0.072 | -0.129 | -0.057 | -0.010 | -0.004 | 0.145 | -0.016 | -0.018 | -0.091 | -0.029 |
| Lmin43 | SEF | 0.195 | -0.165 | -0.091 | 0.116 | 0.090 | 0.056 | 0.009 | -0.057 | 0.031 | -0.021 |
| Lmin48 | SEF | 0.133 | -0.107 | -0.038 | 0.035 | 0.072 | -0.060 | 0.055 | -0.116 | 0.054 | -0.026 |
| Lmin60 | SEF | 0.262 | -0.192 | -0.130 | 0.165 | 0.027 | 0.024 | 0.066 | 0.039 | -0.013 | 0.032 |
| Lminyou1 | SEF | 0.053 | -0.017 | 0.014 | -0.048 | -0.073 | -0.049 | -0.078 | 0.004 | -0.013 | 0.028 |
| Lminyou10 | SEF | 0.138 | -0.086 | -0.043 | 0.039 | -0.026 | -0.027 | -0.002 | 0.013 | -0.002 | 0.013 |
| Lminyou11 | SEF | 0.081 | -0.021 | -0.039 | -0.025 | -0.047 | -0.038 | -0.044 | -0.008 | -0.014 | 0.003 |
| Lminyou2 | SEF | -0.024 | -0.072 | -0.033 | -0.029 | 0.011 | -0.010 | -0.019 | 0.013 | 0.013 | 0.037 |
| Lminyou4 | SEF | 0.089 | -0.049 | -0.015 | 0.034 | 0.035 | -0.007 | -0.026 | 0.069 | -0.036 | 0.045 |
| Lminyou43 | SEF | 0.038 | -0.018 | 0.009 | -0.051 | -0.067 | -0.050 | -0.079 | 0.012 | -0.034 | 0.018 |
| Lminyou48 | SEF | 0.097 | 0.000 | -0.115 | 0.038 | -0.013 | -0.005 | 0.004 | -0.005 | -0.024 | 0.038 |
| Lminyou5 | SEF | 0.051 | -0.029 | 0.002 | -0.011 | -0.033 | -0.021 | -0.010 | 0.009 | 0.003 | 0.002 |
| Lminyou6 | SEF | 0.025 | -0.012 | 0.022 | -0.013 | 0.047 | -0.029 | -0.035 | 0.073 | -0.031 | 0.034 |
| Lminyou60 | SEF | -0.024 | 0.012 | 0.013 | 0.040 | -0.064 | -0.012 | 0.014 | -0.041 | -0.016 | 0.125 |
| Lminyou8 | SEF | 0.096 | 0.096 | -0.197 | 0.034 | 0.017 | -0.020 | -0.008 | -0.012 | -0.033 | 0.037 |
| Lminyou9 | SEF | 0.055 | -0.036 | 0.018 | -0.040 | -0.046 | -0.040 | -0.035 | -0.047 | 0.010 | -0.032 |
| Ltong10 | SWG | 0.007 | 0.004 | 0.032 | -0.023 | 0.014 | -0.016 | -0.002 | -0.007 | 0.016 | -0.008 |
| Ltong11 | SWG | -0.003 | 0.027 | 0.044 | -0.007 | 0.087 | -0.015 | -0.030 | 0.045 | -0.155 | -0.063 |
| Ltong12 | SWG | -0.021 | -0.006 | 0.018 | 0.014 | 0.113 | 0.076 | -0.107 | 0.098 | 0.144 | -0.060 |
| Ltong18 | SWG | -0.111 | -0.028 | 0.003 | 0.109 | 0.014 | 0.009 | -0.060 | -0.004 | -0.032 | 0.015 |
| Ltong29 | SWG | -0.017 | -0.018 | 0.034 | 0.023 | 0.135 | -0.016 | -0.095 | 0.132 | -0.070 | 0.077 |
| Ltong3 | SWG | -0.025 | 0.011 | 0.064 | 0.000 | 0.173 | -0.048 | -0.081 | 0.166 | -0.128 | 0.074 |
| Ltong30 | SWG | -0.070 | -0.058 | -0.020 | -0.007 | 0.147 | 0.046 | -0.108 | 0.073 | -0.022 | 0.008 |
| Ltong32 | SWG | 0.020 | -0.012 | 0.018 | 0.007 | 0.010 | 0.021 | -0.013 | -0.036 | 0.041 | -0.016 |
| Ltong38 | SWG | -0.006 | 0.110 | -0.101 | -0.034 | 0.064 | -0.086 | -0.040 | 0.021 | -0.135 | -0.020 |
| Ltong39 | SWG | 0.008 | 0.009 | 0.086 | -0.015 | 0.259 | -0.085 | -0.077 | -0.040 | -0.129 | -0.063 |
| Ltong4 | SWG | -0.018 | 0.015 | 0.062 | 0.000 | 0.168 | -0.041 | -0.059 | 0.067 | -0.075 | 0.033 |
| Ltong40 | SWG | -0.027 | 0.012 | 0.044 | 0.009 | 0.158 | -0.063 | -0.091 | 0.145 | -0.061 | 0.044 |
| Ltong41 | SWG | -0.006 | 0.053 | -0.015 | -0.011 | 0.051 | -0.031 | -0.069 | 0.037 | -0.075 | -0.013 |
| Ltong6 | SWG | -0.097 | -0.091 | -0.063 | -0.015 | 0.011 | 0.097 | -0.060 | -0.035 | 0.029 | -0.002 |
| Ltong9 | SWG | -0.174 | -0.077 | -0.038 | 0.154 | 0.066 | 0.037 | -0.139 | 0.025 | -0.054 | -0.030 |
| Lwu | MZJHN | 0.005 | 0.010 | 0.045 | 0.002 | -0.009 | 0.023 | 0.021 | 0.009 | -0.026 | -0.042 |
| Lwu-1 | MZJHN | 0.038 | -0.010 | 0.028 | -0.022 | 0.007 | 0.076 | 0.043 | -0.014 | -0.154 | -0.119 |
| Lwu5-1 | MZJHN | 0.065 | -0.035 | 0.008 | -0.131 | -0.225 | -0.163 | -0.315 | -0.026 | -0.011 | -0.071 |
| Lwu5-2 | MZJHN | 0.002 | -0.016 | 0.002 | -0.032 | -0.034 | -0.022 | -0.049 | 0.009 | 0.021 | 0.006 |
| Lwupai5 | MZJHN | 0.147 | -0.087 | -0.067 | 0.075 | -0.003 | -0.014 | 0.019 | 0.029 | 0.014 | 0.020 |
| Lwuyiwu2 | MZJHN | 0.015 | -0.011 | 0.012 | -0.024 | -0.028 | -0.014 | 0.000 | 0.002 | 0.009 | 0.003 |
| Lxiang3 | MZJHN | 0.258 | -0.192 | -0.128 | 0.163 | 0.021 | 0.035 | 0.070 | 0.035 | -0.019 | 0.029 |
| Lxiang4 | MZJHN | 0.083 | -0.070 | -0.043 | 0.034 | 0.000 | -0.039 | 0.038 | 0.006 | 0.034 | 0.014 |
| Lxiang47 | MZJHN | 0.032 | -0.018 | 0.049 | -0.032 | 0.046 | 0.010 | -0.008 | -0.154 | 0.055 | -0.060 |
| Lxiang5 | MZJHN | 0.059 | -0.069 | -0.013 | 0.020 | 0.030 | 0.103 | -0.027 | -0.022 | -0.006 | -0.072 |
| Lxiang50 | MZJHN | 0.156 | -0.135 | -0.067 | 0.093 | 0.003 | 0.075 | 0.047 | 0.013 | -0.056 | -0.024 |
| Lxiang6 | MZJHN | -0.024 | -0.025 | 0.007 | -0.036 | -0.025 | -0.007 | 0.028 | 0.040 | 0.018 | 0.030 |
| Lxiang7 | MZJHN | 0.033 | -0.004 | 0.027 | -0.026 | 0.003 | 0.005 | -0.023 | 0.078 | 0.062 | 0.008 |
| Lxiang8 | MZJHN | 0.003 | -0.047 | 0.012 | -0.015 | 0.070 | 0.086 | -0.076 | -0.093 | 0.057 | -0.083 |
| Lxiang9 | MZJHN | 0.031 | -0.021 | 0.001 | -0.020 | -0.047 | -0.012 | -0.006 | -0.002 | -0.001 | 0.013 |
| Lyajia1 | MZJHN | 0.002 | -0.011 | 0.018 | -0.024 | -0.052 | -0.048 | 0.016 | 0.019 | 0.042 | -0.005 |
| Lyajia2 | MZJHN | 0.076 | -0.044 | -0.002 | -0.106 | -0.198 | -0.147 | -0.269 | -0.024 | 0.008 | -0.073 |
| Lyajia6 | MZJHN | -0.007 | -0.024 | -0.018 | -0.044 | -0.038 | -0.081 | 0.048 | 0.012 | 0.010 | -0.010 |
| Lyajia8 | MZJHN | 0.165 | -0.093 | -0.085 | 0.074 | -0.007 | -0.017 | 0.031 | 0.026 | 0.012 | 0.032 |
| Lyawu1 | MZJHN | 0.018 | -0.014 | -0.006 | -0.035 | -0.031 | -0.025 | 0.009 | -0.016 | -0.006 | 0.010 |
| Lyawu2 | MZJHN | 0.067 | -0.039 | -0.008 | -0.015 | -0.042 | -0.039 | -0.003 | 0.011 | 0.016 | 0.007 |
| Lyawu4 | MZJHN | 0.237 | -0.160 | -0.102 | 0.132 | 0.047 | -0.020 | 0.018 | 0.061 | -0.023 | 0.047 |
| Lyawu6 | MZJHN | 0.013 | -0.015 | 0.007 | -0.038 | -0.044 | -0.045 | 0.005 | -0.002 | 0.007 | 0.004 |
| Lyawu7 | MZJHN | 0.227 | -0.154 | -0.106 | 0.121 | 0.010 | 0.001 | 0.039 | 0.030 | 0.007 | 0.034 |

Table S22. The first ten components in PCA results of association population

| Primer ID | Primer sequence (forword / reverse ) | Tm , ℃ | Detected SNP loci |
| --- | --- | --- | --- |
| SDP1_1 | 5'-CGTTGTTATGCCTGCTACCT-3' 5'-CCCTCTGGTCAAACTCCAC-3' | 65.1 64.7 | HiC_scaffold_6_21921279 |
|  |  |  | HiC_scaffold_6_21921481 |
|  |  |  | HiC_scaffold_6_21921487 |
|  |  |  | HiC_scaffold_6_21921722 |
| IAA26_1 | 5'-TTCCATTTGGGTTTCCTTT-3' 5'-GGCTGTGATGATTCCTTTG-3' | 59.9 61.2 | HiC_scaffold_7_51393500 |
|  |  |  | HiC_scaffold_7_51393143 |
| IAA26_2 | 5'-TGAACAACAATGGAAACCAA-3' 5'-CTTTCTTACTACCGAGGCACA-3' | 60.9 64.3 | HiC_scaffold_7_51390025 |
| FabD_1 | 5'-TTCACCATTCTCTCCTCTCTTAC-3' 5'-TCTACTGTTTCCACTCCTTTCA-3' | 63.7 63.5 | HiC_scaffold_8_39586734 |
| FabD_2 | 5'-GACGCAGCCAATGAAGAAG-3' 5'-CCAAGGACACAACCAATACAAA-3' | 64.1 63.8 | HiC_scaffold_8_39589896 |
|  |  |  | HiC_scaffold_8_39590966 |
|  |  |  | HiC_scaffold_8_39590985 |
| SAC8_1 | 5'-AAAGGCTGACATCGGTTTG-3' 5'-CAACTCCAATTCGCTCCAC-3' | 63.4 63.8 | HiC_scaffold_3_185417048 |
|  |  |  | HiC_scaffold_3_185416953 |
|  |  |  | HiC_scaffold_3_185416913 |
| SAC8_2 | 5'-CTATTGTGGCAACTCAAACCT-3' 5'-GATTGTATCTTTCCTTCTGCGT-3' | 63.3 63.2 | HiC_scaffold_3_185411128 |
| KASIII_2 | 5'-AAACGCTCCTCTTATTCCTG-3' 5'-ATTGATGCCGCACTTGTAT-3' | 62.2 62.6 | HiC_scaffold_15_17317824 |
| SAD1_1 | 5'-GATTGCGTATGTCCGTCTC-3' 5'-GATAAGTTGGCAGGGCTTC-3' | 63.0 63.2 | HiC_scaffold_10_1115361 |
|  |  |  | HiC_scaffold_10_1115176 |
|  |  |  | HiC_scaffold_10_1113856 |
| SAD1_2 | 5'-CAGCATCCATCAGCAAACT-3' 5'-GCATGTAGCACTACCGAACA-3' | 63.2 65.0 | HiC_scaffold_10_1111762 |
| SAD6_1 | 5'-CAAACATAGAGAGAGAGAGAGAGG-3' 5'-AATCTTGGTGTTGGTGGTG-3' | 63.8 63.0 | HiC_scaffold_3_55811950 |
| SAD6_2 | 5'-TACACTGCCGATGATTATGC-3' 5'-TAACCCACACACGAAGTCC-3' | 62.7 64.3 | HiC_scaffold_3_55809902 |
|  |  |  | HiC_scaffold_3_55809903 |

Table S23. Description of the Sanger sequencing primers used in our studies.

| Gene Symbol | Primer sequence (forword / reverse ) | Tm  ℃ | Amplicon length  bp |
| --- | --- | --- | --- |
| *SDP1* | 5'-ATGGCAAAGGATAGAAGTGG-3' | 62.3 | 54 |
|  | 5'-CAAATGAAATGGTGGGTGA-3' | 60.7 |  |
| *IAA26* | 5'-AACTCTCCTCTGCTGTTGATG-3' | 64.6 | 103 |
|  | 5'-ATTGCTTTCTCTGCCTCTTG-3' | 63.1 |  |
| *FabD* | 5'-AGCCAGCCAGCTATCTATGT-3' | 65.6 | 126 |
|  | 5'-AGCAGTGTATTCTCCCAAGC-3' | 64.7 |  |
| *oleosin3* | 5'-GATACCAGCAGCAGAACCAG-3' | 65.4 | 103 |
|  | 5'-GCCAGAGTCAAACCAGACAG-3' | 65.5 |  |
| *SAC8* | 5'-GTTGGTGGCAGTGGATAAA-3' | 62.7 | 188 |
|  | 5'-CTTGAATGTGGTTGAATCTCC-3' | 61.7 |  |
| *KASIII* | 5'-ACAGTGATGGTGATGGTCAA-3' | 64 | 122 |
|  | 5'-GCAGGAATAAGAGGAGCGT-3' | 64.2 |  |
| *SAD1* | 5'-CCCTCCCTCAACATCTATTTC-3' | 62.4 | 141 |
|  | 5'-CCACCCTCCTCACACATAA-3' | 63.5 |  |
| *SAD6* | 5'-CACTCAATGCCTCCTGAAA-3' | 62.4 | 124 |
|  | 5'-GGTCGGGTAAGAAATCCTG-3' | 62.2 |  |
| *ACT* | 5'-CAACTTCGCTGGTGTCTTCA-3' | 55.2 | 212 |
|  | 5'-ACCCTCTACGCAGAAGCAAA-3' | 55.8 |  |

Table S24. Description of the Real-time quantitative PCR primers used in our studies.
