# Supplementary material for: The genome of oil-Camellia and population genomics analysis provide insights into seed oil domestication
Source: Genome Biol. 2022 Jan 10;23:14. doi: 10.1186/s13059-021-02599-2 (PMC8744323; doi:10.1186/s13059-021-02599-2)
Supplement: Supplementary file 2 — Additional file 2 Fig. S1. The diploid progenitor C. oleifera “Nanyongensis” (CON) and the karyotyping of the CON plant. Fig. S2. The k-mer distribution of sequencing reads. Fig. S3. The strategy of the genome assembly based on multiple sequencing datasets. Fig. S4. Heatmap of Hi-C chromosomal interaction. Fig. S5. The SNP-based genetic map for C. oleifera using the ‘Changlin 53’ × ‘Changlin 81’ population. Fig. S6. Pearson correlation matrix for eight oil traits of C. oleifera population. Fig. S7. PCA plots of C. oleifera accessions. Fig. S8. LD levels among pairwise SNPs in seven subpopulations. Fig. S9. Genomic signatures of domestication detected by selective sweep analysis. Fig. S10. The relationship between co-expression module and ORTs in the oil-Camellia cultivar population. Fig. S11. The scatter plots for the expression profiles of eight key candidate genes by qRT-PCR analysis and RNA-seq results. Fig. S12. Combine QQ plots for eight oil traits of C. oleifera accessions. [file 13059_2021_2599_MOESM2_ESM.docx]

**Additional File 2: Supplementary Figures S1-12**


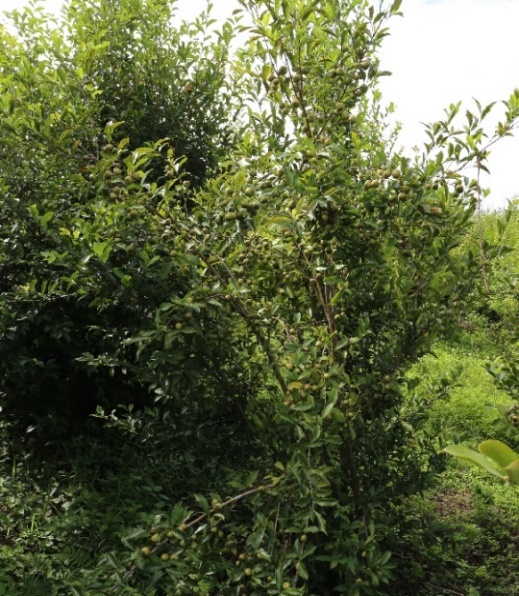


***Camellia oleifera* Abel*.* ‘Nanyongnesis’**


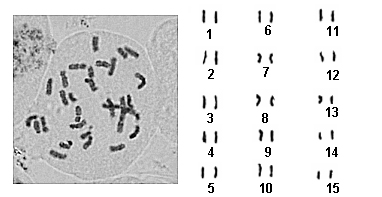


B

A

Fig S1. The diploid progenitor *C. oleifera* “Nanyongensis” (CON) and the karyotyping of the CON plant.

A. A picture of CON. B. The karyotype of CON (2n = 2x = 30) is shown on the left. The symmetry of karyotype is classified, and 15 pairs of chromosomes are identified (right).


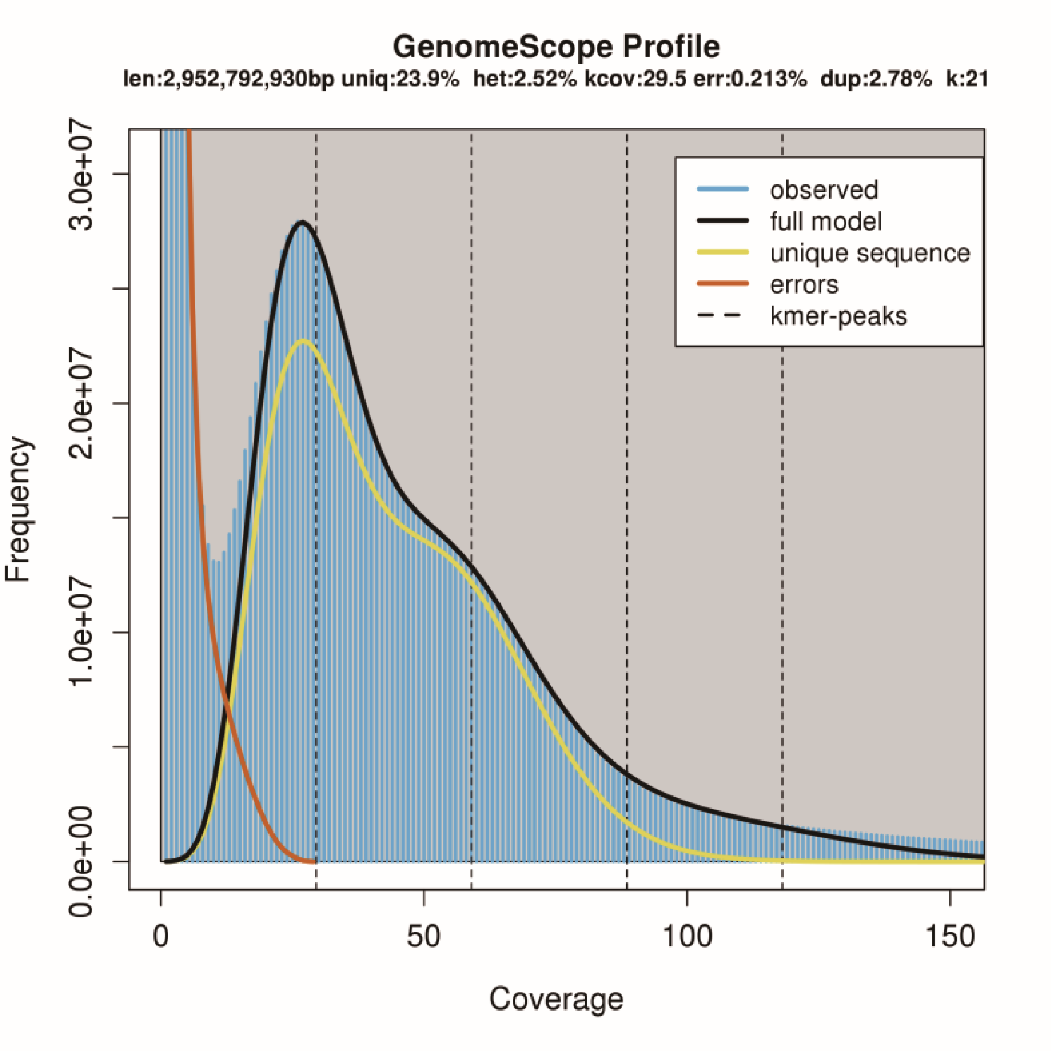


Fig S2. The k-mer distribution of sequencing reads

According to the distribution, we estimated the genome size of CON as 2.95Gb, with a 2.52% heterozygosity rate and a 76.1% repeat sequence, based an analysis of k-mer =21.


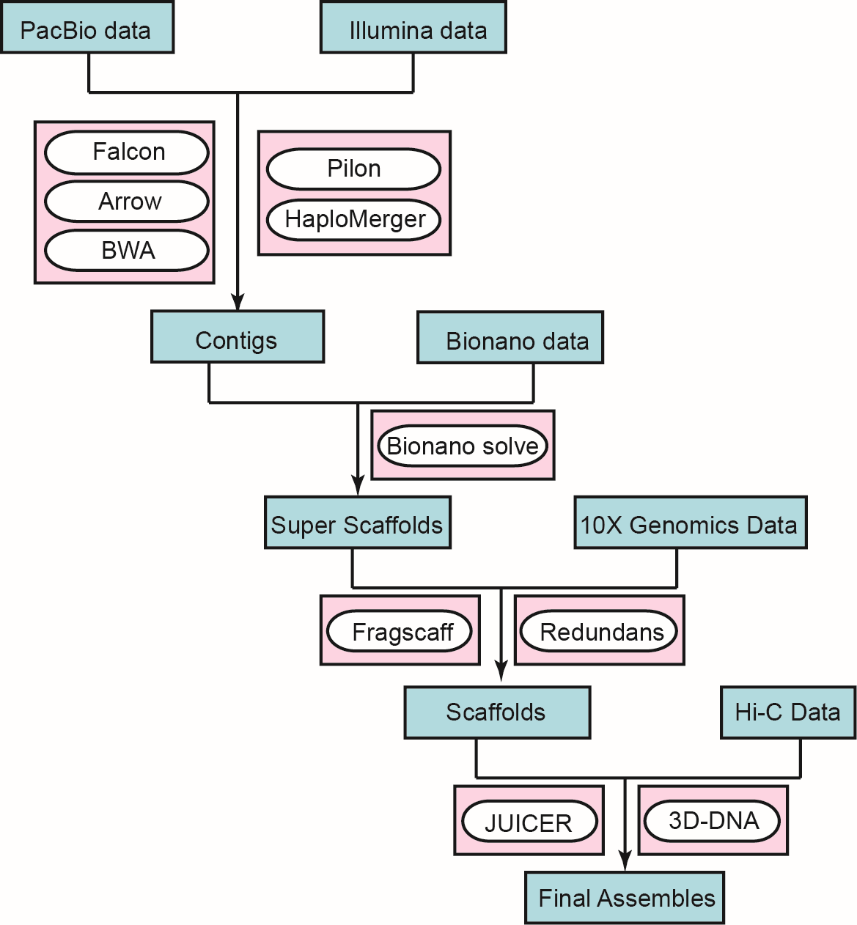


Fig S3. The strategy of the genome assembly based on multiple sequencing datasets.

The sequencing datasets are indicated in the blue boxes, and The main software are in the pink boxes. A hybrid assembly strategy is showed that combined the PacBio, Illumina, BioNano, 10X Genomics and Hi-C technologies.


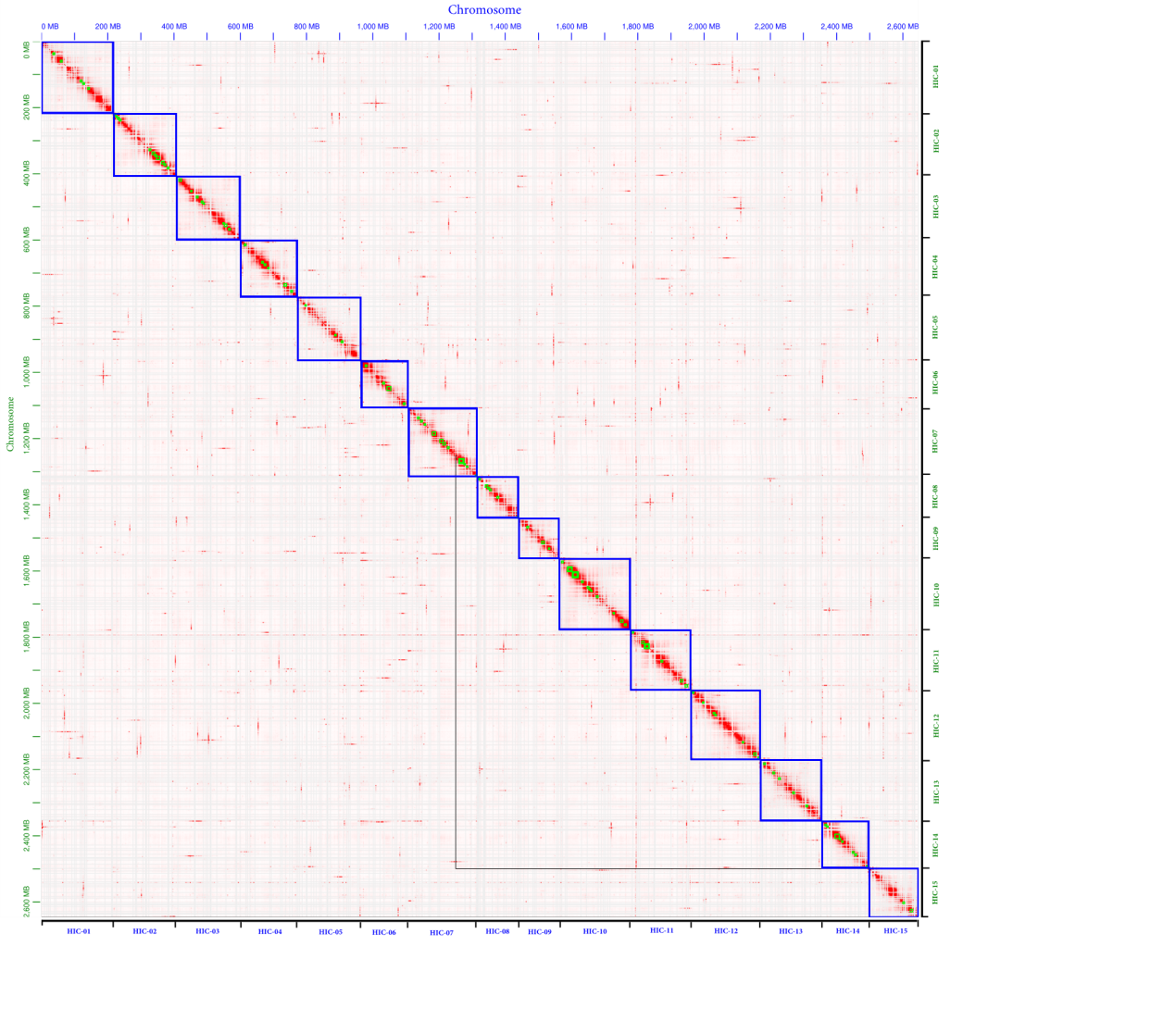


Fig S4. Heatmap of Hi-C chromosomal interaction.

Hi-C interactions among 15 chromosomes with a 100 kb resolution. Dark red indicates strong interactions and pink indicates weak interactions.


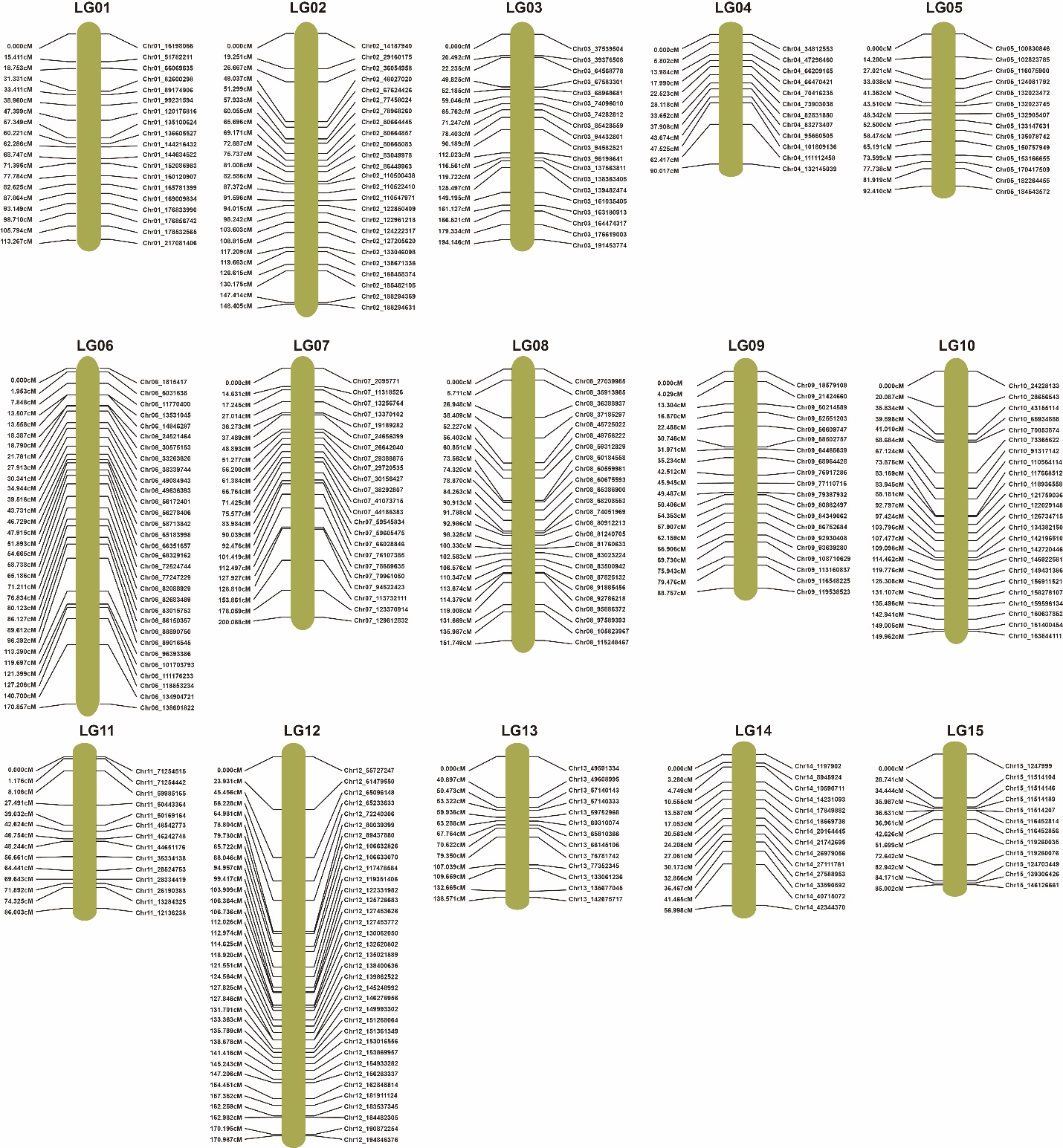


Fig S5. The SNP-based genetic map for *C. oleifera* using the ‘Changlin 53’ × ‘Changlin 81’ population.

Markers were shown on the right of the LGs, and map distances were shown on the left side.


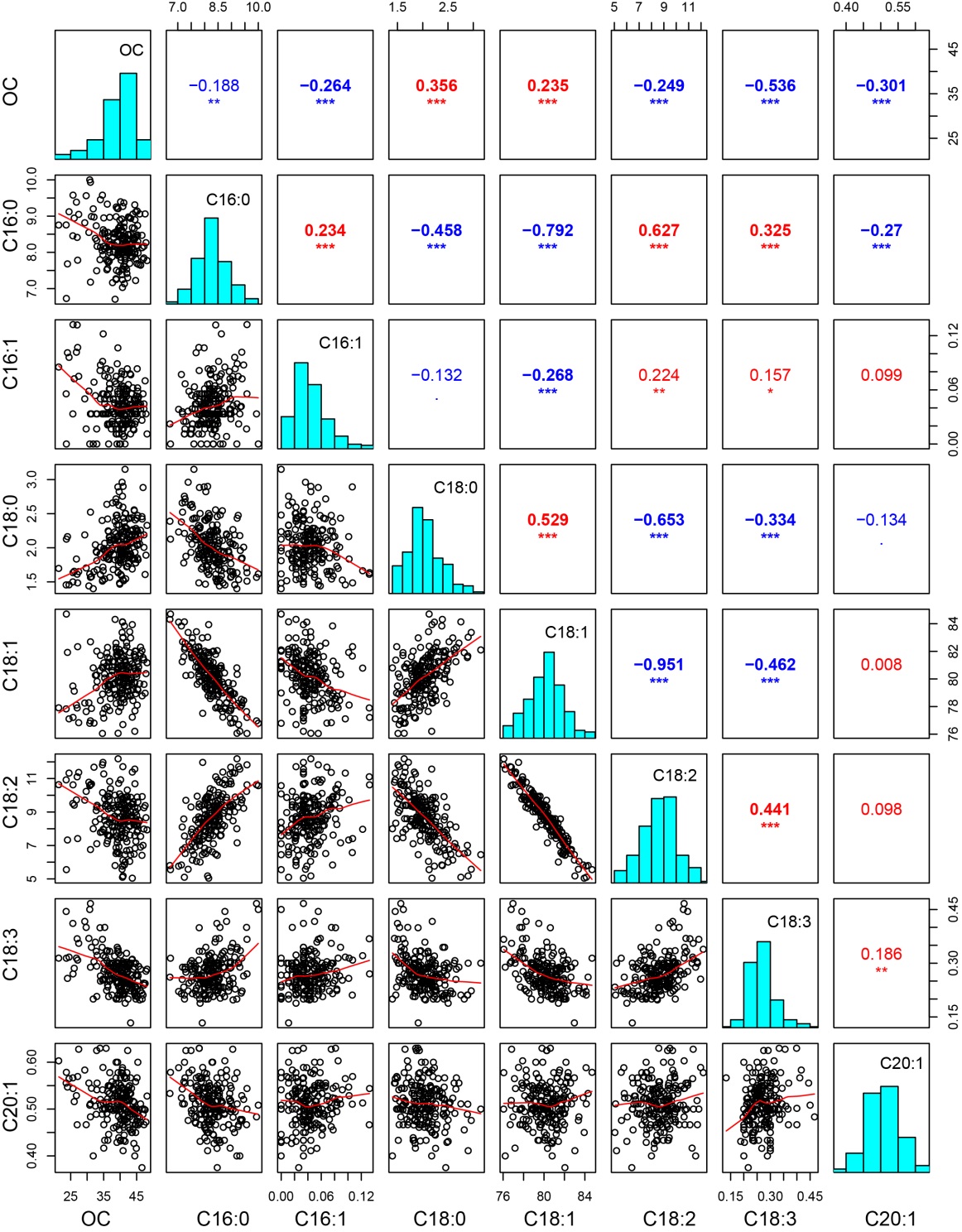


Fig S6. Pearson correlation matrix for eight oil traits of *C. oleifera* population.

The distribution of OC and seven kinds of fatty acid component contents were shown on the diagonal. To the bottom left were the bivariate scatter plots with best fit lines displayed. Correlation cofficients were shown above the diagonal. “***”, “**” and “*” denote significance with *P* values of 0.001, 0.01 and 0.05, respectively. Red and blue denote positive and negative correlations, respectively. C16:0, palmitic acid; C16:1, palmitoleic acid; C18:0, stearic acid; C18:1, oleic acid; C18:2, linoleic acid; C18:3, linolenic acid; C20:1, cis-11-eicosenoic acid


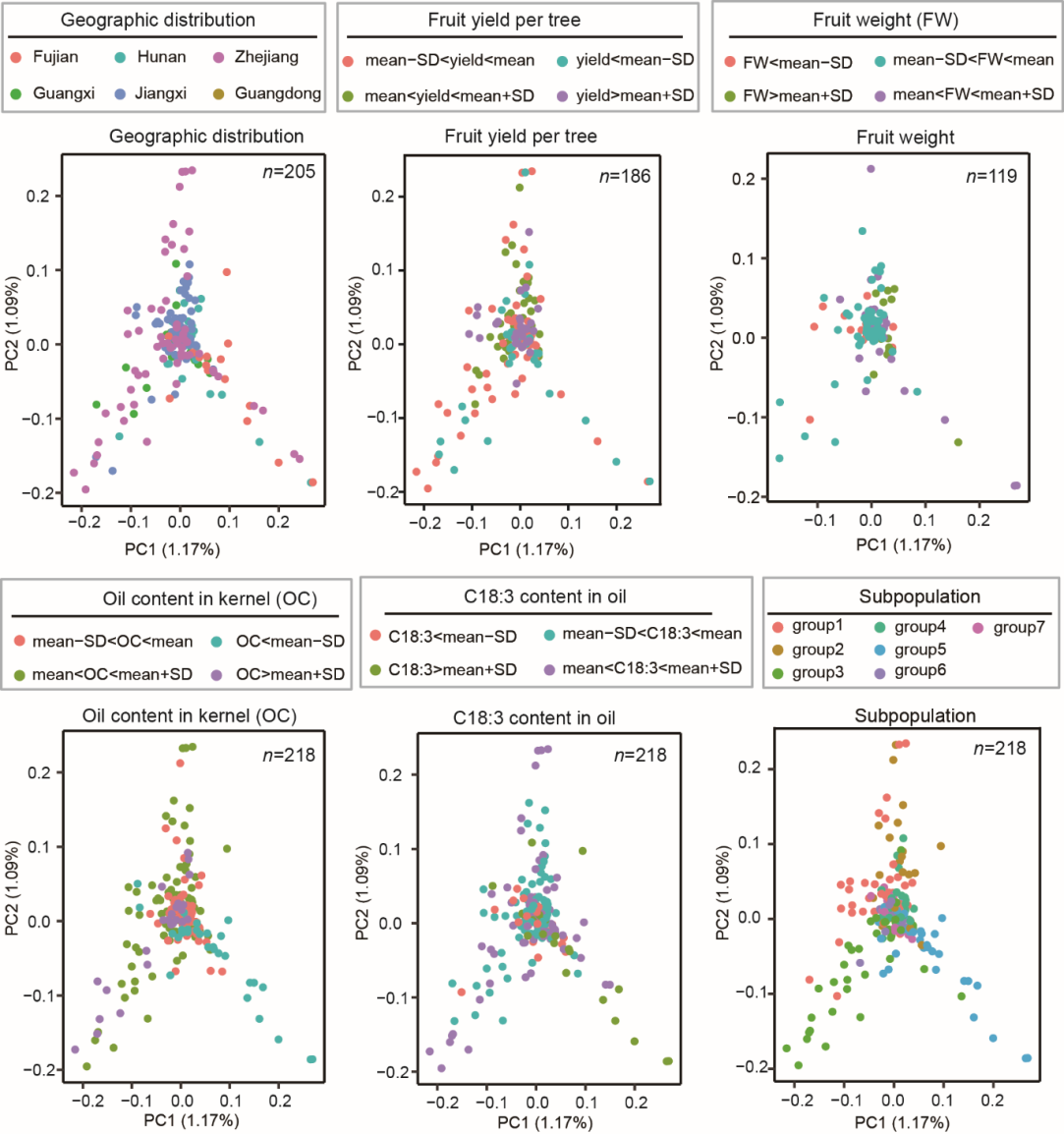


Fig S7. PCA plots of *C. oleifera* accessions.

The meanings of colors of each point were indicated by legend in the top.


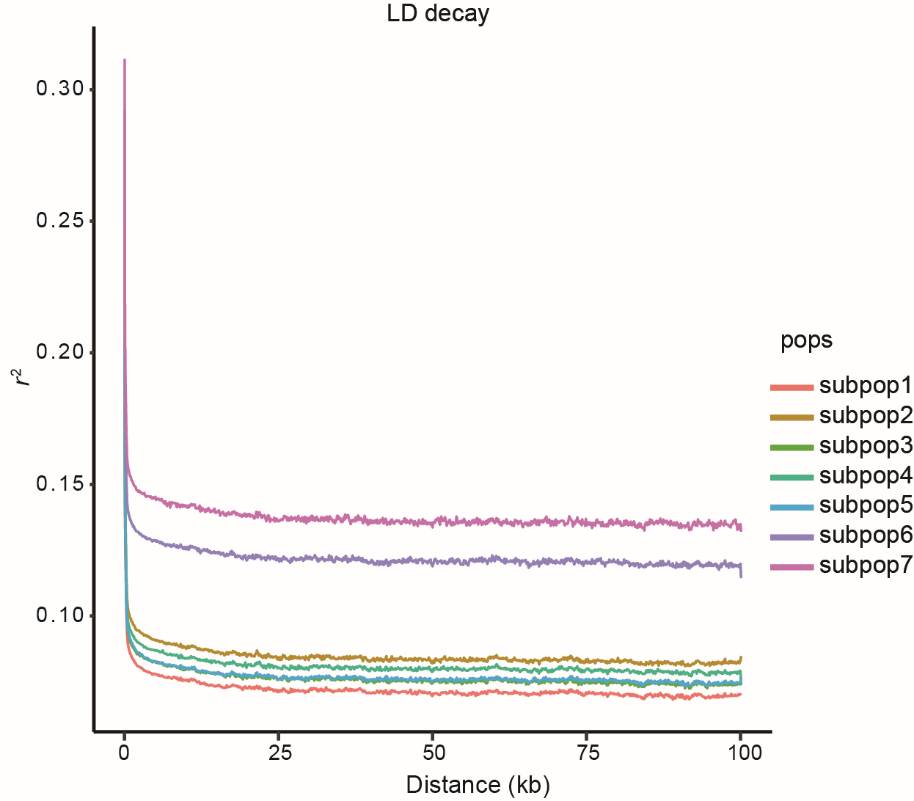


Fig S8. LD levels among pairwise SNPs in seven subpopulations.

Different colored lines show different subpopulations. Source data are provided as Data S3 [20, 21].


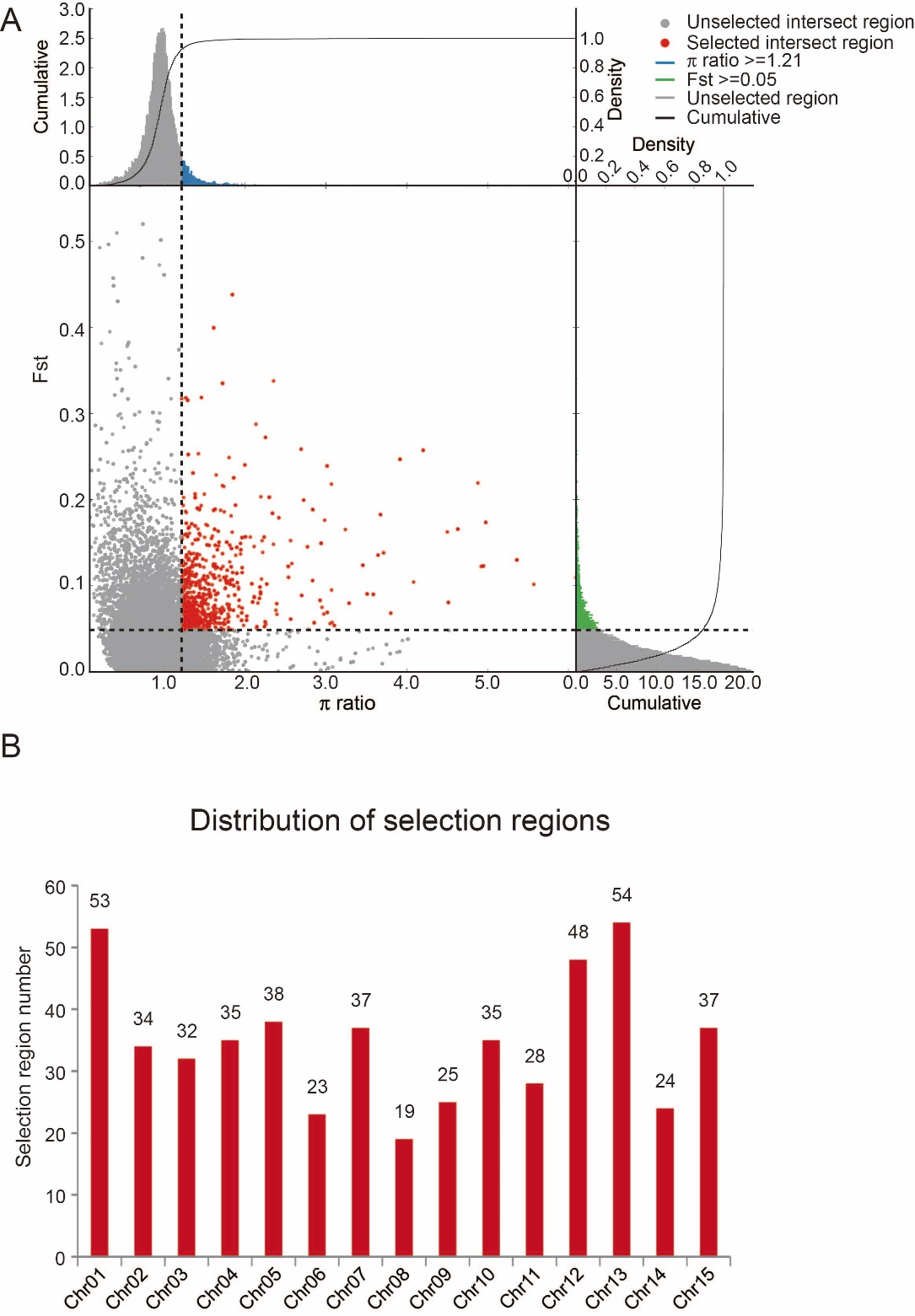


Fig S9. Genomic signatures of domestication detected by selective sweep analysis.

**A.** Scatter plot of selection windows. **B.** Distribution of selection regions on chromosomes. Source data are provided as Data S4 [20, 21].
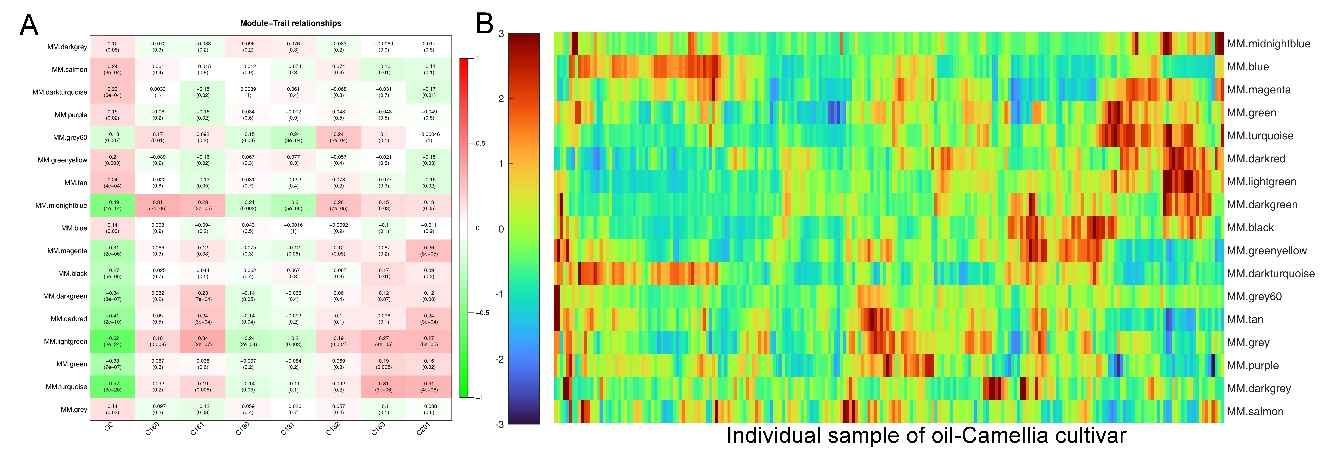


Fig S10. The relationship between co-expression module and ORTs in the oil-Camellia cultivar population.

**A**, The co-expression modules constructed from gene expression data are indicated on the left. In each block, the number represents the correlation between co-expression module and trait, which is color coded. The numbers in parentheses represent the *p*-value of the Pearson’s correlation. **B**, The eigenvalue expression of co-expression modules in each oil-Camellia cultivar.


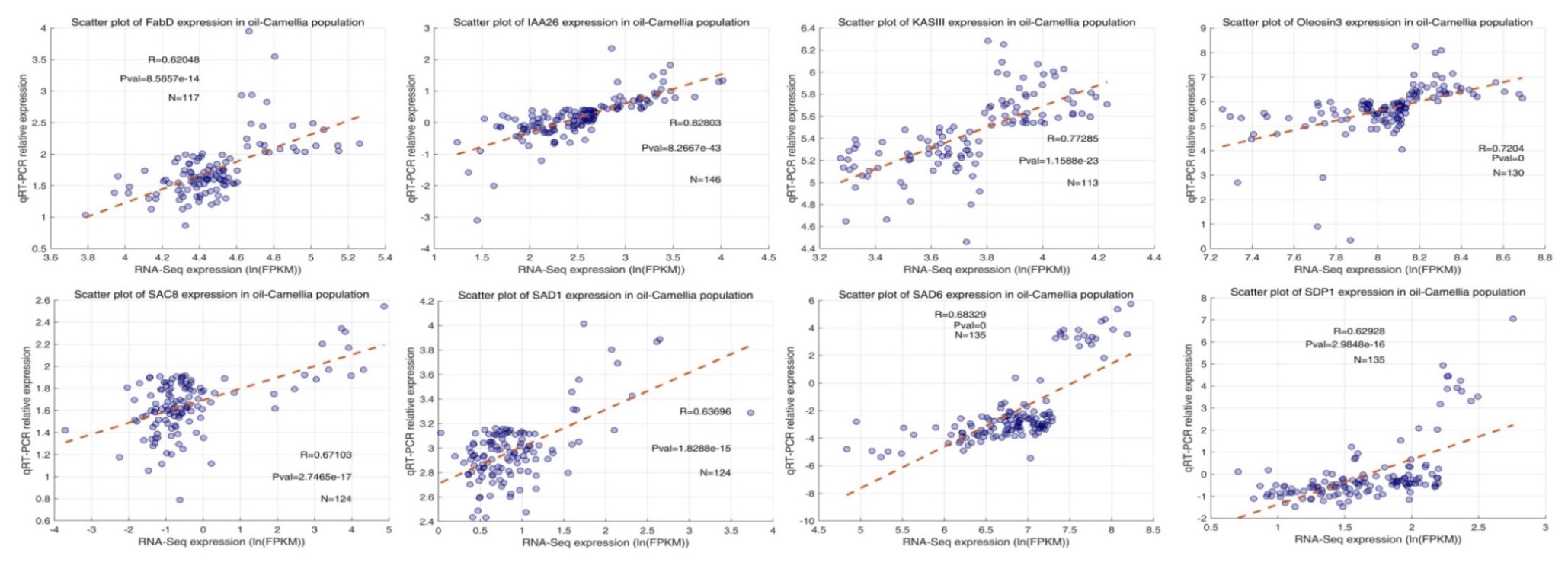

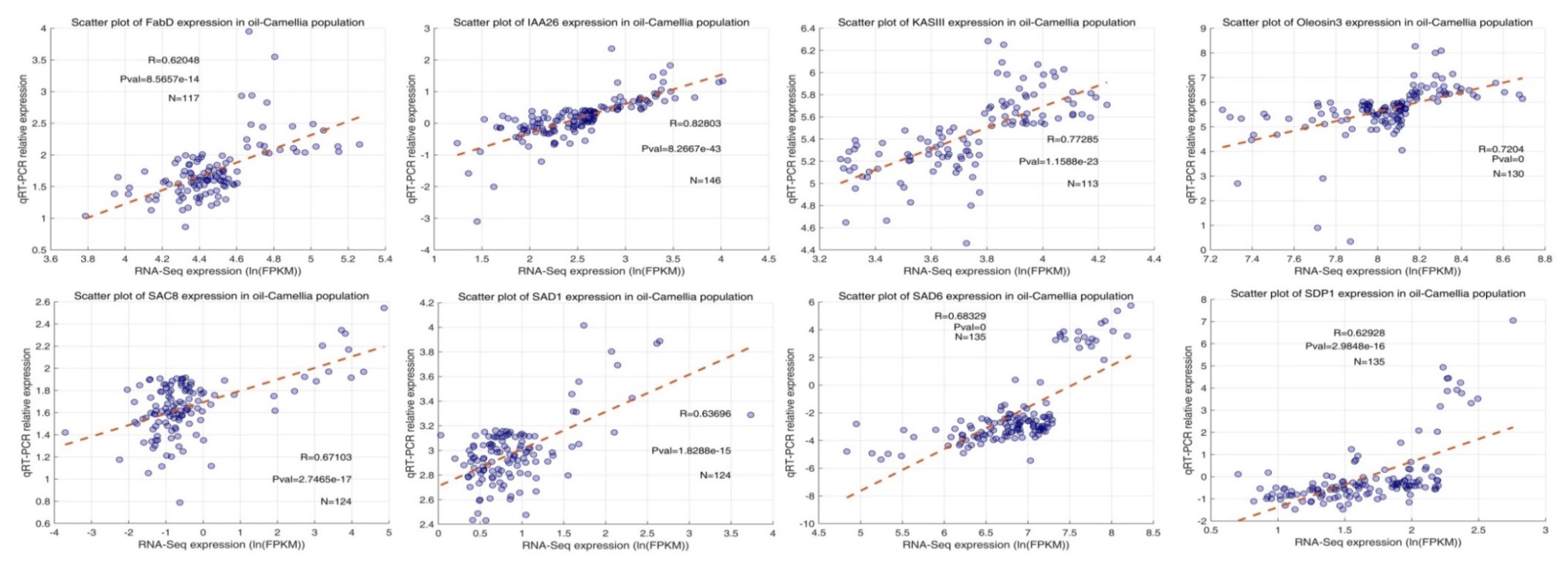

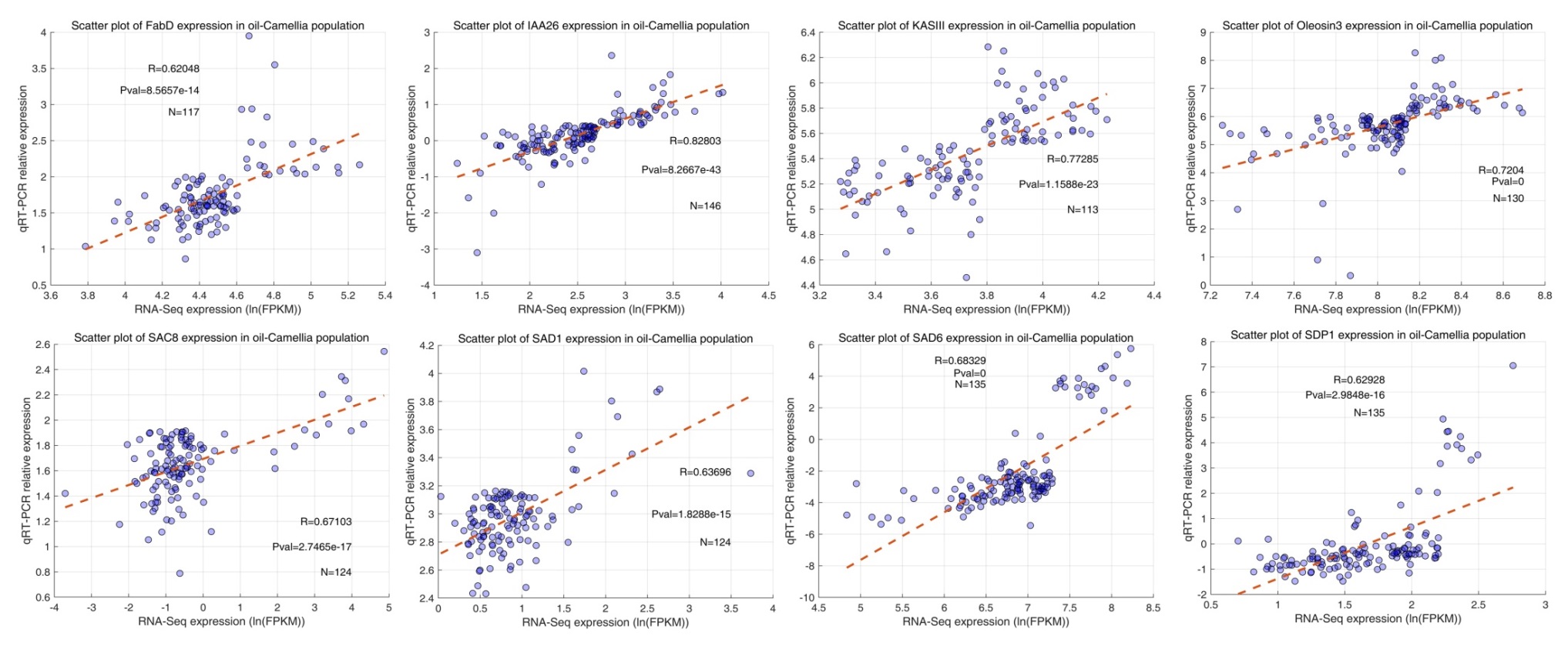

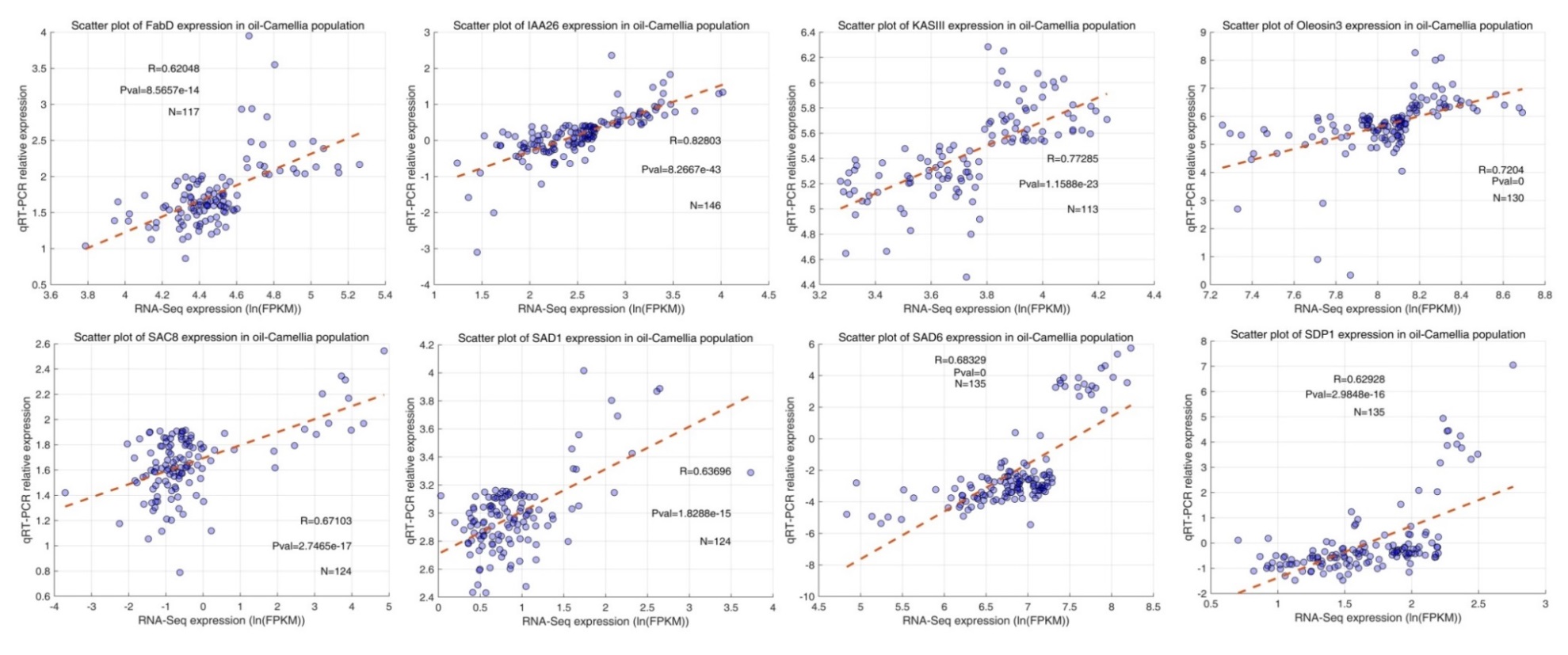


**Fig S11. The scatter plots for the expression profiles of eight key candidate genes by qRT-PCR analysis and RNA-seq results.**

The pearson’s correlation coefficiencies, p-value and number of samples are listed in each panel. The polynomial fitted trendlines are indicated by red dashed lines.


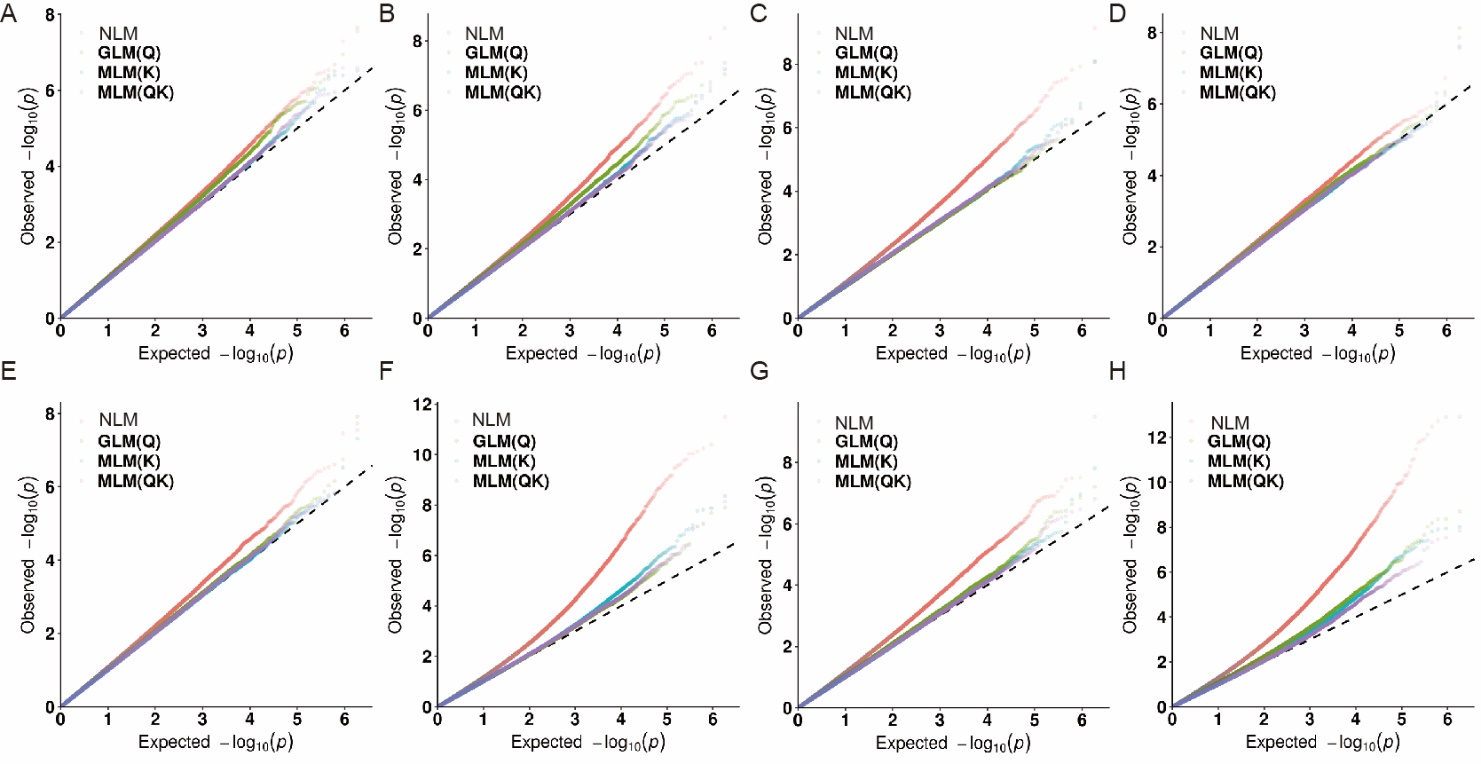


Fig S12. Combine QQ plots for eight oil traits of *C. oleifera* accessions.

Combine QQ plot of four GWAS models, NLM, QLM (Q), MLM (K) and MLM (QK), were showed for (A) palmitic acid content, (B) palmitoleic acid content, (C) stearic acid content, (D) oleic acid content, (E) linoleic acid content, (F), linolenic acid content, (G) cis-11-eicosenoic acid content and (H) OC, respectively.
