# Supplementary material for: The genome of oil-Camellia and population genomics analysis provide insights into seed oil domestication
Source: Genome Biol. 2022 Jan 10;23:14. doi: 10.1186/s13059-021-02599-2 (PMC8744323; doi:10.1186/s13059-021-02599-2)
Supplement: Supplementary file 3 — Additional file 3. Method S1. Genome sequencing and assembly. Method S2. Genome annotation and evaluation. Method S3. Double digest restriction site-associated sequencing (ddRAD) and linkage map construction. [file 13059_2021_2599_MOESM3_ESM.docx]

**Additional File 3: Supplementary methods**

**Method S1. Genome sequencing and assembly**

**Karyotype and ploidy level analysis**

Oil-Camellia, referred to over 60 species of the genus Camellia in the family Theaceae with rich oil in the kernel, is the most important edible oil-bearing tree species in south China. We collected the young leaves samples of 16 cultivated oil-camellia and wild species close to the oil-Camellia to analyze the genome ploidy level (Additional File 1: Table S1). The flow cytometry analysis of the DNA content estimation was performed according to the method by Dolezel [71] using flow cytometry analysis by a BD Accuri C6 Plus device.

A progenitor of cultivated oil-Camellia, named as “Nanyonesis” (CON) was sampled for karyotype analysis (Additional File 2: Fig S1A). The root tips (0.2-0.6 cm) of the CON plants were obtained from aseptic cuttings and pretreated with saturated aqueous solution of p-dichlorobenzene at room temperature for about 3 h. The chromosome squashing was performed according to [72]. A DP72 system (Olympus, Japan) was used for the chromosome image analysis. Images were adjusted using Adobe Photoshop and the chromosomes were counted using ImageJ software (Additional File 2: Fig S1B).

**Genome survey of CON**

High quality genomic DNA was extracted from the young leaves of CON using the TaKaRa MiniBEST Plant Genomic DNA Extraction Kit (TaKaRa, Dalian, China) according to the user manual. Genomic DNA was used to constructed three 350bp Illumina HiSeq libraries and paired-end 150bp sequencing strategy was performed for each library on Illumina NovaSeq6000 platform. After trimming the adapters, reads with more than 3% of ambiguous bases (N), reads with more than 50% bases of Q < 30 and other low quality sequences by Trimmomatic [46] (v0.32) with default parameters, about 210 Gb clean data were obtained and used for genome survey and correction of genome assembly. The 210 Gb Illumina clean data were used to estimate the genome size by Jellyfish [73] (v1.1.12) program with the parameters *kmer* = 21.

***De novo* assembly of PacBio SMRT reads**

A total of 27,876,348 PacBio SMRT subreads were used for contigs assembly by Falcon [74] (v0.3.0) program with the parameters as “length_cutoff = 10000, length_cutoff_pr = 16000” and others default parameters. The PacBio SMRT data were mapped back to the above-mentioned contigs again and Arrow program (https://github.com/PacificBiosciences/SMRT-Link) with default parameters was used for correcting the sequencing errors according to the alignments. In total, 210Gb Illumina paired-end clean reads were mapped to the above-mentioned corrected contigs using BWA [47] (v0.7.16a) with default parameters, and high-quality mapped reads (MAQ >20) were further used to polish the assembly with Pilon [75]. HaploMerger2 [76] (v20180603) program (default parameters) was used for reducing redundancy.

A hybrid assembly stategy combining PacBio, 10x Genomics, BioNano DLS optical mapping, and Hi-C mapping technologies was used to construct the high-quality reference genome. In this study, the fresh regeneration leaves from tissue culture were harvested to BioNano detection, and a total of 6,264,305 molecule maps were generated with a total length of 624.47 Gb; high quality genomic DNA extracted from the regeneration leaves from tissue culture of CON was used to 10X Genomics sequencing and 749,489,884 Linked-Reads with a total length of 224.85 Gb clean data was obtained, and 429.77 Gb high quality clean data was generated from chromosome conformation capture sequencing (Hi-C) using callus of CON.

The genome hybrid assembly was performed using a method as described in Additional File 2: Fig S3. Hybrid assembly of PacBio contigs and BioNano data was performed using Hybrid Scaffold procedure in the Bionano solve software. The procedures details are: the clean data obtained from BioNano sequencing was assembled to in silico maps; then, BioNano genome maps and PacBio contigs were aligned and merged to generate hybrid scaffolds; the PacBio contigs were mapped on the hybrid scaffolds once more to correct and generate super-scaffolds.

Further, Linked-Reads data generated from 10X Genomics platform were hybrid assembled with the super-scaffolds mentioned above using Fragscaff software. This procedure resulted in the longer super-scaffolds but with abundant ‘N’. Therefore, we performed the elimination of redundancy using Redundans [77] with the parameters of --identity 0.8, --overlap 0.894. Finally, the clean reads obtained from sequenced Hi-C libraries were mapped to draft super-scaffolds genome to group, sort and target the contigs of draft genome using JUICER [78]. And then 3D-DNA [79] (v2.0) were applied to finally evaluate and polish the assembly results. A heatmap of Hi-C chromosomal interaction was created using HiC-pro [36] software (v2.5.0) (Additional File 2: Fig S4).

**Method S2. Genome annotation and evaluation**

Evaluation of genome assembly

To investigate the quality, accuracy and completeness of the genome assembly, the mapping rate, GC content and BUSCOs data sets from the plant lineage were evaluated. 1,427,494,820 Illumina paired-end clean reads, mapped to assembly genome by SOAP aligner [80] (v2.21) program, were used for evaluation of the mapping rate, GC content and sequencing depth of genome. The results showed that over 98% reads-pairs were mapped on the genome (Additional File 1: Table S3), the GC distribution of the genome concentrated at about 35%, and the depth concentrated at about 44X. BUSCO (Benchmarking Universal Single-Copy Orthologs: http://busco.ezlab.org/) v3.0 was implemented for evaluation of the contigs genome assembly by against contigs to embryophyta_odb10 database [81].

Genome annotation

We investigated two kinds of repetitive families in the genome, including tandem repeat and interspersed repeat. Tandem repeats were identified using Tandem Repeats Finder v4.07b [82]. The interspersed repeat, mainly transposable elements (TEs), included long terminal repeat (LTR), long interspersed nuclear elements (LINE), short interspersed nuclear elements (SINE) and DNA TEs. The TEs were predicted using LTR-Finder [83], RepeatScout [84] and PILER [85] software. The predicted results were further searched against the RepBase and TE protein database using WU-BLAST to trim the known Simple repeat, Satellite and ncRNA, etc. RepeatMasker [86] and RepeatProteinMask softwares were used to shield the repetitive sequences.

Non-coding RNA includes rRNA, tRNA, snRNA, miRNA, etc. We used tRNAscan-SE [87] (v1.3.1) to *de novo* identify tRNA genes. The other types ncRNA were predicted by searching the genome assembly against the Rfam database [88] (v12.0) using BlastN [41]. In total, 641 tRNA, 970 rRNA, 97 miRNA and 611 snRNA were annotated in the whole genome (Additional File 1: Table S4).

To aid in gene annotation, RNA-seq was conducted on seven different tissues from CON (i.e., bud, young leaf, petals, stamens, pistil, young seed and mature seed). Total RNA of each sample was extracted with RNAprep Pure Plus Kit (Polysaccharides & Polyphenolics-rich, TIANGEN, Beijing, China) according to the manufacturer’s instructions. RNA-seq libraries were prepared using the Illumina standard mRNA-seq library preparation kit and sequenced on the illumina NovaSeq6000 platform with 150 bp paired-end sequencing strategy. The prediction of non-redundant protein-encoding gene models was implemented by the following strategy: first, RNA-Seq reads from seven different tissues mentioned above and the protein sequences from some relative species were mapped to reference genome to predict gene models using Maker [89] (v2.31.10). Then, we trained parameters based on the predicted gene models in transcripts-based prediction on the first step for *de novo* prediction using Augustus [90] (v3.3.1) and SNAP [91] (v2006-07-28). Finally, transcripts-based prediction and *de novo* prediction were submitted to MAKER together.

**Comparative genomics analysis**

The comparative genomics analysis mainly included intergenomic syntenic analysis among related species, whole genome duplication (WGD) analysis, phylogenetic reconstruction and gene family expansion and contraction analysis. To investigate the evolutionary trajectory of the CON genome, eight other species (*A. trichopoda*, *A. thaliana*, *V. vinifera*, *Ca. sinensis*, *Ci. sinensis*, *P. trichocarpa*, *A. chinensis* and *D.kaki*) were selected for syntenic and phylogenetic analysis: Syntenic analysis was performed by MUMmer software [43] with parameters of “nucmer-g 1000 –c 90 -11000” for CON and eight other species.

Genes of nine sequenced species (CON, *A. trichopoda*, *A. thaliana*, *V. vinifera*, *Ca. sinensis*, *Ci. sinensis*, *P. trichocarpa*, *A. chinensis* and *D.kaki*) were used for gene family clustering analysis. Gene families of orthologous genes were determined with the OrthoMCL software [92] with default settings except for the inflation factor which was set at 1.5. The input for OrthoMCL was the result of an all-versus-all BLASTP analysis of the protein sequences of eight selected plant species with the proteins of CON added as the ninth species. BLASTP was run with an *E*-value cutoff of 1 × 10^–5^ and with number of reported alignments set at 10,000. Protein clustering of the predicted CON genes with the eight other species resulted in 5,165 core gene families for all nine species, with an additional 12,076 CON-specific gene families.

A phylogenetic tree was constructed on the basis of 308 single-copy ortholog genes shared by CON and eight other plant species (*A. trichopoda*, *A. thaliana*, *V. vinifera*, *Ca. sinensis*, *Ci. sinensis*, *P. trichocarpa*, *A. chinensis* and *D.kaki*). Multiple alignments were carried out with Mafft software for each single copy gene family, and PAML [44] was used to construct the phylogenetic tree based on the ML model. The constructed phylogenetic tree was used to calculate divergence time among species. The divergence time of each tree node was inferred using Bayesian Markov-chain Monte Carlo tree (MCMCTree) package in PAML [44] with the parameters, burn-in=5,000,000, sample-number=1,000,000, sample-frequency=50. The calibration tree was generated using a fossil date for the split of *A. thaliana* and *V. vinifera* from TimeTree [93]. We also measured the expansion and contraction of orthologous gene families using the software CAFE 4.2 [94]. On the basis of the maximum likelihood modeling of gene gain and loss, we analyzed gene families for signs of expansion or contraction using genome data from mentioned above nine species.

To estimate the WGD evens in the CON genome, synonymous substitutions per synonymous site (*Ks*)-based age distributions of paralog genes were constructed, as previously described by Vanneste *et al*. [42]. Briefly, an all-against-all protein sequence similarity was performed using BLASTP with an *E*-value cutoff of 1 × 10^–10^. Then, gene families were built using the mclblastline pipeline (v10-201) [95]. *Ks* estimates for all pairwise comparisons within a gene family were obtained using Yn00 tools from the PAML package [44]. The resulting *Ks* distribution of the CON paralog genes pairs was shown in Fig. 1B. The peaks of *Ks* values identified confirmed the presence of two WGD peaks around *Ks* of 0.5 and 1.25, respectively. The same methods were implemented to estimate the *Ks* distribution in ortholog gene pairs of CON, *A. chinensis* and *D.kaki* genomes (Fig. 1B)*.*

**Method S3. Double digest restriction site-associated sequencing (ddRAD) and linkage map construction**

**Construction and sequencing of ddRAD libraries**

Tender leaf were harvested from each of the 180 *F_1_* individuals and their parents in the linkage mapping population for DNA extracted, using the TaKaRa MiniBEST Plant Genomic DNA extraction Kit (TaKaRa, Dalian, China) according to the user manual. The ddRADseq protocol was used to construct reduced representation libraries for the mapping population as described in a previous study [96]. Based on previous success in reducing genome complexity [96, 97], and the analysis of restriction enzymes digestion sites, *EcoRI* (G^AATTC) and *NlaIII* (*Hin1II*，CATG^) were selected to digest the genomic DNA samples. First, 500ng genomic DNA from each sample was double-digested using 20U *EcoRI* and *NlaIII* (New England Biolabs (NEB), Ipswich, MA, USA) in a combined reaction for 30min at 37°C. Second, the fragments were ligated to P1 and P2 adapters that include a unique 4- to 8-bp plant-specific index (barcode), and bound to the *EcoRI* and *NlaIII* overhangs, respectively. The ligation reaction was performed in a reaction volume of 40 μl using the following conditions: 37°C for 180 min, 65°C for 10min. The reaction mixture containing 500ng of DNA, 10 pmol of P1 and P2 adapters, 1U T4 DNA ligase (NEB) and 1× T4 ligation buffer. The ligation products were size-selected (400-600bp) in the 2% agarose gel, and the samples from 24 individuals were pooled together. The DNA product was subsequently purified using a Qiagen MinElute Gel Purification Kit. The ddRADseq libraries were submitted to Illumina HiSeqXten platform (Illumina, Inc., San Diego, CA, USA) and performed 150bp paired-end sequencing. Based on the Illumina raw data, the sequencing data for each individual was extracted according to the specific barcode using a custom Perl script. Only sequences that presented an exact match to a barcode, followed by the expected sequence of nucleotides after an *EcoRI* or *NlaIII* digest site were retained. The low-quality, contaminant sequences were trimmed using Trimmomatic (v0.32) [51] in two steps: (1) removal of adapters; (2) removal of reads with bases of the Phred quality threshold Q < 30.

**SNP identification and genotyping**

The clean data were mapped to the reference genome using BWA software [52] to evaluate the sequencing depth and genome cover degree for every individual. Calling of single nucleotide polymorphisms (SNPs) and insertion deletion polymorphisms (InDels) were performed using GATK (v3.7) software package based on the alignment of the clean data to the reference genome sequence in the following steps: (1) InDel realignment; (2) base recalibration; (3) variation calling, including SNPs and InDels; (4) variation data calibration. The SNPs data was further filtered to fulfill the following criteria: (1) the sequencing depth >10X in parents, >8X in F1 progeny; (2) SNP quality value Q>30; (3) miss rates (number of samples with no genotype information / number of total samples) less than 30%; (4) trimmed segregation distortion SNPs at *p*<0.01 level based on chi-square test. A total of 253,700 SNPs were identified with eight kinds of segregation patterns (Additional File 1: Table S9), and only SNP markers with the segregation patterns of lm × ll, nn × np, hk × hk and ef × eg (see description of segregations in Additional File 1: Table S9) were used for the genetic map construction based on the double pseudo-testcross strategy (Data S1 [20, 21]) [22].

**Linkage map construction**

The F1 mapping population, consisting of 180 progenies and their parents, was utilized to construct a genetic map. The JoinMap4.1 [49] was used to calculate the marker order and genetic distance. The linkage group (LG) assignments were made according the alignment result of clean reads (covered the markers) to reference genome. The regression mapping algorithm and Kosambi’s mapping function were used for marker distance calculation with the parameters of recombination frequencies ≤ 0.4, LOD ≥ 1.0 and Jump=5. A graphic representation of the map was generated using a custom perl script (https://github.com/Niuyongchao/Fish_linkage_map).

References:

71. Dolezel J, Kubaláková M, Paux E, Bartos J, Feuillet C. Chromosome-based genomics in the cereals. Chromosome Res. 2007;15(1):51–66. https://doi.org/10.1007/s10577-006-1106-x .

72. Ishikawa T, Ishizaka H. Chromosome association and giemsa c-banding of meiotic chromosomes in interspecific hybrid of alstroemeria ligtu l. hybrid and A. pelegrina L. var. rosea, its amphidiploid, and sesquidiploid between the amphidiploid and the parents. Breeding Sci. 2002;52(1):27–33. https://doi.org/10.1270/jsbbs.52.27 .

73. Liu B, Shi Y, Yuan J, Hu X, Zhang H, Li N, et al. Estimation of genomic characteristics by analyzing k-mer frequency in denovo genome projects. Arxiv Preprint Arxiv. 2013;1308.2012.

74. Chin CS, Peluso P, Sedlazeck FJ, Nattestad M, Concepcion GT, Clum A, et al. Phased diploid genome assembly with single-molecule real-time sequencing. Nat Methods. 2016;13(12):1050–4. https://doi.org/10.1038/nmeth.4035 .

75. Walker BJ, Abeel T, Shea T, Priest M, Abouelliel A, Sakthikumar S, et al. Pilon: an integrated tool for comprehensive microbial variant detection and genome assembly improvement. PLoS One. 2014;9(11):e112963. https://doi.org/10.1371/journal.pone.0112963 .

76. Huang S, Kang M, Xu A. HaploMerger2: rebuilding both haploid sub-assemblies from high-heterozygosity diploid genome assembly. Bioinformatics. 2017;33(16):2577–9. https://doi.org/10.1093/bioinformatics/btx220 .

77. Pryszcz LP, Toni G. Redundans: an assembly pipeline for highly heterozygous genomes. Nucleic Acids Res. 2016;44(12):e113. https://doi.org/10.1093/nar/gkw294 .

78. Durand NC, Shamim MS, Machol I, Rao SSP, Huntley MH, Lander ES, et al. Juicer provides a one-click system for analyzing loopresolution Hi-C experiments. Cell Syst. 2016;3(1):95–8. https://doi.org/10.1016/j.cels.2016.07.002 .

79. Dudchenko O, Batra SS, Omer AD, Nyquist SK, Hoeger M, Durand NC, et al. De novo assembly of the Aedes aegypti genome using Hi-C yields chromosome-length scaffolds. Science. 2017;356(6333):92–5. https://doi.org/10.1126/science.aal3327 .

80. Li R, Li Y, Kristiansen K, Wang J. SOAP: short oligonucleotide alignment program. Bioinformatics. 2008;24(5):713–4. https://doi.org/10.1093/bioinformatics/btn025 .

81. Simão FA, Waterhouse RM, Ioannidis P, Kriventseva EV, Zdobnov EM. BUSCO: assessing genome assembly and annotation completeness with single-copy orthologs. Bioinformatics. 2015;31(19):3210–2. https://doi.org/10.1093/bioinformatics/btv351 .

82. Gary B. Tandem repeats finder: a program to analyze DNA sequences. Nucleic Acids Res. 1999;27(2):573–80. https://doi.org/10.1093/nar/27.2.573 .

83. Zhao X, Hao W. LTR_FINDER: an efficient tool for the prediction of full-length LTR retrotransposons. Nucleic Acids Res. 2007;35(Web Server):W265–8. https://doi.org/10.1093/nar/gkm286 .

84. Price AL, Jones NC, Pevzner PA. De novo identification of repeat families in large genomes. Bioinformatics. 2005;21(suppl_1):i351–8.

85. Edgar RC, Myers EW. PILER: identification and classification of genomic repeats. Bioinformatics. 2005;21(Suppl 1):i152–8.

86. Tarailo-Graovac M, Chen N. Using Repeat Masker to identify repetitive elements in genomic sequences. Curr Protoc Bioinforma. 2009;25:4–10.

87. Lowe TM, Eddy SR. tRNAscan-SE: a program for improved detection of transfer RNA genes in genomic sequence. Nucleic Acids Res. 1997;25(5):955–64. https://doi.org/10.1093/nar/25.5.955 .

88. Griffiths-Jones S, Moxon S, Marshall M, Khanna A, Eddy SR, Bateman A. Rfam: annotating non-coding RNAs in complete genomes. Nucleic Acids Res. 2005;33(Database issue):D121–4. https://doi.org/10.1093/nar/gki081 .

89. Holt C, Yandell M. MAKER2: an annotation pipeline and genome-database management tool for second-generation genome projects. BMC Bioinformatics. 2011;12(1):491. https://doi.org/10.1186/1471-2105-12-491 .

90. Stanke M, Keller O, Gunduz I, Hayes A, Waack S, Morgenstern B. AUGUSTUS: ab initio prediction of alternative transcripts. Nucleic Acids Res. 2006;34(Web Server):W435–9. https://doi.org/10.1093/nar/gkl200 .

91. Johnson AD, Handsaker RE, Pulit SL, Nizzari MM, O'Donnell CJ, De Bakker PI. SNAP: a web-based tool for identification and annotation of proxy SNPs using HapMap. Bioinformatics. 2008;24(24):2938–9. https://doi.org/10.1093/bioinformatics/btn564 .

92. Li L. OrthoMCL: Identification of Ortholog Groups for Eukaryotic Genomes. Genome Res. 2003;13(9):2178–89. https://doi.org/10.1101/gr.1224503 .

93. Blair HS, Julie M, Michael S, Madeline P, Sudhir K. Tree of Life Reveals Clock-Like Speciation and Diversification. Mol Biol Evol. 2015;32(4):835–45. https://doi.org/10.1093/molbev/msv037 .

94. Han MV, Thomas GWC, Lugo-Martinez J, Hahn MW. Estimating gene gain and loss rates in the presence of error in genome assembly and annotation using CAFE 3. Mol Biol Evol. 2013;30(8):1987–97. https://doi.org/10.1093/molbev/mst100 .

95. Enright AJ, Dongen SV, Ouzounis CA. An efficient algorithm for large-scale detection of protein families. Nucleic Acids Res. 2002;30(7):1575–84. https://doi.org/10.1093/nar/30.7.1575 .

96. Peterson BK, Weber JN, Kay EH, Fisher HS, Hoekstra HE, Orlando L. Double Digest RADseq: an inexpensive method for de novo snp discovery and genotyping in model and non-model species. PLoS One. 2012;7(5):e37135.

https://doi.org/10.1371/journal.pone.0037135 .

97. Chen X, Li X, Zhang B, Xu J, Wu Z, Wang B, et al. Detection and genotyping of restriction fragment associated polymorphisms in polyploid crops with a pseudo-reference sequence: a case study in allotetraploid Brassica napus. BMC Genomics. 2013;14(1):346–57.

https://doi.org/10.1186/1471-2164-14-346 .
