# Supplementary material for: The genome of oil-Camellia and population genomics analysis provide insights into seed oil domestication
Source: Genome Biol. 2022 Jan 10;23:14. doi: 10.1186/s13059-021-02599-2 (PMC8744323; doi:10.1186/s13059-021-02599-2)
Supplement: Supplementary file 4 — Additional file 4. Review history. [file 13059_2021_2599_MOESM4_ESM.docx]

Review History

**First round of review**

**Reviewer 1**

**Are you able to assess all statistics in the manuscript, including the appropriateness of statistical tests used?**

Yes, and I have assessed the statistics in my report.

**Comments to author:**

The authors assemble a diploid wild oil-Camellia genome using multiple sequencing methods. The assembly looks better. This reference genome is important for oil camellia studies. Moreover, they sequenced the transcriptome of kernels from 221 cultivars. Through of GWAS and qGWAS, they identified some genetic variations that are associated with oil contents. These findings and conclusions are very important. However, I have a big concern that the analysis is lacking of quality control and validation.

1. Generally, as far as I know, about a great portion of SNPs that are identified based RNA-seq data are false positive. I would like to know how the authors deal with this problem.

2. The authors need to use sanger sequencing to validate part of SNPs.

3. In line 264-266: I recommend that the authors used qRT-PCR to validate the gene expression difference among different cultivars.

4. Line 269: As for the figure 6, the expression level of different genotype of SDP1 seem did not supported the main text described, the same as Oleosin3, SAC8, SAD1, and SAD6. Significantly different between expression level and phenotype of each allele are not consistent. High resolution images are required. Gene expression is very low and it is uncertain whether it is representative, such as SDP1, IAA26, SAC8, SAD1.

5 The authors said they investigated eight important oil traits of all accessions for three consecutive years (Table S12). However, it seems that they only show one year data.

6 There are many grammatical and expressive errors in this manuscript. Please find a professional editor to correct them.

**Reviewer 2**

**Are you able to assess all statistics in the manuscript, including the appropriateness of statistical tests used?**

No, I do not feel adequately qualified to assess the statistics.

**Comments to author:**

This study "The genome of oil-Camellia and population genomics analysis provide insights into seed oil domestication" by Lin et al. presented results from a chromosome-scale genome reference for oil-Camellia, a construction of a genetic linkage map and a population transcriptomics analysis. Indeed, the woody oilseed oil-Camellia is economically potential and important. However, this study appear to be "provide a chromosome-scale genome reference for oil-camellia as fundamental information" mentioned by authors. Overall, conclusions and insights from this study are very limited. Several experimental designs are vague. Many points or statements are difficult to understand. For instance, in the construction of a genetic linkage map, both parents Changlin 53 and Changlin 81 (both are Hexaploid), how authors applied the F1 population to define the segregation of alleles and traits among individuals of F1 population for constructing the genetic linkage map?

Indeed, this study might provide fundamental information for comparative and evolutionary genomics analyses in oil-Camellia in the future, but the scientific interest from this study is weak. Major concerns include:

1) Cultivation is NOT equivalent to domestication (if a oil-camellia seedling was transferred to plantation, we do not think this is a domestication). Authors did not show any evidences for oil-Camellia domestication based on genome data (such as genetic changes or differentiations).

2) Based on the F1 population the construction of a genetic linkage map is unbelievable.

3) For the genome-wide association studies, the population of 221 C. oleifera accessions, covering most of the natural distribution regions of C. oleifera in China, was used for transcriptome sequencing and oil traits analyses. However, the duration of seed development is great variable in different accessions. Usually expressions of lipid genes largely vary among collected samples at the different development stages (such as the early stage, middle stage and late stage of oil accumulations), resulting in that the identified genes (at the transcriptome level) is loosely associated with the oil content or fatty acid compounds.

In addition, the chromosome ploidy is unclear in the natural accessions. The expression level of a specific lipid gene is probably different in accessions with different ploidy.

**Authors Response**

**Point-by-point responses to the reviewers’ comments:**

Reviewer #1:

The authors assemble a diploid wild oil-Camellia genome using multiple sequencing methods. The assembly looks better. This reference genome is important for oil camellia studies. Moreover, they sequenced the transcriptome of kernels from 221 cultivars. Through of GWAS and qGWAS, they identified some genetic variations that are associated with oil contents. These findings and conclusions are very important. However, I have a big concern that the analysis is lacking of quality control and validation.

*Response: Thank you for the comments. In this revision, to support the key conclusions, we have performed gene expression analysis by qRT-PCR experiments and the Sanger sequencing of key SNPs. Please find detailed information in this revised manuscript.*

1. Generally, as far as I know, about a great portion of SNPs that are identified based RNA-seq data are false positive. I would like to know how the authors deal with this problem.

*Response: Thank you for the comment.*

*We agree that the identification of SNPs based on the large-scale RNA-seq data can be challenging due to the variable expression of genes and the uneven sequencing depth among individuals of the population. To ensure the SNP precision of RNA-seq based analysis, in this study, we have used a stringent filtering process, including different filters regarding read coverage and the SNP call rate in the population, to yield high-quality SNPs for downstream analyses.*

*The detailed SNP calling and filtering process is as following: (1) Variant calling was performed for all samples using “UnifiedGenotyper” function of GATK4 with option “-stand_call_conf 10.0,” “-min_base_quality_score 17” and “-stand_emit_conf 30.0” (which are the defaults values), generating one gVCF file for each sample. (2) To remove false variants, biallelic SNPs were initially extracted using the “SelectVariant” function in GATK4, and the variants were filtered using GATK’s VariantFiltration with option: “-Window 4, -filter QD < 4.0||FS > 60.0||MQ < 40.0, -G_filter GQ < 20”. (3) SNPs with minor allele frequency (MAF) < 5% and genotype call-rate < 50% in the population were discarded.*

*The filtered SNPs were further processed by PLINK software to mitigate the effect of LD. Only SNPs with heterozygosity (Het) ≤ 0.8 and Linkage disequilibrium (LD) ≤ 0.03 were used to perform Principal Component Analysis (PCA), population structure analysis and phylogenetic analysis of association population.*

*In this revision, we have added the information of the SNP calling and filtering. Please find the detailed information in Line 192-194 and Line 483-491.*

2. The authors need to use sanger sequencing to validate part of SNPs.

*Response: We agree with the comment.*

*During this revision, we have used the Sanger sequencing technology to validate the SNPs located in the eight key candidate genes (as shown in Fig. 6) in the oil-Camellia cultivars. In total, we have obtained 3781 Sanger sequences and detected 4696 successful SNP events; and 4591 SNPs were consistent with the RNA-seq data (Table S21, see below). Below are some examples of the Sanger sequences that are evaluated based on the chromatogram analysis. Please find all original sequencing data in the supplementary Data S7.*

| HiC_scaffold_6_21921279 |  |  |
| --- | --- | --- |
| Genotype | GG | GA |
| RNA-seq/Sanger | 186/185 | 27/26 |
| Verification rate | 99.46% | 96.30% |

| HiC_scaffold_8-39590966 |  |  |
| --- | --- | --- |
| Genotype | CC | CT |
| RNA-seq/Sanger | 134/134 | 79/78 |
| Verification rate | 100% | 98.73% |

*We find that all of the 23 SNPs are validated with high accuracies; the confirmation rates of SNPs are between 88.59% to 100% for each SNP, with an overall accuracy rate of 97.76% (Table S21, see below). These additional validation results indicate that the previous identification of SNPs based on the RNA-seq analysis is highly confident, which supports our main conclusions of this work. The summary of detailed sequencing results is in Table S21 and the details of primers are in Table S23. Please find revised contents in Line 285-291.*

| SNP loci | Total number | Verified | Verificati-on rate | Geno-type1 | RNA-seq | Sanger | Geno-  type2 | RNA-seq | Sanger | Geno-  type3 | RNA-seq | Sanger |
| --- | --- | --- | --- | --- | --- | --- | --- | --- | --- | --- | --- | --- |
| HiC_scaffold_6_21921279 | 213 | 211 | 99.06% | GG | 186 | **185** | GA | 27 | **26** | - | - | **-** |
| HiC_scaffold_6_21921481 | 212 | 196 | 92.45% | CC | 47 | **46** | CT | 149 | **143** | TT | 16 | **7** |
| HiC_scaffold_6_21921487 | 211 | 194 | 91.94% | AA | 17 | **4** | AG | 147 | **144** | GG | 47 | **46** |
| HiC_scaffold_6_21921722 | 213 | 211 | 99.06% | CC | 145 | **145** | CT | 68 | **66** | - | - | **-** |
| HiC_scaffold_7_51393500 | 126 | 122 | 96.83% | AA | 109 | **108** | AT | 17 | **14** | - | - | **-** |
| HiC_scaffold_7_51393143 | 212 | 210 | 99.06% | TT | 189 | **189** | CT | 23 | **21** | - | - | **-** |
| HiC_scaffold_7_51390025 | 211 | 211 | 100.00% | CT | 78 | **78** | TT | 133 | **133** | - | - | **-** |
| HiC_scaffold_8_39586734 | 199 | 197 | 98.99% | CC | 151 | **151** | CT | 48 | **46** | - | - | **-** |
| HiC_scaffold_8_39589896 | 212 | 212 | 100.00% | AT | 206 | **206** | TT | 6 | **6** | - | - | **-** |
| HiC_scaffold_8_39590966 | 213 | 212 | 99.53% | CC | 134 | **134** | CT | 79 | **78** | - | - | **-** |
| HiC_scaffold_8_39590985 | 213 | 211 | 99.06% | AT | 205 | **203** | TT | 8 | **8** | - | - | **-** |
| HiC_scaffold_3_185417048 | 213 | 211 | 99.06% | GC | 31 | **30** | GG | 182 | **181** | - | - | **-** |
| HiC_scaffold_3_185416953 | 213 | 213 | 100.00% | GA | 34 | **34** | GG | 179 | **179** | - | - | **-** |
| HiC_scaffold_3_185416913 | 213 | 206 | 96.71% | CC | 171 | **168** | CA | 42 | **38** | - | - | **-** |
| HiC_scaffold_3_185411128 | 210 | 209 | 99.52% | CC | 174 | **174** | CT | 36 | **35** | - | - | **-** |
| HiC_scaffold_15_17317824 | 213 | 213 | 100.00% | CC | 184 | **184** | CT | 29 | **29** | - | - | **-** |
| HiC_scaffold_10_1115361 | 186 | 178 | 95.70% | GT | 156 | **151** | TT | 30 | **27** | - | - | **-** |
| HiC_scaffold_10_1115176 | 213 | 211 | 99.06% | CA | 32 | **30** | CC | 181 | **181** | - | - | **-** |
| HiC_scaffold_10_1113856 | 213 | 212 | 99.53% | TT | 125 | **124** | TA | 88 | **88** | - | - | **-** |
| HiC_scaffold_10_1111762 | 149 | 132 | 88.59% | GG | 112 | **112** | GT | 37 | **20** | - | - | **-** |
| HiC_scaffold_3_55811950 | 212 | 211 | 99.53% | CC | 112 | **111** | CT | 100 | **100** | - | - | **-** |
| HiC_scaffold_3_55809902 | 213 | 212 | 99.53% | AA | 185 | **184** | AC | 28 | **28** | - | - | **-** |
| HiC_scaffold_3_55809903 | 213 | 196 | 92.02% | GT | 29 | **29** | TT | 184 | **167** | - | - | **-** |
| Total | 4696 | 4591 | 97.76% | - | - | **-** | - | - | **-** | - | - | **-** |

*Table S21. The summary of Sanger sequencing validation of SNPs identified by the RNA-seq analysis.*

3. In line 264-266: I recommend that the authors used qRT-PCR to validate the gene expression difference among different cultivars.

*Response: We agree on this comment. During this revision, we have performed the qRT-PCR analysis of the eight key candidate genes (in Fig. 6) to validate the expression profiles according to the Reviewer’s suggestions.*

*We have randomly selected the samples from the oil-Camellia cultivars for the gene expression analysis. Initially, we evaluated and filtered the quantitation results based on the amplification curve and melting curve for each PCR reaction. And we used the Actin gene as the internal reference. To calculate the expression, a standard curve of each candidate was obtained; and we performed the normalization of the relative expression using Actin among samples. Three amplification replicates were obtained; the average values were used for the correlation analysis (as shown in figure below). The results showed that all genes displayed significant positive correlations between the RNA-seq and qRT-PCR results. The pearson’s correlations ranged from 0.62 to 0.83.*

*We have modified the abovementioned the sentence in this revised manuscript (Line 291-298) and added the validated results in Figure S11. Please find the details of primers in Table S24.*

4. Line 269: As for the figure 6, the expression level of different genotype of SDP1 seem did not supported the main text described, the same as Oleosin3, SAC8, SAD1, and SAD6. Significantly different between expression level and phenotype of each allele are not consistent. High resolution images are required. Gene expression is very low and it is uncertain whether it is representative, such as SDP1, IAA26, SAC8, SAD1.

*Response: Thank you for the comment. The comment is sensible. We agree that, based on the expression level analysis, the expression abundance of SDP1, IAA26, SAC8 and SAD1 are relatively lower than the rest genes including FabD, Oleosin3, KASIII and SAD6. These results are also consistent with our qRT-PCR analysis from this revision.*

*As we have mentioned, the candidate genes in figure 6 were identified through the integrative analysis of GWAS, qGWAS and eQTL. To further evaluate the allelic variations of candidates in the cultivar population, it is necessary to reveal the effects of the SNP combinations when one candidate gene contains several SNPs. It is expected that the different SNPs might have interactive effects which influences the gene expression levels and the oil traits.*

*We examined all combinations of SNP group in the cultivar population—except for the genotypes with less than 3 individuals. We found that some genotype groups displayed significant changes in gene expression and oil traits; the inconsistence of significant differences among gene expression and oil traits was also observed, probably due to the interactive effects of the SNPs.*

*To reduce the confusions, we have modified the abovementioned statements in this revision. We also have included some discussions of using different populations to verify the elite alleles of candidates. In this revision, we have provided high-resolution images in the online reviewing system.*

*Please find the revised the contents in Line 273-277 and 360-366.*

5 The authors said they investigated eight important oil traits of all accessions for three consecutive years (Table S12). However, it seems that they only show one year data.

*Response: We are sorry for this confusion. In this study, we have used the eight oil traits that were measured for three consecutive years; Table S12 is the average number of the results. In this revision, we have provided the full data for three years in the new Table S12.*

6 There are many grammatical and expressive errors in this manuscript. Please find a professional editor to correct them.

*Response: To improve the writing of the manuscript, the authors of the manuscript have focused on the grammatical issues; we have used a commercial English editing service (Charlesworth Author Services, Paper #87744) that is based on native and professional English speakers.*

Reviewer #2:

This study "The genome of oil-Camellia and population genomics analysis provide insights into seed oil domestication" by Lin et al. presented results from a chromosome-scale genome reference for oil-Camellia, a construction of a genetic linkage map and a population transcriptomics analysis. Indeed, the woody oilseed oil-Camellia is economically potential and important. However, this study appears to be "provide a chromosome-scale genome reference for oil-camellia as fundamental information" mentioned by authors. Overall, conclusions and insights from this study are very limited.

*Response: Thank you for the comment.*

*Genus Camellia contains several important species that are extensively domesticated for producing ornaments, beverage and edible oil. As an oil crop, the domestication of oil-Camellia presents a unique research system to understand the molecular mechanism that is underlying the selection of oil biosynthesis in woody plants; on the other hand, the characterization of the genomic variations associated with the oil traits is informative to accelerate the breeding process. Please find our responses in the following paragraph.*

Several experimental designs are vague. Many points or statements are difficult to understand. For instance, in the construction of a genetic linkage map, both parents Changlin 53 and Changlin 81 (both are Hexaploid), how authors applied the F1 population to define the segregation of alleles and traits among individuals of F1 population for constructing the genetic linkage map?

*Response: We are sorry for the confusions. In this revised manuscript, we have provided sufficient information on the construction of the genetic linkage map. Please find our response in the following paragraph.*

Indeed, this study might provide fundamental information for comparative and evolutionary genomics analyses in oil-Camellia in the future, but the scientific interest from this study is weak.

Major concerns include:

1) Cultivation is NOT equivalent to domestication (if an oil-camellia seedling was transferred to plantation, we do not think this is a domestication). Authors did not show any evidences for oil-Camellia domestication based on genome data (such as genetic changes or differentiations).

*Response: Thank you for the comment. We agree with the statement of cultivation and domestication. We are sorry for the confusion.*

*The oil-Camellia possesses a long domestication history in China. The oil-Camellia cultivars used in this study are originally obtained from different areas of China; those are local cultivars that have been artificially selected over a long time. Based on the genetic structure analysis, we found that the cultivars are distantly related to the wild accessions. To enhance the domestication evidence of cultivars, in this revision, we have included the selective sweep analysis to reveal the genomic signatures of domestication. To identify candidate regions potentially affected by domestication, nucleotide diversity (π), nucleotide diversity ratio (π ratio) and population fixation index (Fst) were calculated; and we identified the domestication signals between the cultivars and wild accessions through evaluating the genomic variations using the Fst and π ratio (see Figure below). In total，we detected 2,156 selection windows, corresponding to 522 selection regions (Data S4 and Fig.S9). These genomic regions ranged in size from 100 kb to 260 kb and number from 19 to 54 among the chromosomes (Fig. S9).*

*Fig. S9 Genomic signatures of domestication detected by selective sweep analysis. (A) Scatter plot of selection windows. (B) Distribution of selection regions on chromosomes. Source data are provided in Data S4.*

*We retrieved 1000 genes from the selection regions (Data S5) and performed functional enrichment analyses. Through Gene Ontology analysis, we revealed 163 GO terms that are significantly enriched in the genes from the selection regions, including stress response, hormone biosynthesis, response to light and lipid metabolic process (Table S17). These results suggest that promotion of the oil accumulation and the stress tolerance, improvement of the developmental traits may be involved in C. oleifera domestication. Please find the details in the Line 212-223.*

2) Based on the F1 population the construction of a genetic linkage map is unbelievable.

*Response: We are sorry for the confusion.*

*The genetic linkage maps of plants are ideally constructed from the segregating populations derived from crosses between inbred lines. Due the high level of heterozygosity and time limit, those populations (e.g. the three-generation pedigrees) are generally not available in trees.*

*C. oleifera is an outcrossed and highly heterogeneous species with heterozygosity over 2%. In this work, we constructed the genetic linkage map using the F1 population by the two-way pseudo-test cross method. In the cross between two heterozygous individuals, a large proportion of gene markers are heterozygous in one parent and absent or homozygous in the other, and therefore segregate 1:1 in their F1 progeny following a testcross configuration.*

*Based on the two-way pseudo-test cross strategy, F1 populations are widely used to construct the linkage map of many woody perennials. One classic example in Eucalyptus has reported the construction of a comprehensive genetic linkage map using the RAPD makers in 1994 [1]. Since the advent of high-throughput sequencing technology, applications and refinements of this approach have been widely reported in many tree species to construct the genetic linage maps, such as sweet cherry [2], grape [3], willow [4], apple [5], Prunus mume [6], and etc.*

*To clarify the misleading, we have provided sufficient information related to the genetic linkage map in this revised manuscript. Please find the detailed segregation formats in Table S9.*

3) For the genome-wide association studies, the population of 221 *C. oleifera* accessions, covering most of the natural distribution regions of C. oleifera in China, was used for transcriptome sequencing and oil traits analyses. However, the duration of seed development is great variable in different accessions. Usually expressions of lipid genes largely vary among collected samples at the different development stages (such as the early stage, middle stage and late stage of oil accumulations), resulting in that the identified genes (at the transcriptome level) is loosely associated with the oil content or fatty acid compounds.

*Response: The comment is sensible.*

*Prior to the transcriptome sequencing of the oil-Camellia cultivars, we have performed gene expression and oil accumulation studies to reveal the critical stage of oil biosynthesis. We divided 4 developmental stages of the seed kernels and showed that the oil content, as well as the fatty acids components, displayed an S like curve, in which Stage B (294 days after fertilization) to Stage C (324 days after fertilization) represented a rapid accumulation stage of lipids [7].*

*In this work, we collected the samples at about 305 days after fertilization and selected the seed kernels based on the size of kernels and the partial browning of seed coat to mitigate the variation of developmental stages. In this revision, we have provided detailed information of sampling for the RNA-seq analysis in the Line 475-478.*

*We agree that the seed development process in different accessions may vary to cause the changes of the gene expression. To reinforce the findings of this work, we have combined the analysis of gene expression and genome-wide association of SNP to reveal the candidates for the domestication of oil traits.*

4) In addition, the chromosome ploidy is unclear in the natural accessions. The expression level of a specific lipid gene is probably different in accessions with different ploidy.

*Response: We do not have the ploidy information for all the accessions of the population at the moment. It is expected that the majority of cultivars is polyploidy. In this work, we constructed a reference genome using the wild diploid oil-Camellia plant; and it is not feasible for allelic-specific expression analysis.*

*In the current analysis of gene expression, we took the expression of all alleles as a whole for statistical analysis. In our future work, the discrimination of different alleles of key genes is a central perspective for finding the alleles that are economically significant to the domestication of oil traits.*

*Thank you.*

*References*

1. *Grattapaglia D, Sederoff R. Genetic linkage maps of Eucalyptus grandis and Eucalyptus urophylla using a pseudo-testcross: mapping strategy and RAPD markers. Genetics 1994, 137:1121-37.*
2. *Jin W, Wang H, Li M, Wang J, Yang Y, Zhang X, et al. The R2R3 MYB transcription factor PavMYB10.1 involves in anthocyanin biosynthesis and determines fruit skin colour in sweet cherry (Prunus avium L.). Plant Biotechnology Journal, 2016, 14:2120-2133.*
3. *Guo Y, Shi G, Liu Z, Zhao Y, Yang X, Zhu J, et al. Using specific length amplified fragment sequencing to construct the high-density genetic map for Vitis (Vitis vinifera L. × Vitis amurensis Rupr.). Frontiers in Plant Science, 2015, 6:393.*
4. *Hanley S, Barker A, Ooijen WV, Aldam C, Karp A. A genetic linkage map of willow ( Salix viminalis ) based on AFLP and microsatellite markers****.*** *Theoretical and Applied Genetics 2002, 105****:****1087-1096.*
5. *Jia D, Fei S, Yi W, Wu T, Han Z. Apple fruit acidity is genetically diversified by natural variations in three hierarchical epistatic genes MdSAUR37, MdPP2CH and MdALMTII. Plant Journal 2018, 95:1-14.*
6. *Zhang QX, Cheng TR, Yang WR, Pan HT, Zhong JJ. High-density genetic map construction and identification of a locus controlling weeping trait in an ornamental woody plant (Prunus mume Sieb. et Zucc). DNA Research 2015, 22(3):183-191.*
7. *Lin P, Wang K, Zhou C, Xie Y, Yao X, Yin H. Seed transcriptomics analysis in Camellia oleifera uncovers genes associated with oil content and fatty acid composition. International Journal of Molecular Science, 2018;19:118.*

**Second round of review**

**Reviewer 1**

The authors have answered all my questions and concerns, and thus recommend to accept this manuscript.

**Reviewer 2**

no comment.
